# Burden of tuberculosis and its association with socio-economic development status in 204 countries and territories, 1990–2019

**DOI:** 10.3389/fmed.2022.905245

**Published:** 2022-07-22

**Authors:** Yi Xue, Jie Zhou, Peng Wang, Jun-hong Lan, Wen-qin Lian, Yue-Ying Fan, Bei-Ni Xu, Jia-Peng Yin, Zi-hao Feng, Jian Zhou, Chi-Yu Jia

**Affiliations:** ^1^Department of Burns and Plastic and Wound Repair Surgery, Xiang'an Hospital of Xiamen University, School of Medicine, Xiamen University, Xiamen, China; ^2^School of Medicine, Xiamen University, Xiamen, China; ^3^Department of Clinical Laboratory, Shanghai Ninth people's Hospital, Shanghai Jiaotong University School of Medicine, Shanghai, China; ^4^Division of Plastic Surgery, The First Affiliated Hospital of Xiamen University, Xiamen, China; ^5^Department of Plastic Surgery, Zhongshan Hospital, Fudan University, Shanghai, China; ^6^Department of Otolaryngology, Eye Ear Nose and Throat Hospital, Fudan University, Shanghai, China

**Keywords:** tuberculosis, sociodemographic index, disability-adjusted life years, global burden of disease (GBD), incidence rate

## Abstract

**Background:**

Tuberculosis (TB) always runs in the forefront of the global burden when it comes to infectious diseases. Tuberculosis, which can lead to impairment of quality of life, financial hardship, discrimination, marginalization, and social barriers, is a major public health problem. The assessment of TB burden and trend can provide crucial information for policy decision and planning, and help countries in the world to achieve the goal of sustainable development of ending the epidemic of TB in 2030.

**Methods:**

All data are from the Global Burden of Disease 2019 (GBD 2019) database, which analyzed the burden trend of age-standardized incidence, DALYs, and deaths rate in TB and HIV/AIDS-infected TB over the past 30 years. Also, GBD 2019 not only analyzed the burden distribution of TB in 204 countries and main regions of the world but also analyzed the relationship between the burden of global TB and the socio–demographic Index (SDI).

**Results:**

The age-standardized incidence, age-standardized disability-adjusted life years (DALYs), and age-standardized deaths rate for HIV-negative TB were 10,671.45 (9,395.60–12,194.10), 59,042.45 (53,684.78–64,641.53), and 1,463.62 (1,339.24–1,602.71) (95% CI, per 100,000 person-years) in 2019, respectively. Age-standardized incidence, age-standardized DALYs, and age-standardized deaths rate of HIV/AIDS-XDR-TB (95% CI, per 1,000 person-years) were 2.10 (1.51–2.90), 64.23 (28.64–117.74), and 1.01 (0.42–1.86), respectively. We found that TB is inversely proportional to SDI, the age-standardized incidence, DALYs, and deaths rate low burden countries were in high SDI areas, while high burden countries were in low SDI areas. The global TB showed a slow decline trend, but the age-standardized incidence of HIV-positive TB was increasing, and mainly distributed in sub-Saharan Africa.

**Conclusion:**

Age-standardized incidence, age-standardized DALYs, and age-standardized deaths rate of TB is related to SDI, and the burden of low SDI countries is lighter than that of high SDI countries. Without effective measures, it will be difficult for countries around the world to achieve the goal of ending the TB epidemic by 2030. Effective control of the spread of TB requires concerted efforts from all countries in the world, especially in the countries with low SDI, which need to improve the diagnosis and preventive measures of TB and improve the control of HIV/AIDS-TB.

## Introduction

Tuberculosis is an ancient disease. In 1882, Robert Koch announced the discovery of the main bacterium of TB and named it Mycobacterium TB (1). Tuberculosis is caused by the Bacillus Mycobacterium TB, which is mainly transmitted through air. About one-fourth of the world's population is infected with Mycobacterium TB (2). Over the past 25 years, TB has been the leading cause of global adult infectious diseases and has been regarded as a worldwide public health emergency (3). Tuberculosis is the leading cause of death (single infectious source disease), ranking higher than HIV/AIDS, and is one of the 10 leading causes of death worldwide (2). Tuberculosis is a disease of poverty. People affected by TB often face financial difficulties, marginalization, and discrimination (2). According to WHO estimates, in 2019, there were 10.0 million (8.9–11.0) TB cases in the world, about 8.2% of them were HIV infected patients, about 1.2 million (1.1–1.3) of HIV-negative TB patients died, and 208,000 (177,000–242,000) HIV-positive TB patients died (2).

Although TB has dropped from No. 7 position in 1990 (all ages) [3.1 (2.8–3.4), percentage of DALYs] to the No. 20 position in 2019 [1.9(1.7–2.0), percentage of DALYs] (4). However, it is estimated that the global incidence of TB is slowly decreasing at a rate of 1.6% per year, which is far from reaching the WHO target of 4–5% (5). If the current trend continues, it will be difficult to achieve the 2030 agenda of Sustainable Development Goals (SDGs). Including the goal of ending the TB epidemic, and reducing the number of TB deaths by 90% by 2030, and reducing the incidence of TB by 80% compared with 2015 (including new and relapse cases per 100,000 per year) (2). Evaluating the progress of this goal can provide intuitive and important information for future policy formulation. Many low- and middle-income countries lack high-quality data and high-quality health testing systems. Accurately assessing the burden of TB poses certain challenges (6). The drug-resistant TB not only poses a huge challenge to global TB prevention and control but also is one of the important burdens of TB (7). Individuals who have received the previous treatment are still at high risk of recurrent TB. Follow-up after the treatment and secondary preventive treatment can accelerate the decline in the incidence of TB, and follow-up after the treatment is also very important (8). In addition, innovations in patient-centered strategies and advances in testing and treatment are also critical to ending the TB epidemic (9, 10). Therefore, the prevention and control of TB require joint efforts of prevention, diagnosis, treatment, and follow-up after treatment.

This study can provide the latest data on the age-standardized incidence, DALYs, and mortality of global TB through GBD 2019 data, which is of great significance for the prevention and control of tuberculosis. Combined with age-standardized incidence rates of TB, the association of DALYs and deaths rate analyses with SDI was more reflective of the impact of TB on socioeconomic status. Moreover, the GBD 2019 database can analyze the changes of TB in 204 countries and regions in the past 30 years (1990–2019). The burden of TB is related to many factors, such as gender, location, AIDS, and drug resistance. Analyzing the correlation between TB and these factors through the GBD 2019 database system and updating relevant information in a timely manner will help control the burden of TB and epidemiological trends.

## Methods

### Overview and data sources

The GBD database is a systematic and scientific work aimed at quantifying the comparative magnitude of health losses caused by diseases, injuries, and risk factors by age, gender, and geographical location. The latest data for estimating age-standardized incidence rate, DALYs rate, and deaths rate of TB were extracted from GBD 2019 (http://ghdx.healthdata.org/gbd-2019). The GBD 2019 includes 369 diseases and injuries in 204 countries or regions around the world as well as more than 80 behavioral, environmental, and other risk factors, whose estimation of attributable burden followed the general framework established for the comparative risk assessment (CRA) (11, 12) used in GBD since 2002. According to the GBD world population, an age-standardized ratio analysis per 100,000 person-years was recorded.

### Disability-adjusted life years

The disability-adjusted life years (DALYs) was proposed by Global Burden of Disease to measure the disease burden, whose calculation takes into account the sum of years of life lost (YLL) and years of disability (YLD) for each reason, age, location, duration, gender, and year (11, 13). There has been a detailed description elsewhere for the GBD method used to estimate mortality and DALYs (14, 15).

### Socio-demographic index

The SDI (http://ghdx.healthdata.org/gbd-2019) is a comprehensive indicator that reflects the social conditions and population, including per capita income, average years of education, and total fertility rate. The SDI score ranges from 0 to 1, which means that the correspondence relationship from the lowest income, the lowest average years of education, and the highest fertility rate to the highest income, the highest average years of education, and the lowest fertility rate, and each location is assigned an SDI score every year. The SDI was developed for GBD 2015 (16) and updated for GBD 2016 (14, 17). As for GBD 2017, due to the U-shaped pattern of age-specific fertility, the age of total fertility was changed to below 25 years. Similar to GBD 2017, GBD 2019 divides countries and regions into five levels according to SDI: High SDI (>0.81), high-middle SDI (0.70–0.81), middle SDI (0.61–0.69), low-middle SDI (0.46–0.60), and Low SDI (<0.46) (18).

### Uncertainty analysis

We adopted the same techniques used elsewhere in the GBD study design to propagate uncertainty (19–21). For all steps, we calculated uncertainty for estimation of incidence, DALYs, and deaths systematically by generating 1,000 draws. The uncertainty was determined from the sampling error and the uncertainty of the model coefficients. We used Software Prism 8.0.1 and Adobe Photoshop 20.0.0 for drawing related graphics. The *p* < 0.05 (two tailed) was statistically significant. When reporting uncertainty intervals, the 95% UIs were calculated using the 2.5th and 97.5th percentiles of the draw-level values.

## Results

### Burden of major types of TB

**Figure 1:**
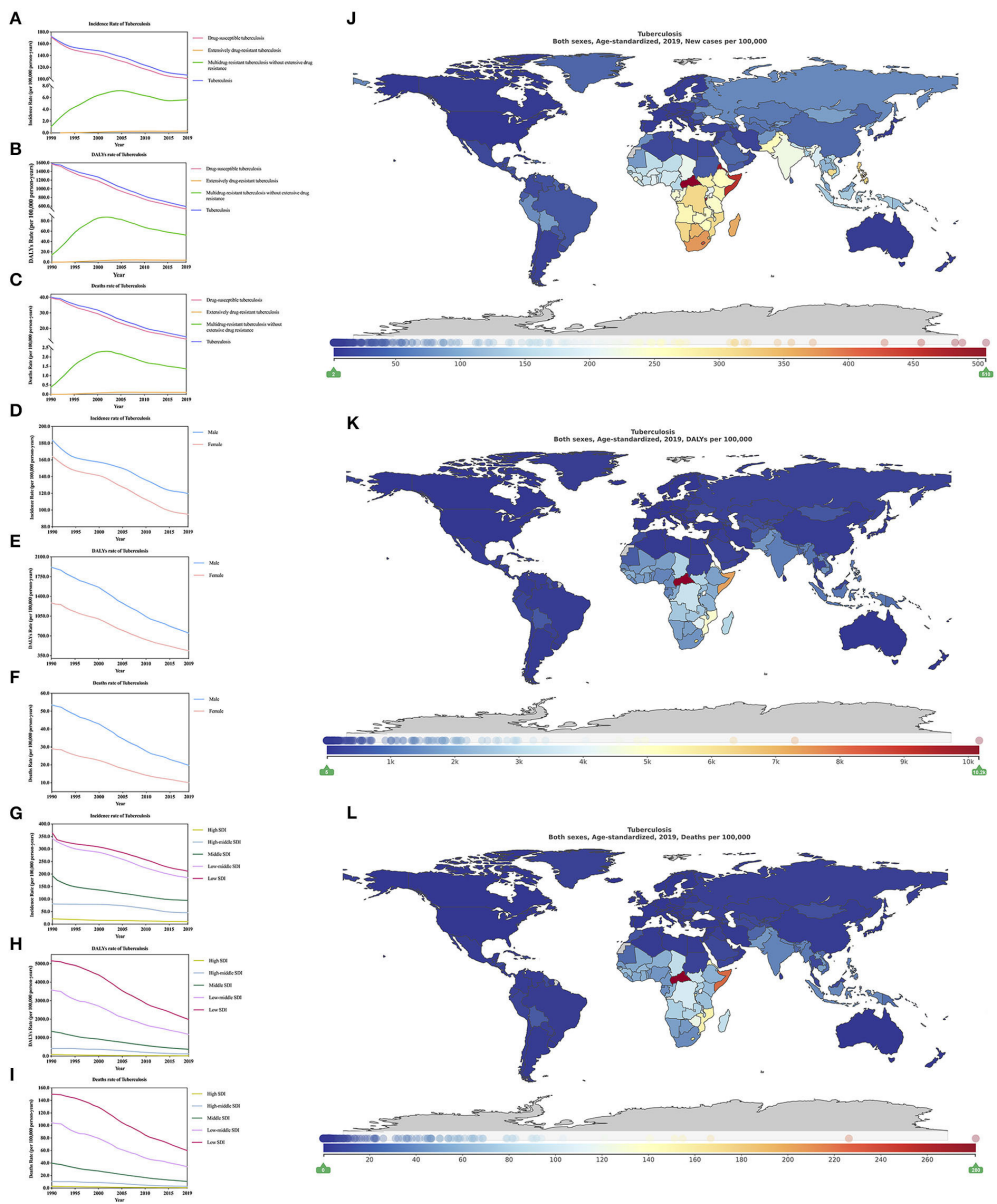
Burden of all HIV-negative tuberculosis for 204 countries and territories. Age-standardized incidence, DALYs, and deaths rate per 100,000 population of tuberculosis stratified by type **(A–C)**, sex **(D–F)**, and SDI **(G–I)** in global (1990–2019) and the distribution of tuberculosis globally in 2019 **(J–L)** were generated by GDB 2019.

From 1990 to 2019, according to the GBD 2019 age-standardized incidence, DALYs, and deaths rate estimates, all HIV-negative TB including the drug-susceptible tuberculosis (DS-TB), multidrug-resistant tuberculosis without extensive drug resistance (MDR-TB without XDR), and extensively drug-resistant tuberculosis (XDR-TB) were analyzed. The results of age-standardized incidence, DALYs, and deaths rate all show that DS-TB ranks first among the three types of HIV-negative TB, followed by MDR-TB without XDR and XDR-TB (Figures 1A–C; Supplementary Table S1). For HIV/AIDS-positive TB, HIV/AIDS-drug-susceptible tuberculosis (HIV/AIDS-DS-TB) ranks first, followed by MDR-TB without XDR and XDR-TB (Supplementary Figures S1A–C; Supplementary Table S2). In 2019, age-standardized incidence, DALYs, and deaths rate, all HIV-negative TB (95% CI, Supplementary Tables S1–S3) are 10.67 million (9.40–12.19), 59.04 million (53.68–64.64), 1.46 million (1.34–1.60). Regardless of age-standardized incidence and DALYs or age-standardized deaths rate of all HIV-negative TB, men are always higher than women (Figures 1D–F; Supplementary Table S3), and men's age-standardized incidence, DALYs, and deaths rate (95% CI) were 11.96 million (10.52–13.64), 75.05 million (67.57–82.50), 1.97 million (1.79–2.16), respectively; female age-standardized incidence, DALYs, and deaths rates (95% CI) are as follows: 9.49 million (8.30–10.91), 43.70 million (38.59–50.70), 1.01 million (0.88–1.21).

**Figure 2:**
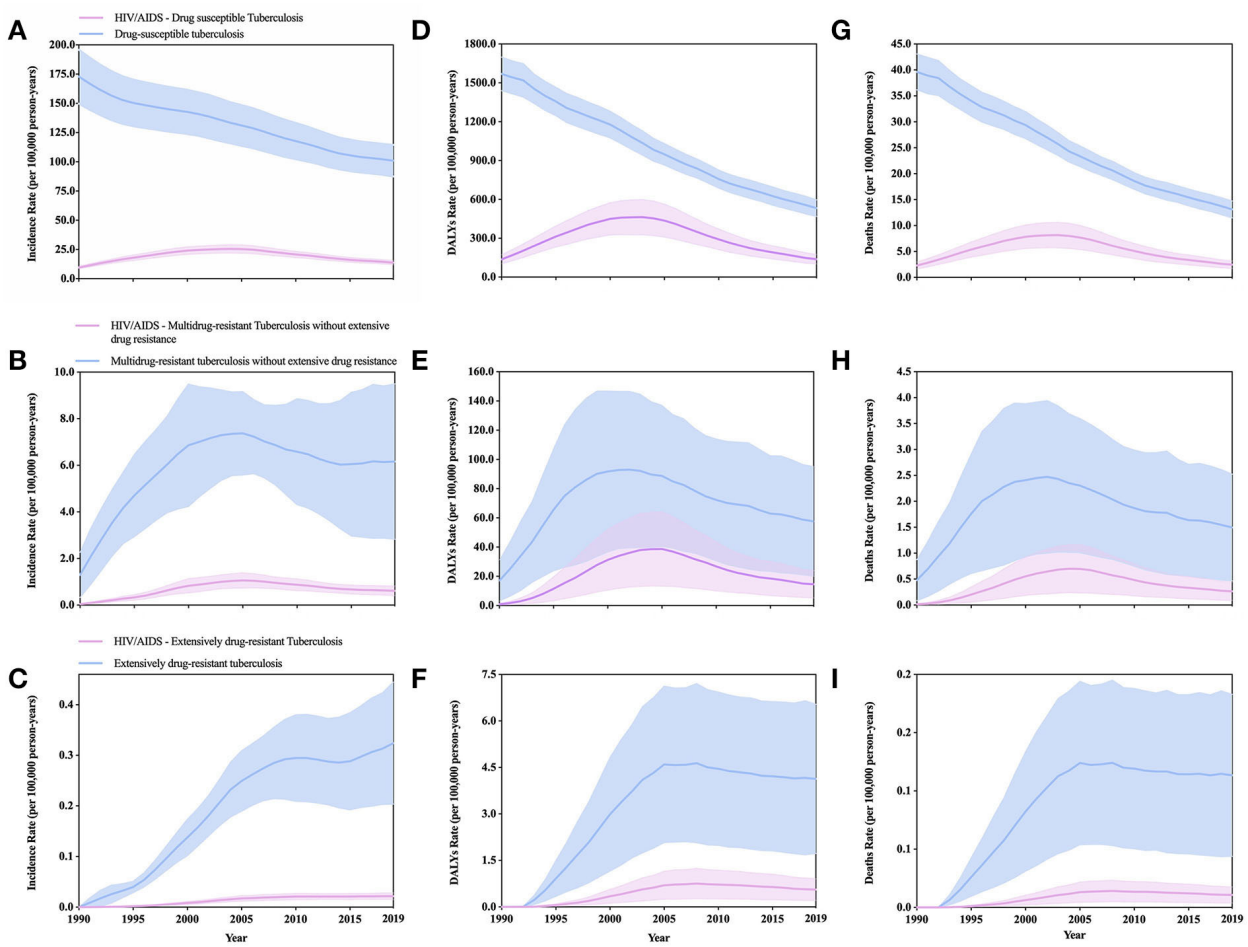
Comparison of major HIV/AIDS-negative and positive tuberculosis burdens estimates (solid lines) and 95% uncertainty intervals (shaded areas) for the age-standardized incidence **(A–C)**, DALYs **(D–F)**, and deaths **(G–I)** rate per 100,000 population of tuberculosis stratified by HIV/AIDS status in global (1990–2019).

From 1990–2019, the change of age-standardized incidence, DALYs, and deaths rate of all-HIV negative TB (Supplementary Table S3) have all decreased, respectively, by 38.16% (from −40.73 to −35.26), 62.75% (from −66.61 to −57.98) and 63.49% (−67.62 to −57.85). In addition, the decline of men is lower than that of women (Supplementary Table S3). From 1990 to 2019, we found that regardless of age-standardized incidence, DALYs or deaths rate, the regions with the highest burden of all HIV TB were in low SDI regions, followed by low-middle SDI, middle SDI, high-middle SDI, and high SDI regions (Figures 1G–I; Tables 1–3). In the low SDI regions, age-standardized incidence, DALYs, and deaths rates are 21.15 million (18.74–24.09), 198.31 million (175.76–227.72), 5.99 million (5.33–6.85), respectively; in high SDI regions, age-standardized incidence, DALYs, and deaths rates are 1.01 million (0.87–1.16), 1.97 million (1.77–2.21), 0.07 million (0.06–0.08), respectively. In the low SDI area, the mortality rate accounts for 28.32% of the morbidity, while in the high SDI area, the mortality rate accounts for 6.9% of the morbidity. The mortality/morbidity rate in the low SDI area is more than four times that of the high SDI area. The SDI areas are more likely to die from TB than high SDI areas. In addition, the age-standardized incidence of TB shows that low SDI areas are more than 20 times higher than high SDI areas, age-standardized DALYs, and low SDI areas are more than 100 times higher than high SDI areas. In terms of age-standardized deaths rate, low SDI area is about 85 times that of the high SDI area. Unfortunately, the GBD 2019 data lacks an overall estimate of HIV/AIDS-related TB. Nevertheless, we will discuss in detail the different drug resistance types of HIV/AIDS-related TB. In addition, our age-standardized incidence rates for DS-TB and HIV/AIDS-DS-TB, MDR-TB without XDR and HIV/AIDS-multidrug-resistant tuberculosis without extensive drug resistance (HIV/AIDS-MDR-TB without XDR), XDR-TB and HIV/AIDS-extensively drug-resistant tuberculosis (HIV/AIDS-XDR-TB), respectively, DALYs rate and deaths rate, compared from 1990–2019 change trends, the results show that the HIV-negative group is always higher than the latter (Figures 2A–I, Supplementary Tables S1, S2).

**Table 1:** Incidence of major HIV-negative individuals in 2019, and change of age-standardized incidence during the periods 1990–2010 and 2010–2019 for both sexes.

**Group**	**Drug-susceptible tuberculosis**			**Extensively drug-resistant tuberculosis**			**Multidrug-resistant tuberculosis without extensive drug resistance**			**Tuberculosis**		
	**Number of incident cases, 2019**	**Annualized rate of change of age-standardized incidence**		**Number of incident cases, 2019**	**Annualized rate of change of age-standardized incidence**		**Number of incident cases, 2019**	**Annualized rate of change of age-standardized incidence**		**Number of incident cases, 2019**	**Annualized rate of change of age-standardized incidence**	
		**1990–2010**	**2010–2019**		**1990–2010**	**2010–2019**		**1990–2010**	**2010–2019**		**1990–2010**	**2010–2019**
Global	10,077,419 (8,738,073 to 11,455,752)	−31.8 (−34.3 to −29.3)	−13.8 (−17.3 to −11.0)	31,016 (21,115 to 45,110)	–	7.5 (−23.0 to 51.3)	563,014 (312,112 to 973,098)	504.0 (156.8 to 1421.3)	−11.1 (−46.6 to 47.6)	10,671,449 (9,395,605 to 12,194,100)	−28.4 (−30.7 to −26.0)	−13.6 (−15.4 to −11.7)
High SDI	982,370 (851,387 to 1,134,431)	−42.2 (−45.3 to −39.0)	−20.6 (−22.8 to −18.3)	2,432 (1,609 to 3,875)	–	12.2 (−26.5 to 70.3)	22,415 (13,717 to 38,727)	14.2 (−36.6 to 108.7)	−23.5 (−55.2 to 26.3)	1,007,217 (870,478 to 1,161,202)	−41.4 (−44.4 to −38.3)	−20.6 (−22.5 to −18.6)
High–middle SDI	4,024,958 (3,443,847 to 4,629,602)	−31.8 (−36.0 to −28.1)	−26.1 (−30.8 to −21.9)	72,070 (48,408 to 102,383)	–	−3.7 (−32.8 to 44.0)	482,617 (288,844 to 773,317)	512.3 (111.4 to 1803.0)	−33.4 (−57.2 to 10.4)	4,579,645 (3,989,516 to 5,271,730)	−23.0 (−26.0 to −19.5)	−26.6 (−28.8 to −24.6)
Middle SDI	9,015,100 (7936886 to 10,194,207)	−45.6 (−47.6 to −43.6)	−13.5 (−16.9 to −10.5)	23,778 (14,148 to 39,473)	–	16.1 (−29.7 to 85.9)	432,597 (215,512 to 797,771)	179.9 (3.9 to 857.5)	−16.5 (−55.2 to 48.9)	9471475 (8,369,573 to 10,711,366)	−43.3 (−44.9 to −41.6)	−13.6 (−15.5 to −11.6)
Low–middle SDI	17,500,166 (14,921,311 to 20,233,346)	−37.4 (−40.6 to −34.2)	−18.1 (−23.3 to −14.3)	3,135,2 (14,502 to 61,831)	–	39.6 (−32.3 to 161.7)	1,024,299 (390,379 to 2,262,371)	1421.6 (351.2 to 5,385.7)	−2.2 (−59.5 to 102.4)	18,555,817 (1,608,7711 to 21,293,251)	−34.4 (−37.1 to −31.5)	−17.3 (−19.5 to −14.9)
Low SDI	20,228,727 (17,704,872 to 22,958,495)	−31.8 (−34.6 to −29.2)	−18.5 (−21.6 to −15.6)	15,135 (6,721 to 32,291)	–	48.0 (−35.5 to 242.3)	903,688 (498,968 to 1,630,859)	1,388.7 (572.5 to 3,070.8)	0.1 (−44.7 to 86.5)	21,147,549 (18,738,135 to 24,087,649)	−29.4 (−31.9 to −27.0)	−17.8 (−19.9 to −15.7)
High–income	732,692 (635,151 to 849795)	−48.3 (−51.1 to −45.5)	−20.9 (−23.2 to −18.4)	1,573 (993 to 2,597)	–	22.5 (−18.9 to 90.6)	12,511 (7,896 to 20,652)	−2.5 (−40.9 to 62.2)	−21.6 (−48.1 to 21.9)	746,776 (646,686 to 865,802)	−47.8 (−50.7 to −45.0)	−20.8 (−23.0 to −18.5)
High–income North America	230,533 (197,720 to 270,984)	−57.1 (−60.4 to −53.9)	−25.4 (−27.4 to −23.3)	400 (186 to 829)	–	21.8 (−43.7 to 141.8)	3,180 (1,479 to 6,589)	−81.4 (−89.4 to −65.8)	−22.0 (−64.0 to 54.7)	234,113 (201,052 to 275,784)	−57.8 (−61.1 to −54.7)	−25.3 (−27.2 to −23.3)
Australasia	590,651 (507,362 to 696,802)	−38.5 (−42.9 to −33.6)	−3.9 (−9.3 to 2.0)	2,106 (933 to 4,242)	–	131.5 (−18.6 to 530.7)	16,753 (7,424 to 33,746)	115.2 (−32.8 to 678.0)	48.1 (−47.9 to 303.6)	609,510 (522,345 to 715,371)	−37.7 (−41.9 to −32.6)	−2.7 (−7.8 to 2.5)
High–income Asia Pacific	1,607,829 (1,377,005 to 1,874,647)	−54.3 (−58.4 to −49.5)	−22.6 (−26.4 to −18.5)	2,529 (659 to 8,084)	–	8.1 (−67.6 to 189.1)	20,115 (5,246 to 64,285)	14.6 (−68.5 to 367.9)	−30.8 (−79.3 to 85.0)	1,630,472 (1,401,086 to 1,901,303)	−53.9 (−58.0 to −49.0)	−22.7 (−26.2 to −18.8)
Western Europe	685,048 (576,372 to 812,893)	−39.0 (−41.8 to −35.9)	−21.9 (−24.6 to −19.0)	2,005 (1,344 to 2,953)	–	27.9 (−10.9 to 82.6)	15,945 (10,689 to 23,494)	77.4 (0.6 to 208.8)	−18.2 (−43.0 to 16.8)	702,998 (591,087 to 833,887)	−38.0 (−40.9 to −34.8)	−21.7 (−24.4 to −18.9)
Southern Latin America	1,173,708 (1009774 to 1,368,438)	−49.9 (−53.9 to −45.4)	−1.3 (−7.9 to 5.0)	2,021 (549 to 6,350)	–	30.9 (−56.1 to 240.9)	16,075 (4,365 to 50,501)	155.7 (−31.2 to 1,162.8)	−16.2 (−71.9 to 118.2)	1,191,803 (1,031,101 to 1,389,821)	−49.2 (−53.1 to −44.8)	−1.5 (−8.0 to 4.9)
Central Europe, Eastern Europe, and Central Asia	3,649,114 (2,975,777 to 4,450,656)	−20.0 (−28.3 to −11.2)	−33.3 (−40.3 to −25.6)	265,316 (181,799 to 371,193)	–	4.8 (−26.5 to 53.1)	1,210,897 (829,736 to 1,694,144)	2,801.0 (1,081.2 to 6,781.8)	−34.9 (−54.3 to −4.9)	5,125,328 (4,306,898 to 6,134,301)	9.8 (2.7 to 18.6)	−32.4 (−36.1 to −28.6)
Eastern Europe	4,645,812 (3,592,545 to 5,909,367)	−14.5 (−26.5 to −2.0)	−37.1 (−46.4 to −26.6)	413,474 (258,785 to 607,709)	–	7.6 (−31.5 to 72.0)	1,887,081 (1,181,052 to 2,773,550)	2,604.2 (897.2 to 7,216.9)	−33.1 (−57.5 to 6.8)	6,946,367 (5,743,355 to 8,474,562)	21.2 (12.0 to 32.2)	−34.4 (−38.7 to −29.8)
Central Europe	1,367,729 (1,175,106 to 1,605,953)	−32.4 (−37.0 to −27.1)	−25.1 (−28.9 to −21.2)	6,455 (3,499 to 11,420)	–	4.6 (−59.6 to 149.3)	29,460 (15,972 to 52,105)	210.3 (31.7 to 610.0)	−35.0 (−75.0 to 54.9)	1,403,644 (1,203,102 to 1,642,587)	−30.8 (−35.5 to −25.5)	−25.2 (−28.6 to −21.4)
Central Asia	4,163,006 (3,466,650 to 4,972,969)	−32.8 (−37.3 to −27.9)	−24.8 (−33.6 to −16.0)	250,154 (165,407 to 351,612)	–	−3.9 (−35.0 to 37.8)	1,141,704 (755,001 to 1,604,852)	122,63.4 (4268.0 to 412,20.1)	−40.3 (−59.6 to −14.4)	5,554,864 (4,800,209 to 6,410,598)	−6.6 (−12.4 to −0.2)	−27.9 (−31.7 to −23.8)
Latin America and Caribbean	2,687,923 (2,322,433 to 3,118,899)	−46.7 (−49.4 to −43.8)	−11.3 (−14.7 to −8.1)	8,076 (4,696 to 13,653)	–	44.8 (−10.7 to 128.8)	102,262 (59,464 to 172,901)	419.2 (125.6 to 1,116.2)	−6.0 (−42.0 to 48.6)	2,798,261 (2,407,528 to 3,248,892)	−44.9 (−47.6 to −42.0)	−11.0 (−13.9 to −8.1)
Central Latin America	1,741,175 (1,509,253 to 1,998,885)	−41.5 (−44.9 to −37.9)	−5.8 (−9.8 to −1.9)	4,234 (1789 to 8,384)	–	60.4 (−24.3 to 219.9)	53,607 (22,652 to 106,151)	1,159.6 (374.6 to 3,577.9)	4.2 (−50.9 to 107.8)	1,799,015 (1,566,902 to 2,059,309)	−39.9 (−43.1 to −36.2)	−5.4 (−8.6 to −2.0)
Andean Latin America	6,124,687 (5,259,831 to 7,168,303)	−62.0 (−65.2 to −58.6)	−25.1 (−29.9 to −20.2)	33,607 (18,646 to 56,365)	–	8.4 (−36.5 to 83.4)	425,536 (236,132 to 713,547)	251.6 (30.7 to 1,168.5)	−29.6 (−58.7 to 19.2)	6,583,829 (5,668,696 to 7,628,324)	−59.4 (−62.6 to −55.9)	−25.3 (−28.8 to −21.3)
Caribbean	3,237,851 (2,819,755 to 3,712,534)	−28.7 (−32.6 to −24.8)	0.7 (−4.5 to 6.2)	1,424 (508 to 3,752)	–	121.6 (−14.8 to 547.9)	18,025 (6,435 to 47,514)	−37.0 (−79.6 to 98.2)	44.0 (−44.7 to 320.9)	3,257,300 (2,830,119 to 3,740,111)	−28.7 (−32.6 to −24.7)	0.9 (−4.4 to 6.5)
Tropical Latin America	2,685,272 (2,268,547 to 3,154,004)	−38.4 (−42.1 to −34.2)	−9.8 (−16.3 to −4.2)	6,753 (1,427 to 18,456)	–	96.5 (−51.6 to 390.4)	85,506 (18,069 to 233,712)	2,677.4 (528.3 to 20,956.4)	27.6 (−68.5 to 218.6)	2,777,531 (2,349,676 to 3,274,901)	−37.0 (−40.7 to −32.8)	−8.8 (−14.1 to −3.7)
Southeast Asia, East Asia, and Oceania	7,740,765 (6,824,498 to 8,685,989)	−48.5 (−50.2 to −46.7)	−16.3 (−19.7 to −13.3)	21,884 (10,815 to 45,334)	–	2.3 (−49.3 to 103.4)	240,008 (118,604 to 497,212)	36.0 (−50.8 to 383.8)	−33.8 (−67.2 to 31.6)	8,002,657 (7,112,167 to 8,932,688)	−47.2 (−48.6 to −45.7)	−16.9 (−19.0 to −14.7)
East Asia	4,216,185 (3,632,791 to 4,791,289)	−43.4 (−46.5 to −39.9)	−29.9 (−36.1 to −25.9)	16,480 (3,769 to 50,367)	–	−18.1 (−78.1 to 120.8)	180,740 (41,330 to 552,404)	3.4 (−65.7 to 360.6)	−47.0 (−85.8 to 42.9)	4,413,405 (3,925,381 to 4,929,774)	−41.9 (−44.0 to −39.5)	−30.8 (−32.6 to −28.5)
Southeast Asia	16,218,276 (14,403,770 to 18,060,079)	−54.0 (−55.5 to −52.4)	−10.1 (−12.7 to −7.4)	34,506 (20,369 to 56,154)	–	35.8 (−25.4 to 149.6)	378,431 (223,424 to 615,799)	438.9 (116.3 to 1,491.7)	−12.1 (−51.8 to 61.5)	16,631,213 (14,840,698 to 18,503,116)	−52.9 (−54.5 to −51.3)	−10.1 (−12.3 to −7.6)
Oceania	10,005,424 (8,931,039 to 11,114,474)	−25.1 (−28.0 to −22.0)	−11.0 (−16.0 to −6.5)	32,189 (12,006 to 72,679)	–	310.9 (3.9 to 1,388.1)	353,027 (131,635 to 797,369)	1,953.4 (418.5 to 9,235.6)	165.9 (−32.8 to 862.9)	10,390,640 (9,346,294 to 1,1462,527)	−24.2 (−27.2 to −21.1)	−8.7 (−12.5 to −5.1)
North Africa and Middle East	2,550,310 (2,204,205 to 2958695)	−42.0 (−45.0 to −38.4)	−21.3 (−24.3 to −18.4)	2,680 (1,633 to 4,496)	–	17.1 (−29.6 to 93.0)	75,937 (46,262 to 127,391)	884.2 (402.7 to 1,845.2)	−22.9 (−53.6 to 27.2)	2,628,927 (2,270,964 to 3,049,615)	−40.3 (−43.4 to −36.7)	−21.3 (−23.9 to −18.8)
South Asia	20,480,991 (17,185,849 to 24013953)	−37.4 (−41.6 to −33.2)	−19.9 (−26.1 to −15.1)	36,443 (10,237 to 83,179)	–	47.7 (−48.3 to 230.1)	1,486,893 (417,644 to 3,393,773)	4,155.7 (929.3 to 21,758.0)	−2.3 (−65.8 to 118.2)	22,004,327 (1,899,5837 to 25,346,862)	−33.7 (−37.3 to −29.9)	−18.8 (−21.2 to −16.2)
Sub–Saharan Africa	23,240,221 (20,576,148 to 26,367,753)	−23.4 (−26.1 to −20.7)	−16.5 (−19.2 to −13.9)	4,902 (3,291 to 7,452)	–	44.1 (2.6 to 113.5)	759,768 (510,186 to 1,154,992)	1,047.3 (557.4 to 1,920.5)	−4.1 (−31.7 to 42.0)	24,004,890 (2,125,7144 to 27,278,756)	−21.4 (−24.1 to −18.6)	−16.2 (−18.6 to −13.7)
Southern Sub–Saharan Africa	33,196,173 (28600662 to 38,515,379)	−19.3 (−24.7 to −12.3)	−1.3 (−8.5 to 5.1)	7,443 (3,754 to 14,316)	–	42.2 (−38.3 to 229.7)	1,153,614 (581,862 to 2,219,107)	662.7 (148.0 to 3,180.6)	−5.4 (−59.0 to 119.2)	34,357,230 (2,961,9366 to 39,715,574)	−16.6 (−22.0 to −9.5)	−1.5 (−7.9 to 3.9)
Western Sub–Saharan Africa	17,327,843 (15,123,163 to 19,818,589)	−24.0 (−28.3 to −19.9)	−20.7 (−24.5 to −17.2)	3,840 (1,786 to 7,886)	–	16.5 (−41.2 to 137.7)	595,242 (276,819 to 1,222,360)	1,001.2 (424.1 to 2,245.5)	−22.5 (−60.9 to 58.1)	17,926,925 (15,745,074 to 20,465,886)	−21.5 (−25.9 to −17.4)	−20.8 (−23.6 to −17.7)
Eastern Sub–Saharan Africa	27,122,987 (23,891,709 to 30,941,243)	−26.8 (−29.8 to −23.3)	−16.0 (−19.1 to −13.0)	5,918 (3,717 to 10,058)	–	75.0 (12.1 to 185.2)	917,287 (576,147 to 1,558,791)	2,275.7 (861.1 to 5,530.1)	16.4 (−25.4 to 89.7)	28,046,191 (24,753,966 to 32,002,059)	−25.1 (−28.2 to −21.5)	−15.2 (−18.1 to −12.5)
Central Sub–Saharan Africa	29,618,907 (26,124,997 to 33,250,345)	−1.8 (−6.9 to 2.3)	−13.9 (−18.8 to −8.7)	4,762 (1,263 to 13,700)	–	65.5 (−44.0 to 376.1)	738,193 (195,674 to 2,122,783)	603.6 (31.8 to 3,379.0)	10.1 (−62.7 to 216.9)	30,361,862 (27,020,825 to 33,866,880)	−0.2 (−4.3 to 3.8)	−13.4 (−17.8 to −8.9)

**Table 2:** The DALYs of major HIV–negative individuals in 2019, and change of age–standardized DALYs during the periods 1990–2010 and 2010–2019 for both sexes.

**Group**	**Drug–susceptible tuberculosis**				**Extensively drug–resistant tuberculosis**			**Multidrug–resistant tuberculosis without extensive drug resistance**			**Tuberculosis**		
	**Number of DALYs, 2019**	**Annualized rate of change of age–standardized DALYs**		**Number of DALYs, 2019**	**Annualized rate of change of age–standardized DALYs**		**Number of DALYs, 2019**	**Annualized rate of change of age–standardized DALYs**		**Number of DALYs, 2019**	**Annualized rate of change of age–standardized DALYs**	
		**1990–2010**	**2010–2019**		**1990–2010**	**2010–2019**		**1990–2010**	**2010–2019**		**1990–2010**	**2010–2019**
Global	53,422,865 (46,676,267 to 59,669,580)	−51.6 (−55.9 to −47.3)	−29.8 (−35.9 to −23.3)	381,509 (189,136 to 668,316)	—	−8.2 (−33.7 to 31.8)	5,238,076 (2,263,595 to 9,759,929)	386.5 (137.5 to 1006.8)	−22.7 (−51.5 to 21.3)	59,042,451 (53,684,783 to 64,641,528)	−47.5 (−50.6 to −43.4)	−29.1 (−34.5 to −23.6)
High SDI	1,864,033 (1,657,623 to 2,112,640)	−69.1 (−71.6 to −66.5)	−28.2 (−33.7 to −21.4)	17,269 (8,183 to 33,055)	—	−7.5 (−35.2 to 30.2)	83,813 (37,325 to 173,545)	−28.2 (−56.0 to 19.4)	−34.8 (−59.6 to 9.1)	1,965,116 (1,769,448 to 2,214,210)	−68.0 (−70.6 to −65.5)	−28.4 (−33.6 to −22.1)
High–middle SDI	9,656,432 (8,011,615 to 11,122,816)	−61.2 (−66.5 to −56.1)	−39.1 (−46.5 to −31.3)	508,129 (285,413 to 777,826)	—	−27.5 (−45.6 to 0.8)	1,823,912 (961,776 to 3,188,864)	227.0 (28.0 to 905.4)	−46.7 (−64.4 to −12.9)	11,988,473 (1,087,9230 to 13,089,954)	−52.3 (−55.4 to −48.9)	−40.0 (−44.4 to −35.4)
Middle SDI	34,089,012 (30,391,151 to 37,561,252)	−60.2 (−63.7 to −56.9)	−35.3 (−40.7 to −29.5)	297,278 (142,144 to 539,010)	—	−14.5 (−45.3 to 25.5)	2,672,977 (1,058,878 to 5,048,207)	93.4 (−17.2 to 495.5)	−39.2 (−65.9 to 4.9)	37,059,267 (3,404,6223 to 40,091,006)	−57.3 (−59.7 to −54.3)	−35.5 (−39.7 to −30.6)
Low–middle SDI	105,034,556 (87,174,675 to 121,173,835)	−57.0 (−62.2 to −51.8)	−31.6 (−40.6 to −22.5)	668,617 (221,464 to 1,426,008)	—	15.5 (−42.9 to 110.7)	11,779,956 (3,784,100 to 26,265,593)	811.7 (211.1 to 2,792.2)	−17.9 (−63.4 to 60.1)	117,483,128 (104,647,935 to 130,986,372)	−53.0 (−57.0 to −48.4)	−30.3 (−36.9 to −23.0)
Low SDI	181,931,292 (157,033,169 to 2,099,424,78)	−48.4 (−53.1 to −42.7)	−31.3 (−37.9 to −24.1)	447,550 (168,786 to 916,468)	—	18.9 (−37.2 to 127.3)	15,935,638 (6,985,776 to 28,018,760)	922.7 (406.2 to 1910.8)	−17.4 (−46.0 to 33.7)	198,314,481 (175,760,628 to 227,715,901)	−44.8 (−48.9 to −39.2)	−30.3 (−36.6 to −23.7)
High–income	1,365,328 (1,251,503 to 1,473,973)	−74.6 (−75.8 to −73.4)	−25.3 (−28.0 to −22.4)	9,890 (4,205 to 20,051)	—	5.9 (−31.1 to 71.8)	39,429 (17,676 to 81,357)	−52.2 (−72.8 to −17.1)	−31.8 (−55.4 to 9.9)	1,414,647 (1,313,602 to 1,517,534)	−74.1 (−75.2 to −73.0)	−25.3 (−27.4 to −22.8)
High–income North America	533,216 (485,434 to 582,727)	−70.5 (−72.2 to −68.5)	−8.3 (−12.6 to −4.4)	3,442 (1,125 to 8,315)	—	45.4 (−33.3 to 189.6)	13,737 (4,796 to 32,873)	−88.4 (−93.0 to −78.8)	−7.3 (−57.4 to 84.4)	550,394 (505,799 to 598,674)	−71.5 (−72.8 to −70.0)	−8.1 (−11.4 to −4.8)
Australasia	525,608 (429,935 to 634,807)	−59.7 (−63.8 to −55.6)	−13.7 (−24.7 to −2.2)	6,997 (2,364 to 16,116)	—	105.1 (−26.5 to 440.4)	28,770 (10,269 to 63,003)	22.9 (−63.4 to 366.0)	31.9 (−53.0 to 249.6)	561,374 (470,595 to 673,032)	−58.5 (−62.2 to −54.3)	−11.5 (−20.3 to −0.4)
High–income Asia Pacific	2,674,148 (2,402,789 to 2,920,938)	−79.7 (−81.2 to −78.4)	−33.7 (−38.0 to −29.5)	15,325 (2,829 to 51,088)	—	−9.3 (−73.8 to 131.2)	60,475 (11,867 to 191,002)	−57.4 (−88.5 to 83.8)	−41.7 (−83.1 to 48.5)	2,749,948 (2,508,546 to 2,983,379)	−79.4 (−80.6 to −78.2)	−33.8 (−37.2 to −30.2)
Western Europe	858,780 (778,328 to 945,557)	−71.6 (−73.0 to −70.1)	−26.6 (−29.8 to −23.3)	8,772 (4,073 to 17,054)	—	10.6 (−22.0 to 55.1)	35,593 (17,467 to 66,028)	−25.5 (−56.0 to 30.8)	−28.5 (−49.4 to 0.4)	903,144 (828,704 to 986,257)	−70.7 (−72.0 to −69.3)	−26.4 (−29.4 to −23.3)
Southern Latin America	5,086,566 (4,565,267 to 5,581,735)	−69.7 (−71.6 to −67.7)	−21.4 (−28.4 to −14.5)	33,196 (7,214 to 108,697)	—	−3.7 (−66.1 to 151.1)	129,996 (29,615 to 412,271)	38.4 (−62.0 to 566.5)	−37.9 (−78.2 to 61.6)	5,249,758 (4,838,940 to 5,692,082)	−68.8 (−70.3 to −67.1)	−21.8 (−27.3 to −15.8)
Central Europe, Eastern Europe, and Central Asia	11,592,264 (8,482,904 to 14,534,708)	−22.1 (−42.4 to −4.0)	−44.7 (−53.9 to −35.2)	227,6718 (1,318,449 to 3,426,821)	—	−17.0 (−35.9 to 10.8)	5,172,251 (3,195,930 to 7,354,642)	2,174.6 (964.8 to 4, 890.3)	−47.8 (−59.8 to −30.5)	19,041,233 (17,388,589 to 20,780,727)	23.0 (19.4 to 27.6)	−43.4 (−47.8 to −38.8)
Eastern Europe	11,754,739 (7,752,734 to 15862,953)	5.1 (−27.8 to 35.8)	−50.8 (−62.4 to −36.1)	2,871,273 (1,627,275 to 4,347,025)	—	−16.1 (−39.9 to 23.4)	6,539,035 (3,880,816 to 9,664,468)	1925.1 (715.6 to 5394.4)	−47.1 (−62.3 to −22.8)	21,165,047 (18,898,229 to 23,668,148)	70.0 (64.7 to 81.6)	−46.7 (−52.6 to −40.6)
Central Europe	4,249,250 (3,663,795 to 4,820,303)	−55.9 (−59.2 to −53.4)	−37.8 (−46.0 to −29.3)	78,787 (29,413 to 166,328)	—	−15.8 (−70.2 to 123.9)	178,828 (68,097 to 382,136)	73.8 (−29.5 to 329.9)	−47.2 (−81.2 to 39.9)	4,506,865 (3,949,646 to 5,117,948)	−53.7 (−55.4 to −51.8)	−38.0 (−45.3 to −30.2)
Central Asia	19,543,646 (13,662,140 to 25,002,920)	−43.3 (−57.8 to −29.7)	−38.8 (−51.4 to −23.3)	3,562,648 (1,929,378 to 5,746,708)	—	−23.5 (−47.3 to 5.7)	8,051,066 (4,607,795 to 12,155,328)	7,854.0 (3,041.9 to 26,277.3)	−52.1 (−66.9 to −34.1)	31,157,360 (27,790,597 to 35,046,387)	−5.6 (−9.2 to −1.4)	−41.7 (−47.5 to −35.0)
Latin America and Caribbean	10,979,950 (9,439,112 to 12,617,838)	−74.0 (−75.9 to −72.1)	−28.4 (−35.6 to −19.7)	116,022 (46,765 to 236,294)	—	10.1 (−29.4 to 66.5)	724,032 (305,884 to 1,462,396)	119.9 (−1.0 to 424.2)	−27.7 (−53.7 to 8.7)	11,820,004 (10425048 to 13,425,278)	−72.3 (−73.8 to −70.6)	−28.1 (−35.3 to −19.7)
Central Latin America	7,966,157 (6,664,474 to 9,424,585)	−77.1 (−78.6 to −75.7)	−21.8 (−33.0 to −8.4)	74,611 (25,510 to 172,152)	—	22.6 (−39.6 to 132.7)	467,048 (165,144 to 1,085,389)	402.9 (107.1 to 1,264.3)	−19.4 (−60.4 to 51.7)	8,507,816 (7,272,120 to 10,018,707)	−75.7 (−76.8 to −74.5)	−21.4 (−32.0 to −8.7)
Andean Latin America	23,164,834 (17,515,269 to 29468116)	−82.1 (−85.0 to −79.0)	−41.5 (−53.3 to −27.9)	482,303 (167,515 to 1,026,341)	—	−13.6 (−51.5 to 50.7)	3,001,873 (1,158,458 to 6,275,897)	44.9 (−46.8 to 410.1)	−43.3 (−68.3 to −1.8)	26,649,010 (21,309,186 to 33,069,045)	−79.7 (−82.3 to −76.8)	−41.4 (−53.0 to −28.9)
Caribbean	22,807,329 (18,287,742 to 29,009,686)	−54.1 (−61.7 to −44.9)	−11.3 (−24.5 to 5.4)	37,643 (7,725 to 120,555)	—	105.9 (−33.6 to 622.4)	232,306 (51,572 to 741,040)	−59.5 (−89.9 to 74.9)	33.9 (−56.6 to 357.8)	23,077,279 (18,630,886 to 29,226,489)	−54.1 (−61.7 to −45.0)	−10.9 (−23.9 to 5.5)
Tropical Latin America	8,607,012 (7,496,690 to 9,408,760)	−65.6 (−68.1 to −63.4)	−31.9 (−38.9 to −26.4)	80,462 (13,334 to 232,007)	—	35.3 (−62.7 to 226.6)	502,935 (95,276 to 1,371,378)	1,329.1 (223.4 to 10,666.9)	−11.3 (−75.7 to 114.2)	9,190,409 (8,653,034 to 9,806,860)	−63.9 (−65.8 to −62.1)	−30.7 (−34.1 to −26.7)
Southeast Asia, East Asia, and Oceania	27,721,911 (24,998,776 to 30,485,228)	−65.0 (−68.1 to −61.4)	−35.9 (−41.5 to −29.3)	222,866 (93,163 to 470,337)	—	−24.0 (−55.3 to 25.4)	1,224,305 (555,745 to 2,413,379)	−24.9 (−70.7 to 142.9)	−50.0 (−70.9 to −17.3)	29,169,082 (26,653,071 to 31,884,461)	−63.8 (−66.5 to −60.6)	−36.6 (−41.7 to −30.7)
East Asia	7,613,119 (6,188,762 to 9,041,545)	−80.6 (−83.6 to −77.3)	−41.4 (−49.8 to −32.0)	95,393 (20,783 to 270,768)	—	−37.9 (−81.0 to 52.4)	551,961 (129,122 to 1,501,930)	−66.5 (−88.2 to 43.1)	−58.2 (−87.1 to 2.1)	8,260,473 (7,130,296 to 9,642,073)	−79.6 (−82.0 to −77.0)	−42.9 (−49.2 to −35.7)
Southeast Asia	79,921,750 (71,686,540 to 8,8512,449)	−57.2 (−61.7 to −52.0)	−36.1 (−42.7 to −28.4)	550,895 (220,139 to 1,187,069)	—	−18.7 (−54.6 to 43.3)	2,950,391 (1,277,201 to 6,064,725)	305.3 (54.6 to 1162.0)	−46.9 (−70.5 to −5.9)	83,423,036 (75,109,968 to 92,043,542)	−55.3 (−59.6 to −50.3)	−36.4 (−42.7 to −29.3)
Oceania	72,522,825 (51,687,644 to 96,117,455)	−34.4 (−43.5 to −23.8)	−25.8 (−37.2 to −13.2)	930,510 (222,124 to 2,399,806)	—	227.3 (−15.3 to 1157.5)	4,957,693 (1,224,164 to 1,252,1788)	1,502.6 (300.7 to 7,255.0)	112.1 (−45.3 to 711.1)	78,411,028 (55,989,827 to 10,439,6559)	−32.7 (−41.7 to −21.7)	−21.9 (−32.6 to −9.9)
North Africa and Middle East	10,938,453 (8,928,694 to 13,286,644)	−64.5 (−69.2 to −57.0)	−36.4 (−43.0 to −27.6)	50,896 (18,133 to 111,731)	—	−14.1 (−53.9 to 64.2)	708,928 (279,633 to 1,553,081)	633.3 (251.2 to 1,406.6)	−43.0 (−69.0 to 8.3)	11,698,277 (9,679,327 to 14092394)	−61.9 (−66.4 to −54.4)	−36.8 (−42.3 to −27.9)
South Asia	106,259,629 (84,804,062 to 124,786,790)	−60.3 (−65.8 to −54.7)	−32.8 (−43.7 to −21.0)	726,544 (179,676 to 1,714,811)	—	21.5 (−53.4 to 152.2)	1,442,9579 (3,622,339 to 33,497,063)	2,088.2 (445.5 to 11,503.1)	−19.3 (−69.1 to 69.1)	121,415,751 (107,635,515 to 137,204,601)	−55.8 (−59.8 to −50.8)	−31.3 (−39.2 to −22.1)
Sub–Saharan Africa	209,666,954 (179,686,586 to 243,717,359)	−41.3 (−46.4 to −35.2)	−32.1 (−39.1 to −24.8)	205,302 (86,281 to 397,275)	—	19.1 (−13.4 to 66.9)	15,297,529 (7,062,894 to 27,182,866)	793.0 (395.8 to 1,491.4)	−20.5 (−42.3 to 11.1)	225,169,785 (196,603,360 to 259,821,718)	−37.9 (−42.6 to −32.1)	−31.4 (−38.2 to −24.3)
Southern Sub–Saharan Africa	193,731,974 (16,7462,698 to 219,891,304)	5.6 (−11.0 to 23.5)	−41.4 (−47.3 to −34.5)	210,923 (72,097 to 493,198)	—	−9.9 (−56.2 to 97.3)	15,768,923 (5,637,570 to 32,943,763)	893.6 (209.9 to 4,339.5)	−39.4 (−70.8 to 31.3)	209,711,820 (186,844,567 to 233,887,784)	13.0 (−2.6 to 31.1)	−41.3 (−46.3 to −35.5)
Western Sub–Saharan Africa	146,451,589 (119,644,604 to 175,356,045)	−44.7 (−53.8 to −34.1)	−26.1 (−37.4 to −14.0)	136,381 (47,579 to 309,964)	—	6.9 (−40.5 to 89.7)	10,213,312 (3,669,096 to 21,324,858)	571.5 (239.1 to 1,320.0)	−28.6 (−60.3 to 26.8)	156,801,283 (131,487,103 to 187,162,945)	−41.0 (−50.3 to −30.2)	−26.3 (−36.8 to −15.1)
Eastern Sub–Saharan Africa	252,389,897 (210,268,128 to 297,240,990)	−49.6 (−55.3 to −43.4)	−31.2 (−38.0 to −24.0)	274,597 (115,496 to 559,228)	—	38.2 (−10.8 to 111.2)	20,415,191 (8,886,884 to 39,007,962)	1,588.0 (578.6 to 4,040.5)	−7.8 (−40.5 to 41.0)	273,079,684 (232,125,392 to 317,441,640)	−46.6 (−52.2 to −40.5)	−29.8 (−36.1 to −23.2)
Central Sub–Saharan Africa	323,076,125 (254,165,463 to 419,281,016)	−28.1 (−39.8 to −14.5)	−36.8 (−48.2 to −24.8)	239,338 (48,121 to 729,088)	—	21.0 (−56.9 to 234.5)	17,788,355 (3,873,758 to 57,171,430)	382.4 (−2.8 to 2,290.0)	−19.4 (−71.2 to 123.0)	341,103,817 (269,445,333 to 438,643,029)	−25.4 (−37.1 to −11.7)	−36.1 (−46.9 to −24.6)

**Table 3:** Deaths of major HIV–negative individuals in 2019, and change of age–standardized deaths during the periods 1990–2010 and 2010–2019 for both sexes.

**Group**	**Drug–susceptible tuberculosis**			**Extensively drug–resistant tuberculosis**			**Multidrug–resistant tuberculosis without extensive drug resistance**			**Tuberculosis**		
	**Number of deaths, 2019**	**Annualized rate of change of age–standardized incidence**		**Number of deaths, 2019**	**Annualized rate of change of age–standardized incidence**		**Number of deaths, 2019**	**Annualized rate of change of age–standardized incidence**		**Number of deaths, 2019**	**Annualized rate of change of age–standardized incidence**	
		**1990–2010**	**2010–2019**		**1990–2010**	**2010–2019**		**1990–2010**	**2010–2019**		**1990–2010**	**2010–2019**
Global	1,317,296 (1,147,331 to 1,473,409)	−53.0 (−57.3 to −48.3)	−29.4 (−36.3 to −22.3)	10,419 (4901 to 18734)	—	−5.6 (−32.8 to 37.6)	135,906 (54,012 to 258,625)	348.4 (114.3 to 929.2)	−21.6 (−52.4 to 27.5)	1,463,621 (1,339,240 to 1,602,711)	−48.9 (−52.0 to −44.4)	−28.6 (−34.4 to −22.2)
High SDI	67,262 (59,261 to 74,320)	−67.8 (−70.3 to −65.5)	−24.4 (−28.9 to −19.1)	661 (287 to 1326)	—	2.1 (−30.0 to 51.3)	2,798 (1,152 to 5,487)	−33.3 (−58.8 to 10.8)	−31.0 (−54.8 to 8.1)	70,722 (62,719 to 77,388)	−66.8 (−69.2 to −64.8)	−24.5 (−28.6 to −19.8)
High–middle SDI	226,851 (186,851 to 259,473)	−64.3 (−69.6 to −59.4)	−38.4 (−46.1 to −30.0)	12,425 (6,810 to 19,394)	—	−26.0 (−44.2 to 2.3)	44,025 (22,300 to 77,554)	171.3 (2.9 to 755.1)	−45.6 (−63.7 to −10.5)	283,301 (261,676 to 307,145)	−56.1 (−59.1 to −52.6)	−39.2 (−44.0 to −34.2)
Middle SDI	976,867 (877,622 to 1,087,632)	−60.7 (−64.3 to −56.8)	−36.5 (−42.0 to −30.2)	8,988 (3,931 to 16,609)	—	−16.1 (−46.6 to 25.3)	79,109 (29,736 to 148,793)	86.5 (−18.4 to 454.3)	−39.6 (−65.5 to 0.9)	1,064,964 (986,626 to 1,165,682)	−57.8 (−60.7 to −53.9)	−36.7 (−40.9 to −31.5)
Low–middle SDI	3,027,314 (2,490,850 to 3,494,543)	−57.9 (−63.3 to −52.3)	−30.8 (−40.4 to −20.5)	20,786 (6,453 to 45,487)	—	18.0 (−43.7 to 118.5)	357,339 (108,036 to 799,780)	811.8 (214.5 to 2,823.3)	−16.5 (−63.2 to 64.1)	3,405,439 (3,010,264 to 3,859,166)	−53.8 (−58.1 to −48.4)	−29.4 (−37.1 to −21.2)
Low SDI	5,472,994 (4,760,874 to 6,280,801)	−47.3 (−52.1 to −40.1)	−30.5 (−37.2 to −23.1)	15,018 (5,331 to 31,846)	—	25.5 (−36.6 to 147.8)	506,356 (209,116 to 921,183)	965.5 (434.6 to 1,966.7)	−15.1 (−45.6 to 39.0)	5,994,367 (5,330,137 to 6,846,309)	−43.4 (−47.7 to −36.8)	−29.3 (−35.7 to −22.0)
High–income	55,620 (49,348 to 59,943)	−69.8 (−71.9 to −68.5)	−22.0 (−25.4 to −18.5)	448 (175 to 946)	—	12.7 (−29.2 to 84.8)	16,99 (667 to 3,632)	−45.4 (−67.0 to −4.3)	−27.9 (−54.7 to 18.2)	57,767 (51,199 to 61,873)	−69.2 (−71.1 to −67.9)	−22.0 (−24.9 to −19.0)
High–income North America	17,825 (16,352 to 18,996)	−71.6 (−73.1 to −69.7)	−5.7 (−10.4 to −1.1)	135 (42 to 328)	—	46.7 (−31.5 to 188.9)	511 (168 to 1,200)	−88.2 (−93.0 to −78.7)	−6.2 (−56.2 to 84.8)	18,471 (17,200 to 19,421)	−72.6 (−73.6 to −71.5)	−5.4 (−9.0 to −1.6)
Australasia	19,915 (15,627 to 24,956)	−60.4 (−65.0 to −55.9)	−12.2 (−25.7 to 3.4)	336 (106 to 786)	—	113.1 (−26.5 to 492.6)	1,279 (406 to 2,958)	31.4 (−58.6 to 397.9)	36.6 (−53.0 to 279.2)	21,531 (17,482 to 26,434)	−59.0 (−63.0 to −54.6)	−9.4 (−21.7 to 5.3)
High–income Asia Pacific	127,176 (108,521 to 140,684)	−73.7 (−76.3 to −71.9)	−28.7 (−33.3 to −24.4)	773 (137 to 2465)	—	1.7 (−68.9 to 159.0)	2,931 (563 to 8,561)	−48.3 (−85.5 to 101.7)	−35.0 (−80.1 to 65.7)	130,880 (112,363 to 143,490)	−73.3 (−75.7 to −71.7)	−28.8 (−32.2 to −25.0)
Western Europe	33,983 (30,329 to 36,885)	−71.3 (−72.8 to −69.8)	−25.1 (−29.5 to −20.4)	418 (181 to 847)	—	13.5 (−21.7 to 59.4)	1,584 (698 to 3,101)	−17.9 (−51.6 to 40.4)	−27.4 (−49.9 to 2.0)	35,985 (32,289 to 38,566)	−70.2 (−71.6 to −68.9)	−24.9 (−29.0 to −20.7)
Southern Latin America	149,755 (134,830 to 162,766)	−68.2 (−70.2 to −66.2)	−21.6 (−28.6 to −14.2)	1,053 (248 to 3,259)	—	−1.9 (−64.4 to 136.8)	4,003 (997 to 12,092)	46.2 (−58.5 to 549.3)	−36.9 (−77.2 to 51.5)	154,811 (143,088 to 167,740)	−67.2 (−68.8 to −65.4)	−22.0 (−27.8 to −15.6)
Central Europe, Eastern Europe, and Central Asia	239,788 (166,733 to 304,989)	−27.3 (−47.4 to −9.2)	−44.9 (−54.5 to −34.3)	50,867 (28,928 to 77,287)	—	−15.9 (−35.5 to 13.0)	111,080 (65,478 to 160,237)	1,839.5 (812.1 to 4,167.6)	−47.6 (−59.9 to −29.8)	401,735 (366,858 to 439,255)	16.1 (13.2 to 21.2)	−43.2 (−48.1 to −38.0)
Eastern Europe	245,360 (151,499 to 339,703)	−4.8 (−36.6 to 25.1)	−51.0 (−63.8 to −34.9)	66,648 (36,789 to 101,647)	—	−15.0 (−38.9 to 24.2)	145,643 (83,153 to 219,320)	1,710.5 (646.4 to 4,723.3)	−47.0 (−62.1 to −22.8)	457,652 (402,407 to 517,082)	57.8 (52.8 to 68.9)	−46.4 (−52.9 to −39.5)
Central Europe	111,364 (96,464 to 127,628)	−60.0 (−63.0 to −57.8)	−36.9 (−45.2 to −27.7)	2,109 (792 to 4,440)	—	−15.7 (−68.2 to 108.9)	4,616 (1,719 to 10,014)	69.0 (−28.2 to 289.3)	−47.3 (−80.3 to 29.8)	118,088 (102,308 to 133,859)	−58.0 (−59.3 to −56.3)	−37.1 (−45.1 to −28.7)
Central Asia	407,026 (268,228 to 533,068)	−42.5 (−59.1 to −26.9)	−37.9 (−51.5 to −20.7)	82,029 (43,857 to 133,860)	—	−21.6 (−45.7 to 7.5)	178,817 (100,017 to 274,260)	7,803.1 (2,987.1 to 26,971.3)	−51.2 (−66.2 to −33.2)	667,872 (596,064 to 755,204)	−1.5 (−5.0 to 2.7)	−40.7 (−47.3 to −33.2)
Latin America and Caribbean	281,371 (242,462 to 322,727)	−73.8 (−75.7 to −71.9)	−29.2 (−36.9 to −20.2)	3,359 (1,342 to 6,864)	—	11.0 (−28.7 to 68.9)	20,295 (8,326 to 41,956)	146.7 (12.2 to 475.0)	−27.6 (−53.7 to 9.7)	305,025 (269,127 to 346,499)	−71.9 (−73.3 to −70.3)	−28.8 (−35.8 to −20.1)
Central Latin America	223,839 (184,989 to 266,927)	−78.2 (−79.6 to −76.9)	−22.9 (−34.6 to −8.9)	2,316 (772 to 5,388)	—	22.4 (−38.5 to 128.7)	14,035 (4,851 to 32,591)	412.4 (111.0 to 1,341.2)	−20.0 (−60.0 to 48.5)	240,190 (205,693 to 283,352)	−76.8 (−77.7 to −75.7)	−22.5 (−33.5 to −9.1)
Andean Latin America	624,191 (470,040 to 792,892)	−80.8 (−84.2 to −77.5)	−39.8 (−52.8 to −25.2)	14,253 (4,888 to 30,166)	—	−10.6 (−49.9 to 52.5)	86,109 (32,036 to 179,472)	63.0 (−40.7 to 457.5)	−41.6 (−67.5 to −1.0)	72,4554 (575,397 to 901,576)	−78.1 (−81.0 to −75.0)	−39.6 (−51.6 to −26.2)
Caribbean	465,542 (372,093 to 587,816)	−53.7 (−61.2 to −44.4)	−12.0 (−24.9 to 4.8)	863 (189 to 2,667)	—	101.1 (−31.2 to 549.7)	5,173 (1,169 to 15,725)	−57.9 (−88.3 to 66.5)	30.8 (−55.3 to 322.1)	471,578 (377,189 to 593,127)	−53.7 (−61.2 to −44.2)	−11.6 (−24.5 to 5.5)
Tropical Latin America	219,804 (189,629 to 240,003)	−64.0 (−66.5 to −61.9)	−32.7 (−40.0 to −27.0)	2,278 (382 to 6,614)	—	35.5 (−62.7 to 225.6)	13,738 (2,587 to 38,203)	1,416.6 (249.9 to 10,740.6)	−11.9 (−75.8 to 111.5)	235,820 (222,893 to 249,304)	−62.2 (−63.6 to −60.4)	−31.5 (−34.7 to −27.4)
Southeast Asia, East Asia, and Oceania	771,555 (697,343 to 852,161)	−65.4 (−68.8 to −61.6)	−38.6 (−44.0 to −31.6)	7,177 (2,860 to 15,216)	—	−25.5 (−55.3 to 20.3)	37,502 (16,030 to 75,935)	−21.9 (−69.3 to 146.2)	−51.7 (−71.1 to −22.2)	816,234 (746,042 to 890,269)	−64.0 (−67.0 to −60.4)	−39.2 (−44.5 to −33.5)
East Asia	193,491 (153,881 to 229,273)	−81.6 (−84.6 to −78.2)	−44.5 (−54.6 to −33.1)	3,136 (685 to 8,749)	—	−37.1 (−80.8 to 52.7)	16,405 (3,650 to 45,583)	−65.4 (−87.8 to 44.3)	−59.2 (−87.6 to −1.2)	213,033 (184,453 to 244,465)	−80.4 (−82.9 to −77.5)	−45.9 (−53.6 to −37.0)
Southeast Asia	257,9881 (2,302,945 to 2,850,244)	−54.6 (−59.7 to −48.5)	−36.8 (−43.1 to −29.0)	19,804 (7,810 to 42,049)	—	−18.6 (−53.9 to 41.0)	103,422 (44,149 to 212,575)	308.1 (60.4 to 1,128.1)	−47.3 (−70.2 to −8.8)	2,703,107 (2,435,044 to 2,965,429)	−52.4 (−57.2 to −46.4)	−37.2 (−43.2 to −30.4)
Oceania	1,994,397 (1,357,173 to 2,679,431)	−36.1 (−45.5 to −25.4)	−25.4 (−37.4 to −12.3)	26,734 (6,233 to 69,366)	—	230.3 (−13.8 to 1,139.2)	139,813 (32,711 to 349,128)	1,441.7 (289.3 to 6,615.2)	113.6 (−44.2 to 701.8)	2,160,944 (1,471,698 to 2,891,979)	−34.4 (−43.9 to −23.4)	−21.4 (−31.5 to −9.2)
North Africa and Middle East	320,544 (263,822 to 399,027)	−64.7 (−69.7 to −54.6)	−36.7 (−43.4 to −25.3)	1,582 (567 to 3,403)	—	−11.2 (−50.9 to 57.3)	21,266 (8,193 to 45,911)	603.4 (234.1 to 1,367.3)	−41.5 (−67.7 to 3.7)	343,392 (285,529 to 423,277)	−62.2 (−67.3 to −51.7)	−36.9 (−42.8 to −25.2)
South Asia	3,119,395 (2,427,290 to 3,725,829)	−61.4 (−67.1 to −55.3)	−32.5 (−44.4 to −19.0)	22,997 (5,435 to 55,147)	—	23.8 (−53.2 to 157.0)	445,982 (106,308 to 1,047,464)	2,077.1 (438.4 to 11,498.4)	−18.2 (−69.0 to 70.0)	3,588,374 (3,145,779 to 4,126,190)	−56.8 (−60.9 to −50.9)	−30.8 (−39.4 to −20.3)
Sub–Saharan Africa	6,420,272 (5,575,936 to 7,434,606)	−39.4 (−45.0 to −32.4)	−30.6 (−37.5 to −23.6)	6,618 (2,753 to 12,825)	—	21.7 (−11.2 to 71.3)	486,964 (218,898 to 865,752)	859.0 (454.6 to 1,583.1)	−18.9 (−41.0 to 14.0)	6,913,854 (6,078,443 to 7,951,553)	−35.7 (−41.1 to −29.2)	−29.8 (−36.1 to −23.2)
Southern Sub–Saharan Africa	5,023,801 (4,382,100 to 5,666,017)	14.1 (−4.3 to 35.6)	−41.7 (−47.7 to −35.3)	5,716 (1,921 to 13,631)	—	−10.3 (−56.8 to 96.3)	420,136 (149,748 to 904,445)	987.1 (237.1 to 4,612.5)	−40.0 (−71.2 to 30.6)	5,449,653 (4,928,930 to 6,011,006)	22.4 (4.7 to 45.2)	−41.5 (−46.3 to −36.3)
Western Sub–Saharan Africa	4,751,910 (3,841,820 to 5,771,344)	−43.7 (−53.2 to −31.4)	−25.1 (−36.0 to −13.5)	4,802 (1,651 to 11,058)	—	9.3 (−39.5 to 96.7)	353,882 (123,347 to 753,343)	639.3 (265.1 to 1,429.2)	−27.1 (−59.8 to 30.9)	5,110,594 (4,280,088 to 6,148,863)	−39.7 (−49.6 to −27.3)	−25.2 (−35.1 to −14.8)
Eastern Sub–Saharan Africa	7,894,787 (6,579,176 to 9,346,079)	−48.1 (−53.9 to −40.9)	−30.8 (−37.0 to −23.9)	8,842 (3,625 to 18,055)	—	40.3 (−7.5 to 115.6)	650,554 (281,043 to 1,262,596)	1,594.5 (591.8 to 4,082.2)	−6.6 (−38.4 to 43.4)	8,554,184 (7,290,283 to 9,901,313)	−45.1 (−50.7 to −38.2)	−29.3 (−35.0 to −22.5)
Central Sub–Saharan Africa	9,577,194 (7,420,478 to 12,726,686)	−22.6 (−35.1 to −6.9)	−33.9 (−45.8 to −21.3)	7,490 (1,472 to 23,347)	—	29.2 (−55.1 to 247.5)	550,241 (115,859 to 1,811,878)	420.4 (4.0 to 2,530.1)	−14.2 (−70.1 to 131.1)	10,134,925 (7,919,764 to 13,250,791)	−19.6 (−32.0 to −3.7)	−33.1 (−44.7 to −20.6)

According to GBD 2019, we analyzed the age-standardized incidence of all HIV negative TB in region-specific and country-specific in 2019 (Table 1). Among more than 21 regions in the world, Southern Sub-Saharan Africa has the highest incidence [34.36 million (29.62–39.72)], followed by Sub-Saharan Africa, Central Sub-Saharan Africa, and Eastern Sub-Saharan Africa. In 2019, the regions with the highest age-standardized DALYs and deaths rates are Central Sub-Saharan Africa (341.10 million (269.44–438.64)] (Table 2) and 10.13 million (7.92–13.25)] (Table 3). In short, in 2019, the regions with the highest age-standardized incidence, DALYs, and deaths rates are all in Africa, and the regions with the lowest burden are high-income North America (Tables 1–3), of which the regions with the highest age-standardized deaths rates It is more than 506 times that of low income areas. In 2019, the age-standardized incidence rate, DALYs rate, and deaths rate of all HIV negative TB (Figures 1J–L; Supplementary Tables S4–S6) are the countries with the heaviest burden in the Central African Republic, and their values are 50.62 million (45.98–55.57), 1,017.09 million (742.75–1,344.40), and 28.08 million (20.71–36.56), respectively. The countries with the least burden are the USA, Andorra, and Bermuda.

From 1990 to 2019, the change of age-standardized incidence rate of all HIV-negative TB (Supplementary Table S7) in 204 countries worldwide decreased in almost all countries except Ukraine, which increased by 10.76% (from −0.12 to 24.57). The country with the largest rate of decline is the USA [−72.08% (from −73.90 to −70.28)], and the country with the smallest decline rate is Sweden [−0.22% (from −9.40 to 9.31)]. For the change of age-standardized DALYs rate of all HIV negative TB (Supplementary Table S7), in addition to Lesotho, Zimbabwe, and Ukraine; these three countries showed an upward trend, and the rest of the countries showed a declined trend. The country with the largest decline is Maldives [−92.20% (from −94.06 to 89.14)]. For the change of age-standardized deaths rate of all HIV negative TB (Supplementary Table S7), among which Ukraine, Tajikistan, Lesotho, and Zimbabwe showed an upward trend, and the rest of the countries showed a declined trend. The country with the largest increase was Ukraine [53.26% (22.00–94.57)], and the country with the fastest decline in deaths is Hungary [−92.50% (from −93.91 to −90.77)]. The change of age-standardized deaths rate of all HIV negative TB, almost all countries have decreased, except for Ukraine, which has increased by 10.76% (from −0.12 to 24.57), and the country with the largest decline is the USA [72.08% (from −73.90 to −70.28)]. We analyzed the changes in 204 countries from 1990 to 2010 and from 2010 to 2019 (Tables 1–3). In addition, due to the lack of total HIV-positive TB data in GBD 2019, we are temporarily unable to analyze the global total HIV-positive TB data from the change of age-standardized incidence, DALYs, and deaths rate from 1990 to 2019.

### Burden of DS-TB and HIV/AIDS-DS-TB

In 2017, the incidence (in thousands) and prevalence (in thousands) of DS-TB were 8,508.6 (7,808.6–9,371.0) and 9,828.6 (8,860.7–10,773.9), respectively (19). In 2015, there were 1.14 million new cases of HIV-1 and TB co-infection, and nearly 400,000 cases died from co-infection (22). In 2017, the number of HIV/AIDS and drug-susceptible TB co-infection cases reached 132 million (19). The median per capita cost of treating drug-sensitive TB in 2019 was US$860.4. According to the WHO report, the disposable US$6.5 billion in 2020, of which the cost for the diagnosis and treatment of drug-susceptible TB is as high as US$4.2 billion (2).

**Figure 3:**
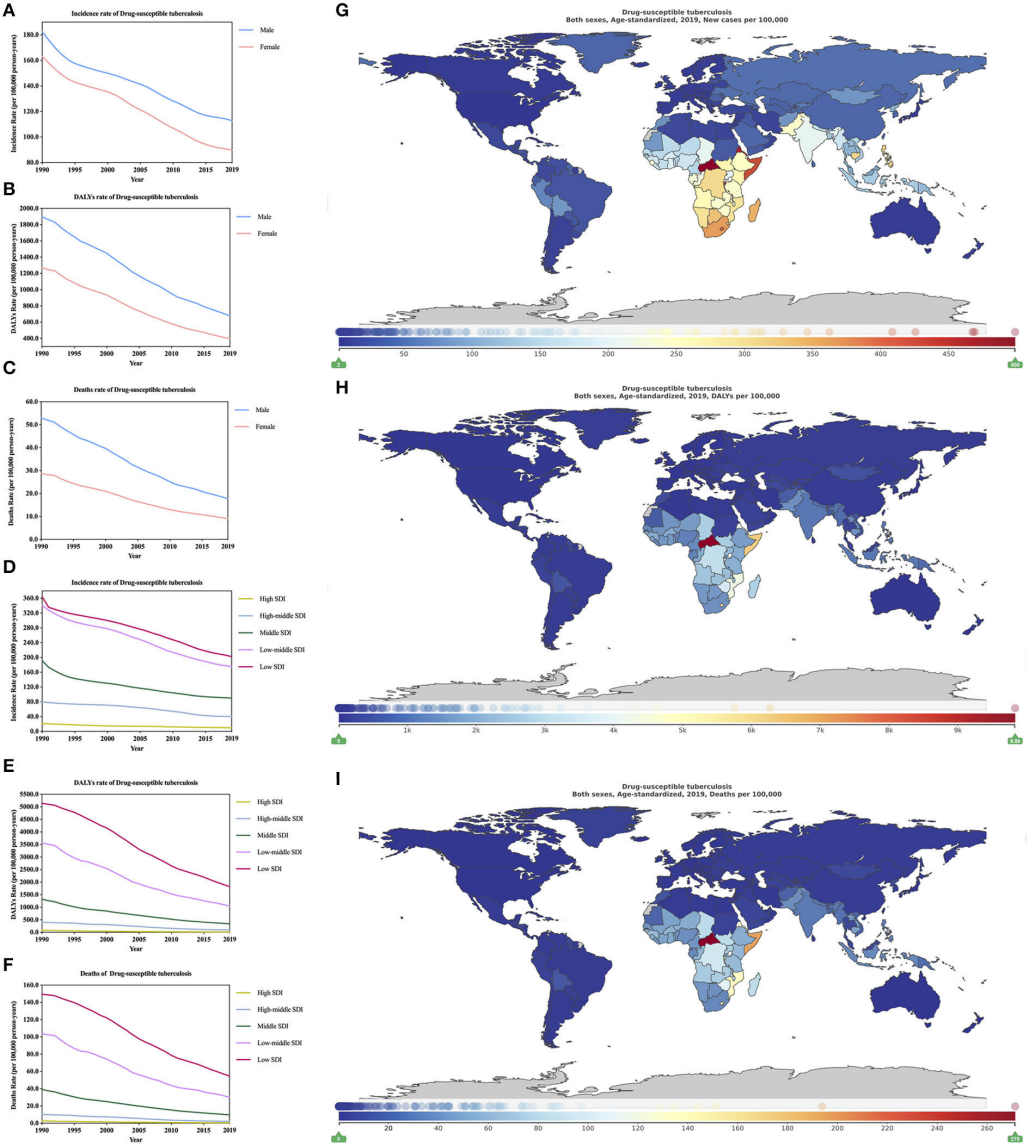
Burden of drug-susceptible tuberculosis for 204 countries and territories. Age-standardized incidence, DALYs, and deaths rate per 100,000 population of drug-susceptible tuberculosis stratified by sex **(A–C)** and SDI **(D–F)** in global (1990–2019) and the distribution of drug-susceptible tuberculosis globally in 2019 **(G–I)** were generated by GDB 2019.

In 2019, age-standardized incidence, DALYs, and deaths rate of DS-TB (95% CI, Supplementary Table S1) are 10.08 million (8.74–11.46), 53.42 million (46.68–59.67), and 1.32 million (1.15–1.47), respectively. In 2019, age-standardized incidence, DALYs, and deaths rate of HIV/AIDS-DS-TB (95% CI, Supplementary Tables S4–S6) are 1.37 million (1.20–1.56), 13.70 million (10.07–17.57), and 0.24 million (0.17–0.32), respectively. From 1990–2019, age-standardized incidence rate, DALYs rate, and deaths rate of DS-TB, males are higher than females (Figures 3A–C; Supplementary Table S8), for HIV/AIDS-DS-TB, the proportion of females is higher than males (Supplementary Figures S1D–F; Supplementary Table S9). In 2019, the increase in the burden of DS-TB is in the opposite order of the increase in SDI. Low SDI locations record the highest age-standardized incidence, DALYs, and deaths rates of DS-TB. However, in high SDI areas, there are lowest rates (Figures 3D–F; Supplementary Table S10). The increase in the burden of HIV/AIDS-DS-TB has similar results with the trend of SDI and DS-TB (Supplementary Figures S1G–I; Supplementary Table S11).

In 2019, in high SDI and low SDI regions, the age-standardized incidence rate of DS-TB (Supplementary Table S10; 1,000 per year) is 982.37 (851.39–1,134.43) and 20,228.73 (17,704.87–22,958.49), respectively, and the low SDI region is high, which is more than 20 times the SDI area. The age-standardized DALYs rate of DS-TB (Supplementary Table S10; 1,000 per year) are 1,864.03 (1,657.62–2,112.64) and 18,193.29 (157,033.17–209,942.48), respectively. The low SDI area is 97 times that of the high SDI area. The age-standardized deaths rate of DS-TB (Supplementary Table S10; 1,000 per year) is 67.26 (59.26–74.32) and 5,472.99 (4,760.87–6,280.80), respectively. The low SDI area is 81 times that of the high SDI area. In 2019, the age-standardized incidence rate of HIV/AIDS-DS-TB (Supplementary Table S11; 1,000 per year) areas with high SDI and low SDI are 30.72 (26.67–35.58) and 3,476.76 (3,037.45–3,977.41), respectively. For age-standardized DALYs rate of HIV/AIDS-DS-TB (Supplementary Table S11; 1,000 per year) are 242.14 (164.86–348.22) and 45,495.49 (34,473.84–56,412.04), respectively. For age-standardized deaths rate of HIV/AIDS-DS-TB (Supplementary Table S11; 1,000 per year) are 5.07 (3.31–7.43) and 891.66 (658.69–1,106.21) respectively. In short, for HIV/AIDS-DS-TB, age-standardized incidence DALYs, and deaths rates in low SDI areas are higher than those in high SDI areas (both are >100 times).

In 2019, among the 21 regions of the world, the highest age-standardized incidence DS-TB of (Table 1) is Southern Sub-Saharan Africa [33.20 million (28.60–38.52)], followed by Central Sub-Saharan Africa and Eastern Sub-Saharan Africa (all >27 million), the region with the lowest burden is high-income North America [0.23 million (0.20–0.27)], the region with the highest burden is more than 142 times that of the lowest region. For age-standardized HIV/AIDS-DS-TB, the region with the highest burden is also Southern Sub-Saharan Africa [39.36 million (30.34–39.36)] (Supplementary Table S12), and the region with the lowest burden is Australasia [140.03 (120.54–164.06), 1,000 per year].

In 2019, for age-standardized DALYs of DS-TB, the region with the highest burden is Central Sub-Saharan Africa [323.07 million (254.17–419.28)], followed by Sub-Saharan Africa and Eastern Sub-Saharan Africa (both are >200 million) (Table 2). For age-standardized DALYs of HIV/AIDS-DS-TB, the region with the highest burden is Southern Sub-Saharan Africa [347.79 million (246.03–460.74)] (Supplementary Table S13). In addition, the area with the lowest burden for age-standardized DALYs of DS-TB and HIV/AIDS-DS-TB is Australasia. In 2019, the region with the highest burden for age-standardized deaths of DS-TB is still Central Sub-Saharan Africa [9.58 million (7.42–12.73)] (Table 3), followed by Eastern Sub-Saharan Africa and Sub-Saharan Africa, the region with the lowest burden is high-income North America. For age-standardized deaths of HIV/AIDS-DS-TB, the highest and lowest burden areas are Southern Sub-Saharan Africa [7.02 million (4.79–9.50)] and Australasia (Supplementary Table S14).

In 2019, the age-standardized incidence rate of DS-TB is different in different countries and regions (Figure 3G; Supplementary Table S4; 1,000 per year). The country with the heaviest burden is the Central African Republic 49,883.99 (45,088.71–54,815.06), followed by Eritrea, Burundi, and the country with the lightest burden is the United States of America [208.72 (177.91–245.42)], and the areas with the heaviest burden are 238 times as many as the areas with light burdens. For age-standardized DALYs (Figure 3H; Supplementary Table S5; 1,000 per year) and deaths rate DS-TB (Figure 3I; Supplementary Table S6; 1,000 per year), the countries with the heaviest burden are the Central African Republic, which are 983,328.10 (717,134.30–1,314,526.54) and 27,131.18 (19,686.98–35,825.78), respectively, the countries with the least burden are Malta [478.23 (382.87–598.79)] and Bermuda [6.38 (5.14–7.98)]. The countries with the highest and lowest age-standardized DALYs burden are 2,056 times the difference. The difference between the countries with the highest and lowest age-standardized deaths burden is 4,283 times. For the age-standardized incidence rate of HIV/AIDS-DS-TB (Supplementary Table S15; 1,000 per year) global distribution is analyzed. The country with the heaviest burden is Lesotho [77,828.41 (65,784.42–91,494.82)], and the country with the lightest burden is Iraq [0.70 (0.59–0.82)]. The age-standardized incidence and deaths rate DS-TB (Supplementary Tables S16, S17; 1,000 per year) also show that the country with the heaviest burden is Lesotho, which is 1,112,636.76 (872,521.60–1,403,125.61)] and 23,229.32 (18,518.84–28,711.45), respectively, the country with the least burden is Iraq, which is 5.44 (3.38–8.61) and 0.08 (0.05–0.14), respectively.

From 1990 to 2019, the change of age-standardized incidence of DS-TB (Supplementary Table S18) has dropped by 41.2% (from −44.4 to −38.3) globally, and all countries have fallen, of which Belarus has the largest decline [73.9% (from −80.7 to −66.9)], Sweden has the smallest decrease [3.1% (from −12.9 to 6.6)]. The change of age-standardized incidence of HIV/AIDS-DS-TB (Supplementary Table S19) is on the rise globally [44.5% (36.2–53.2)], and the existing data shows that 80 countries are on the rise, Mongolia has the largest increase (approximately 56 times). For change of age-standardized DALYs rate of DS-TB (Supplementary Table S18), it shows a downward trend in the world [66.00 (from −70.52 to −61.29]. Except for Lesotho and Zimbabwe, which showed an increasing trend, all other countries showed a downward trend. The largest amplitude is Guatemala [90.0 (from −94.2 to −90.0)]. For change of age-standardized DALYs rate of HIV/AIDS-DS-TB (Supplementary Table S19), the country with the largest increase is Papua New Guinea, which is as high as 105.8 times (95% CI, 35.5–369.3), and the country with the largest decrease is Hungary [95.2% (from −96.0 to −94.4)]. For change of age-standardized deaths rate of DS-TB (Supplementary Table S18) decreased by [41.2% (from −44.4 to −38.3)] globally, except for Lesotho and Zimbabwe, which showed an increasing trend. The rest countries are showing a downward trend, with the largest decline being Hungary [92.7% (from −94.3 to −91.0)]. For change of age-standardized deaths rate of HIV/AIDS-DS-TB (Supplementary Table S19), the country with the largest increase is Papua New Guinea, which is as high as 167.6 times (95% CI, 46.8–4,767.8), and the country with the largest decrease is Hungary [95.5% (from −96.3 to −94.6)]. In addition, more detailed information can be learned through the changes in the 204 countries from 1990 to 2010 and from 2010 to 2019 (Supplementary Tables S4–S6, S15–S17).

### Burden of MDR-TB without XDR and HIV/AIDS-MDR-TB without XDR

Globally, 4.6% of TB patients have MDR-TB, but in some regions, such as Kazakhstan, Kyrgyzstan, Moldova, and Ukraine, this proportion exceeds 25% (23). Of the estimated total in 2020, US$8.3 billion (64%) is for diagnosis and treatment of drug-susceptible TB, US$4.3 billion is for diagnosis and treatment of MDR-TB (2). In 2019, age-standardized incidence, DALYs, and deaths rate of MDR-TB without XDR (95% CI, Supplementary Table S1) are 0.56 million (0.31–0.97), 5.24 million (2.64–9.76) and 0.14 million (0.05–0.26). In 2019 age-standardized incidence, DALYs, and deaths rate of HIV/AIDS-MDR-TB without XDR (95% CI, Supplementary Table S3) are 0.06 million (0.04–0.08), 1.34 million (0.58–2.46), and 0.02 million (0.01–0.05), respectively. We found that about one-third of people with HIV-infected MDR-TB without XDR died, which indicates that HIV infection is associated with increased mortality during multi-drug resistance (24).

**Figure 4:**
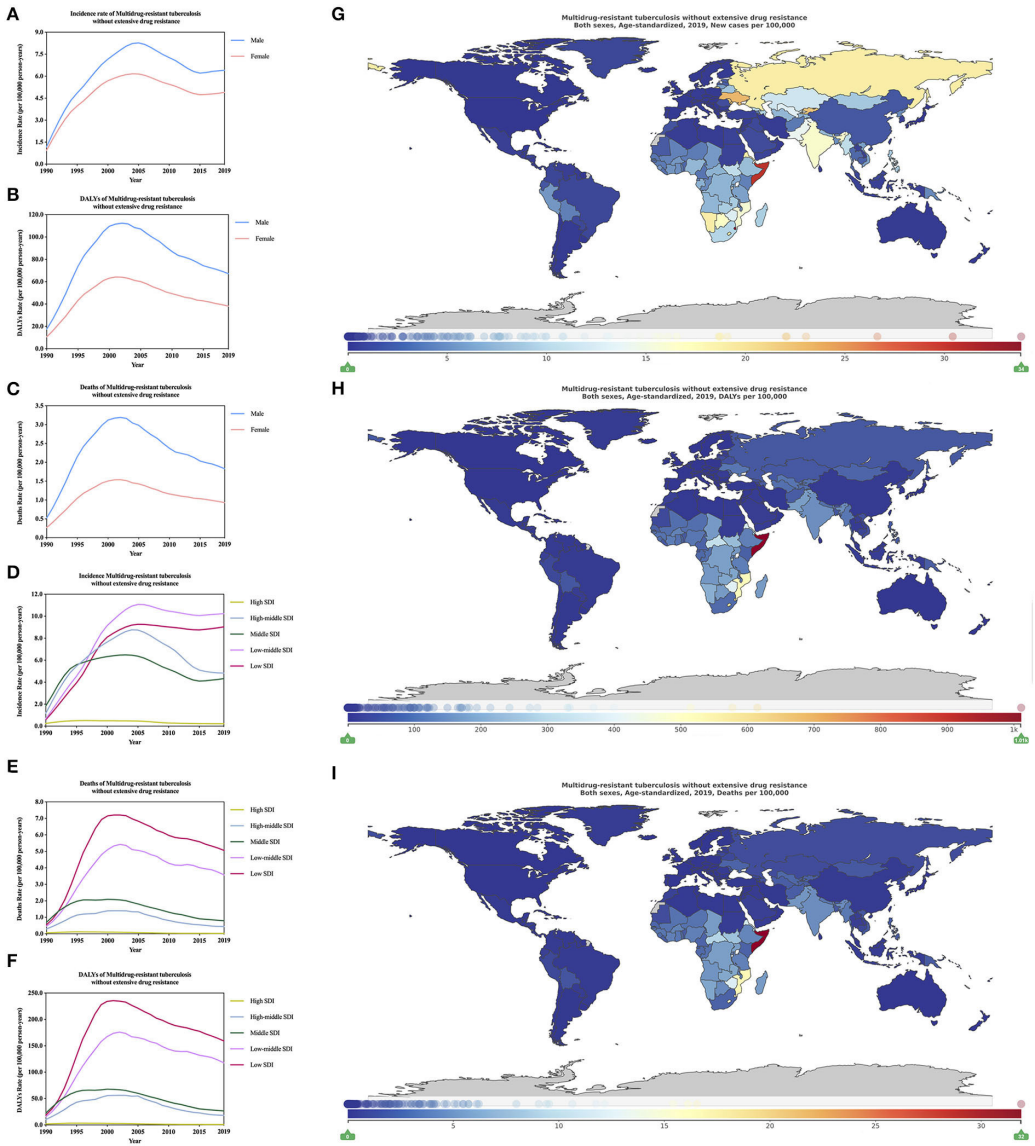
Burden of multidrug-resistant tuberculosis without extensive drug resistance for 204 countries and territories. Age-standardized incidence, DALYs, and deaths rate per 100,000 population of multidrug-resistant tuberculosis without extensive drug resistance stratified by sex **(A–C)** and SDI **(D–F)** in global (1990–2019) and the distribution of multidrug-resistant tuberculosis without extensive drug resistance globally in 2019 **(G–I)** were generated by GDB 2019.

From 1990 to 2019, the age-standardized incidence rate, DALYs rate, and deaths rate of MDR-TB without XDR between different genders all show that male MDR-TB without XDR is always higher than female (Figures 4A–C, Supplementary Table S20). For age-standardized incidence rate, DALYs rate, and deaths rate of HIV/AIDS-MDR-TB without XDR, women's MDR-TB without XDR is generally higher than men's, which is contrary to the results of MDR-TB without XDR (Supplementary Figures S2A–C; Supplementary Table S21). In 2019, the age-standardized incidence rate of MDR-TB without XDR (Supplementary Table S22; Figure 4D; 1,000 per year) is the low-middle SDI region [1024.30 (390.38–2262.37)], and the least burden is High SDI areas [22.41 (13.72–22.41)], and the incidence of low-middle SDI areas is 45 times that of high SDI areas. For the age-standardized DALYs rate of MDR-TB without XDR (Supplementary Table S22; Figure 4E), the heaviest burden is the low SDI area [15,935.64 (6,985.78–28,018.76)], and the lightest burden is the high SDI area [83.81 (37.32–173.54)], the incidence in low SDI areas is 190 times that of high SDI areas. For the age-standardized deaths rate of MDR-TB without XDR (Supplementary Table S22; Figure 4F), the lowest burden is in low SDI regions [506.36 (209.12–921.18)], and the least burden is in high SDI regions [2.80 (1.15) – 5.49)]. In 2019, the difference from MDR-TB without XDR is the age-standardized incidence rate of HIV/AIDS-MDR-TB without XDR (Supplementary Table S23; Supplementary Figure S2D). The region with the heaviest burden is low SDI [148.38 (92.05–230.78)], and the areas with the least burden are high SDI areas [0.86 (0.56–1.27)]. In addition, the age-standardized incidence rate of MDR-TB without XDR is 6.9 times that of MDR-TB without XDR. Similar to the age-standardized incidence rate of HIV/AIDS-MDR-TB without XDR, age-standardized DALYs and deaths rate of MDR-TB without XDR (Supplementary Table S23; Supplementary Figures S2E,F), the areas with the heaviest burden are low SDI regions are 4,499.35 (1,771.05–8,649.15) and 89.20 (34.64–173.04), respectively; the least burdened regions are high SDI regions, which are 13.29 (5.54–27.29) and 0.28 (0.11–0.57), respectively.

In 2019, the regions with the highest burden of age-standardized incidence rate, DALYs rate, and deaths rate of MDR-TB without XDR are Eastern Europe [1.89 million (1.18–2.78)] (Table 1), Eastern Europe Sub-Saharan Africa [20.42 million (8.87–39.00)] (Supplementary Table S2) and Eastern Sub-Saharan Africa [0.65 million (0.28–1.26)] (Supplementary Table S3), the areas with the lowest burden are high-income North America in 21 regions of the world. For age-standardized incidence rate, DALYs rate, and deaths rate of HIV/AIDS-MDR-TB without XDR, the regions with the highest burden are Southern Sub-Saharan Africa [1.42 million (0.73–2.67)] (Supplementary Table S12), [31.95 million (11.58–65.82)] (Supplementary Table S13), and [0.66 million (0.23–1.38) (Supplementary Table S14), respectively. It is worth mentioning that among the 21 regions in the world, the age-standardized deaths rate of HIV/AIDS-MDR-TB is higher than that of MDR-TB without XDR, and the age-standardized incidence rate of HIV/AIDS-MDR-TB is the lowest. The region is also high-income North America. The regions with the lowest burden of age-standardized DALYs rate and deaths rate are in Australasia.

In 2019, the age-standardized incidence rate of MDR-TB without XDR (Supplementary Table S24; Figure 4G; 1,000 per year) is different in 204 countries. The country with the heaviest burden is Eswatini in Africa [3 386.25 (813.60–8,316.47)], the country with the lightest burden is Slovenia [0.49 (0.07–1.79)], and the highest region is 6,910 times that of the lowest region. The country with the highest burden of age-standardized DALYs and deaths rates of MDR-TB without XDR is Somalia and the values are [101,091.76 (23,053.54–277,892.34)] (Supplementary Table S24; Figure 4H) and [3,189.81 (711.50–8,858.71)] (Supplementary Table S24; Figure 4I), respectively. The least burdened country is Slovenia. For the age-standardized incidence rate of HIV/AIDS-MDR-TB without XDR (Supplementary Table S25; Supplementary Figure S2G), the country with the heaviest burden is Eswatini in southern Africa [7,000.85 (1,657.59–17,734.44)] The country with the lightest burden is Slovenia [0.01 (0.00–0.02)]. For age-standardized DALYs (Supplementary Table S25; Supplementary Figure S2H) and deaths rate of HIV/AIDS-MDR-TB without XDR (Supplementary Table S25, Supplementary Figure S2I) both show that the country with the heaviest burden is Eswatini, respectively, 167,061.73 (38,554.53–362,196.88) and 3,503.48 (792.18–7,698.64); the country with the least burden is Slovenia. HIV/AIDS-MDR-TB without XDR and MDR-TB without XDR have a heavy burden in Africa, and Africa should be a key area for global TB prevention and treatment.

From 1990 to 2019, the change of age-standardized incidence of MDR-TB without XDR (Supplementary Table S26) showed an upward trend in the world, rising by 4.37 times (1.03–14.11), and Slovenia had the largest decline [91.55% (from −99.08 to −18.55)], Kyrgyzstan increased [278.08 times (49.95–3,288.03)]. The change of age-standardized DALYs rate (Supplementary Table S26) of MDR-TB without XDR is on the rise globally [2.76 times (0.59 to 8.61)], and the largest increase is Tajikistan [135.31 times (28.89–1,517.36)] Slovenia [97.02% (from −71.48 to −99.68)] has the most significant decline. The MDR-TB without XDR change of age-standardized deaths rate (Supplementary Table S26) was 2.51 times (0.46–8.00), the largest increase was Tajikistan [162.74 times (35.39 to 1,783.52)], and the decrease was 96.87% (from −99.67 to −70.04). For change of age-standardized incidence of HIV/AIDS-MDR-TB (Supplementary Table S27), it is on the rise globally [15.75 times (8.60–27.41)], and the country with the largest increase is Mongolia (4,399.00 times). For change of age-standardized DALYs rate of HIV/AIDS-MDR-TB (Supplementary Table S27) is on the rise globally [17.36 times (9.10–30.48)], the countries with the largest increase are Djibouti and Papua New Guinea (both are greater than 10,000 times). The HIV/AIDS-MDR-TB change of age-standardized deaths rate (Supplementary Table S27) is on the rise globally, increasing by 18.15 times (9.62–32.15). The countries with the largest increase are Djibouti and Papua New Guinea (both are >14,000 times). Supplementary Tables S4–S6, S15–S17 show in detail the changes in the two phases of 1990–2010 and 2010–2019.

### Burden of XDR-TB and HIV/AIDS-XDR-TB

It is noted that XDR-TB, like MDR-TB, remains a major challenge faced by clinicians and staff in global TB prevention and treatment (25). In 2019, age-standardized incidence, DALYs, and deaths rate of XDR-TB (95% CI, Supplementary Table S1; per 1,000 person-years) are 31.01 (21.15–45.11), 38.15 (18.91–66.83), and 10.42 (4.90–18.73), respectively. In 2019, age-standardized incidence, DALYs, and deaths rate of HIV/AIDS-XDR-TB (95% CI, per 1,000 person-years; Supplementary Table S3) are 2.10 [1.51–2.90], 64.23 (28.64–117.74), and 1.01 (0.42–1.86), respectively.

**Figure 5:**
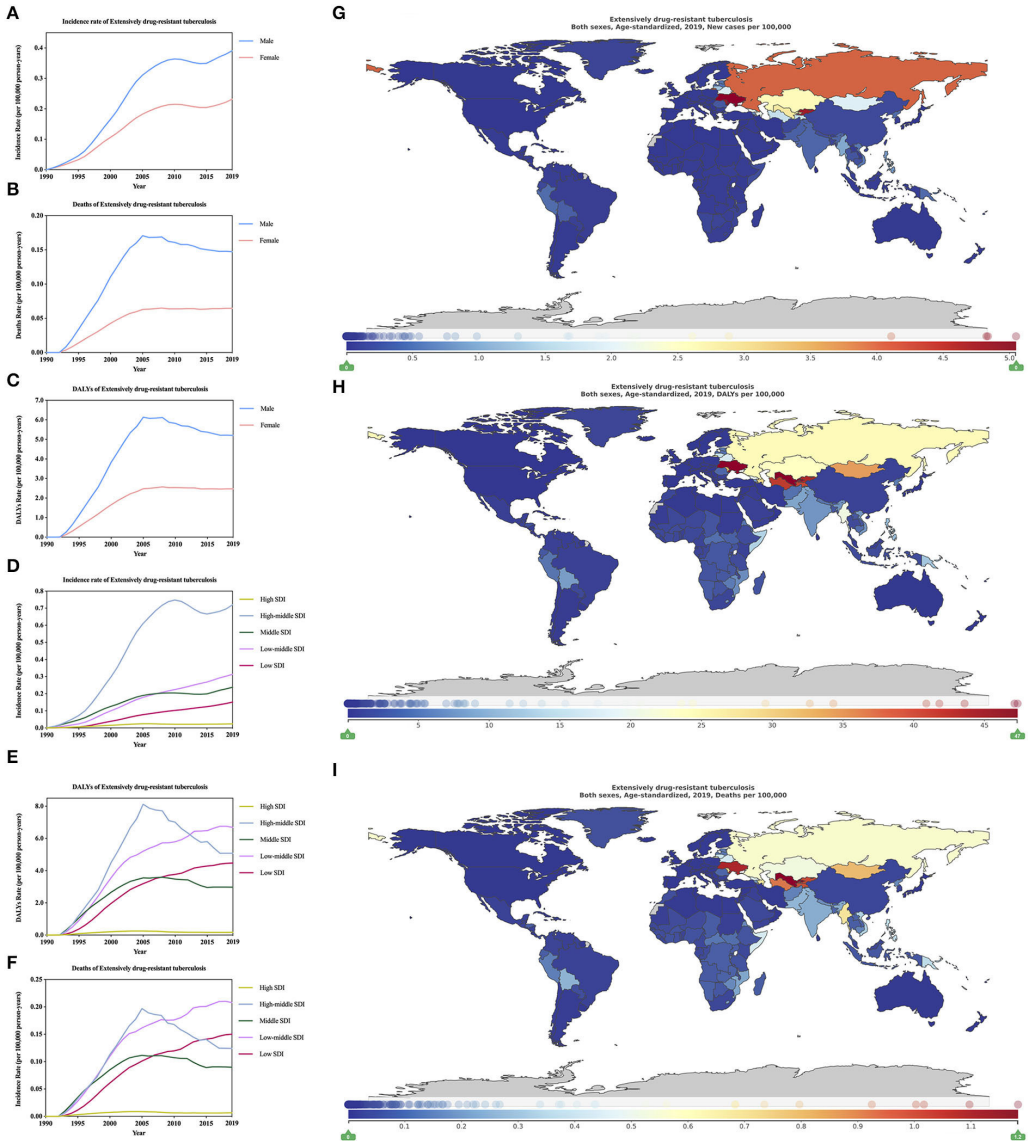
Burden of extensively drug-resistant tuberculosis for 204 countries and territories. Age-standardized incidence, DALYs, and deaths rate per 100,000 population of extensively drug-resistant tuberculosis stratified by sex **(A–C)** and SDI **(D–F)** in global (1990–2019) and the distribution of extensively drug-resistant tuberculosis globally in 2019 **(G–I)** were generated by GDB 2019.

In 1990–2019, age-standardized incidence rate, DALYs rate, and deaths rate of XDR-TB all showed that male XDR-TB is always higher than female (Figures 5A–C; Supplementary Table S28). In 2019, age-standardized incidence rate, DALYs rate, and deaths rate of XDR-TB show (Supplementary Table S28, per 1000 person-years), males are 39.10 (26.83–56.47), 520.45 (261.99–902.84), and 14.73 (7.06–26.12), respectively; females are 23.18 (15.65–34.32), 247.24 (114.94–455.83), and 6.46 (2.86–12.30), respectively. For age-standardized incidence rate, DALYs rate, and deaths rate of HIV/AIDS-MDR-TB without XDR also shows that men are higher than women (Supplementary Figures S3A–C; Supplementary Table S29; per 1,000 person-years). Males are 2.56 (1.81–3.57), 60.95 (26.89–111.33), and 1.19 (0.51–2.19), respectively; females are 1.65 (1.21–2.24), 43.00 (18.76–78.31), and 0.80 (0.35–1.49), respectively. In 2019, the age-standardized incidence rate of XDR-TB (Figure 5D; Supplementary Table S30; per 1,000 person-years) is the area with the heaviest burden in high-middle SDI [72.07 (48.40–102.38)], and the area with the lowest burden, it is the high SDI area [2.43 (1.61–3.87)]. Moreover, the incidence rate in high-middle SDI areas is more than 29 times that of high SDI areas. For age-standardized DALYs rate of XDR-TB (Figure 5E; Supplementary Table S30; per 1,000 person-years), the heaviest burden is the low-middle SDI area [668.62 (221.46–1,426.01)], the least burden is high SDI area [17.27 (8.18–33.060)]. For age-standardized deaths rate of XDR-TB (Figure 5F; Supplementary Table S30; per 1,000 person-years), the area with the heaviest burden is low-middle SDI [20.19 (6.45–45.49)], and the area with the lightest burden is high SDI area [0.66 (0.29–1.33)]. The area with the heaviest burden of mortality is 30 times as much as the area with the least burden, which is consistent with the incidence rate results. In 2019, the age-standardized incidence rate of HIV/AIDS-XDR-TB (Supplementary Figure S3D; Supplementary Table S31; per 1,000 person-years), the highest burden is high-middle SDI area [5.34 (3.41–7.77)], the least burden is high SDI area [0.10 (0.07–0.15)], the heaviest burden is 53 times that of the least burdened area. For age-standardized DALYs rate of HIV/AIDS-XDR-TB (Supplementary Table S3E; Supplementary Figure S31; per 1,000 person-years). The highest burden is in the high-middle SDI area [85.31 (40.72–146.91)], and the lightest burden is also in the high SDI area [3.35 (1.36–6.87)]. For age-standardized deaths rate of HIV/AIDS-XDR-TB (Supplementary Figure S3F; Supplementary Table S31; per 1,000 person-years), the heaviest burden is the high-middle SDI area [1.63 (0.75–2.83)], the least burden is high SDI area [0.07 (0.03–0.15)] In contrast, low SDI regions are not the most affordable regions. This trend is different from DS-TB and MDR-TB without XDR.

In 2019, among 21 regions in the world (Table 1), the age-standardized incidence of XDR-TB, the highest region is Eastern Europe [0.41 million (0.26–0.61)], For age-standardized incidence of HIV/AIDS-XDR-TB, the highest region is also Southern Sub-Saharan Africa [34.62 million (30.34–39.36)] (Supplementary Table S12). For age-standardized DALYs of XDR-TB, the region with the highest burden is Central Asia [3.56 million (1.93–5.75)], followed by Central Sub-Saharan Africa (Table 2). For age-standardized DALYs of HIV/AIDS-XDR-TB, the region with the highest burden is Southern Sub-Saharan Africa [347.79 million (246.03–460.74)] (Supplementary Table S13). The region with the highest burden for age-standardized deaths of XDR-TB is still Central Asia [0.08 million (0.04–0.13)], followed by Eastern Europe (Table 3). For age-standardized deaths of HIV/AIDS-MDR-TB, the region with the highest burden is Eastern Europe [9.63 (95% CI, 4.46–16.79)] per 1,000 person-years, (Supplementary Table S14). For age-standardized incidence, DALYs, and deaths rate of XDR-TB, the region with the lowest burden is high-income North America, and for age-standardized incidence DALYs, and deaths rate of HIV/AIDS-MDR-TB, the region with the lowest burden is Australasia.

In 2019, the age-standardized incidence rate of XDR-TB (Figure 5G; Supplementary Table S4; per 1,000 person-years) is the country with the heaviest burden in Europe [504.89 (269.41–792.53)], and the country with the least burden is Slovenia [0.11 (0.02–0.39)]. For age-standardized DALYs rate of XDR-TB (Figure 5H; Supplementary Table S5), the country with the heaviest burden is Uzbekistan [4,733.82 (1,820.45–8,760.31)], followed by Ukraine, Kyrgyzstan, Turkmenistan, and the least burden country is Andorra [0.53 (0.05–2.37)]. For age-standardized deaths rate of XDR-TB (Figure 5I; Supplementary Table S6), the country with the heaviest burden is Uzbekistan [118.30 (44.54–220.48)], and the country with the lightest burden is Bermuda. In 2019, age-standardized incidence rate of HIV/AIDS-XDR-TB (Supplementary Figure S3G; Supplementary Table S15), the country with the heaviest burden is Ukraine [58.21 (30.04–93.29)], the country with the lightest burden is Iraq. For age-standardized DALYs, and deaths rate of HIV/AIDS-XDR-TB, the countries with the heaviest burden are Eswatini, 2,261.59 (455.28–5,543.59) (Supplementary Figure S3H; Supplementary Table S16) and 47.94 (9.64–114.62) (Supplementary Figure S3I; Supplementary Table S17), the countries with the least burden are Qatar. In short, unlike XDR-TB, countries with a heavier burden of age-standardized incidence, DALYs, and deaths rate of HIV/AIDS-XDR-TB are basically in Africa.

The GBD 2019 lacks relevant data on 1990–2019 change of age-standardized incidence, DALYs, and deaths rate of XDR-TB and HIV/AIDS-XDR-TB, but we have analyzed the relevant changes from 2010–2019. From 2010 to 2019, the change of age-standardized incidence of XDR-TB (Supplementary Table S4) is on the rise globally [7.51% (from −23.02 to 51.28)], of which Papua New Guinea has increased by 3.07 times (from −1.17 to 14.66) For change of age-standardized DALYs rate of XDR-TB (Supplementary Table S5), there is a downward trend in the world [−8.21% (from −33.68 to 31.82)], with the largest decline in Iceland [79.81% (from −96.02 to −31.56). For change of age-standardized deaths rate of XDR-TB (Supplementary Table S6) globally decreased by 5.62% (from −32.75 to 37.62), the largest decrease was Iceland [−80.57% (from −96.19 to −34.25)]. From 2010–2019, for change of age-standardized incidence of HIV/AIDS-XDR-TB (Supplementary Table S15), there is an upward trend in the world [3.36% (from −23.04 to 38.91)], the largest increase in Comoros [3.24 times [−0.12 to 18.99)]. For change of age-standardized DALYs of HIV/AIDS-XDR-TB (Supplementary Table S16) showed a global downward trend [−21.18% (from −36.02 to −0.97)], the largest decline The country is Iceland [−87.42% (from −97.57 to −59.17)]. For HIV/AIDS-XDR-TB change of age-standardized deaths rate (Supplementary Table S17), the global trend is declining [−19.05% (−34.50 to 1.55)], the country with the largest decline was also Iceland [80.36% (−96.22 to −36.28)].

## Discussion

This study evaluated the level and trend of the burden of TB classified by drug resistance type and HIV status in 204 countries and regions in the past 30 years, focusing on the relationship between different types of TB and socioeconomic status (SDI). From 1990 to 2019, the global HIV-negative TB showed a downward trend. The age-standardized incidence rate dropped by 38.15%, the age-standardized DALYs dropped by 62.75%, and the age-standardized deaths rate dropped by 63.49%. However, the age-standardized incidence of HIV-positive TB is increasing, and it is more significant in sub-Saharan Africa. In addition, the age-standardized incidence, DALYs and deaths rates of TB vary greatly from country to country.

The HIV-related TB and drug-resistant TB have become the main burden of drug-resistant TB worldwide. We found that HIV-negative TB is inversely proportional to SDI. Countries with a low burden of age-standardized incidence, DALYs, and deaths rate are in high SDI regions, and countries with high burdens are in low SDI regions. HIV-positive TB (except HIV/AIDS-XDR-TB), age-standardized incidence, DALYs, and deaths rate of HIV/AIDS-DS-TB and HIV/AIDS-MDR-TB countries with the highest burden are in high SDI areas, the burden of the lowest countries are in low SDI regions. The age-standardized incidence of HIV-negative TB in men is higher than that in women. For HIV-positive TB, in addition to HIV/AIDS-XDR-TB, the other two types of HIV-positive TB both show that women are higher than men. Although GBD2017 reported that the global age-standardized incidence rate of HIV-positive TB women is lower than that of men (26), the age-standardized incidence rate of HIV/AIDS-DS-TB and HIV/AIDS-MDR-TB in women is higher than that in men. This may be because women are more susceptible to TB after being infected with HIV than men.

In the past 30 years, the deaths rate of TB has been decreasing year by year, and the prevention and control of TB have achieved remarkable results (25). However, the age-standardized mortality rate in many countries has fallen much faster than the age-standardized morbidity rate. Both AIDS and TB can easily lead to poverty and death, and there is a strong interaction between the two. The incidence of HIV-positive TB is increasing, which reflects that the current prevention and control of HIV-positive TB is facing quite severe challenges, especially in Africa, which is facing the burden of dual infections of AIDS and TB. The previous studies have also concluded that Africa has the highest HIV prevalence rate, and that a large proportion of TB is related to HIV, and the deaths rate of TB among people living with HIV is very high (27). All in all, in terms of TB prevention and control, the African region is currently the focus of global attention (6). While preventing TB, it is necessary to prevent the spread of HIV in a timely manner. Both can be truly controlled under good control.

Although the deaths rate of TB has declined, according to GBD 2019, it is estimated that there will still be about 1.46 million deaths among HIV-negative TB patients worldwide, and about 0.27 million deaths among HIV-positive TB patients in 2019. In HIV-negative and HIV-positive TB, the estimate of GBD 2019 is higher than the estimate of HIV-negative (1.2 million) and HIV-positive TB [208,000] in the WHO2020 report (2). This may be due to the GBD 2019 method of calculating the burden of TB. Unlike the method of the World Health Organization, the estimated value may be different in some regions, so there may be some differences in the global mortality rate. In many countries where TB is endemic, the incidence of TB has either stagnated or declined more slowly than the deaths rate. This indicates that diagnosis and treatment have been delayed. According to the type of TB, appropriate treatment strategies should be adopted to avoid drug resistance (28). The production of sexual TB. Many factors will affect the prevention and control of TB, and there will be unpredictable results. For example, the recent pandemic of coronavirus disease 2019 (COVID-19) may reverse the progress made in TB prevention and control. According to WHO estimates, in 2020 alone, the number of deaths from TB worldwide may increase by 2 to 400,000, and in 2020 (January–June) Several countries (India, Indonesia, Philippines, and South Africa) that account for 44% of global TB have reported a significant drop in the number of confirmed cases, especially India. Indonesia and the Philippines have dropped by 25–30% compared to the same period in 2019 (2). Studies have reported that the cumulative number of deaths from TB in China, India and South Africa affected by COVID-19 is about 0.20 million, and the cumulative number of deaths from TB has increased by 8–14% (29).

Our research still has some limitations. (1) The data in GBD 2019 are estimates, and the lack of overall HIV-positive TB data is not conducive to the assessment of overall HIV-positive TB. (2) In 1990–2010, the data of change of HIV/AIDS-MDR-TB without XDR incidence, DALYs and deaths rate is insufficient in some countries and regions. (3) This study did not analyze the epidemiology of TB, the incidence of TB in various age groups, DALYs and deaths rates, including the status of potentially infected TB. Nevertheless, GBD2019 still shows us a wealth of data for researchers to use, macroscopically showing the trend and burden of TB. We conduct a comprehensive and systematic assessment of the global burden of TB, which provides vital information for reducing the burden of TB.

In 2017, low-income and middle-income countries spent US$10.90 billion (10.3–11.8) on TB and US$20.20 billion (17.0–25.0) on HIV/AIDS (30). World Health Organization predicts that the world's TB will be affected by 2022. At least US$13 billion per year is provided for prevention, diagnosis, treatment, and care (2). Access to treatment may reduce the number of deaths from TB. A lot of funds are still needed to support TB treatment, prevention, and control. Despite a concerted global effort to reduce the burden of TB, it still causes a huge burden of disease globally. Strengthening the health system to detect TB early and improve the quality of TB care, including timely and accurate diagnosis, early initiation of treatment, and regular follow-up are the priorities. In terms of the level of socio–demographic development, countries with a higher incidence of TB than expected should investigate the causes of backwardness and take remedial measures. To achieve the 2035 global target, public health cooperative organizations need to work harder to reduce TB incidence; DALYs, and deaths; expand TB preventive treatment; use new TB treatment options; and prevent and control HIV will help reduce TB (27).

## Data availability statement

The original contributions presented in the study are included in the article/supplementary material, further inquiries can be directed to the corresponding author/s.

## Author contributions

C-YJ, JiaZ, Z-hF, and YX: study conception and design. YX, JiaZ, PW, and J-hL: data acquisition and analysis. JieZ and B-NX: organize the article table section. W-qL and Y-YF: draw the picture. C-YJ: study supervision. Z-hF: administrative support. YX: drafting the manuscripts. C-YJ, JiaZ, and Z-hF: critical revision of the manuscript. All authors contributed to the article and approved the submitted version.

## Funding

This study was supported by the Starting Package of Xiang'an Hospital of Xiamen University (PM201809170010), Open project of Provincial Key Laboratory of Union Hospital Affiliated to Fujian Medical University in 2020 (Nos. XHZDSYS202004 and XHZDSYS202005), and Xiamen municipal Bureau of Science and Technology Grant (3502Z20174079), and the National Natural Science Foundation of China (Grant numbers: 82003178).

## Conflict of interest

The authors declare that the research was conducted in the absence of any commercial or financial relationships that could be construed as a potential conflict of interest.

## Publisher's note

All claims expressed in this article are solely those of the authors and do not necessarily represent those of their affiliated organizations, or those of the publisher, the editors and the reviewers. Any product that may be evaluated in this article, or claim that may be made by its manufacturer, is not guaranteed or endorsed by the publisher.

## Abbreviations

DS-TB: Drug-susceptible tuberculosis
MDR-TB without XDR: Multidrug-resistant tuberculosis without extensive drug resistance
XDR-TB: extensively drug-resistant tuberculosis
HIV/AIDS-DS-TB: HIV/AIDS-drug-susceptible tuberculosis
HIV/AIDS-MDR-TB without XDR: HIV/AIDS-multidrug-resistant tuberculosis without extensive drug resistance
HIV/AIDS-XDR-TB: HIV/AIDS-extensively drug-resistant tuberculosis
DALYs: disability-adjusted life years
SDI: sociodemographic Index
COVID-19: coronavirus disease 2019.
