# Supplementary figures and images for: Burden of tuberculosis and its association with socio-economic development status in 204 countries and territories, 1990–2019
Source: Front Med (Lausanne). 2022 Jul 22;9:905245. doi: 10.3389/fmed.2022.905245 (PMC9355511; doi:10.3389/fmed.2022.905245)

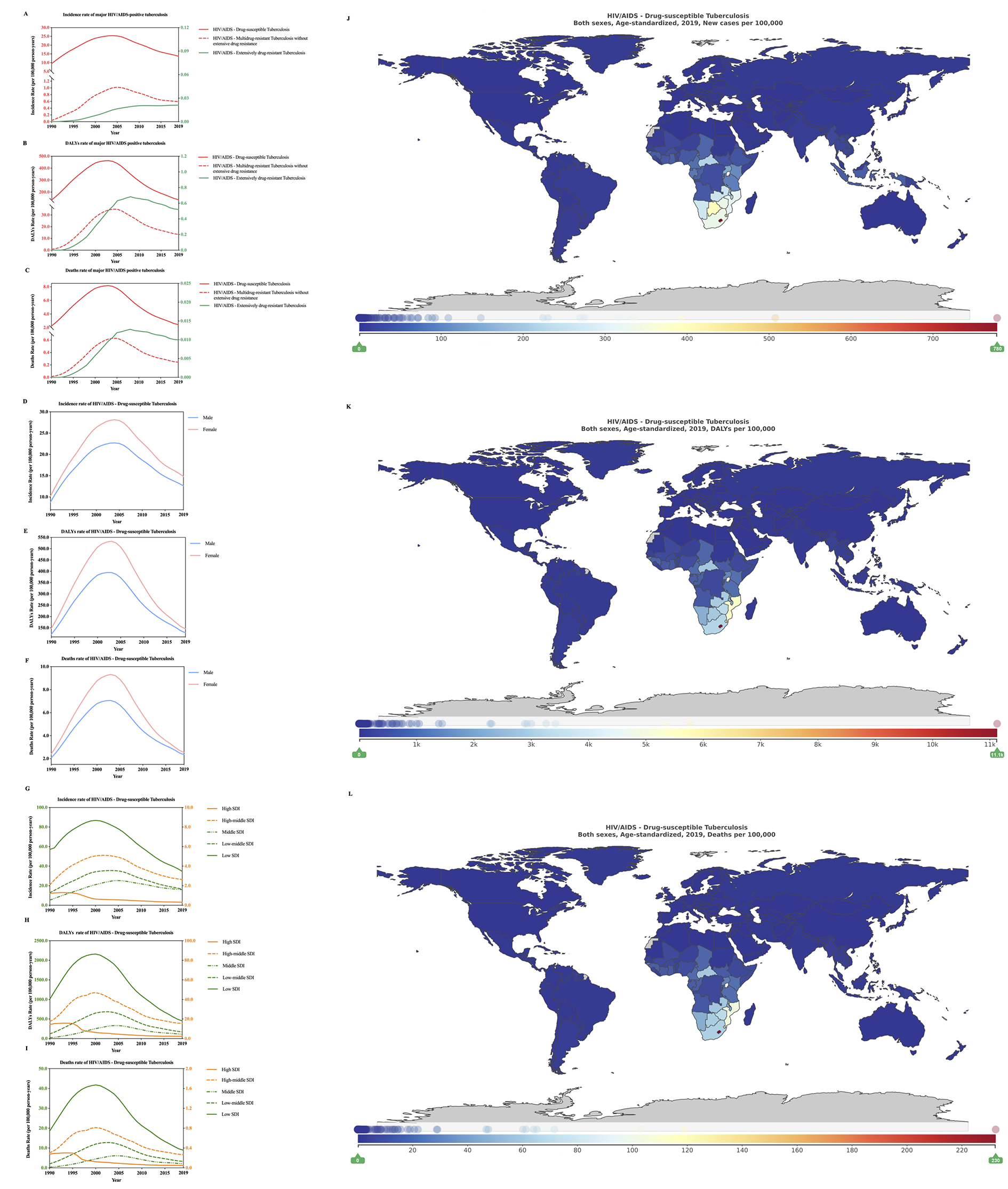

Supplement: Supplementary file 1 [file Image_1.tif]

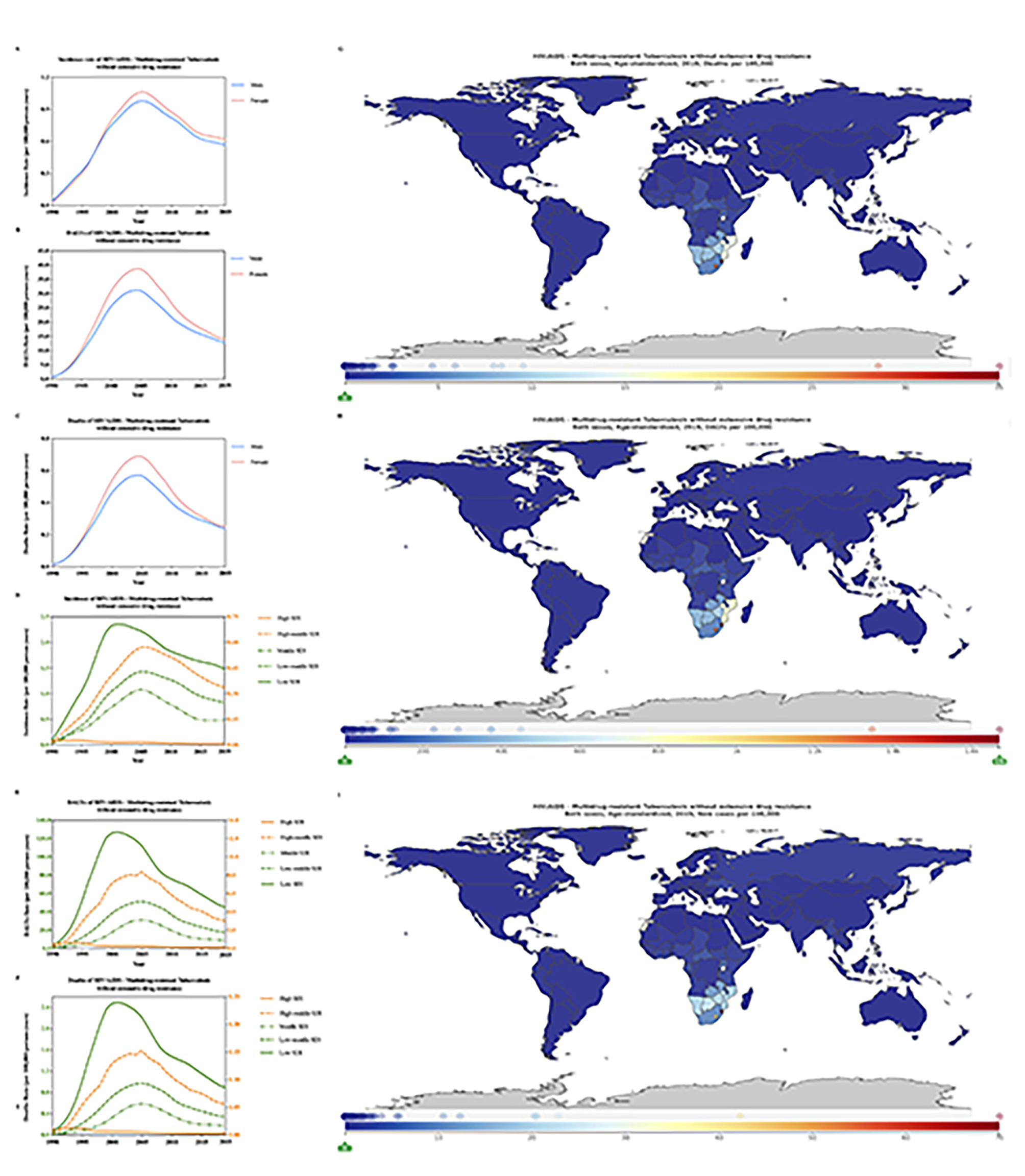

Supplement: Supplementary file 2 [file Image_2.tif]

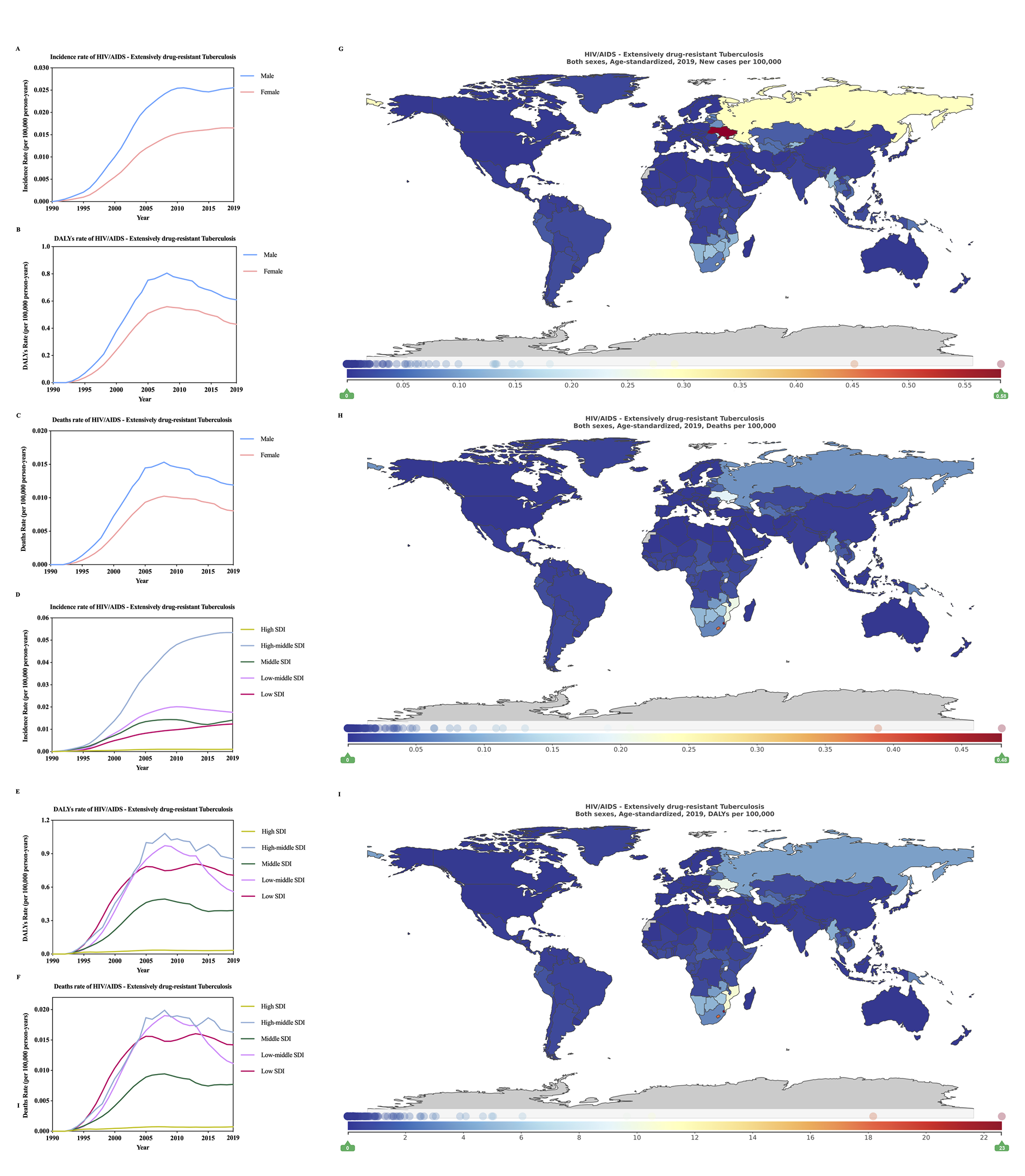

Supplement: Supplementary file 3 [file Image_3.tif]
