# Supplementary material for: Burden of tuberculosis and its association with socio-economic development status in 204 countries and territories, 1990–2019
Source: Front Med (Lausanne). 2022 Jul 22;9:905245. doi: 10.3389/fmed.2022.905245 (PMC9355511; doi:10.3389/fmed.2022.905245)
Supplement: Supplementary file 4 [file Table_1.docx]

| **S1** | | | | | | | | | | | | | | | | | |  |
| --- | --- | --- | --- | --- | --- | --- | --- | --- | --- | --- | --- | --- | --- | --- | --- | --- | --- | --- |
| **Year** | **Incidence** | | | | | |  | **DALYs (Disability-Adjusted Life Years)** | | | |  | **Deaths** | | | | | |
|  | **Drug-susceptible tuberculosis** | **Extensively drug-resistant tuberculosis** | **Multidrug-resistant tuberculosis without extensive drug resistance** | | | **Tuberculosis** |  | **Drug-susceptible tuberculosis** | **Extensively drug-resistant tuberculosis** | **Multidrug-resistant tuberculosis without extensive drug resistance** | **Tuberculosis** |  | **Drug-susceptible tuberculosis** | **Extensively drug-resistant tuberculosis** | **Multidrug-resistant tuberculosis without extensive drug resistance** | | | **Tuberculosis** |
| **1990** | 17150.65(15041.67 to 19655.72) | 0.00(0.00 to 0.00) | | 104.79(46.86 to 236.27) | 17255.45(15095.61 to 19779.28) | |  | 157118.41(143898.54 to 169833.73) | 0.00(0.00 to 0.00) | 1392.75(487.73 to 3251.00) | 158511.17(145422.48 to 171073.59) |  | 3969.86(3616.53 to 4305.31) | 0.00(0.00 to 0.00) | | 38.68(13.53 to 91.33) | 4008.53(3656.92 to 4334.12) | |
| **1991** | 16566.15(14524.24 to 18988.17) | 0.51(0.13 to 1.95) | | 186.02(101.30 to 339.57) | 16752.68(14674.25 to 19202.14) | |  | 154022.64(141538.54 to 167100.23) | 0.24(0.05 to 0.99) | 2121.28(854.02 to 4570.28) | 156144.16(143809.92 to 169206.54) |  | 3893.70(3540.34 to 4246.11) | 0.00(0.00 to 0.00) | | 58.47(22.43 to 126.81) | 3952.17(3622.90 to 4300.67) | |
| **1992** | 16019.81(14058.61 to 18362.52) | 1.19(0.50 to 3.57) | | 263.97(161.34 to 426.55) | 16284.97(14282.46 to 18655.42) | |  | 151771.03(139022.53 to 165093.69) | 0.56(0.20 to 1.77) | 2969.86(1295.27 to 6023.90) | 154741.45(142086.35 to 167181.95) |  | 3842.24(3506.67 to 4183.02) | 0.00(0.00 to 0.00) | | 81.61(34.67 to 167.12) | 3923.85(3575.83 to 4264.16) | |
| **1993** | 15542.73(13646.12 to 17825.61) | 2.00(1.12 to 4.57) | | 334.67(224.58 to 509.89) | 15879.41(13937.74 to 18182.31) | |  | 145337.67(133930.35 to 156834.51) | 20.04(8.80 to 38.59) | 3854.04(1827.52 to 7341.97) | 149211.75(137720.23 to 160997.09) |  | 3676.87(3365.40 to 3994.14) | 0.57(0.24 to 1.11) | | 105.28(47.30 to 202.64) | 3782.71(3479.30 to 4095.61) | |
| **1994** | 15164.30(13319.04 to 17389.07) | 2.90(2.00 to 4.76) | | 394.09(276.48 to 582.20) | 15561.29(13663.09 to 17812.54) | |  | 140144.38(128850.78 to 151254.62) | 48.35(22.16 to 89.51) | 4854.80(2305.07 to 9214.17) | 145047.53(133828.17 to 155766.13) |  | 3535.15(3240.30 to 3833.01) | 1.37(0.62 to 2.60) | | 131.31(61.08 to 252.63) | 3667.84(3364.84 to 3950.81) | |
| **1995** | 14911.74(13106.81 to 17103.80) | 3.87(2.92 to 5.19) | | 438.07(311.25 to 659.52) | 15353.68(13480.06 to 17579.25) | |  | 135595.96(124510.33 to 146704.33) | 82.66(39.23 to 152.86) | 5803.13(2785.14 to 11082.82) | 141481.75(130634.50 to 152260.63) |  | 3410.59(3107.12 to 3694.85) | 2.34(1.10 to 4.38) | | 155.68(72.55 to 300.16) | 3568.61(3297.60 to 3837.66) | |
| **1996** | 14742.17(12960.37 to 16877.64) | 5.21(4.04 to 6.86) | | 477.40(349.84 to 719.48) | 15224.79(13371.16 to 17417.38) | |  | 130860.50(119178.34 to 141726.10) | 118.20(55.51 to 213.89) | 6561.92(3129.57 to 12809.79) | 137540.62(127441.88 to 147799.10) |  | 3287.75(2987.32 to 3564.14) | 3.32(1.54 to 6.08) | | 175.09(80.75 to 346.29) | 3466.15(3221.50 to 3732.07) | |
| **1997** | 14584.29(12827.95 to 16691.83) | 7.11(5.58 to 9.35) | | 522.11(383.77 to 768.63) | 15113.51(13256.06 to 17285.93) | |  | 127888.87(116039.50 to 138563.66) | 152.16(70.66 to 281.30) | 7100.44(3323.46 to 13869.16) | 135141.47(124739.10 to 145071.77) |  | 3216.57(2916.33 to 3488.84) | 4.25(1.96 to 7.91) | | 188.74(85.72 to 366.20) | 3409.57(3173.96 to 3639.19) | |
| **1998** | 14437.79(12672.05 to 16513.31) | 9.29(7.26 to 12.06) | | 567.29(415.24 to 824.43) | 15014.37(13164.14 to 17161.37) | |  | 124620.48(112992.76 to 134874.58) | 186.82(88.20 to 345.09) | 7630.81(3554.67 to 14623.54) | 132438.10(122881.33 to 142311.74) |  | 3133.14(2851.77 to 3394.47) | 5.19(2.41 to 9.63) | | 201.93(91.71 to 390.57) | 3340.27(3103.76 to 3565.23) | |
| **1999** | 14301.48(12561.99 to 16362.59) | 11.49(8.96 to 14.82) | | 607.82(435.08 to 902.29) | 14920.79(13075.24 to 17044.63) | |  | 121481.92(109956.56 to 131691.99) | 229.26(109.56 to 418.96) | 8205.78(3787.13 to 15055.72) | 129916.96(120549.68 to 139075.42) |  | 3038.97(2749.86 to 3282.07) | 6.33(2.94 to 11.77) | | 216.03(97.49 to 399.36) | 3261.32(3032.10 to 3468.49) | |
| **2000** | 14171.72(12426.50 to 16206.13) | 13.46(10.31 to 17.48) | | 638.22(450.50 to 970.14) | 14823.39(13008.79 to 16929.16) | |  | 118330.25(107155.47 to 127966.10) | 271.94(130.52 to 492.78) | 8567.12(3967.28 to 14985.30) | 127169.31(118302.30 to 136114.04) |  | 2954.98(2673.83 to 3192.38) | 7.47(3.54 to 13.68) | | 225.32(101.98 to 395.50) | 3187.77(2977.91 to 3396.17) | |
| **2001** | 14003.74(12284.94 to 16012.10) | 15.53(12.24 to 19.71) | | 662.35(486.32 to 955.30) | 14681.61(12875.53 to 16754.92) | |  | 113370.44(103054.52 to 123177.94) | 307.98(149.66 to 550.66) | 8718.13(4182.94 to 14942.70) | 122396.55(113712.75 to 130616.43) |  | 2827.96(2558.10 to 3055.92) | 8.44(4.08 to 15.45) | | 229.50(106.33 to 399.17) | 3065.90(2868.99 to 3251.79) | |
| **2002** | 13771.23(12057.92 to 15766.99) | 18.00(14.29 to 22.34) | | 685.24(518.56 to 946.87) | 14474.47(12691.57 to 16517.71) | |  | 108411.80(98268.51 to 117568.01) | 343.13(173.24 to 599.58) | 8750.80(4218.95 to 14929.63) | 117505.73(109469.79 to 125042.21) |  | 2705.58(2452.78 to 2928.81) | 9.41(4.67 to 16.89) | | 230.81(108.76 to 401.74) | 2945.80(2759.84 to 3126.41) | |
| **2003** | 13511.09(11833.60 to 15455.19) | 20.53(16.24 to 25.45) | | 704.01(548.43 to 935.51) | 14235.64(12482.18 to 16251.08) | |  | 103574.83(94147.61 to 112959.11) | 375.74(193.16 to 660.71) | 8673.45(4238.64 to 14739.55) | 112624.01(104848.02 to 120534.65) |  | 2582.34(2346.45 to 2789.07) | 10.26(5.17 to 18.37) | | 228.41(109.99 to 391.13) | 2821.01(2643.66 to 2996.49) | |
| **2004** | 13256.80(11620.37 to 15142.74) | 22.81(18.03 to 28.88) | | 715.77(565.78 to 924.37) | 13995.38(12264.35 to 15982.59) | |  | 98433.66(89443.04 to 107239.67) | 397.44(207.90 to 690.58) | 8420.45(4265.28 to 14235.80) | 107251.55(100170.12 to 114536.18) |  | 2437.27(2224.50 to 2639.18) | 10.75(5.47 to 19.02) | | 219.63(109.86 to 376.39) | 2667.65(2506.18 to 2832.67) | |
| **2005** | 13040.94(11400.56 to 14889.86) | 24.53(19.16 to 31.09) | | 717.29(569.30 to 926.30) | 13782.76(12062.96 to 15749.19) | |  | 94678.91(86588.55 to 103312.47) | 427.89(224.46 to 727.05) | 8305.30(4323.31 to 13975.91) | 103412.10(96409.79 to 110597.80) |  | 2340.79(2136.14 to 2536.75) | 11.45(5.89 to 19.84) | | 215.57(109.07 to 366.52) | 2567.81(2414.33 to 2721.76) | |
| **2006** | 12816.07(11225.93 to 14642.67) | 25.83(20.34 to 32.56) | | 707.65(572.45 to 889.15) | 13549.56(11863.67 to 15487.94) | |  | 90771.50(82791.57 to 98956.67) | 426.29(225.78 to 720.65) | 7995.03(4175.05 to 13321.14) | 99192.82(92522.55 to 105770.01) |  | 2240.74(2042.91 to 2430.59) | 11.36(5.88 to 19.45) | | 206.86(104.71 to 355.39) | 2458.96(2318.75 to 2609.68) | |
| **2007** | 12530.39(10961.58 to 14315.41) | 26.98(21.27 to 34.07) | | 690.21(552.69 to 865.37) | 13247.58(11596.69 to 15135.44) | |  | 87079.52(79365.87 to 94925.35) | 428.71(226.71 to 720.98) | 7676.45(4006.39 to 13067.68) | 95184.68(88823.76 to 101896.98) |  | 2147.77(1963.85 to 2318.13) | 11.41(5.90 to 19.58) | | 198.40(100.23 to 342.23) | 2357.58(2221.38 to 2509.34) | |
| **2008** | 12222.33(10712.70 to 13969.15) | 27.91(21.74 to 35.86) | | 669.20(524.84 to 866.12) | 12919.43(11302.30 to 14765.87) | |  | 83877.08(76557.45 to 91370.61) | 432.24(224.64 to 735.50) | 7413.75(3814.98 to 12373.64) | 91723.07(85086.96 to 98142.31) |  | 2067.76(1884.32 to 2241.73) | 11.48(5.83 to 19.94) | | 191.23(94.79 to 325.56) | 2270.47(2135.57 to 2416.65) | |
| **2009** | 11930.24(10473.18 to 13640.44) | 28.56(21.69 to 37.43) | | 648.80(482.25 to 871.14) | 12607.60(11024.35 to 14398.83) | |  | 79900.46(72629.43 to 87171.13) | 417.84(217.35 to 715.90) | 7060.82(3536.33 to 11848.81) | 87379.11(81507.29 to 93382.05) |  | 1964.34(1786.32 to 2129.78) | 11.11(5.60 to 19.28) | | 181.40(88.57 to 311.64) | 2156.85(2033.55 to 2289.78) | |
| **2010** | 11690.02(10278.85 to 13369.55) | 28.85(21.28 to 38.32) | | 632.99(444.68 to 897.63) | 12351.87(10803.70 to 14097.70) | |  | 76047.61(68693.84 to 82648.41) | 415.64(215.28 to 707.47) | 6775.33(3293.11 to 11638.20) | 83238.58(77361.15 to 88880.71) |  | 1865.89(1696.40 to 2020.78) | 11.04(5.51 to 19.21) | | 173.40(82.21 to 305.40) | 2050.33(1933.45 to 2179.55) | |
| **2011** | 11457.85(10087.65 to 13087.51) | 28.73(21.25 to 38.49) | | 616.57(433.36 to 892.36) | 12103.15(10593.78 to 13794.14) | |  | 72579.58(65375.00 to 79238.80) | 408.58(208.82 to 698.11) | 6523.79(3042.79 to 11476.06) | 79511.95(73885.57 to 85555.85) |  | 1780.16(1609.77 to 1934.99) | 10.89(5.37 to 18.98) | | 167.14(75.58 to 300.84) | 1958.18(1842.72 to 2084.33) | |
| **2012** | 11185.74(9853.78 to 12755.90) | 28.31(21.03 to 38.22) | | 594.63(409.92 to 879.47) | 11808.69(10350.38 to 13431.69) | |  | 70097.76(62791.26 to 76847.04) | 407.05(206.25 to 689.97) | 6368.36(2912.91 to 11443.58) | 76873.18(71647.21 to 82407.78) |  | 1721.22(1547.68 to 1872.18) | 10.91(5.34 to 18.78) | | 163.81(71.43 to 301.19) | 1895.94(1784.87 to 2011.25) | |
| **2013** | 10910.24(9597.69 to 12414.01) | 27.84(20.70 to 37.74) | | 572.20(384.66 to 882.56) | 11510.28(10118.88 to 13077.99) | |  | 67781.43(60458.14 to 74528.76) | 401.39(201.60 to 687.87) | 6228.76(2806.48 to 11429.25) | 74411.58(69265.03 to 80043.36) |  | 1670.21(1483.22 to 1823.10) | 10.78(5.18 to 19.05) | | 160.94(68.52 to 304.65) | 1841.93(1736.20 to 1965.30) | |
| **2014** | 10669.48(9389.37 to 12111.39) | 27.53(20.17 to 38.06) | | 554.20(354.76 to 900.78) | 11251.21(9924.31 to 12797.46) | |  | 65561.66(58029.53 to 72039.12) | 391.43(199.69 to 679.23) | 6004.25(2646.16 to 10945.76) | 71957.33(66795.86 to 77329.50) |  | 1620.54(1438.50 to 1761.83) | 10.54(5.11 to 18.67) | | 155.73(64.81 to 287.86) | 1786.81(1681.24 to 1901.44) | |
| **2015** | 10499.62(9215.75 to 11938.78) | 27.58(19.90 to 39.02) | | 545.53(330.96 to 939.78) | 11072.73(9765.15 to 12609.37) | |  | 62849.10(55340.66 to 69479.02) | 389.69(198.04 to 676.60) | 5821.58(2577.65 to 10476.76) | 69060.37(63804.53 to 74604.55) |  | 1550.83(1373.86 to 1704.11) | 10.51(5.11 to 18.68) | | 150.83(62.97 to 277.05) | 1712.17(1599.97 to 1833.48) | |
| **2016** | 10368.97(9086.40 to 11750.11) | 28.32(20.40 to 40.40) | | 548.18(325.91 to 950.72) | 10945.47(9667.65 to 12420.92) | |  | 60443.58(53132.09 to 66756.29) | 385.83(194.60 to 675.87) | 5688.32(2522.84 to 10507.39) | 66517.73(61105.25 to 71885.74) |  | 1493.45(1317.92 to 1648.03) | 10.48(5.07 to 18.91) | | 147.75(60.60 to 280.24) | 1651.68(1531.01 to 1772.44) | |
| **2017** | 10279.77(9008.30 to 11657.04) | 29.14(20.77 to 42.00) | | 553.51(322.09 to 974.49) | 10862.42(9616.60 to 12305.40) | |  | 58114.28(51044.87 to 64907.90) | 381.36(190.33 to 673.09) | 5550.13(2461.45 to 10230.89) | 64045.77(58194.26 to 69798.19) |  | 1438.38(1270.46 to 1599.52) | 10.42(4.95 to 18.68) | | 144.61(59.44 to 274.95) | 1593.40(1464.11 to 1727.46) | |
| **2018** | 10198.93(8897.53 to 11558.73) | 29.93(21.06 to 43.01) | | 557.64(318.33 to 965.18) | 10786.49(9522.07 to 12275.51) | |  | 55713.85(48729.19 to 62506.69) | 382.24(186.99 to 680.39) | 5407.83(2327.95 to 9887.68) | 61503.91(56061.87 to 67606.39) |  | 1375.83(1208.89 to 1539.38) | 10.44(4.94 to 19.05) | | 140.65(55.18 to 267.67) | 1526.93(1399.98 to 1666.65) | |
| **2019** | 10077.42(8738.07 to 11455.75) | 31.02(21.12 to 45.11) | | 563.01(312.11 to 973.10) | 10671.45(9395.60 to 12194.10) | |  | 53422.87(46676.27 to 59669.58) | 381.51(189.14 to 668.32) | 5238.08(2263.60 to 9759.93) | 59042.45(53684.78 to 64641.53) |  | 1317.30(1147.33 to 1473.41) | 10.42(4.90 to 18.73) | | 135.91(54.01 to 258.62) | 1463.62(1339.24 to 1602.71) | |

| **S2** | | | | | | | | | | | | | | | | | | | | | | |
| --- | --- | --- | --- | --- | --- | --- | --- | --- | --- | --- | --- | --- | --- | --- | --- | --- | --- | --- | --- | --- | --- | --- |
| **Group** | **Incidence** | | | | |  | **DALYs (Disability-Adjusted Life Years)** | | | | | |  | | **Deaths** | | | | | | |  |
|  | **HIV/AIDS - Drug-susceptible Tuberculosis** | **HIV/AIDS - Extensively drug-resistant Tuberculosis** | | **HIV/AIDS - Multidrug-resistant Tuberculosis without extensive drug resistance** | |  | **HIV/AIDS - Drug-susceptible Tuberculosis** | **HIV/AIDS - Extensively drug-resistant Tuberculosis** | | **HIV/AIDS - Multidrug-resistant Tuberculosis without extensive drug resistance** | |  | | **HIV/AIDS - Drug-susceptible Tuberculosis** | | | **HIV/AIDS - Extensively drug-resistant Tuberculosis** | | **HIV/AIDS - Multidrug-resistant Tuberculosis without extensive drug resistance** | |  |  |
| **1990** | 948.42(840.37 to 1069.53) | | 0.00(0.00 to 0.00) | | 3.54(2.21 to 5.81) |  | 13471.43(10373.61 to 17433.19) | | 0.00(0.00 to 0.00) | | 73.06(28.66 to 150.03) | |  | | | 223.45(166.37 to 297.21) | | 0.00(0.00 to 0.00) | | 1.28(0.48 to 2.67) | |  |
| **1991** | 1139.72(1007.01 to 1285.56) | | 0.02(0.00 to 0.05) | | 8.60(5.81 to 13.58) |  | 16558.45(12735.25 to 21337.86) | | 0.01(0.00 to 0.03) | | 142.25(57.39 to 300.76) | |  | | | 275.78(205.29 to 365.84) | | 0.00(0.00 to 0.00) | | 2.45(0.94 to 5.19) | |  |
| **1992** | 1322.57(1166.71 to 1492.90) | | 0.04(0.02 to 0.11) | | 14.19(9.84 to 21.67) |  | 19960.87(15335.76 to 25762.75) | | 0.02(0.01 to 0.07) | | 257.95(105.92 to 551.86) | |  | | | 334.37(250.10 to 441.43) | | 0.00(0.00 to 0.00) | | 4.44(1.77 to 9.55) | |  |
| **1993** | 1492.69(1311.44 to 1687.14) | | 0.07(0.04 to 0.16) | | 20.06(14.18 to 29.70) |  | 23591.75(18176.81 to 30482.24) | | 0.95(0.41 to 1.87) | | 434.91(181.05 to 888.36) | |  | | | 397.76(299.82 to 521.37) | | 0.02(0.01 to 0.04) | | 7.53(3.05 to 15.56) | |  |
| **1994** | 1648.36(1449.82 to 1868.33) | | 0.11(0.07 to 0.20) | | 25.92(18.52 to 38.68) |  | 27263.73(20917.57 to 35098.23) | | 2.73(1.15 to 5.38) | | 681.69(282.54 to 1402.59) | |  | | | 462.63(347.97 to 605.36) | | 0.05(0.02 to 0.10) | | 11.87(4.77 to 24.78) | |  |
| **1995** | 1784.58(1558.44 to 2027.84) | | 0.16(0.11 to 0.25) | | 31.45(22.20 to 46.60) |  | 30793.42(23492.04 to 39804.26) | | 5.39(2.23 to 10.22) | | 991.99(411.44 to 2045.90) | |  | | | 525.65(394.66 to 690.66) | | 0.10(0.04 to 0.19) | | 17.35(7.06 to 36.33) | |  |
| **1996** | 1917.66(1672.06 to 2181.24) | | 0.23(0.16 to 0.36) | | 38.70(27.49 to 56.00) |  | 33919.93(25529.04 to 43872.84) | | 8.76(3.71 to 16.67) | | 1340.06(566.12 to 2698.74) | |  | | | 582.20(432.47 to 764.34) | | 0.17(0.07 to 0.31) | | 23.50(9.65 to 47.52) | |  |
| **1997** | 2057.56(1790.20 to 2343.00) | | 0.35(0.25 to 0.52) | | 48.77(35.14 to 69.63) |  | 36917.44(27723.69 to 47739.20) | | 12.61(5.32 to 23.83) | | 1687.32(716.01 to 3289.46) | |  | | | 636.67(468.57 to 839.47) | | 0.24(0.10 to 0.45) | | 29.61(12.09 to 58.14) | |  |
| **1998** | 2191.14(1905.25 to 2500.46) | | 0.49(0.35 to 0.73) | | 59.90(43.32 to 84.73) |  | 39869.98(29864.46 to 51867.68) | | 17.18(7.29 to 32.41) | | 2085.55(899.84 to 4026.70) | |  | | | 690.40(507.02 to 908.93) | | 0.32(0.13 to 0.60) | | 36.63(15.39 to 71.94) | |  |
| **1999** | 2306.95(2006.26 to 2629.93) | | 0.64(0.45 to 0.94) | | 70.30(50.42 to 101.35) |  | 42367.07(31391.23 to 55021.52) | | 23.93(10.14 to 45.29) | | 2492.58(1089.83 to 4738.63) | |  | | | 736.78(534.61 to 970.08) | | 0.45(0.19 to 0.85) | | 43.93(18.62 to 84.30) | |  |
| **2000** | 2391.72(2081.61 to 2732.69) | | 0.78(0.54 to 1.16) | | 78.06(55.26 to 115.62) |  | 44479.83(32769.89 to 57672.84) | | 30.88(13.20 to 58.10) | | 2831.21(1216.84 to 5315.21) | |  | | | 777.19(558.90 to 1017.74) | | 0.58(0.24 to 1.09) | | 50.15(21.02 to 94.41) | |  |
| **2001** | 2451.84(2130.56 to 2803.30) | | 0.93(0.67 to 1.34) | | 84.26(59.81 to 121.04) |  | 45573.16(33068.29 to 59226.41) | | 37.39(16.04 to 70.05) | | 3091.14(1325.82 to 5754.30) | |  | | | 799.43(568.06 to 1044.74) | | 0.70(0.30 to 1.32) | | 55.04(23.23 to 103.28) | |  |
| **2002** | 2499.66(2166.54 to 2859.14) | | 1.12(0.82 to 1.56) | | 90.65(65.64 to 124.98) |  | 46177.29(32788.78 to 59777.23) | | 44.25(19.12 to 83.20) | | 3294.54(1426.42 to 6113.62) | |  | | | 812.88(569.39 to 1059.97) | | 0.83(0.35 to 1.57) | | 58.94(24.84 to 110.35) | |  |
| **2003** | 2530.43(2189.74 to 2896.35) | | 1.33(0.97 to 1.84) | | 96.35(71.12 to 131.41) |  | 46341.92(32665.70 to 60132.89) | | 50.99(22.06 to 96.61) | | 3431.25(1504.54 to 6454.99) | |  | | | 818.61(566.84 to 1065.10) | | 0.96(0.41 to 1.83) | | 61.67(26.53 to 116.79) | |  |
| **2004** | 2540.38(2201.09 to 2908.52) | | 1.51(1.11 to 2.09) | | 100.49(74.61 to 136.65) |  | 45634.11(31899.89 to 58767.48) | | 56.27(24.75 to 105.04) | | 3494.63(1538.79 to 6551.15) | |  | | | 807.56(552.60 to 1044.96) | | 1.06(0.46 to 2.00) | | 63.00(27.32 to 119.30) | |  |
| **2005** | 2526.03(2188.61 to 2889.43) | | 1.64(1.20 to 2.29) | | 102.20(75.09 to 141.21) |  | 43941.97(30644.01 to 56228.48) | | 63.25(27.96 to 118.12) | | 3487.51(1537.07 to 6593.87) | |  | | | 777.92(530.60 to 1002.22) | | 1.19(0.52 to 2.24) | | 62.98(27.07 to 119.20) | |  |
| **2006** | 2473.33(2143.84 to 2832.27) | | 1.74(1.28 to 2.39) | | 100.97(75.77 to 136.31) |  | 41410.94(29120.82 to 52905.46) | | 64.70(28.74 to 122.87) | | 3344.52(1498.17 to 6245.12) | |  | | | 733.13(503.19 to 941.56) | | 1.21(0.53 to 2.32) | | 60.51(26.60 to 114.97) | |  |
| **2007** | 2382.72(2068.73 to 2725.06) | | 1.84(1.36 to 2.51) | | 97.54(74.30 to 129.99) |  | 38271.17(27029.13 to 48753.97) | | 66.61(29.51 to 124.28) | | 3128.77(1399.51 to 5819.10) | |  | | | 676.18(467.88 to 868.34) | | 1.25(0.55 to 2.35) | | 56.61(24.79 to 106.02) | |  |
| **2008** | 2272.47(1974.86 to 2596.54) | | 1.92(1.42 to 2.59) | | 92.98(71.19 to 122.33) |  | 35054.88(25230.34 to 44639.36) | | 68.33(29.97 to 127.91) | | 2909.58(1319.13 to 5306.87) | |  | | | 617.09(431.35 to 786.79) | | 1.28(0.56 to 2.40) | | 52.52(23.23 to 96.62) | |  |
| **2009** | 2161.89(1881.46 to 2467.48) | | 1.99(1.45 to 2.67) | | 88.35(66.85 to 117.44) |  | 31935.51(23136.88 to 40669.71) | | 66.71(29.33 to 123.62) | | 2694.93(1234.82 to 4925.55) | |  | | | 560.79(395.16 to 717.12) | | 1.25(0.54 to 2.32) | | 48.52(21.84 to 89.28) | |  |
| **2010** | 2069.89(1804.33 to 2360.14) | | 2.04(1.48 to 2.75) | | 84.69(63.51 to 113.77) |  | 29163.32(21061.71 to 37237.57) | | 65.97(29.29 to 121.01) | | 2443.43(1136.01 to 4466.81) | |  | | | 511.74(358.73 to 655.32) | | 1.23(0.54 to 2.27) | | 43.85(19.89 to 80.76) | |  |
| **2011** | 1983.29(1731.80 to 2260.65) | | 2.05(1.51 to 2.75) | | 81.04(61.64 to 107.56) |  | 26529.17(19233.07 to 33886.84) | | 64.89(28.81 to 119.28) | | 2220.60(1041.45 to 4027.89) | |  | | | 465.74(325.52 to 599.38) | | 1.22(0.53 to 2.27) | | 39.82(18.20 to 73.19) | |  |
| **2012** | 1883.34(1646.22 to 2146.52) | | 2.05(1.54 to 2.73) | | 76.33(58.52 to 100.29) |  | 24189.89(17571.45 to 30867.47) | | 64.23(28.64 to 117.74) | | 2048.41(968.67 to 3678.91) | |  | | | 424.96(298.72 to 546.23) | | 1.21(0.53 to 2.21) | | 36.77(17.13 to 66.80) | |  |
| **2013** | 1780.36(1557.86 to 2028.03) | | 2.04(1.56 to 2.71) | | 71.40(54.89 to 93.45) |  | 22141.97(16168.35 to 28217.77) | | 61.77(27.40 to 113.30) | | 1907.87(912.76 to 3422.23) | |  | | | 389.34(276.07 to 500.09) | | 1.16(0.51 to 2.13) | | 34.28(16.11 to 62.26) | |  |
| **2014** | 1685.43(1476.66 to 1920.19) | | 2.04(1.54 to 2.74) | | 67.12(51.38 to 89.34) |  | 20444.57(15047.52 to 25999.29) | | 59.97(26.31 to 110.20) | | 1781.45(828.49 to 3197.59) | |  | | | 360.26(258.02 to 462.86) | | 1.13(0.49 to 2.08) | | 32.01(14.36 to 58.17) | |  |
| **2015** | 1609.69(1410.76 to 1830.47) | | 2.04(1.53 to 2.76) | | 64.34(48.63 to 87.84) |  | 18941.04(13947.00 to 24191.74) | | 58.72(25.79 to 108.15) | | 1683.89(766.73 to 3085.49) | |  | | | 335.02(240.13 to 432.34) | | 1.12(0.48 to 2.05) | | 30.33(13.49 to 56.19) | |  |
| **2016** | 1548.79(1364.37 to 1760.62) | | 2.06(1.55 to 2.75) | | 62.87(47.05 to 85.42) |  | 17610.78(12928.80 to 22541.75) | | 57.08(24.83 to 103.93) | | 1592.86(719.18 to 2938.31) | |  | | | 312.78(223.32 to 406.62) | | 1.09(0.46 to 2.00) | | 28.76(12.71 to 53.44) | |  |
| **2017** | 1494.34(1318.70 to 1700.67) | | 2.09(1.56 to 2.79) | | 61.77(44.98 to 85.01) |  | 16141.86(11851.69 to 20702.53) | | 54.30(23.85 to 98.41) | | 1494.63(664.38 to 2739.23) | |  | | | 287.42(205.32 to 374.18) | | 1.04(0.44 to 1.90) | | 27.04(11.67 to 50.69) | |  |
| **2018** | 1436.83(1263.51 to 1639.42) | | 2.10(1.54 to 2.86) | | 60.58(43.67 to 84.26) |  | 14638.25(10776.29 to 18831.35) | | 52.76(23.27 to 95.44) | | 1395.85(611.15 to 2520.72) | |  | | | 261.07(185.37 to 339.52) | | 1.01(0.43 to 1.86) | | 25.35(10.66 to 46.85) | |  |
| **2019** | 1370.31(1199.74 to 1564.78) | | 2.10(1.51 to 2.90) | | 59.23(41.55 to 83.61) |  | 13697.12(10071.81 to 17569.09) | | 52.00(22.94 to 93.85) | | 1341.46(582.21 to 2456.71) | |  | | | 244.92(173.25 to 319.00) | | 1.00(0.43 to 1.82) | | 24.45(10.23 to 45.37) | |  |

| **S3** | | | | | | | | | | | |
| --- | --- | --- | --- | --- | --- | --- | --- | --- | --- | --- | --- |
| **Group** | **Incidence** | | |  | **DALYs (Disability-Adjusted Life Years)** | | |  | **Deaths** | | |
|  | **Male** | **Female** | **Both** |  | **Male** | **Female** | **Both** |  | **Male** | **Female** | **Both** |
| **Global** | -34.87(-37.54 to -31.87) | -42.20(-44.66 to -39.44) | -38.16(-40.73 to -35.26) |  | -60.85(-65.26 to -56.49) | -65.95(-72.04 to -54.65) | -62.75(-66.61 to -57.98) |  | -63.15(-67.36 to -58.74) | -65.34(-72.17 to -52.00) | -63.49(-67.62 to -57.85) |
| **High SDI** | -52.59(-55.36 to -49.60) | -56.23(-59.04 to -53.27) | -53.47(-56.18 to -50.56) |  | -78.98(-80.96 to -76.88) | -74.45(-79.25 to -66.17) | -77.10(-79.39 to -74.19) |  | -77.25(-79.09 to -75.51) | -71.40(-76.26 to -63.43) | -74.92(-77.39 to -72.31) |
| **High-middle SDI** | -39.91(-42.48 to -36.78) | -49.52(-51.99 to -46.62) | -43.49(-45.95 to -40.62) |  | -71.19(-74.39 to -67.70) | -72.97(-76.54 to -64.86) | -71.38(-74.04 to -68.60) |  | -73.72(-76.71 to -70.18) | -74.56(-78.11 to -64.70) | -73.32(-75.91 to -70.45) |
| **Middle SDI** | -48.13(-49.94 to -46.14) | -54.07(-55.79 to -52.16) | -51.03(-52.79 to -49.19) |  | -69.72(-72.83 to -66.45) | -76.29(-79.39 to -71.41) | -72.46(-74.90 to -69.39) |  | -71.28(-74.44 to -67.87) | -76.49(-80.05 to -69.86) | -73.27(-75.77 to -69.64) |
| **Low-middle SDI** | -42.35(-45.41 to -38.93) | -48.90(-51.72 to -45.77) | -45.74(-48.52 to -42.62) |  | -64.14(-69.76 to -58.69) | -70.83(-78.44 to -60.45) | -67.21(-72.17 to -62.21) |  | -65.11(-71.15 to -59.24) | -69.96(-78.63 to -53.72) | -67.36(-72.56 to -61.12) |
| **Low SDI** | -40.05(-42.77 to -37.00) | -43.78(-46.39 to -40.81) | -41.99(-44.49 to -39.18) |  | -59.64(-65.22 to -52.96) | -64.04(-70.31 to -48.43) | -61.55(-66.24 to -54.90) |  | -59.06(-64.65 to -52.34) | -61.00(-69.10 to -40.47) | -60.04(-64.85 to -52.69) |

| **S4** | | | | | | | | | | | | | | | |  |
| --- | --- | --- | --- | --- | --- | --- | --- | --- | --- | --- | --- | --- | --- | --- | --- | --- |
| **Group** | **Number of incident cases (age-standardized, both sexes) with 95% uncertainty intervals (2019)** | | | |  | **Annualized rate of change of** **age-standardized rate (%) with 95% uncertainty intervals** | | | | | | | | | | |
|  |  |  |  |  |  | **1990-2010** | | | |  | **2010-2019** | | | | | |
|  | **Drug-susceptible tuberculosis** | **Extensively drug-resistant tuberculosis** | **Multidrug-resistant tuberculosis without extensive drug resistance** | **Tuberculosis** |  | **Drug-susceptible tuberculosis** | **Extensively drug-resistant tuberculosis** | **Multidrug-resistant tuberculosis without extensive drug resistance** | **Tuberculosis** |  | **Drug-susceptible tuberculosis** | **Extensively drug-resistant tuberculosis** | | **Multidrug-resistant tuberculosis without extensive drug resistance** | | **Tuberculosis** |
| **Global** | 10077419(8738073 to 11455752) | 31016(21115 to 45110) | 563014(312112 to 973098) | 10671449(9395605 to 12194100) |  | -31.8(-34.3 to -29.3) | - | 504.0(156.8 to 1421.3) | -28.4(-30.7 to -26.0) |  | -13.8(-17.3 to -11.0) | | 7.5(-23.0 to 51.3) | | -11.1(-46.6 to 47.6) | -13.6(-15.4 to -11.7) |
| **Afghanistan** | 8121219(6798738 to 9528128) | 13206(2274 to 43776) | 374180(64406 to 1240323) | 8508605(7369346 to 9846194) |  | -42.4(-46.4 to -38.3) | - | 2964.1(624.7 to 38506.1) | -38.6(-42.4 to -34.5) |  | -17.4(-27.0 to -8.8) | | -11.6(-83.1 to 209.0) | | -41.7(-88.9 to 103.7) | -18.8(-25.0 to -12.2) |
| **Albania** | 289709(249387 to 337632) | 486(96 to 1386) | 2217(440 to 6325) | 292412(252025 to 340301) |  | -51.0(-55.7 to -45.8) | - | 264.8(-20.2 to 3350.0) | -50.3(-54.9 to -45.0) |  | -24.0(-29.3 to -18.3) | | -37.9(-87.7 to 107.9) | | -61.4(-92.4 to 29.1) | -24.6(-29.9 to -19.0) |
| **Algeria** | 1687076(1435192 to 1969102) | 1235(111 to 5801) | 34978(3146 to 164423) | 1723288(1491861 to 2005256) |  | -57.8(-61.0 to -54.5) | - | -42.9(-93.0 to 210.8) | -57.6(-60.5 to -54.6) |  | -15.5(-22.2 to -9.2) | | 66.5(-69.6 to 484.0) | | 9.7(-80.0 to 284.6) | -15.1(-20.3 to -9.6) |
| **American Samoa** | 1383784(1200530 to 1603677) | 381(38 to 1642) | 4175(416 to 18015) | 1388340(1205190 to 1612450) |  | -14.7(-19.3 to -9.5) | - | 119.4(-84.6 to 3184.4) | -14.5(-19.1 to -9.5) |  | -11.6(-16.8 to -6.4) | | 54.1(-71.8 to 814.1) | | -0.3(-81.8 to 491.3) | -11.6(-16.6 to -6.4) |
| **Andorra** | 343330(285722 to 414336) | 107(12 to 426) | 854(95 to 3387) | 344291(286212 to 415572) |  | -36.3(-40.2 to -32.0) | - | 0.6(-90.8 to 915.9) | -36.2(-40.2 to -31.8) |  | -16.8(-21.1 to -12.0) | | -6.8(-85.6 to 451.6) | | -40.3(-90.8 to 253.1) | -16.9(-21.2 to -12.2) |
| **Angola** | 25790430(22103856 to 29424079) | 4918(633 to 17399) | 762370(98061 to 2696600) | 26557719(23459820 to 30060243) |  | -11.7(-18.2 to -6.3) | - | 792.2(-16.1 to 10235.9) | -9.5(-14.0 to -5.0) |  | -19.6(-26.3 to -12.9) | | 28.9(-73.3 to 498.7) | | -14.3(-82.2 to 298.3) | -19.4(-24.7 to -14.1) |
| **Antigua and Barbuda** | 1868398(1565380 to 2211654) | 1062(104 to 4821) | 13454(1316 to 61061) | 1882915(1573864 to 2234872) |  | -24.4(-30.8 to -17.3) | - | -48.4(-95.3 to 499.1) | -24.6(-30.7 to -18.0) |  | -27.3(-32.3 to -22.6) | | 51.8(-72.0 to 912.9) | | -1.4(-81.8 to 557.9) | -27.2(-31.9 to -22.5) |
| **Argentina** | 1188782(1009596 to 1392592) | 2365(334 to 8669) | 18811(2655 to 68985) | 1209958(1039582 to 1421390) |  | -47.5(-53.1 to -41.4) | - | 183.6(-43.8 to 2187.6) | -46.6(-51.8 to -40.5) |  | 4.8(-4.1 to 13.9) | | 28.0(-74.6 to 292.8) | | -18.1(-83.7 to 151.1) | 4.4(-4.0 to 13.0) |
| **Armenia** | 1899770(1327339 to 2380392) | 76086(15856 to 179447) | 347256(72394 to 818996) | 2323112(2002555 to 2716422) |  | -34.4(-44.1 to -25.3) | - | 16597.8(3244.2 to 188114.6) | -17.2(-25.0 to -8.6) |  | -38.1(-54.3 to -26.6) | | -21.3(-78.5 to 63.2) | | -51.1(-86.6 to 1.4) | -40.1(-44.9 to -34.8) |
| **Australia** | 505325(433851 to 598913) | 2052(840 to 4366) | 16325(6675 to 34735) | 523703(451469 to 615565) |  | -31.1(-36.5 to -25.1) | - | 211.2(-32.8 to 1802.1) | -30.0(-35.4 to -24.0) |  | -8.6(-16.1 to -0.7) | | 157.0(-29.0 to 863.2) | | 64.5(-54.6 to 516.1) | -7.1(-14.0 to 0.3) |
| **Austria** | 571782(480973 to 678623) | 2327(867 to 4982) | 18506(6900 to 39617) | 592615(504353 to 699534) |  | -48.4(-52.1 to -44.6) | - | 385.7(29.9 to 4408.9) | -46.2(-50.1 to -42.2) |  | -17.3(-23.8 to -10.7) | | -5.5(-65.6 to 118.2) | | -39.5(-78.0 to 39.6) | -18.2(-23.9 to -12.5) |
| **Azerbaijan** | 3615363(2642853 to 4537977) | 214428(66849 to 412619) | 978646(305326 to 1882874) | 4808437(4056287 to 5701591) |  | -27.6(-38.4 to -18.4) | - | 26113.7(5984.5 to 276656.3) | -10.8(-18.6 to -1.8) |  | -23.9(-44.8 to -2.7) | | 61.6(-60.6 to 355.8) | | 0.4(-75.5 to 183.0) | -17.9(-25.3 to -9.2) |
| **Bahamas** | 1166206(999273 to 1365808) | 1445(198 to 5466) | 18301(2512 to 69179) | 1185952(1020890 to 1380164) |  | -40.3(-44.5 to -35.4) | - | 640.1(29.3 to 7770.7) | -39.7(-43.8 to -34.6) |  | -14.5(-20.5 to -7.9) | | 93.4(-72.7 to 873.6) | | 25.6(-82.3 to 532.1) | -14.0(-19.4 to -7.8) |
| **Bahrain** | 1619796(1389723 to 1888762) | 1679(277 to 5050) | 47569(7853 to 143154) | 1669044(1428063 to 1948660) |  | -47.3(-51.8 to -42.5) | - | 1085.1(160.5 to 13516.6) | -45.9(-50.4 to -41.0) |  | -26.3(-32.1 to -20.7) | | 19.5(-82.0 to 364.2) | | -21.2(-88.2 to 205.8) | -26.2(-30.2 to -21.9) |
| **Bangladesh** | 13423277(11555175 to 15620959) | 12305(2954 to 30347) | 502063(120566 to 1238209) | 13937646(12020468 to 16137438) |  | -57.9(-62.3 to -53.2) | - | 2132.1(346.5 to 24035.6) | -56.7(-61.1 to -51.8) |  | -31.3(-37.7 to -24.7) | | 35.1(-66.4 to 263.4) | | -10.7(-77.8 to 140.2) | -30.7(-36.5 to -25.0) |
| **Barbados** | 624432(524795 to 735601) | 171(13 to 831) | 2160(164 to 10516) | 626762(528430 to 737319) |  | -31.9(-37.3 to -26.1) | - | -83.9(-98.8 to 215.5) | -32.4(-37.6 to -27.0) |  | -22.7(-27.5 to -17.3) | | 80.9(-78.7 to 1217.2) | | 17.5(-86.1 to 756.0) | -22.6(-27.5 to -17.4) |
| **Belarus** | 1296552(920557 to 1678995) | 190494(129967 to 264984) | 869407(593084 to 1209259) | 2356454(2002864 to 2785991) |  | -46.8(-53.2 to -39.3) | - | 2302.9(411.8 to 20835.2) | -15.8(-23.5 to -6.3) |  | -51.0(-63.1 to -38.6) | | 0.1(-31.7 to 40.6) | | -37.8(-57.5 to -12.6) | -44.3(-49.1 to -39.3) |
| **Belgium** | 721723(611604 to 854146) | 1659(531 to 4002) | 13198(4223 to 31816) | 736580(625623 to 870584) |  | -33.0(-38.2 to -27.4) | - | 64.8(-51.8 to 904.7) | -32.0(-37.2 to -26.2) |  | -10.1(-16.9 to -3.3) | | 13.3(-64.5 to 186.4) | | -27.5(-77.3 to 83.2) | -10.4(-16.7 to -3.9) |
| **Belize** | 2452648(2130502 to 2850996) | 975(116 to 4245) | 12341(1464 to 53773) | 2465964(2135357 to 2862541) |  | -23.9(-30.6 to -17.2) | - | -43.3(-94.1 to 548.6) | -23.9(-30.7 to -17.1) |  | -19.2(-24.9 to -12.8) | | 103.2(-63.0 to 1080.1) | | 32.0(-76.0 to 666.3) | -19.1(-24.6 to -12.5) |
| **Benin** | 14473191(12584472 to 16636334) | 1782(322 to 4939) | 276192(49966 to 765468) | 14751165(12825009 to 16990164) |  | -27.6(-33.8 to -21.0) | - | 540.6(50.3 to 5659.1) | -26.6(-33.0 to -20.0) |  | -19.0(-24.4 to -12.7) | | 55.3(-71.3 to 355.0) | | 3.3(-80.9 to 202.6) | -18.7(-23.7 to -12.9) |
| **Bermuda** | 1696514(1415223 to 2029851) | 710(40 to 4128) | 8989(501 to 52237) | 1706213(1423900 to 2033524) |  | -33.7(-39.3 to -27.3) | - | -67.2(-98.2 to 316.5) | -33.8(-39.4 to -27.5) |  | -29.1(-34.1 to -23.8) | | 151.8(-72.1 to 1764.5) | | 63.5(-81.9 to 1110.8) | -28.9(-33.7 to -23.8) |
| **Bhutan** | 9511603(7966592 to 11315003) | 9817(1407 to 32308) | 400544(57394 to 1318083) | 9921964(8506210 to 11633954) |  | -61.0(-66.1 to -55.6) | - | 405.3(-52.0 to 7558.4) | -59.6(-63.9 to -54.7) |  | -27.5(-35.1 to -18.6) | | 13.0(-73.1 to 418.8) | | -25.3(-82.2 to 243.1) | -27.4(-32.8 to -20.9) |
| **Bolivia (Plurinational State of)** | 8302694(6922127 to 9766177) | 32734(4860 to 105173) | 414494(61527 to 1332553) | 8749923(7500864 to 10110190) |  | -49.3(-55.3 to -43.2) | - | 144.2(-70.6 to 2581.2) | -48.0(-52.9 to -42.4) |  | -29.5(-37.1 to -23.2) | | 76.2(-51.9 to 672.2) | | 14.5(-68.8 to 401.9) | -28.1(-32.8 to -23.0) |
| **Bosnia and Herzegovina** | 1235352(1063406 to 1447173) | 1242(327 to 3294) | 5668(1491 to 15040) | 1242263(1068799 to 1452323) |  | -43.8(-48.6 to -38.4) | - | 27.0(-69.5 to 777.8) | -43.6(-48.5 to -38.1) |  | -21.2(-26.0 to -16.0) | | -4.1(-74.3 to 169.3) | | -40.4(-84.0 to 67.4) | -21.3(-26.0 to -16.2) |
| **Botswana** | 32853169(27130238 to 39673913) | 10676(2000 to 32323) | 1654767(309978 to 5010611) | 34518612(29337263 to 41364627) |  | -24.1(-33.1 to -13.6) | - | 1063.8(190.1 to 9253.5) | -21.7(-30.7 to -10.8) |  | -14.5(-25.0 to -4.4) | | 91.5(-59.4 to 415.8) | | 27.4(-73.0 to 243.1) | -13.1(-21.8 to -4.2) |
| **Brazil** | 2679824(2256464 to 3148199) | 6720(1270 to 18711) | 85091(16077 to 236888) | 2771635(2343304 to 3273462) |  | -38.9(-42.6 to -34.7) | - | 2883.9(509.4 to 36723.6) | -37.5(-41.2 to -33.3) |  | -9.5(-16.2 to -3.8) | | 96.8(-55.6 to 404.2) | | 27.8(-71.2 to 227.4) | -8.6(-14.0 to -3.4) |
| **Brunei Darussalam** | 4997827(4361055 to 5775848) | 2057(323 to 6783) | 16361(2566 to 53937) | 5016246(4369096 to 5794960) |  | -35.9(-41.7 to -29.0) | - | 13.9(-85.5 to 1215.4) | -35.8(-41.6 to -29.0) |  | -13.8(-19.3 to -7.6) | | 121.4(-70.4 to 1109.9) | | 41.6(-81.1 to 674.1) | -13.7(-19.2 to -7.5) |
| **Bulgaria** | 1368945(1182310 to 1597493) | 7359(1996 to 17395) | 33585(9108 to 79383) | 1409889(1216586 to 1641168) |  | -15.7(-22.2 to -8.9) | - | 1594.7(312.4 to 15410.9) | -11.2(-18.2 to -4.0) |  | -25.2(-31.1 to -19.3) | | -40.3(-84.5 to 46.8) | | -62.9(-90.3 to -8.9) | -27.1(-32.4 to -21.6) |
| **Burkina Faso** | 18905058(16355195 to 21581077) | 3641(539 to 12030) | 564381(83553 to 1865687) | 19473080(17032506 to 22138329) |  | -2.4(-11.9 to 6.6) | - | 812.5(22.9 to 12968.8) | 0.4(-7.7 to 9.1) |  | -13.5(-20.8 to -6.1) | | 16.9(-75.7 to 470.4) | | -22.2(-83.9 to 279.7) | -13.8(-19.6 to -7.9) |
| **Burundi** | 46754879(39737587 to 54519870) | 9491(1352 to 32587) | 1471152(209506 to 5051186) | 48235522(42029180 to 55859370) |  | -22.2(-30.1 to -14.3) | - | 2197.4(174.8 to 31542.5) | -20.4(-27.4 to -12.4) |  | -11.0(-18.3 to -3.9) | | 77.7(-57.2 to 620.1) | | 18.2(-71.5 to 379.1) | -10.3(-15.9 to -4.7) |
| **Cabo Verde** | 9038825(7620784 to 10563635) | 2013(278 to 6115) | 311955(43178 to 948295) | 9352792(8082587 to 10911679) |  | -35.0(-41.7 to -28.3) | - | 458.1(-35.4 to 6561.2) | -33.1(-38.7 to -27.0) |  | -29.2(-35.9 to -22.6) | | 11.4(-76.0 to 423.2) | | -25.9(-84.1 to 248.1) | -29.1(-34.1 to -23.7) |
| **Cambodia** | 30914946(27051887 to 35497826) | 20376(3033 to 67769) | 223470(33265 to 743401) | 31158792(27231976 to 35660006) |  | -27.2(-31.6 to -22.9) | - | 1212.6(124.8 to 13264.6) | -26.2(-30.7 to -22.0) |  | -23.9(-28.8 to -18.0) | | -32.7(-86.9 to 111.8) | | -56.5(-91.5 to 37.0) | -24.3(-29.1 to -18.7) |
| **Cameroon** | 15003280(12939787 to 17396163) | 2268(327 to 6894) | 351548(50725 to 1068136) | 15357096(13302654 to 17662222) |  | -22.9(-31.5 to -15.2) | - | 492.7(-41.5 to 7140.1) | -21.2(-28.7 to -14.0) |  | -20.0(-26.7 to -12.9) | | 8.9(-74.2 to 432.3) | | -27.5(-82.8 to 254.1) | -20.2(-26.2 to -14.1) |
| **Canada** | 425076(367182 to 495070) | 666(241 to 1508) | 5297(1921 to 11989) | 431039(372253 to 501919) |  | -24.2(-28.7 to -19.1) | - | 1.7(-62.6 to 247.6) | -23.9(-28.4 to -19.1) |  | -2.9(-10.0 to 3.5) | | 53.1(-47.3 to 265.8) | | -2.0(-66.3 to 134.0) | -2.9(-9.8 to 3.4) |
| **Central African Republic** | 49883987(45088709 to 54815060) | 4748(742 to 15690) | 735933(115036 to 2431720) | 50624668(45981420 to 55572173) |  | -2.3(-7.9 to 3.7) | - | 395.0(-41.0 to 4581.9) | -1.3(-6.5 to 4.9) |  | -11.7(-16.4 to -6.9) | | 54.8(-65.4 to 594.2) | | 3.0(-77.0 to 361.7) | -11.5(-15.5 to -7.1) |
| **Chad** | 20766148(17994561 to 23793029) | 3657(503 to 11226) | 566883(77943 to 1740608) | 21336689(18618063 to 24469660) |  | -11.2(-19.0 to -3.5) | - | 610.3(-36.2 to 10575.5) | -8.8(-15.2 to -1.5) |  | -14.9(-21.7 to -6.4) | | 14.9(-74.7 to 391.4) | | -23.6(-83.2 to 226.8) | -15.1(-21.3 to -8.3) |
| **Chile** | 994770(868586 to 1155074) | 1364(457 to 3258) | 10852(3636 to 25903) | 1006986(878625 to 1165573) |  | -57.4(-60.5 to -54.0) | - | 76.1(-66.4 to 1347.5) | -57.0(-60.1 to -53.6) |  | -19.2(-24.9 to -13.8) | | 33.0(-71.2 to 424.0) | | -14.9(-81.6 to 235.3) | -19.1(-24.3 to -13.8) |
| **China** | 4099614(3515559 to 4665500) | 16119(2999 to 51309) | 176777(32897 to 562753) | 4292509(3815890 to 4792282) |  | -44.2(-47.3 to -40.5) | - | 0.3(-67.1 to 355.9) | -42.6(-44.8 to -40.2) |  | -30.7(-37.1 to -26.5) | | -20.0(-82.9 to 126.8) | | -48.2(-88.9 to 46.7) | -31.6(-33.5 to -29.4) |
| **Colombia** | 1779492(1498739 to 2058330) | 6269(1064 to 16919) | 79383(13472 to 214153) | 1865144(1618148 to 2131907) |  | -44.7(-50.0 to -39.4) | - | 903.0(106.3 to 8279.7) | -42.7(-47.5 to -37.6) |  | -14.5(-22.6 to -7.6) | | 60.2(-62.7 to 297.8) | | 4.1(-75.8 to 158.2) | -13.7(-20.0 to -7.2) |
| **Comoros** | 24342286(19657125 to 29044890) | 17182(2203 to 56113) | 2663226(341517 to 8697374) | 27022695(23276964 to 31562542) |  | -34.7(-41.7 to -26.8) | - | 2835.1(244.9 to 35495.6) | -32.3(-38.2 to -25.0) |  | -12.9(-26.8 to -2.7) | | 276.9(-22.3 to 1696.8) | | 150.7(-48.3 to 1094.7) | -6.9(-14.1 to 1.8) |
| **Congo** | 22778749(19845900 to 26025306) | 3060(363 to 10705) | 474229(56338 to 1658886) | 23256038(20354056 to 26528213) |  | -15.5(-20.8 to -10.5) | - | 353.3(-57.2 to 6105.9) | -14.4(-18.7 to -10.0) |  | -12.8(-19.1 to -5.7) | | 73.5(-63.4 to 759.1) | | 15.4(-75.7 to 471.7) | -12.4(-17.7 to -6.1) |
| **Cook Islands** | 1780292(1519614 to 2082055) | 456(41 to 1960) | 4998(451 to 21507) | 1785746(1524251 to 2085585) |  | -19.9(-24.1 to -15.6) | - | 156.8(-79.9 to 3005.6) | -19.8(-24.0 to -15.2) |  | -12.9(-17.8 to -7.8) | | 60.6(-70.9 to 831.5) | | 3.9(-81.2 to 502.9) | -12.8(-17.7 to -7.8) |
| **Costa Rica** | 744909(640353 to 857011) | 1180(167 to 4098) | 14941(2121 to 51906) | 761030(660746 to 873132) |  | -44.8(-49.1 to -40.3) | - | 1201.8(118.0 to 15416.0) | -43.7(-47.9 to -39.3) |  | -31.5(-36.9 to -26.5) | | 11.7(-74.8 to 240.8) | | -27.4(-83.6 to 121.4) | -31.4(-35.8 to -26.6) |
| **Croatia** | 700959(611269 to 814751) | 563(133 to 1565) | 2571(608 to 7145) | 704094(612967 to 818186) |  | -50.9(-54.3 to -46.8) | - | 9.6(-75.6 to 697.7) | -50.7(-54.3 to -46.6) |  | -39.2(-43.6 to -35.0) | | -43.1(-85.9 to 68.9) | | -64.6(-91.2 to 4.9) | -39.4(-43.6 to -35.2) |
| **Cuba** | 498162(428256 to 584139) | 940(184 to 2688) | 11908(2328 to 34036) | 511011(441056 to 598432) |  | -42.7(-47.7 to -36.9) | - | -49.3(-84.1 to 109.4) | -42.8(-47.5 to -36.9) |  | -31.8(-37.4 to -26.2) | | 83.4(-63.8 to 525.9) | | 19.1(-76.5 to 306.3) | -31.0(-36.4 to -25.8) |
| **Cyprus** | 266290(224069 to 315380) | 652(123 to 1957) | 5185(980 to 15564) | 272126(231560 to 320672) |  | -51.9(-56.0 to -47.8) | - | 529.8(34.4 to 6952.2) | -48.5(-52.4 to -44.5) |  | -20.2(-25.7 to -14.0) | | -66.0(-92.7 to 2.0) | | -78.3(-95.3 to -34.7) | -24.3(-28.4 to -20.0) |
| **Czechia** | 391613(338050 to 458046) | 1743(620 to 3988) | 7957(2831 to 18201) | 401313(345353 to 469061) |  | -53.0(-57.1 to -48.7) | - | 38.7(-61.7 to 764.5) | -52.2(-56.3 to -47.9) |  | -28.7(-33.7 to -23.3) | | 4.6(-65.4 to 158.5) | | -35.0(-78.5 to 60.6) | -28.8(-33.3 to -23.9) |
| **C么te d'Ivoire** | 16424279(14076385 to 18879564) | 3536(530 to 11706) | 548014(82155 to 1814643) | 16975828(14856999 to 19408700) |  | -15.3(-22.6 to -7.6) | - | 120.9(-52.2 to 1268.3) | -13.7(-20.6 to -6.9) |  | -16.9(-24.0 to -9.9) | | 33.8(-66.8 to 309.9) | | -11.0(-77.9 to 172.7) | -16.7(-22.5 to -9.9) |
| **Democratic People's Republic of Korea** | 10658546(9315412 to 12008300) | 44102(14103 to 100381) | 483685(154709 to 1101130) | 11186333(9867367 to 12539473) |  | -25.8(-31.9 to -20.2) | - | 4445.5(718.0 to 44751.3) | -22.5(-28.1 to -17.0) |  | -15.0(-22.2 to -7.0) | | 39.6(-67.5 to 472.0) | | -9.7(-79.0 to 270.4) | -14.6(-19.9 to -8.7) |
| **Democratic Republic of the Congo** | 30485759(26634180 to 34346527) | 4863(615 to 17698) | 753802(95253 to 2741993) | 31244424(27689184 to 35022132) |  | 3.8(-3.6 to 10.4) | - | 588.0(-29.3 to 6847.2) | 5.4(-0.8 to 11.7) |  | -11.9(-18.3 to -4.9) | | 82.0(-64.4 to 628.3) | | 21.1(-76.3 to 384.5) | -11.3(-16.6 to -5.6) |
| **Denmark** | 464670(394922 to 547599) | 1067(311 to 2730) | 8490(2476 to 21708) | 474227(402594 to 559334) |  | -38.0(-42.1 to -32.7) | - | 143.0(-46.7 to 1647.5) | -37.3(-41.5 to -32.1) |  | -25.1(-30.5 to -19.8) | | 62.9(-54.2 to 407.9) | | 4.2(-70.8 to 224.8) | -24.7(-29.7 to -19.5) |
| **Djibouti** | 22010658(18483877 to 25676133) | 9992(4238 to 20295) | 1548760(657068 to 3145854) | 23569410(20327387 to 27305347) |  | -30.4(-37.5 to -22.4) | - | 7534.3(1603.7 to 73284.8) | -27.4(-33.9 to -19.8) |  | -28.3(-36.6 to -20.2) | | 74.6(-53.4 to 541.1) | | 16.1(-69.0 to 326.3) | -26.4(-32.7 to -20.1) |
| **Dominica** | 2374788(2033421 to 2794477) | 1286(118 to 5593) | 16282(1500 to 70805) | 2392356(2057912 to 2804784) |  | -31.0(-36.0 to -25.0) | - | -69.5(-97.3 to 434.1) | -31.4(-36.1 to -26.3) |  | -10.1(-16.0 to -3.9) | | 94.2(-64.3 to 1056.5) | | 26.2(-76.8 to 651.5) | -9.9(-15.7 to -4.0) |
| **Dominican Republic** | 4163387(3520887 to 4922490) | 1759(179 to 8428) | 22271(2270 to 106713) | 4187417(3543564 to 4931215) |  | -37.9(-44.0 to -30.9) | - | -51.2(-96.0 to 449.9) | -37.9(-44.1 to -31.0) |  | 2.0(-5.3 to 9.1) | | 165.7(-57.7 to 1553.5) | | 72.6(-72.5 to 974.5) | 2.2(-4.7 to 9.6) |
| **Ecuador** | 3289560(2673783 to 3876750) | 13914(2572 to 42516) | 176182(32559 to 538565) | 3479655(2972470 to 4041066) |  | -61.7(-65.9 to -57.3) | - | 836.1(32.6 to 9288.9) | -59.6(-63.0 to -55.5) |  | -22.7(-30.7 to -15.3) | | 12.6(-73.6 to 247.1) | | -26.9(-82.8 to 125.4) | -22.8(-27.3 to -17.9) |
| **Egypt** | 1296458(1117184 to 1491281) | 2188(494 to 5576) | 61991(14003 to 157928) | 1360637(1192558 to 1569720) |  | -31.9(-37.4 to -26.1) | - | 2213.4(500.1 to 20171.9) | -28.1(-34.0 to -22.1) |  | -19.3(-27.0 to -11.7) | | 5.9(-78.7 to 204.7) | | -30.2(-86.0 to 100.6) | -19.9(-25.2 to -13.9) |
| **El Salvador** | 1192667(1021008 to 1376328) | 1745(218 to 6694) | 22092(2765 to 84756) | 1216503(1044407 to 1399542) |  | -53.5(-58.3 to -48.9) | - | 398.0(-50.6 to 4979.9) | -52.6(-56.9 to -47.9) |  | -14.6(-20.7 to -7.7) | | 21.9(-70.3 to 517.0) | | -20.8(-80.7 to 301.1) | -14.6(-20.2 to -8.7) |
| **Equatorial Guinea** | 15532708(13335533 to 17930517) | 2608(330 to 8374) | 404225(51171 to 1297839) | 15939541(13802717 to 18381934) |  | -50.7(-55.2 to -45.9) | - | 217.9(-66.7 to 3612.6) | -50.0(-54.4 to -45.2) |  | -19.1(-25.4 to -12.5) | | 77.5(-62.7 to 657.4) | | 18.1(-75.2 to 403.7) | -18.5(-23.6 to -12.9) |
| **Eritrea** | 46910517(39689047 to 54765004) | 12034(1794 to 40000) | 1865210(278119 to 6199794) | 48787761(42414750 to 56311407) |  | -16.3(-25.2 to -7.7) | - | 2818.5(241.0 to 42639.6) | -13.9(-21.2 to -6.0) |  | -20.6(-28.1 to -12.7) | | 60.2(-62.3 to 626.0) | | 6.5(-74.9 to 383.0) | -19.8(-25.5 to -13.8) |
| **Estonia** | 1000321(792058 to 1233959) | 48935(25182 to 82903) | 223343(114910 to 378286) | 1272600(1081679 to 1503646) |  | -57.4(-61.7 to -51.9) | - | 74.7(-14.9 to 354.6) | -45.9(-50.9 to -40.4) |  | -36.5(-47.9 to -26.2) | | -29.9(-62.8 to 19.6) | | -56.5(-76.9 to -25.7) | -41.0(-45.8 to -36.0) |
| **Eswatini** | 28550174(22052021 to 35479900) | 21847(5249 to 53654) | 3386255(813599 to 8316470) | 31958276(26990997 to 38472069) |  | -50.2(-57.7 to -41.5) | - | 1510.0(383.7 to 7651.2) | -44.6(-52.4 to -35.3) |  | -4.7(-21.7 to 11.6) | | 46.2(-62.9 to 233.9) | | -2.7(-75.3 to 122.0) | -4.5(-16.2 to 8.7) |
| **Ethiopia** | 24623039(21238783 to 28249720) | 5232(996 to 17748) | 810887(154426 to 2751281) | 25439158(22387878 to 28981945) |  | -34.1(-37.7 to -30.6) | - | 2314.5(227.9 to 31937.5) | -32.7(-36.0 to -29.3) |  | -19.4(-25.4 to -15.3) | | 85.9(-52.3 to 501.2) | | 23.7(-68.3 to 299.8) | -18.5(-21.4 to -15.5) |
| **Fiji** | 3133121(2749356 to 3533479) | 557(50 to 2351) | 6112(550 to 25772) | 3139790(2754411 to 3533964) |  | -16.1(-20.4 to -11.4) | - | 205.0(-80.4 to 3903.2) | -16.0(-20.4 to -11.3) |  | -7.7(-12.5 to -2.4) | | 137.1(-61.3 to 1259.5) | | 53.4(-75.0 to 780.1) | -7.7(-12.4 to -2.2) |
| **Finland** | 414083(348244 to 488575) | 1784(573 to 4544) | 14192(4555 to 36160) | 430059(365350 to 503377) |  | -48.8(-52.6 to -44.9) | - | 322.8(-1.8 to 3389.4) | -47.8(-51.6 to -43.9) |  | -27.4(-33.1 to -22.4) | | 82.9(-44.5 to 439.5) | | 17.1(-64.5 to 245.3) | -26.3(-30.5 to -21.7) |
| **France** | 630499(535005 to 742955) | 1542(552 to 3365) | 12265(4396 to 26771) | 644306(545119 to 760179) |  | -49.3(-53.0 to -45.4) | - | 69.1(-45.3 to 655.7) | -48.6(-52.3 to -44.8) |  | -11.1(-16.9 to -5.0) | | 57.9(-46.8 to 283.6) | | 1.1(-66.0 to 145.3) | -10.8(-16.2 to -4.7) |
| **Gabon** | 23162171(19845894 to 26766436) | 4731(619 to 17143) | 733262(95844 to 2657007) | 23900164(20807230 to 27431551) |  | -14.7(-20.8 to -8.9) | - | 584.2(-32.4 to 8539.9) | -12.9(-17.6 to -7.9) |  | -16.8(-24.0 to -9.5) | | 60.7(-62.1 to 630.5) | | 6.9(-74.8 to 385.8) | -16.2(-21.4 to -10.2) |
| **Gambia** | 23210059(19863618 to 26974920) | 3460(443 to 11411) | 536232(68583 to 1768795) | 23749751(20571518 to 27472779) |  | -19.6(-28.3 to -11.3) | - | 511.5(-31.2 to 7391.3) | -17.7(-25.4 to -9.2) |  | -24.2(-29.1 to -18.2) | | -3.0(-80.4 to 323.1) | | -35.5(-87.0 to 181.4) | -24.5(-28.6 to -19.8) |
| **Georgia** | 4282637(3486270 to 5143814) | 218271(125039 to 344199) | 996180(570865 to 1571029) | 5497088(4740716 to 6408060) |  | -16.5(-22.6 to -9.7) | - | 8648.2(2332.2 to 73733.5) | 1.5(-5.8 to 9.8) |  | -26.9(-37.7 to -16.1) | | 42.9(-17.9 to 124.0) | | -11.2(-49.0 to 39.2) | -22.9(-29.6 to -15.9) |
| **Germany** | 589332(494256 to 707596) | 2957(1172 to 6215) | 23521(9319 to 49385) | 615811(513811 to 739935) |  | -55.4(-58.8 to -52.0) | - | 55.0(-50.4 to 638.2) | -54.6(-58.1 to -51.2) |  | 14.0(2.7 to 27.3) | | 197.7(16.3 to 565.9) | | 90.5(-25.7 to 325.9) | 16.1(5.0 to 29.0) |
| **Ghana** | 19400502(16763622 to 22670041) | 3290(498 to 9497) | 509958(77249 to 1471439) | 19913750(17309905 to 23217771) |  | -19.1(-27.2 to -11.7) | - | 488.0(-30.5 to 7364.1) | -17.3(-24.0 to -10.2) |  | -18.5(-25.8 to -10.4) | | 23.8(-72.7 to 449.9) | | -17.6(-81.8 to 265.8) | -18.5(-25.0 to -11.4) |
| **Greece** | 373340(314853 to 437761) | 1461(253 to 4530) | 11618(2010 to 36005) | 386418(328438 to 452663) |  | -45.2(-48.9 to -40.6) | - | 635.4(65.0 to 7359.6) | -42.2(-46.2 to -37.7) |  | -19.0(-24.9 to -13.1) | | -28.0(-83.6 to 93.1) | | -53.9(-89.5 to 23.5) | -20.9(-25.1 to -16.0) |
| **Greenland** | 4054302(3413175 to 4780078) | 10537(1514 to 36683) | 83809(12038 to 291915) | 4148648(3555600 to 4865424) |  | 8.4(0.4 to 17.3) | - | 24.5(-86.5 to 1042.2) | 8.8(1.9 to 16.6) |  | -12.0(-18.0 to -6.3) | | 74.7(-62.7 to 798.0) | | 11.8(-76.1 to 474.7) | -11.5(-16.3 to -6.7) |
| **Grenada** | 1107363(943447 to 1299718) | 693(59 to 3462) | 8774(743 to 43836) | 1116829(953164 to 1311516) |  | -30.3(-36.2 to -23.3) | - | -31.0(-93.5 to 502.7) | -30.2(-36.1 to -23.3) |  | -25.4(-30.6 to -20.0) | | 141.7(-68.5 to 1491.1) | | 57.0(-79.5 to 933.7) | -25.1(-29.9 to -20.0) |
| **Guam** | 2879302(2468018 to 3332083) | 1257(138 to 4468) | 13788(1512 to 49005) | 2894347(2492404 to 3345597) |  | -38.2(-41.7 to -34.1) | - | 803.9(65.5 to 10690.8) | -37.1(-40.6 to -33.3) |  | -11.0(-16.4 to -5.3) | | -63.4(-94.9 to 39.0) | | -76.3(-96.7 to -10.1) | -12.2(-17.0 to -7.3) |
| **Guatemala** | 1635419(1438866 to 1848638) | 3302(486 to 10323) | 41817(6154 to 130654) | 1680539(1489375 to 1893800) |  | -57.4(-61.2 to -53.3) | - | 653.5(9.5 to 8490.9) | -56.2(-59.9 to -52.5) |  | -15.7(-21.5 to -9.1) | | 18.9(-68.6 to 269.6) | | -22.8(-79.6 to 140.1) | -15.9(-20.7 to -10.5) |
| **Guinea** | 17682993(15382728 to 20273740) | 2511(340 to 8225) | 389144(52728 to 1274888) | 18074647(15753364 to 20719995) |  | -17.0(-24.8 to -9.4) | - | 462.1(-41.0 to 7263.3) | -15.2(-22.3 to -8.1) |  | -12.1(-19.1 to -4.3) | | 11.3(-71.9 to 399.0) | | -26.0(-81.3 to 232.1) | -12.5(-18.6 to -5.8) |
| **Guinea-Bissau** | 15275163(13238755 to 17539652) | 1814(276 to 5897) | 281132(42860 to 914458) | 15558110(13615937 to 17978857) |  | -34.8(-40.4 to -28.9) | - | 379.4(-40.5 to 5602.8) | -33.4(-38.6 to -27.6) |  | -12.4(-19.5 to -4.4) | | -1.1(-78.6 to 314.3) | | -34.2(-85.8 to 175.6) | -13.0(-19.1 to -5.8) |
| **Guyana** | 4162526(3569112 to 4876654) | 2593(244 to 13452) | 32831(3089 to 170384) | 4197950(3615501 to 4891913) |  | -14.4(-21.7 to -6.5) | - | 59.6(-85.0 to 1728.1) | -14.2(-21.5 to -6.1) |  | -14.0(-21.3 to -6.5) | | 124.3(-63.6 to 1311.5) | | 45.7(-76.3 to 816.7) | -13.7(-21.0 to -6.5) |
| **Haiti** | 7344548(6485991 to 8265394) | 2343(242 to 8940) | 29664(3063 to 113223) | 7376555(6505508 to 8307601) |  | -40.4(-44.7 to -36.3) | - | -40.8(-94.8 to 526.3) | -40.3(-44.7 to -36.3) |  | -8.2(-14.8 to -1.4) | | 121.0(-64.9 to 1502.0) | | 43.5(-77.2 to 941.0) | -8.1(-14.4 to -1.3) |
| **Honduras** | 3079433(2654318 to 3510079) | 6170(989 to 19674) | 78121(12526 to 249133) | 3163724(2761922 to 3583564) |  | -19.7(-26.0 to -13.7) | - | 2147.5(235.9 to 27544.1) | -17.8(-23.7 to -11.9) |  | -9.9(-16.3 to -3.2) | | 46.0(-63.6 to 336.2) | | -5.2(-76.4 to 183.3) | -9.7(-15.8 to -4.0) |
| **Hungary** | 513733(444162 to 600545) | 1542(282 to 5156) | 7037(1287 to 23524) | 522312(453003 to 610367) |  | -47.7(-52.2 to -42.8) | - | 624.1(64.3 to 7504.4) | -45.7(-50.4 to -40.7) |  | -43.4(-47.9 to -38.2) | | -65.2(-93.1 to 20.6) | | -78.4(-95.7 to -25.1) | -44.7(-49.0 to -40.0) |
| **Iceland** | 526546(436337 to 628379) | 658(95 to 2444) | 5233(752 to 19448) | 532437(440283 to 634781) |  | -38.5(-43.8 to -33.2) | - | 668.5(31.6 to 6797.4) | -35.4(-39.7 to -30.8) |  | -16.3(-21.7 to -9.8) | | -74.1(-95.0 to -12.9) | | -83.4(-96.8 to -44.3) | -19.7(-23.9 to -15.2) |
| **India** | 20652593(16983549 to 24534917) | 39444(7404 to 98765) | 1609316(302136 to 4029593) | 22301352(19136523 to 25971953) |  | -34.6(-40.0 to -29.0) | - | 5409.5(840.5 to 81040.4) | -30.3(-35.1 to -25.2) |  | -19.0(-26.7 to -13.2) | | 46.6(-62.1 to 282.5) | | -3.0(-74.9 to 152.9) | -18.0(-20.6 to -15.0) |
| **Indonesia** | 13680734(12117682 to 15300044) | 4810(575 to 18303) | 52752(6299 to 200716) | 13738296(12157611 to 15337343) |  | -57.5(-60.2 to -54.8) | - | 110.9(-84.5 to 3158.1) | -57.2(-59.7 to -54.7) |  | -31.0(-33.2 to -28.0) | | -50.8(-90.4 to 203.6) | | -68.2(-93.8 to 96.4) | -31.3(-33.3 to -28.9) |
| **Iran (Islamic Republic of)** | 1207004(1049028 to 1380847) | 762(215 to 1858) | 21581(6095 to 52635) | 1229347(1071067 to 1409718) |  | -30.9(-34.2 to -27.6) | - | 1295.5(168.6 to 18799.7) | -29.8(-33.0 to -26.7) |  | -16.0(-20.0 to -12.1) | | 39.3(-70.2 to 487.7) | | -8.2(-80.4 to 287.2) | -15.9(-18.7 to -12.7) |
| **Iraq** | 3300534(2834808 to 3838668) | 3456(1026 to 8058) | 97919(29094 to 228331) | 3401909(2942206 to 3954269) |  | -41.0(-45.7 to -35.4) | - | 1608.5(299.0 to 24018.7) | -39.5(-44.4 to -34.0) |  | -28.9(-34.3 to -24.0) | | 28.2(-68.4 to 350.2) | | -15.5(-79.1 to 196.7) | -28.6(-32.7 to -24.5) |
| **Ireland** | 540169(458068 to 634881) | 964(244 to 2721) | 7668(1943 to 21644) | 548801(466143 to 647765) |  | -40.6(-45.0 to -36.0) | - | 112.9(-48.9 to 1902.0) | -39.7(-44.2 to -35.1) |  | -25.8(-30.2 to -20.9) | | -11.8(-79.8 to 174.6) | | -43.6(-87.1 to 75.7) | -26.2(-30.2 to -21.4) |
| **Israel** | 290758(244005 to 341731) | 2184(711 to 5084) | 17368(5658 to 40419) | 310309(264692 to 364089) |  | -42.5(-47.0 to -37.2) | - | 32.9(-64.5 to 839.9) | -40.6(-45.1 to -35.8) |  | -31.9(-39.0 to -25.4) | | 23.3(-63.6 to 225.3) | | -21.1(-76.7 to 108.2) | -31.1(-35.3 to -26.7) |
| **Italy** | 753336(614450 to 921367) | 2747(1084 to 5640) | 21851(8620 to 44857) | 777935(634252 to 947923) |  | -41.1(-44.2 to -37.5) | - | 155.9(-27.6 to 1372.8) | -39.1(-42.2 to -35.6) |  | -18.5(-22.5 to -14.7) | | -10.8(-64.5 to 100.2) | | -42.9(-77.3 to 28.1) | -19.5(-22.3 to -16.6) |
| **Jamaica** | 364806(311360 to 428631) | 447(70 to 1579) | 5658(881 to 19995) | 370911(318627 to 434808) |  | -38.0(-42.7 to -33.0) | - | 33.0(-86.1 to 2111.2) | -37.7(-42.4 to -33.0) |  | -29.0(-34.5 to -23.6) | | 86.9(-66.3 to 875.6) | | 21.4(-78.1 to 533.7) | -28.4(-33.2 to -23.1) |
| **Japan** | 663248(557748 to 785050) | 694(75 to 2894) | 5524(593 to 23021) | 669466(562897 to 789144) |  | -62.2(-65.2 to -58.9) | - | -54.8(-93.6 to 179.1) | -62.2(-65.1 to -58.7) |  | -25.0(-28.1 to -22.0) | | 35.0(-75.6 to 448.0) | | -13.6(-84.4 to 250.6) | -24.9(-27.7 to -22.2) |
| **Jordan** | 462684(384009 to 546509) | 689(121 to 2339) | 19511(3424 to 66276) | 482883(407995 to 565601) |  | -57.0(-60.0 to -54.1) | - | 1459.0(237.6 to 19757.5) | -54.3(-56.8 to -51.6) |  | -27.0(-33.9 to -21.1) | | -25.8(-84.8 to 121.8) | | -51.1(-90.0 to 46.1) | -28.4(-32.4 to -24.4) |
| **Kazakhstan** | 4327862(3433220 to 5311293) | 260949(140504 to 421175) | 1190965(641195 to 1921749) | 5779776(4956019 to 6741295) |  | -36.5(-42.6 to -29.2) | - | 8546.2(2141.4 to 72700.6) | 1.2(-7.5 to 10.3) |  | -34.0(-45.7 to -22.2) | | -44.7(-68.9 to -11.7) | | -65.6(-80.7 to -45.2) | -44.9(-49.4 to -39.9) |
| **Kenya** | 26502395(23093431 to 30559927) | 2714(871 to 6357) | 420718(135027 to 985453) | 26925827(23477875 to 31060696) |  | -8.0(-11.5 to -4.3) | - | 3366.4(756.2 to 46090.3) | -6.5(-9.6 to -3.0) |  | -7.3(-10.7 to -3.4) | | 29.6(-70.0 to 423.0) | | -13.8(-80.0 to 247.9) | -7.4(-10.3 to -4.1) |
| **Kiribati** | 21898025(19730109 to 24248055) | 5734(558 to 23095) | 62890(6118 to 253288) | 21966649(19751247 to 24349656) |  | -10.2(-13.9 to -6.3) | - | 209.4(-73.6 to 3782.4) | -10.0(-13.7 to -6.1) |  | 2.1(-2.9 to 7.1) | | 74.9(-70.4 to 1008.9) | | 13.2(-80.9 to 617.6) | 2.1(-2.4 to 7.1) |
| **Kuwait** | 1708490(1471268 to 1985683) | 982(268 to 2493) | 27827(7589 to 70613) | 1737299(1497401 to 2027622) |  | -36.8(-41.4 to -32.0) | - | 597.0(62.4 to 9976.1) | -36.0(-40.6 to -31.2) |  | -31.2(-35.8 to -26.1) | | 16.3(-71.4 to 271.3) | | -23.4(-81.1 to 144.6) | -31.1(-35.3 to -26.2) |
| **Kyrgyzstan** | 5975749(3922503 to 7803934) | 483709(171783 to 898309) | 2207637(784014 to 4099727) | 8667095(7418200 to 10055531) |  | -35.3(-43.3 to -26.3) | - | 34068.4(8226.5 to 351384.2) | -5.6(-14.4 to 3.5) |  | -10.8(-42.2 to 17.9) | | 31.5(-56.2 to 176.6) | | -18.3(-72.8 to 71.8) | -11.3(-17.7 to -3.1) |
| **Lao People's Democratic Republic** | 12052138(10483740 to 14120269) | 5192(593 to 19405) | 56943(6503 to 212823) | 12114273(10515199 to 14223918) |  | -42.2(-45.7 to -38.6) | - | 227.4(-65.3 to 4392.0) | -41.7(-45.1 to -38.2) |  | -15.6(-20.7 to -10.1) | | -30.8(-87.5 to 303.7) | | -55.3(-91.9 to 161.0) | -16.0(-20.7 to -11.1) |
| **Latvia** | 1998400(1681082 to 2387339) | 47346(23057 to 84502) | 216085(105162 to 385724) | 2261831(1915810 to 2673712) |  | -31.9(-40.4 to -19.2) | - | -46.8(-71.5 to 15.8) | -33.1(-38.9 to -26.0) |  | -32.5(-40.0 to -25.2) | | -19.0(-59.3 to 46.8) | | -49.7(-74.7 to -8.8) | -34.4(-39.1 to -29.2) |
| **Lebanon** | 1178096(1002206 to 1390513) | 777(98 to 3289) | 22010(2771 to 93167) | 1200883(1032075 to 1405732) |  | -53.6(-57.3 to -49.6) | - | 462.4(-31.1 to 6607.2) | -53.0(-56.6 to -49.0) |  | -25.5(-30.9 to -20.5) | | 42.3(-71.2 to 420.5) | | -6.2(-81.0 to 242.9) | -25.2(-29.0 to -20.9) |
| **Lesotho** | 40859575(34824987 to 48974629) | 12320(4250 to 26996) | 1909628(658932 to 4185088) | 42781523(36810604 to 50896073) |  | -20.9(-30.9 to -8.4) | - | 3941.9(821.9 to 45885.0) | -17.7(-27.8 to -5.5) |  | 4.4(-8.2 to 19.7) | | 79.1(-60.5 to 620.5) | | 19.1(-73.7 to 379.2) | 4.9(-7.0 to 19.2) |
| **Liberia** | 16488941(14169988 to 19026178) | 2104(247 to 8469) | 326168(38338 to 1312514) | 16817213(14630966 to 19389924) |  | -27.6(-35.2 to -20.1) | - | 300.8(-64.2 to 4990.7) | -26.4(-33.5 to -19.1) |  | -19.6(-25.8 to -13.3) | | 12.6(-73.9 to 402.0) | | -25.1(-82.6 to 233.9) | -19.7(-24.8 to -14.3) |
| **Libya** | 1480432(1264172 to 1726470) | 1041(115 to 3853) | 29496(3256 to 109208) | 1510969(1302076 to 1760984) |  | -45.6(-50.6 to -40.9) | - | 430.4(-45.6 to 6472.4) | -44.7(-49.0 to -40.2) |  | -9.1(-14.9 to -2.7) | | 48.4(-74.3 to 720.2) | | -2.2(-83.1 to 440.7) | -8.9(-13.3 to -4.0) |
| **Lithuania** | 2742581(2283656 to 3270491) | 129463(74333 to 197370) | 590868(339317 to 900845) | 3462913(2969422 to 4079741) |  | -38.2(-44.1 to -30.3) | - | 179.8(1.8 to 1095.0) | -26.7(-33.1 to -19.2) |  | -30.6(-40.6 to -21.3) | | 13.6(-32.7 to 73.3) | | -29.4(-58.2 to 7.7) | -29.4(-34.8 to -23.4) |
| **Luxembourg** | 596397(489699 to 719082) | 2000(290 to 7067) | 15909(2303 to 56230) | 614305(512180 to 729085) |  | -40.2(-45.3 to -34.8) | - | 728.4(44.8 to 7994.0) | -37.7(-42.0 to -33.0) |  | -11.1(-18.5 to -3.5) | | -12.0(-85.9 to 250.9) | | -43.7(-91.0 to 124.7) | -12.4(-17.4 to -6.9) |
| **Madagascar** | 34630911(29393782 to 40463534) | 6086(924 to 18174) | 943365(143159 to 2816543) | 35580362(30734155 to 41379325) |  | -22.2(-29.1 to -14.9) | - | 2044.7(260.5 to 21280.0) | -21.3(-28.1 to -13.9) |  | -19.2(-25.1 to -12.7) | | 157.1(-41.3 to 700.0) | | 71.0(-61.0 to 432.0) | -18.0(-23.1 to -11.8) |
| **Malawi** | 24323860(20838414 to 28237419) | 3236(647 to 9709) | 501619(100199 to 1504864) | 24828715(21372006 to 28927805) |  | -38.8(-45.6 to -31.0) | - | 1477.3(221.4 to 18220.4) | -38.2(-45.1 to -30.4) |  | -18.9(-27.5 to -10.5) | | 156.8(-51.9 to 804.2) | | 70.8(-68.0 to 501.6) | -18.0(-26.8 to -9.6) |
| **Malaysia** | 9609338(8288441 to 11219558) | 7332(1776 to 19969) | 80408(19490 to 218987) | 9697077(8374184 to 11306577) |  | -6.9(-12.9 to -1.1) | - | 1429.4(159.2 to 15483.0) | -5.9(-12.0 to 0.0) |  | -4.9(-10.6 to 0.8) | | 13.5(-79.3 to 486.2) | | -26.5(-86.6 to 279.6) | -5.1(-10.6 to 0.9) |
| **Maldives** | 3127104(2706302 to 3613268) | 1221(131 to 4978) | 13388(1435 to 54618) | 3141712(2717019 to 3620168) |  | -56.3(-59.7 to -52.8) | - | 155.8(-77.9 to 4069.5) | -55.9(-59.1 to -52.4) |  | -15.6(-20.2 to -10.0) | | -41.1(-88.1 to 226.5) | | -61.9(-92.3 to 111.2) | -16.1(-20.3 to -10.8) |
| **Mali** | 15726794(13501145 to 18166740) | 2789(348 to 8503) | 432253(53986 to 1318685) | 16161836(14048455 to 18537209) |  | -22.8(-30.7 to -14.9) | - | 482.6(-34.6 to 7517.4) | -20.9(-27.8 to -13.8) |  | -20.5(-27.0 to -12.5) | | 15.7(-73.6 to 453.9) | | -23.1(-82.5 to 268.5) | -20.5(-26.0 to -13.8) |
| **Malta** | 874062(713112 to 1061361) | 856(121 to 3182) | 6811(961 to 25311) | 881730(721119 to 1074391) |  | -31.8(-37.1 to -25.9) | - | 542.9(-9.7 to 7678.8) | -29.9(-34.8 to -24.1) |  | 8.4(0.2 to 17.6) | | -55.1(-91.6 to 60.4) | | -71.3(-94.6 to 2.7) | 6.0(-1.7 to 14.0) |
| **Marshall Islands** | 11376126(10136106 to 12615174) | 10901(1825 to 36163) | 119550(20007 to 396525) | 11506577(10257947 to 12745951) |  | -26.4(-30.3 to -22.5) | - | 2552.1(400.5 to 34949.4) | -24.7(-28.2 to -20.6) |  | -18.9(-23.4 to -14.1) | | -43.8(-91.0 to 152.3) | | -63.6(-94.2 to 63.3) | -19.9(-23.9 to -15.7) |
| **Mauritania** | 8499650(7286635 to 9932897) | 1275(156 to 4064) | 197613(24261 to 630063) | 8698538(7474610 to 10063278) |  | -23.6(-31.3 to -15.8) | - | 534.6(-35.2 to 6741.4) | -21.7(-28.6 to -14.2) |  | -29.6(-35.3 to -23.2) | | -9.4(-77.8 to 241.5) | | -39.7(-85.2 to 127.2) | -29.9(-34.5 to -24.9) |
| **Mauritius** | 1049379(912702 to 1216195) | 471(110 to 1422) | 5168(1211 to 15593) | 1055019(917483 to 1222807) |  | -23.5(-28.4 to -18.8) | - | 1088.1(109.6 to 12407.0) | -22.6(-27.6 to -17.8) |  | -20.9(-25.6 to -15.8) | | -48.8(-86.8 to 95.0) | | -66.9(-91.5 to 26.2) | -21.4(-26.0 to -16.3) |
| **Mexico** | 1525077(1287647 to 1790646) | 3799(749 to 11315) | 48107(9479 to 143271) | 1576983(1348555 to 1845314) |  | -38.6(-41.8 to -34.9) | - | 2898.2(563.5 to 27556.2) | -36.8(-40.0 to -33.2) |  | -1.0(-8.2 to 4.6) | | 74.2(-60.6 to 445.7) | | 13.2(-74.4 to 254.5) | -0.5(-5.6 to 4.5) |
| **Micronesia (Federated States of)** | 6285339(5527202 to 7065417) | 1783(114 to 8536) | 19549(1255 to 93632) | 6306670(5546181 to 7072835) |  | -24.0(-27.5 to -20.3) | - | 128.8(-83.5 to 2951.0) | -23.9(-27.3 to -20.2) |  | -8.8(-13.7 to -3.9) | | 61.1(-70.9 to 773.1) | | 4.3(-81.2 to 464.9) | -8.8(-13.7 to -3.8) |
| **Monaco** | 947377(797467 to 1121656) | 1519(176 to 5619) | 12082(1397 to 44650) | 960977(808078 to 1133230) |  | -38.3(-42.6 to -34.3) | - | 97.5(-79.3 to 1995.6) | -37.7(-41.4 to -33.7) |  | -19.1(-23.9 to -14.1) | | 23.2(-76.7 to 548.5) | | -21.2(-85.1 to 315.1) | -19.1(-23.4 to -14.4) |
| **Mongolia** | 8097774(6133246 to 9873012) | 195421(38478 to 545781) | 891893(175586 to 2490921) | 9185089(7791656 to 10875950) |  | -19.6(-29.5 to -9.0) | - | 2848.0(436.2 to 30280.9) | -13.1(-21.6 to -3.5) |  | -19.6(-36.2 to -8.4) | | 96.4(-48.4 to 412.4) | | 22.0(-68.0 to 218.5) | -15.8(-22.2 to -8.6) |
| **Montenegro** | 568613(483096 to 672735) | 1167(193 to 3831) | 5327(880 to 17493) | 575108(490559 to 680057) |  | -26.1(-31.8 to -20.0) | - | 403.9(1.8 to 4843.2) | -25.1(-31.0 to -19.0) |  | -22.2(-27.5 to -16.6) | | -15.2(-85.5 to 226.6) | | -47.3(-91.0 to 102.8) | -22.6(-27.5 to -17.2) |
| **Morocco** | 7785452(6631648 to 9203888) | 5652(1625 to 13358) | 160152(46040 to 378726) | 7951256(6741341 to 9397465) |  | -39.9(-45.2 to -33.8) | - | 1051.7(125.8 to 13885.8) | -38.9(-43.9 to -32.6) |  | -20.1(-25.8 to -14.1) | | 49.7(-65.5 to 566.1) | | -1.4(-77.3 to 339.0) | -19.8(-25.2 to -14.7) |
| **Mozambique** | 29254968(24983742 to 34083120) | 10148(3292 to 21715) | 1572866(510311 to 3365399) | 30837982(26941734 to 35633472) |  | -27.2(-34.7 to -18.8) | - | 1021.2(207.1 to 8286.5) | -24.3(-32.0 to -15.5) |  | -15.7(-23.8 to -7.7) | | 61.2(-53.2 to 295.6) | | 7.2(-68.8 to 163.4) | -14.8(-21.2 to -7.8) |
| **Myanmar** | 17667233(15449022 to 19848767) | 98525(34764 to 205075) | 1080550(381219 to 2248727) | 18846308(16837956 to 21127603) |  | -49.6(-52.5 to -46.4) | - | 1576.3(246.7 to 18197.9) | -47.0(-50.1 to -43.7) |  | -29.0(-35.3 to -23.4) | | 32.1(-54.9 to 206.1) | | -14.5(-70.8 to 98.1) | -28.1(-31.9 to -23.7) |
| **Namibia** | 29339602(24945652 to 34600961) | 12062(4876 to 23360) | 1869582(756085 to 3621213) | 31221246(26802542 to 36565758) |  | -29.3(-36.8 to -20.6) | - | 5410.3(1015.3 to 50458.3) | -25.4(-32.7 to -16.8) |  | -18.7(-27.7 to -8.3) | | 41.6(-64.2 to 463.2) | | -5.8(-76.2 to 274.6) | -18.0(-24.7 to -10.6) |
| **Nauru** | 6222234(5427061 to 7074492) | 1662(171 to 7404) | 18231(1872 to 81225) | 6242127(5439983 to 7096646) |  | 1.2(-5.0 to 8.2) | - | 227.0(-79.0 to 4044.2) | 1.3(-4.9 to 8.2) |  | -20.4(-25.8 to -14.9) | | 43.2(-75.5 to 767.7) | | -7.4(-84.1 to 461.2) | -20.4(-25.7 to -14.8) |
| **Nepal** | 16004097(13663535 to 18594328) | 19380(4759 to 50853) | 790692(194096 to 2075658) | 16814168(14488127 to 19520046) |  | -57.5(-60.7 to -53.7) | - | 103.8(-42.3 to 1126.3) | -56.2(-59.6 to -52.3) |  | -27.4(-35.8 to -19.3) | | 41.0(-66.1 to 269.9) | | -6.8(-77.6 to 144.6) | -26.6(-34.4 to -18.9) |
| **Netherlands** | 451948(381894 to 537195) | 1131(433 to 2462) | 8995(3443 to 19584) | 462074(390885 to 548777) |  | -33.8(-38.5 to -28.9) | - | 185.4(-15.3 to 1448.8) | -32.6(-37.3 to -27.6) |  | -18.6(-24.1 to -12.1) | | 19.1(-55.9 to 193.1) | | -23.8(-71.7 to 87.6) | -18.6(-23.7 to -12.5) |
| **New Zealand** | 1077459(897924 to 1295421) | 2416(565 to 7156) | 19218(4490 to 56897) | 1099094(917108 to 1320683) |  | -53.1(-56.7 to -48.6) | - | 18.7(-66.8 to 603.6) | -52.4(-56.2 to -48.1) |  | 16.0(7.2 to 25.2) | | 63.7(-62.2 to 355.0) | | 4.7(-75.8 to 191.2) | 15.8(7.2 to 24.7) |
| **Nicaragua** | 2314473(2000815 to 2667882) | 3418(586 to 9749) | 43285(7414 to 123500) | 2361176(2046263 to 2710722) |  | -40.2(-45.2 to -35.2) | - | 243.7(-40.0 to 2683.1) | -39.3(-44.3 to -34.2) |  | -21.2(-26.6 to -15.9) | | 36.6(-68.6 to 312.6) | | -11.2(-79.6 to 168.0) | -21.0(-25.6 to -15.8) |
| **Niger** | 15779230(13640442 to 18036525) | 2491(353 to 7831) | 386149(54707 to 1213533) | 16167870(14156399 to 18539224) |  | -28.8(-36.2 to -21.9) | - | 443.7(-41.5 to 7993.3) | -27.0(-33.0 to -20.4) |  | -12.3(-19.2 to -4.2) | | 11.3(-73.1 to 421.6) | | -26.0(-82.1 to 247.0) | -12.7(-18.4 to -5.5) |
| **Nigeria** | 17529504(14943002 to 20126470) | 4953(1117 to 13530) | 767675(173187 to 2096748) | 18302132(15999778 to 20966855) |  | -28.0(-31.9 to -24.1) | - | 3484.2(693.9 to 37763.0) | -25.0(-28.9 to -21.2) |  | -24.1(-29.8 to -20.3) | | 16.0(-73.3 to 216.9) | | -22.8(-82.3 to 110.8) | -24.0(-26.5 to -21.5) |
| **Niue** | 3590235(3119715 to 4139463) | 881(99 to 3887) | 9665(1081 to 42630) | 3600782(3123392 to 4147612) |  | -22.8(-27.0 to -18.2) | - | 160.8(-80.1 to 2742.1) | -22.6(-26.9 to -18.1) |  | -8.9(-13.6 to -3.6) | | 55.3(-71.8 to 936.8) | | 0.5(-81.7 to 571.2) | -8.9(-13.5 to -3.6) |
| **North Macedonia** | 1028994(875611 to 1212961) | 3058(1000 to 7524) | 13958(4567 to 34344) | 1046010(893389 to 1229120) |  | -51.5(-55.6 to -47.0) | - | 425.9(21.9 to 4974.7) | -50.3(-54.5 to -45.7) |  | -19.8(-25.0 to -13.9) | | -25.8(-79.7 to 114.4) | | -53.9(-87.4 to 33.2) | -20.6(-25.2 to -15.5) |
| **Northern Mariana Islands** | 3680582(3159924 to 4273886) | 3585(520 to 12473) | 39320(5704 to 136753) | 3723488(3202858 to 4327223) |  | -61.2(-63.5 to -59.0) | - | 50.4(-78.6 to 2174.2) | -60.9(-62.9 to -58.5) |  | -14.7(-20.1 to -9.4) | | 18.9(-80.3 to 355.6) | | -23.1(-87.2 to 194.7) | -14.8(-19.9 to -9.7) |
| **Norway** | 516946(420481 to 626726) | 1737(460 to 4620) | 13816(3660 to 36749) | 532499(435031 to 642946) |  | -10.4(-20.1 to -0.8) | - | 90.5(-44.7 to 785.2) | -8.8(-18.4 to 0.8) |  | -37.3(-41.2 to -33.6) | | -12.2(-75.2 to 155.5) | | -43.8(-84.1 to 63.5) | -37.4(-40.4 to -34.3) |
| **Oman** | 1446634(1243713 to 1678380) | 981(209 to 2687) | 27782(5914 to 76140) | 1475397(1269768 to 1707387) |  | -49.7(-53.8 to -45.2) | - | 4.8(-71.2 to 512.5) | -49.3(-53.4 to -44.8) |  | -33.1(-37.4 to -29.1) | | 13.3(-77.3 to 279.8) | | -25.4(-85.0 to 150.3) | -33.0(-36.8 to -28.9) |
| **Pakistan** | 24244507(20460517 to 28357737) | 34529(10993 to 82666) | 1408796(448498 to 3372097) | 25687832(22137632 to 29598508) |  | -35.8(-40.2 to -31.1) | - | 5301.1(1056.7 to 55671.7) | -33.0(-37.1 to -28.3) |  | -22.4(-30.8 to -14.9) | | 57.9(-63.2 to 517.1) | | 4.4(-75.7 to 308.0) | -21.2(-26.1 to -16.0) |
| **Palau** | 3978445(3422153 to 4627217) | 1114(113 to 4864) | 12219(1234 to 53397) | 3991778(3424204 to 4641821) |  | -13.1(-17.9 to -8.6) | - | 205.7(-80.9 to 3515.2) | -13.0(-17.7 to -8.4) |  | -4.5(-9.1 to 1.3) | | 68.7(-72.2 to 1011.0) | | 9.1(-82.0 to 618.7) | -4.5(-9.1 to 1.3) |
| **Palestine** | 644015(547211 to 752426) | 493(61 to 2032) | 13964(1719 to 57597) | 658472(567651 to 763605) |  | -63.0(-66.2 to -59.6) | - | 262.4(-62.1 to 4235.1) | -62.4(-65.1 to -59.1) |  | -1.5(-9.1 to 5.9) | | 78.6(-65.3 to 856.5) | | 17.7(-77.2 to 530.2) | -1.1(-6.7 to 5.0) |
| **Panama** | 3159023(2703592 to 3638962) | 5906(734 to 22389) | 74785(9297 to 283594) | 3239714(2806796 to 3726975) |  | -29.5(-35.5 to -22.9) | - | 1027.8(23.3 to 11715.8) | -27.7(-33.1 to -21.7) |  | -9.4(-16.8 to -0.8) | | 28.2(-72.5 to 499.6) | | -16.7(-82.2 to 289.4) | -9.6(-16.1 to -2.7) |
| **Papua New Guinea** | 11732145(10474501 to 13042164) | 42501(15631 to 96508) | 466129(171513 to 1058778) | 12240775(11020506 to 13516242) |  | -30.2(-33.1 to -26.9) | - | 2250.0(384.8 to 23536.3) | -29.2(-32.3 to -25.9) |  | -14.2(-19.6 to -9.5) | | 306.6(-1.2 to 1466.0) | | 163.0(-36.1 to 913.3) | -11.7(-15.7 to -7.9) |
| **Paraguay** | 2937668(2475335 to 3464910) | 8042(938 to 26150) | 101832(11889 to 331095) | 3047542(2625474 to 3547272) |  | -19.7(-26.2 to -12.6) | - | 774.1(56.1 to 8204.5) | -17.9(-24.2 to -10.9) |  | -14.9(-23.0 to -7.2) | | 89.8(-70.3 to 481.4) | | 23.3(-80.7 to 278.0) | -13.9(-20.0 to -7.2) |
| **Peru** | 6884252(5889702 to 8064487) | 44045(22146 to 81375) | 557701(280426 to 1030615) | 7485997(6399950 to 8699093) |  | -65.3(-68.8 to -61.7) | - | 253.1(21.3 to 1487.6) | -62.1(-65.7 to -58.3) |  | -23.2(-30.4 to -16.1) | | -0.9(-49.5 to 83.0) | | -35.6(-67.2 to 18.9) | -24.2(-29.5 to -18.4) |
| **Philippines** | 31251320(27469562 to 35413279) | 82266(23225 to 194230) | 902227(254711 to 2130233) | 32235813(28631797 to 36420079) |  | -69.1(-70.9 to -67.2) | - | 1528.2(309.1 to 14581.0) | -68.1(-69.8 to -66.2) |  | 61.9(50.4 to 73.4) | | 143.4(-40.6 to 631.6) | | 57.5(-61.6 to 373.4) | 61.9(54.0 to 71.5) |
| **Poland** | 1190236(1000054 to 1435724) | 2104(788 to 4788) | 9602(3596 to 21856) | 1201942(1010893 to 1447437) |  | -44.6(-48.8 to -40.0) | - | 22.1(-60.9 to 453.0) | -44.2(-48.5 to -39.6) |  | -28.4(-31.3 to -25.2) | | 0.9(-65.6 to 161.0) | | -37.3(-78.6 to 62.2) | -28.5(-31.3 to -25.3) |
| **Portugal** | 1142638(968623 to 1338809) | 2118(474 to 5612) | 16848(3766 to 44640) | 1161605(993425 to 1361778) |  | -47.5(-51.5 to -43.3) | - | -22.7(-72.3 to 164.9) | -47.1(-51.2 to -42.9) |  | -23.9(-29.4 to -18.3) | | 9.1(-73.6 to 209.0) | | -30.2(-83.1 to 97.7) | -24.0(-28.9 to -19.0) |
| **Puerto Rico** | 266486(225814 to 315429) | 288(57 to 885) | 3653(728 to 11202) | 270427(228881 to 318274) |  | -52.3(-56.4 to -48.0) | - | 446.0(27.7 to 4475.3) | -51.3(-55.3 to -47.0) |  | -26.6(-32.3 to -21.0) | | -30.0(-83.8 to 122.0) | | -54.5(-89.5 to 44.2) | -27.3(-32.2 to -22.1) |
| **Qatar** | 1272094(1090729 to 1484422) | 923(184 to 3037) | 26150(5208 to 86023) | 1299167(1118917 to 1505836) |  | -50.8(-54.9 to -46.3) | - | 284.9(-25.1 to 4351.0) | -50.1(-54.2 to -45.8) |  | -31.4(-36.0 to -26.8) | | 40.6(-69.4 to 408.7) | | -7.4(-79.9 to 235.2) | -31.0(-34.8 to -26.9) |
| **Republic of Korea** | 3904686(3353354 to 4531361) | 7140(1054 to 28311) | 56795(8383 to 225139) | 3968621(3434450 to 4604424) |  | -56.7(-61.3 to -51.4) | - | 41.9(-73.0 to 894.2) | -56.1(-60.7 to -50.7) |  | -29.2(-34.1 to -24.3) | | -7.5(-83.1 to 217.4) | | -40.8(-89.2 to 103.2) | -29.4(-33.9 to -24.5) |
| **Republic of Moldova** | 3128844(2255654 to 4099808) | 482552(327349 to 670294) | 2202361(1493829 to 3059353) | 5813757(4976308 to 6930858) |  | -29.6(-37.3 to -20.7) | - | 2982.1(715.0 to 27420.8) | 10.8(-0.8 to 23.7) |  | -36.3(-52.0 to -19.9) | | 38.5(-7.2 to 93.5) | | -13.9(-42.4 to 20.2) | -25.6(-32.4 to -18.3) |
| **Romania** | 3753187(3213728 to 4421052) | 28342(11573 to 56362) | 129352(52800 to 257316) | 3910881(3343190 to 4583919) |  | -6.9(-16.5 to 3.7) | - | 279.5(5.1 to 1370.9) | -3.9(-12.7 to 6.0) |  | -16.5(-24.2 to -8.7) | | 30.3(-67.8 to 421.9) | | -19.0(-80.0 to 224.1) | -16.3(-22.3 to -9.9) |
| **Russian Federation** | 4890149(3712499 to 6317450) | 410823(215912 to 652224) | 1874987(985411 to 2976775) | 7175959(5902578 to 8786955) |  | -13.6(-28.3 to 0.9) | - | 3043.8(794.3 to 13577.5) | 24.2(13.9 to 36.2) |  | -41.4(-51.1 to -29.9) | | -9.2(-46.1 to 55.6) | | -43.6(-66.5 to -3.3) | -40.8(-45.1 to -36.1) |
| **Rwanda** | 20067889(17369613 to 23392695) | 3993(1554 to 8243) | 618917(240850 to 1277308) | 20690799(17879357 to 24165527) |  | -42.2(-47.9 to -36.2) | - | 3299.9(597.7 to 34579.0) | -40.9(-46.4 to -34.8) |  | -22.7(-28.4 to -16.5) | | 57.8(-60.3 to 576.4) | | 5.0(-73.6 to 350.0) | -22.1(-27.3 to -16.8) |
| **Saint Kitts and Nevis** | 2004863(1696276 to 2366563) | 873(86 to 3799) | 11058(1095 to 48103) | 2016794(1703115 to 2381006) |  | -39.1(-43.7 to -33.5) | - | -53.6(-96.6 to 393.4) | -39.1(-43.7 to -33.6) |  | -15.4(-21.7 to -8.9) | | 108.5(-62.6 to 1022.1) | | 35.4(-75.7 to 628.9) | -15.2(-21.3 to -8.9) |
| **Saint Lucia** | 1220139(1040836 to 1434410) | 441(53 to 1794) | 5587(668 to 22715) | 1226167(1050628 to 1440075) |  | -42.7(-47.3 to -37.9) | - | -77.0(-98.1 to 234.6) | -43.0(-47.5 to -38.3) |  | -17.2(-22.3 to -11.4) | | 67.9(-69.0 to 718.6) | | 9.1(-79.9 to 431.9) | -17.1(-22.1 to -11.6) |
| **Saint Vincent and the Grenadines** | 1527374(1309291 to 1790738) | 722(77 to 2698) | 9141(970 to 34167) | 1537238(1318423 to 1797645) |  | -32.6(-37.7 to -26.7) | - | -76.3(-97.9 to 342.2) | -33.1(-37.9 to -27.8) |  | -18.0(-23.1 to -12.1) | | 77.7(-66.8 to 826.9) | | 15.4(-78.4 to 501.8) | -17.9(-23.0 to -12.0) |
| **Samoa** | 5281984(4602836 to 5987463) | 315(34 to 1114) | 3459(375 to 12211) | 5285758(4606086 to 5988987) |  | -11.6(-15.8 to -7.1) | - | 154.4(-77.9 to 2869.1) | -11.5(-15.7 to -7.1) |  | -5.6(-10.4 to -0.5) | | -3.7(-82.2 to 471.8) | | -37.7(-88.5 to 269.8) | -5.6(-10.4 to -0.5) |
| **San Marino** | 408231(341831 to 484029) | 601(78 to 2134) | 4780(620 to 16971) | 413612(348170 to 493681) |  | -39.7(-43.7 to -35.9) | - | 78.4(-81.0 to 2294.0) | -39.2(-42.8 to -35.4) |  | -22.7(-27.4 to -17.7) | | 12.7(-80.2 to 421.6) | | -27.9(-87.3 to 233.7) | -22.8(-27.1 to -18.2) |
| **Sao Tome and Principe** | 7429445(6255772 to 8699198) | 1795(232 to 5532) | 278295(36020 to 857731) | 7709536(6639106 to 9012155) |  | -18.3(-27.0 to -9.8) | - | 716.9(-9.9 to 10350.8) | -15.8(-23.3 to -7.9) |  | -27.9(-33.9 to -21.4) | | 18.7(-75.6 to 425.8) | | -21.0(-83.8 to 249.8) | -27.7(-32.5 to -22.6) |
| **Saudi Arabia** | 4130576(3524417 to 4845364) | 4188(799 to 12328) | 118653(22653 to 349281) | 4253416(3655319 to 4980871) |  | -38.6(-45.3 to -30.8) | - | 1508.1(268.7 to 16408.3) | -37.0(-43.8 to -29.1) |  | -31.1(-36.4 to -26.1) | | 16.9(-76.0 to 254.2) | | -23.0(-84.2 to 133.6) | -30.9(-34.7 to -26.6) |
| **Senegal** | 15446430(13417167 to 17935661) | 2277(695 to 5030) | 352933(107683 to 779775) | 15801640(13777994 to 18264995) |  | -27.0(-32.8 to -20.9) | - | 2106.3(298.5 to 25228.8) | -25.4(-31.2 to -19.3) |  | -16.8(-22.8 to -9.7) | | 26.1(-74.3 to 452.6) | | -16.1(-82.9 to 267.5) | -16.8(-22.4 to -10.3) |
| **Serbia** | 792311(676754 to 932762) | 1515(420 to 4006) | 6913(1914 to 18280) | 800738(685363 to 940323) |  | -37.0(-42.3 to -31.4) | - | 203.7(-25.6 to 2566.9) | -36.4(-41.6 to -30.6) |  | -26.8(-31.5 to -21.5) | | -8.0(-75.3 to 152.2) | | -42.8(-84.7 to 56.7) | -27.0(-31.7 to -21.4) |
| **Seychelles** | 2798537(2435989 to 3215458) | 575(40 to 2761) | 6308(434 to 30278) | 2805421(2438436 to 3225439) |  | -7.4(-13.0 to -1.8) | - | 568.3(-56.3 to 9021.8) | -7.1(-12.6 to -1.6) |  | -8.6(-13.5 to -3.4) | | -9.6(-89.0 to 453.6) | | -41.5(-92.9 to 258.3) | -8.7(-13.7 to -3.7) |
| **Sierra Leone** | 23400443(20293550 to 27023736) | 3419(479 to 10870) | 530021(74259 to 1685517) | 23933884(20913975 to 27445060) |  | -5.9(-14.7 to 2.6) | - | 263.7(-57.9 to 2908.8) | -4.3(-12.0 to 3.4) |  | -19.2(-25.6 to -12.1) | | 20.3(-72.3 to 394.4) | | -20.0(-81.5 to 228.8) | -19.2(-25.1 to -12.8) |
| **Singapore** | 2913007(2520998 to 3389720) | 4141(1777 to 8387) | 32938(14146 to 66686) | 2950087(2548720 to 3431953) |  | -50.1(-54.9 to -44.5) | - | 62.8(-52.4 to 773.8) | -49.6(-54.4 to -44.0) |  | -7.9(-13.8 to -1.7) | | 21.5(-51.3 to 165.0) | | -22.3(-68.9 to 69.5) | -8.1(-13.7 to -2.0) |
| **Slovakia** | 409320(348799 to 482519) | 643(130 to 2021) | 2934(591 to 9220) | 412897(351081 to 488166) |  | -48.2(-52.2 to -44.0) | - | 23.6(-62.5 to 616.2) | -47.7(-51.8 to -43.8) |  | -28.6(-32.8 to -24.3) | | -30.7(-84.9 to 116.8) | | -57.0(-90.6 to 34.7) | -28.9(-32.9 to -24.5) |
| **Slovenia** | 445107(385294 to 522965) | 107(15 to 392) | 489(69 to 1791) | 445703(385477 to 523311) |  | -50.5(-54.4 to -46.1) | - | -52.8(-91.2 to 260.3) | -50.5(-54.3 to -46.1) |  | -35.9(-40.0 to -31.7) | | -71.2(-94.7 to 2.7) | | -82.1(-96.7 to -36.1) | -36.1(-40.2 to -32.0) |
| **Solomon Islands** | 4417258(3912605 to 4924178) | 1248(89 to 4912) | 13683(972 to 53828) | 4432189(3916254 to 4940949) |  | -10.4(-14.1 to -6.3) | - | 219.1(-76.5 to 4725.0) | -10.2(-14.0 to -6.1) |  | -7.5(-12.6 to -2.0) | | 63.9(-76.5 to 922.1) | | 6.0(-84.8 to 561.5) | -7.5(-12.2 to -2.1) |
| **Somalia** | 42553817(34688020 to 51135975) | 19625(5602 to 56469) | 3041984(868423 to 8750946) | 45615426(38922672 to 54213192) |  | -5.2(-11.5 to 1.4) | - | 14269.8(3531.9 to 141010.9) | 1.6(-5.0 to 8.5) |  | -14.3(-25.7 to -6.0) | | 27.3(-66.6 to 266.7) | | -15.3(-77.8 to 144.0) | -14.3(-20.1 to -8.0) |
| **South Africa** | 36237323(30892179 to 42282008) | 6440(2272 to 14998) | 998280(352039 to 2324905) | 37242044(31789483 to 43356265) |  | -17.2(-22.3 to -10.5) | - | 498.5(73.8 to 3444.2) | -14.9(-19.7 to -8.0) |  | 3.8(-4.7 to 11.8) | | 36.3(-65.4 to 368.7) | | -9.3(-77.0 to 211.8) | 3.4(-3.3 to 10.3) |
| **South Sudan** | 26368390(22528058 to 30584543) | 6998(1098 to 21673) | 1084680(170241 to 3359882) | 27460068(24046571 to 31475045) |  | -16.9(-24.8 to -9.6) | - | 3285.7(314.1 to 41836.4) | -14.2(-20.9 to -7.4) |  | -16.2(-23.3 to -8.8) | | 59.2(-62.9 to 615.6) | | 5.9(-75.3 to 375.8) | -15.5(-21.3 to -9.8) |
| **Spain** | 667999(564250 to 787097) | 1022(134 to 3792) | 8125(1068 to 30155) | 677146(573237 to 797351) |  | -56.4(-59.8 to -52.3) | - | -9.0(-84.3 to 504.1) | -56.0(-59.1 to -52.1) |  | -23.2(-28.7 to -17.6) | | -7.9(-80.3 to 220.7) | | -41.1(-87.4 to 105.1) | -23.5(-28.7 to -18.2) |
| **Sri Lanka** | 3803868(3266932 to 4430295) | 1302(128 to 4996) | 14275(1404 to 54770) | 3819444(3283051 to 4450090) |  | -3.8(-9.8 to 2.6) | - | 704.4(-24.5 to 11167.3) | -3.4(-9.4 to 2.9) |  | -5.9(-11.1 to -0.2) | | 8.3(-80.5 to 461.9) | | -29.9(-87.4 to 263.3) | -6.0(-11.1 to -0.5) |
| **Sudan** | 3082505(2638110 to 3573921) | 2269(260 to 9311) | 64284(7367 to 263705) | 3149058(2720226 to 3641747) |  | -37.1(-42.8 to -30.7) | - | 565.7(-37.7 to 8490.2) | -35.9(-41.4 to -29.8) |  | -25.8(-31.5 to -19.5) | | 18.0(-74.6 to 514.0) | | -22.3(-83.3 to 304.7) | -25.7(-30.3 to -20.3) |
| **Suriname** | 1084396(921951 to 1267384) | 336(41 to 1189) | 4252(524 to 15048) | 1088984(929033 to 1271572) |  | -30.1(-35.5 to -24.1) | - | -42.9(-95.1 to 528.9) | -30.1(-35.4 to -24.4) |  | -13.2(-19.1 to -6.3) | | 73.4(-69.9 to 887.0) | | 12.7(-80.4 to 540.8) | -13.1(-19.0 to -6.5) |
| **Sweden** | 844239(682841 to 1024182) | 3443(1200 to 7561) | 27388(9536 to 60121) | 875070(711693 to 1063781) |  | -2.8(-10.3 to 4.9) | - | 437.8(60.1 to 2879.3) | 0.4(-7.2 to 8.3) |  | -0.3(-8.7 to 6.9) | | 36.8(-54.9 to 222.4) | | -12.5(-71.1 to 106.2) | -0.6(-7.2 to 6.0) |
| **Switzerland** | 564457(469829 to 671223) | 3006(1200 to 6193) | 23912(9537 to 49234) | 591375(493052 to 705478) |  | -39.9(-44.2 to -35.2) | - | 150.5(-33.8 to 1477.5) | -38.8(-43.2 to -34.2) |  | -10.4(-18.3 to -2.5) | | 160.4(-7.5 to 503.2) | | 66.6(-40.8 to 285.7) | -8.4(-15.8 to -0.5) |
| **Syrian Arab Republic** | 914906(773667 to 1081694) | 877(122 to 3565) | 24849(3455 to 101044) | 940632(805788 to 1103764) |  | -53.7(-58.4 to -48.8) | - | 601.0(-7.5 to 9810.7) | -52.3(-56.1 to -47.6) |  | -17.8(-24.5 to -11.2) | | 8.0(-76.8 to 309.8) | | -28.8(-84.8 to 170.0) | -18.1(-23.1 to -13.0) |
| **Taiwan (Province of China)** | 3883233(3351093 to 4459475) | 7596(872 to 27792) | 83307(9563 to 304912) | 3974136(3468698 to 4577079) |  | -35.5(-41.7 to -29.1) | - | 88.1(-82.8 to 2439.6) | -34.4(-39.7 to -28.4) |  | -8.2(-17.5 to 3.1) | | 29.6(-74.4 to 586.9) | | -16.2(-83.4 to 344.8) | -8.3(-16.5 to 2.0) |
| **Tajikistan** | 4424313(3510656 to 5397653) | 166738(76884 to 300123) | 760990(351068 to 1369512) | 5352041(4560997 to 6239739) |  | -1.4(-14.1 to 9.9) | - | 23930.0(4908.9 to 222383.6) | 15.8(6.4 to 25.7) |  | -17.0(-33.5 to 0.8) | | 49.7(-51.6 to 355.7) | | -7.0(-69.9 to 183.3) | -14.5(-20.3 to -7.8) |
| **Thailand** | 11073898(9504424 to 12896600) | 25064(6969 to 62292) | 274889(76419 to 683072) | 11373851(9793315 to 13292684) |  | -53.7(-56.6 to -50.6) | - | 93.9(-44.3 to 1164.5) | -52.7(-55.7 to -49.6) |  | -10.7(-16.8 to -4.5) | | 31.6(-67.6 to 267.7) | | -14.8(-79.0 to 137.8) | -10.7(-15.9 to -5.0) |
| **Timor-Leste** | 12493700(11118268 to 13963843) | 5549(581 to 22754) | 60858(6370 to 249640) | 12560107(11165335 to 14044992) |  | -22.4(-26.7 to -18.0) | - | 323.7(-63.1 to 5723.3) | -21.8(-25.6 to -17.7) |  | 3.1(-2.1 to 9.4) | | -18.5(-85.1 to 343.4) | | -47.3(-90.3 to 186.7) | 2.6(-2.3 to 7.8) |
| **Togo** | 17414831(14992568 to 20296310) | 2811(430 to 7950) | 435760(66686 to 1232822) | 17853403(15525812 to 20670851) |  | -19.3(-27.1 to -11.7) | - | 475.1(-23.9 to 7620.7) | -17.3(-24.6 to -10.3) |  | -19.2(-25.6 to -11.6) | | 6.4(-75.7 to 382.7) | | -29.2(-83.9 to 221.0) | -19.5(-25.1 to -13.5) |
| **Tokelau** | 3944420(3435496 to 4493142) | 1116(89 to 5618) | 12244(974 to 61594) | 3957780(3445981 to 4512247) |  | -28.9(-33.3 to -24.8) | - | 147.0(-79.2 to 3822.4) | -28.7(-33.2 to -24.7) |  | -14.6(-19.7 to -9.6) | | 57.4(-72.7 to 738.5) | | 1.8(-82.4 to 442.3) | -14.6(-19.5 to -9.7) |
| **Tonga** | 3272329(2859441 to 3703609) | 880(84 to 3657) | 9649(918 to 40137) | 3282858(2870668 to 3712622) |  | -11.1(-15.5 to -6.3) | - | 176.6(-76.9 to 3869.9) | -10.9(-15.5 to -6.2) |  | -8.9(-13.7 to -3.9) | | 69.9(-71.9 to 769.3) | | 9.9(-81.8 to 462.0) | -8.9(-13.6 to -4.0) |
| **Trinidad and Tobago** | 978668(846411 to 1132674) | 435(51 to 1845) | 5512(641 to 23369) | 984615(850310 to 1140117) |  | -33.3(-38.3 to -27.3) | - | -43.1(-94.4 to 527.5) | -33.3(-38.3 to -27.4) |  | -15.1(-21.8 to -8.7) | | 78.8(-66.2 to 902.1) | | 16.1(-78.0 to 551.0) | -15.0(-21.3 to -8.6) |
| **Tunisia** | 1384387(1183839 to 1610903) | 637(191 to 1533) | 18046(5412 to 43456) | 1403070(1201056 to 1626463) |  | -43.5(-47.8 to -38.9) | - | 723.6(25.2 to 9041.3) | -42.9(-47.3 to -38.3) |  | -25.1(-29.4 to -20.3) | | 28.4(-68.3 to 449.1) | | -15.4(-79.1 to 261.7) | -24.9(-29.1 to -20.4) |
| **Turkey** | 1628834(1404157 to 1909193) | 2273(1015 to 4490) | 64394(28746 to 127184) | 1695501(1456573 to 1972896) |  | -54.8(-59.3 to -50.0) | - | 1585.3(250.4 to 14901.9) | -53.2(-57.9 to -48.4) |  | -29.8(-35.0 to -24.6) | | 19.3(-55.3 to 203.0) | | -21.4(-70.5 to 99.6) | -29.5(-33.5 to -24.9) |
| **Turkmenistan** | 3790260(2855854 to 4762429) | 168339(61878 to 334750) | 768293(282443 to 1527737) | 4726891(3976495 to 5641891) |  | -26.5(-36.7 to -16.8) | - | 14738.7(3274.4 to 149396.9) | -12.8(-20.4 to -4.2) |  | -25.4(-44.1 to -7.8) | | 46.9(-57.4 to 337.4) | | -8.7(-73.5 to 171.7) | -21.7(-27.5 to -15.3) |
| **Tuvalu** | 5431885(4834482 to 6119929) | 1520(124 to 7000) | 16666(1360 to 76724) | 5450071(4841568 to 6137102) |  | -28.2(-31.9 to -24.2) | - | 145.4(-83.4 to 3670.8) | -28.1(-31.7 to -24.0) |  | -11.1(-15.8 to -6.7) | | 60.0(-71.9 to 935.6) | | 3.6(-81.8 to 569.7) | -11.1(-15.6 to -6.7) |
| **Uganda** | 20292327(18204800 to 22478957) | 4431(921 to 11482) | 686775(142833 to 1780760) | 20983533(18830424 to 23228292) |  | -8.6(-15.7 to -0.9) | - | 5186.9(1136.7 to 57598.6) | -6.5(-13.9 to 1.4) |  | -5.4(-13.5 to 2.8) | | 113.9(-55.9 to 497.3) | | 42.3(-70.7 to 297.8) | -4.3(-12.1 to 3.4) |
| **Ukraine** | 4995961(3515619 to 6652740) | 504894(269413 to 792526) | 2304317(1229651 to 3617332) | 7805173(6538021 to 9447391) |  | -13.0(-30.0 to 1.8) | - | 3235.3(731.1 to 27590.2) | 16.0(5.8 to 27.6) |  | -17.8(-43.2 to 14.8) | | 101.2(-16.3 to 391.7) | | 25.0(-48.0 to 205.4) | -4.5(-12.5 to 4.5) |
| **United Arab Emirates** | 2142212(1833492 to 2496568) | 1570(171 to 5848) | 44476(4844 to 165777) | 2188258(1892737 to 2536192) |  | -32.5(-38.6 to -26.2) | - | 488.5(-38.1 to 6755.7) | -31.4(-37.3 to -25.5) |  | -25.0(-30.8 to -18.5) | | 39.3(-75.4 to 614.2) | | -8.2(-83.8 to 370.5) | -24.6(-29.9 to -19.2) |
| **United Kingdom** | 964726(790105 to 1173755) | 1499(536 to 3333) | 11926(4267 to 26508) | 978151(803117 to 1193967) |  | -4.4(-10.7 to 2.8) | - | 41.3(-51.4 to 343.8) | -3.9(-10.2 to 3.5) |  | -42.0(-44.3 to -39.7) | | -13.1(-67.8 to 104.1) | | -44.4(-79.4 to 30.6) | -42.0(-44.2 to -39.9) |
| **United Republic of Tanzania** | 23995718(20396169 to 28009980) | 3967(698 to 13472) | 614937(108217 to 2088577) | 24614622(21260860 to 28550717) |  | -36.4(-41.9 to -30.1) | - | 1884.3(248.1 to 28268.5) | -35.5(-41.2 to -29.1) |  | -17.3(-24.1 to -10.3) | | 116.6(-48.2 to 508.4) | | 44.1(-65.5 to 304.8) | -16.4(-22.0 to -10.2) |
| **United States of America** | 208720(177907 to 245420) | 369(141 to 803) | 2938(1120 to 6384) | 212027(181204 to 249415) |  | -60.0(-63.4 to -56.8) | - | -83.5(-90.8 to -69.3) | -60.7(-64.1 to -57.6) |  | -29.0(-31.5 to -26.4) | | 17.2(-55.4 to 156.9) | | -25.0(-71.5 to 64.4) | -28.9(-31.1 to -26.5) |
| **United States Virgin Islands** | 555578(471056 to 652037) | 211(25 to 770) | 2672(319 to 9752) | 558461(474424 to 654380) |  | -34.6(-40.0 to -28.3) | - | -51.5(-95.8 to 493.0) | -34.7(-40.0 to -28.7) |  | -16.9(-22.3 to -11.5) | | 76.1(-66.5 to 871.4) | | 14.4(-78.2 to 531.0) | -16.8(-22.1 to -11.4) |
| **Uruguay** | 1753439(1494177 to 2078299) | 707(154 to 2223) | 5627(1223 to 17676) | 1759773(1497274 to 2085003) |  | -38.2(-43.1 to -32.9) | - | 68.9(-73.9 to 1505.0) | -38.1(-43.1 to -32.8) |  | 9.9(2.3 to 18.3) | | 97.6(-61.5 to 727.8) | | 26.5(-75.4 to 430.2) | 10.0(2.4 to 18.3) |
| **Uzbekistan** | 3894978(2615666 to 5023065) | 288456(108012 to 543341) | 1316521(492808 to 2480097) | 5499955(4773474 to 6354894) |  | -37.5(-44.4 to -30.2) | - | 32048.5(7804.5 to 360959.2) | -8.8(-16.9 to -0.5) |  | -23.2(-47.6 to -1.4) | | 3.3(-63.9 to 106.5) | | -35.8(-77.6 to 28.3) | -25.7(-31.8 to -18.9) |
| **Vanuatu** | 6640170(5942855 to 7308324) | 1107(93 to 4842) | 12138(1014 to 53109) | 6653414(5963088 to 7310323) |  | -13.6(-17.8 to -9.2) | - | 219.6(-79.9 to 3341.4) | -13.5(-17.6 to -9.1) |  | -3.0(-7.5 to 1.7) | | 125.1(-60.4 to 1397.1) | | 45.7(-74.4 to 868.0) | -2.9(-7.4 to 1.8) |
| **Venezuela (Bolivarian Republic of)** | 2287262(1959526 to 2634938) | 3468(418 to 11622) | 43911(5296 to 147167) | 2334641(2011034 to 2681741) |  | -45.6(-50.8 to -40.2) | - | 598.2(-23.8 to 9299.7) | -44.5(-49.3 to -39.3) |  | 8.6(0.1 to 17.2) | | 57.7(-68.5 to 623.9) | | 2.4(-79.6 to 370.4) | 8.5(1.1 to 15.9) |
| **Viet Nam** | 14666008(12941672 to 16214852) | 54945(16192 to 131488) | 602596(177584 to 1442367) | 15323549(13678435 to 16838486) |  | -16.1(-20.3 to -11.8) | - | 808.3(150.2 to 4952.1) | -12.3(-16.5 to -8.2) |  | -17.4(-23.8 to -11.9) | | 11.3(-71.3 to 207.4) | | -28.0(-81.4 to 98.8) | -17.8(-21.9 to -13.3) |
| **Yemen** | 3347208(2896414 to 3857175) | 2569(538 to 9123) | 72791(15231 to 258476) | 3422568(2970881 to 3942622) |  | -36.9(-41.9 to -30.8) | - | 889.2(99.4 to 11956.6) | -35.4(-40.5 to -29.6) |  | -13.5(-19.8 to -7.3) | | 20.3(-72.9 to 312.0) | | -20.7(-82.2 to 171.2) | -13.7(-18.8 to -8.2) |
| **Zambia** | 23859013(20264898 to 27958546) | 6010(962 to 20844) | 931495(149052 to 3231169) | 24796518(21308389 to 28910731) |  | -27.2(-35.0 to -19.4) | - | 670.1(57.2 to 6863.5) | -26.1(-33.8 to -18.3) |  | -24.4(-33.8 to -15.2) | | 167.5(-45.4 to 711.4) | | 78.0(-63.7 to 439.9) | -22.7(-30.1 to -14.1) |
| **Zimbabwe** | 24172283(19900494 to 28893414) | 8409(1223 to 27984) | 1303366(189479 to 4337224) | 25484058(21708829 to 30144216) |  | -15.2(-29.9 to -0.3) | - | 1224.5(26.2 to 17548.4) | -11.4(-24.1 to 2.4) |  | -18.0(-29.9 to -3.8) | | 38.3(-66.6 to 564.1) | | -8.0(-77.8 to 341.8) | -17.6(-27.7 to -5.8) |

| **S5** | | | | | | | | | | | | | | | |
| --- | --- | --- | --- | --- | --- | --- | --- | --- | --- | --- | --- | --- | --- | --- | --- |
| **Group** | **Number of DALYs (age-standardized, both sexes) with 95% uncertainty intervals (2019)** | | | |  | **Annualized rate of change of age-standardized rate (%) with 95% uncertainty intervals** | | | | | | | | | |
|  |  |  |  |  |  | **1990-2010** | | | |  | **2010-2019** | | | | |
|  | **Drug-susceptible tuberculosis** | **Extensively drug-resistant tuberculosis** | **Multidrug-resistant tuberculosis without extensive drug resistance** | **Tuberculosis** |  | **Drug-susceptible tuberculosis** | **Extensively drug-resistant tuberculosis** | **Multidrug-resistant tuberculosis without extensive drug resistance** | **Tuberculosis** |  | **Drug-susceptible tuberculosis** | **Extensively drug-resistant tuberculosis** | **Multidrug-resistant tuberculosis without extensive drug resistance** | | **Tuberculosis** |
| **Global** | 53422865(46676267 to 59669580) | 381509(189136 to 668316) | 5238076(2263595 to 9759929) | 59042451(53684783 to 64641528) |  | -51.6(-55.9 to -47.3) | - | 386.5(137.5 to 1006.8) | -47.5(-50.6 to -43.4) |  | -29.8(-35.9 to -23.3) | -8.2(-33.7 to 31.8) | | -22.7(-51.5 to 21.3) | -29.1(-34.5 to -23.6) |
| **Afghanistan** | 62862683(38358556 to 88141570) | 455337(61041 to 1557118) | 6202654(934827 to 20027268) | 69520674(45434465 to 93948864) |  | -54.1(-64.5 to -39.7) | - | 2323.4(420.5 to 29487.8) | -47.1(-57.5 to -32.2) |  | -34.3(-50.6 to -17.8) | -33.3(-87.1 to 114.7) | | -56.0(-91.4 to 42.4) | -37.1(-47.0 to -23.7) |
| **Albania** | 889097(686951 to 1126081) | 4942(800 to 14618) | 11841(1980 to 35506) | 905880(700518 to 1144859) |  | -83.1(-86.0 to -79.7) | - | 1.1(-78.1 to 803.6) | -82.6(-85.5 to -79.1) |  | -16.9(-33.5 to 2.0) | -34.9(-86.7 to 110.7) | | -59.5(-91.7 to 29.5) | -18.2(-34.7 to 0.2) |
| **Algeria** | 4107316(3211329 to 5039830) | 12137(945 to 56435) | 174129(14558 to 827644) | 4293582(3560347 to 5178676) |  | -71.7(-77.7 to -64.5) | - | -64.5(-95.1 to 87.3) | -71.5(-77.0 to -64.5) |  | -37.0(-48.5 to -23.6) | 18.6(-78.5 to 304.8) | | -21.3(-85.6 to 168.0) | -36.4(-46.8 to -22.8) |
| **American Samoa** | 5751150(4793672 to 6906769) | 5723(475 to 24274) | 32920(2799 to 145267) | 5789793(4834885 to 6945084) |  | -50.5(-57.6 to -42.0) | - | 40.2(-90.6 to 1815.2) | -50.3(-57.5 to -41.8) |  | -16.0(-26.1 to -3.8) | 48.5(-73.6 to 789.7) | | -2.8(-83.0 to 475.0) | -15.9(-25.8 to -4.2) |
| **Andorra** | 500446(402077 to 618530) | 532(53 to 2368) | 2187(214 to 10743) | 503166(405403 to 622654) |  | -58.8(-69.7 to -46.4) | - | -42.2(-95.0 to 522.1) | -58.7(-69.7 to -46.5) |  | -15.0(-28.1 to 1.8) | 0.2(-84.6 to 482.7) | | -35.9(-90.2 to 271.4) | -15.1(-27.9 to 1.8) |
| **Angola** | 251504437(188570314 to 319699346) | 221445(21462 to 824670) | 16527360(1644784 to 55830598) | 268253242(210742266 to 333908464) |  | -48.6(-62.2 to -30.8) | - | 383.1(-49.8 to 5940.8) | -45.8(-58.2 to -27.4) |  | -43.2(-55.4 to -29.1) | -8.2(-79.7 to 319.0) | | -38.6(-86.5 to 181.6) | -42.9(-53.4 to -30.2) |
| **Antigua and Barbuda** | 2176431(1756469 to 2711004) | 3103(273 to 14472) | 22459(2009 to 101265) | 2201993(1774893 to 2730983) |  | -49.9(-56.4 to -42.1) | - | -75.4(-97.7 to 177.7) | -50.2(-56.5 to -43.2) |  | -29.0(-38.2 to -17.1) | 59.6(-70.1 to 939.1) | | 2.3(-80.8 to 573.8) | -28.7(-37.9 to -17.3) |
| **Argentina** | 5165345(4514233 to 5738465) | 37257(4400 to 138722) | 145809(16967 to 545126) | 5348411(4868389 to 5859155) |  | -68.2(-71.1 to -65.5) | - | 55.4(-67.6 to 1107.6) | -67.0(-69.2 to -64.6) |  | -17.4(-26.3 to -7.6) | -7.9(-81.1 to 169.5) | | -40.6(-87.9 to 73.1) | -18.2(-25.4 to -10.2) |
| **Armenia** | 6233463(3373021 to 8743622) | 792191(180710 to 1676339) | 1836043(444146 to 3912399) | 8861696(7464275 to 10402013) |  | -30.0(-49.9 to -12.7) | - | 11753.7(2633.3 to 146021.4) | 0.6(-7.6 to 9.9) |  | -49.6(-68.1 to -31.8) | -32.0(-77.6 to 31.4) | | -57.0(-85.9 to -16.2) | -50.2(-57.2 to -42.9) |
| **Australia** | 497037(393350 to 618039) | 7278(2265 to 17078) | 29886(9665 to 67513) | 534201(431538 to 656222) |  | -55.9(-61.3 to -50.2) | - | 71.6(-62.0 to 936.6) | -54.6(-59.6 to -49.0) |  | -12.5(-26.5 to 2.3) | 136.8(-32.6 to 696.8) | | 51.9(-56.7 to 414.3) | -9.6(-21.5 to 4.6) |
| **Austria** | 782828(665813 to 900965) | 11343(3450 to 27117) | 46392(14521 to 108762) | 840563(746825 to 952810) |  | -78.1(-80.7 to -75.2) | - | 69.4(-54.3 to 1412.4) | -76.3(-78.7 to -73.5) |  | -21.0(-31.3 to -11.1) | -12.1(-66.5 to 88.6) | | -43.6(-78.3 to 21.2) | -22.6(-30.4 to -14.6) |
| **Azerbaijan** | 18312102(10547513 to 26477827) | 2952352(892438 to 5681988) | 6657078(2135424 to 12509426) | 27921532(22795300 to 34072138) |  | -57.1(-70.3 to -45.1) | - | 10364.3(2375.7 to 118099.5) | -39.4(-47.5 to -30.2) |  | -39.6(-65.2 to -4.6) | 8.7(-66.3 to 170.5) | | -32.3(-79.0 to 70.3) | -34.9(-45.3 to -22.9) |
| **Bahamas** | 7237593(5740626 to 8897865) | 36238(4190 to 138932) | 226594(26106 to 897306) | 7500424(6028466 to 9229426) |  | -60.4(-65.8 to -54.5) | - | 351.2(-21.4 to 4679.1) | -59.5(-64.8 to -53.5) |  | -17.3(-33.8 to 0.7) | 78.9(-73.5 to 753.0) | | 16.5(-82.8 to 458.1) | -16.3(-30.6 to 1.5) |
| **Bahrain** | 3829038(3043198 to 4660706) | 15819(2132 to 52811) | 226597(32698 to 697513) | 4071454(3355251 to 4855552) |  | -65.3(-70.9 to -58.9) | - | 632.9(61.7 to 8237.2) | -63.3(-68.4 to -56.6) |  | -39.0(-50.3 to -27.2) | -6.4(-86.4 to 241.9) | | -37.7(-90.9 to 128.4) | -38.9(-48.2 to -29.2) |
| **Bangladesh** | 63329933(47622756 to 85503574) | 260238(53381 to 748048) | 5152972(1109561 to 13379518) | 68743143(54191135 to 91797762) |  | -73.4(-77.8 to -66.5) | - | 1282.3(173.6 to 15038.5) | -71.6(-76.0 to -64.3) |  | -47.8(-57.6 to -36.5) | -2.1(-74.1 to 160.3) | | -34.8(-82.8 to 73.5) | -46.9(-55.4 to -36.4) |
| **Barbados** | 1847658(1507510 to 2254903) | 1760(109 to 8765) | 11619(768 to 56766) | 1861037(1527276 to 2271844) |  | -55.9(-60.9 to -49.6) | - | -90.4(-99.3 to 75.7) | -56.5(-61.1 to -51.1) |  | -20.1(-32.5 to -5.2) | 85.9(-77.3 to 1261.0) | | 18.8(-85.3 to 778.0) | -19.9(-32.3 to -5.1) |
| **Belarus** | 4024050(2139940 to 6452944) | 1767974(1006108 to 2667938) | 4125127(2516270 to 5960287) | 9917150(7681783 to 12612748) |  | -18.0(-45.0 to 11.0) | - | 3158.3(596.4 to 29835.8) | 72.4(59.5 to 86.8) |  | -63.6(-77.1 to -46.8) | -35.0(-55.6 to -8.5) | | -58.7(-71.9 to -42.4) | -58.2(-67.4 to -47.4) |
| **Belgium** | 872819(757567 to 991706) | 7001(1829 to 19798) | 28470(7732 to 78122) | 908290(805329 to 1022229) |  | -54.0(-58.0 to -48.9) | - | 2.4(-69.0 to 489.1) | -52.6(-56.2 to -48.2) |  | -31.9(-39.4 to -24.3) | -19.3(-73.3 to 103.4) | | -47.8(-82.6 to 32.4) | -32.5(-38.8 to -25.9) |
| **Belize** | 17144992(14692267 to 19959733) | 27147(2553 to 112851) | 168399(16571 to 705973) | 17340539(14838694 to 20070980) |  | -48.6(-54.6 to -41.3) | - | -65.2(-96.5 to 288.2) | -48.8(-54.6 to -41.5) |  | -22.8(-33.8 to -9.7) | 79.8(-66.7 to 917.4) | | 17.3(-78.4 to 563.8) | -22.4(-33.5 to -9.4) |
| **Benin** | 130516681(95678969 to 176101559) | 76686(10650 to 251845) | 5701299(829302 to 18387221) | 136294666(100388430 to 182559886) |  | -45.7(-58.2 to -30.4) | - | 388.5(12.5 to 3913.4) | -44.0(-56.0 to -28.7) |  | -24.9(-39.3 to -9.4) | 42.3(-74.1 to 328.3) | | -5.7(-82.7 to 185.4) | -24.2(-38.0 to -8.4) |
| **Bermuda** | 1159850(796642 to 1620762) | 716(39 to 4087) | 7134(382 to 40566) | 1167700(809552 to 1624520) |  | -47.3(-56.4 to -38.2) | - | -79.3(-98.8 to 169.3) | -47.5(-56.4 to -38.6) |  | -31.0(-42.3 to -18.2) | 140.4(-74.1 to 1620.8) | | 58.2(-83.2 to 1031.7) | -30.8(-41.8 to -18.2) |
| **Bhutan** | 50717226(31309466 to 93322321) | 236477(26085 to 829406) | 4651658(490357 to 15487875) | 55605361(36069867 to 98416367) |  | -74.8(-83.2 to -60.3) | - | 200.5(-68.7 to 4600.3) | -72.7(-80.8 to -57.9) |  | -36.8(-52.4 to -19.6) | -3.4(-75.4 to 304.1) | | -36.0(-83.7 to 168.7) | -36.6(-48.2 to -22.4) |
| **Bolivia (Plurinational State of)** | 49504755(33010207 to 65550667) | 819645(122858 to 2752727) | 5063528(727520 to 16018333) | 55387927(40637447 to 70870953) |  | -78.7(-84.5 to -72.2) | - | -2.8(-88.5 to 920.9) | -77.5(-82.3 to -71.4) |  | -41.3(-56.0 to -24.4) | 38.5(-60.2 to 507.7) | | -8.7(-74.1 to 296.9) | -38.8(-50.9 to -24.5) |
| **Bosnia and Herzegovina** | 5409203(4305300 to 6735469) | 20956(4153 to 64375) | 47450(9953 to 142449) | 5477608(4348943 to 6803095) |  | -67.4(-70.7 to -63.8) | - | -35.9(-84.5 to 326.6) | -67.1(-70.2 to -63.5) |  | -30.3(-43.9 to -12.4) | -19.7(-78.7 to 124.3) | | -49.8(-86.6 to 40.6) | -30.5(-44.0 to -13.2) |
| **Botswana** | 162693689(110104220 to 224643693) | 228382(29456 to 716219) | 17133235(2595868 to 52026322) | 180055306(129921821 to 246355886) |  | -26.6(-48.5 to 2.6) | - | 921.6(153.3 to 8365.9) | -21.8(-44.8 to 8.5) |  | -39.7(-55.7 to -21.3) | 33.5(-67.9 to 251.1) | | -10.4(-78.6 to 133.9) | -37.7(-51.1 to -20.9) |
| **Brazil** | 8476187(7384138 to 9248624) | 78715(11412 to 229986) | 492131(78241 to 1378076) | 9047032(8513237 to 9646262) |  | -66.1(-68.7 to -63.9) | - | 1405.1(215.7 to 18303.9) | -64.4(-66.4 to -62.7) |  | -32.1(-39.4 to -26.7) | 34.4(-66.7 to 232.1) | | -11.8(-78.1 to 117.8) | -31.0(-34.2 to -27.0) |
| **Brunei Darussalam** | 18840291(16380967 to 21771517) | 33354(4001 to 119035) | 128305(15829 to 453639) | 19001950(16487638 to 21867514) |  | -66.7(-71.4 to -61.3) | - | -46.4(-93.4 to 499.5) | -66.6(-71.4 to -61.4) |  | -15.1(-25.8 to -1.3) | 107.1(-69.8 to 1051.0) | | 32.5(-80.7 to 636.3) | -14.8(-25.5 to -1.8) |
| **Bulgaria** | 3372842(2628754 to 4244313) | 62336(14927 to 166755) | 145602(35268 to 376598) | 3580780(2843240 to 4490166) |  | -39.3(-46.1 to -31.8) | - | 879.8(146.3 to 8941.3) | -32.9(-39.0 to -26.4) |  | -26.8(-42.5 to -7.9) | -43.2(-85.0 to 41.1) | | -64.3(-90.6 to -12.3) | -30.2(-44.2 to -12.7) |
| **Burkina Faso** | 197465039(151211875 to 247383706) | 172427(20383 to 548580) | 12912987(1707559 to 41470792) | 210550454(169293252 to 260292408) |  | -31.8(-46.6 to -17.3) | - | 499.2(-13.0 to 7943.0) | -27.4(-39.6 to -13.5) |  | -9.4(-26.4 to 13.0) | 24.1(-72.0 to 506.3) | | -17.3(-81.4 to 302.2) | -9.9(-24.0 to 9.4) |
| **Burundi** | 463304842(326126660 to 631022743) | 444410(49758 to 1623445) | 33003711(3938943 to 119480312) | 496752962(359100576 to 666522115) |  | -48.7(-62.7 to -33.6) | - | 1330.0(89.5 to 19088.5) | -45.9(-59.1 to -30.4) |  | -17.6(-33.6 to 0.2) | 62.8(-59.7 to 517.2) | | 8.2(-73.0 to 311.7) | -16.2(-29.2 to 0.0) |
| **Cabo Verde** | 37747113(29209819 to 49477065) | 36296(3622 to 120830) | 2744764(309535 to 8818696) | 40528173(32512277 to 51920896) |  | -56.9(-66.0 to -46.4) | - | 269.8(-54.1 to 4438.6) | -54.3(-61.7 to -44.4) |  | -32.5(-44.8 to -14.8) | 7.1(-76.8 to 403.9) | | -28.9(-84.4 to 236.7) | -32.3(-43.4 to -17.4) |
| **Cambodia** | 152749970(114357566 to 200650863) | 438751(45369 to 1739058) | 2355052(256723 to 8901900) | 155543773(116714873 to 203318248) |  | -53.8(-64.4 to -39.5) | - | 654.6(37.9 to 7759.2) | -52.5(-63.3 to -37.6) |  | -40.4(-51.0 to -28.2) | -48.9(-90.7 to 58.2) | | -66.6(-93.9 to 3.0) | -41.1(-51.3 to -29.4) |
| **Cameroon** | 137765631(92503619 to 193582502) | 98054(11837 to 347682) | 7271076(960857 to 26263716) | 145134762(97748419 to 203004294) |  | -21.3(-42.5 to 3.1) | - | 473.3(-38.9 to 6959.2) | -17.4(-37.7 to 6.0) |  | -37.1(-51.5 to -20.6) | -12.8(-78.3 to 273.0) | | -42.1(-85.3 to 150.2) | -37.4(-50.4 to -21.9) |
| **Canada** | 535697(463808 to 609215) | 3038(893 to 7620) | 12401(3620 to 30272) | 551136(482946 to 623656) |  | -62.9(-66.2 to -58.9) | - | -55.7(-83.2 to 51.6) | -62.6(-65.8 to -58.8) |  | -3.9(-13.2 to 6.3) | 43.7(-49.0 to 237.4) | | -7.5(-67.3 to 116.0) | -3.8(-12.5 to 5.9) |
| **Central African Republic** | 983328100(717134302 to 1314526535) | 446061(51989 to 1618936) | 33315618(4069910 to 120591704) | 1017089780(742752276 to 1344402468) |  | -6.1(-26.9 to 21.6) | - | 354.6(-45.6 to 4314.2) | -3.9(-24.6 to 24.9) |  | -13.2(-28.5 to 5.8) | 47.7(-67.2 to 542.1) | | -0.9(-78.1 to 326.4) | -12.8(-27.7 to 6.1) |
| **Chad** | 273987857(209674172 to 343651975) | 227391(26219 to 807531) | 16957288(2146247 to 55871467) | 291172536(230588826 to 360362414) |  | -12.7(-33.8 to 9.1) | - | 574.4(-35.5 to 9558.1) | -7.6(-26.5 to 14.1) |  | -25.6(-38.9 to -7.5) | 2.0(-78.0 to 311.0) | | -32.2(-85.4 to 173.6) | -26.0(-38.0 to -11.3) |
| **Chile** | 5036444(4525122 to 5568212) | 28357(7789 to 76188) | 111164(32777 to 286316) | 5175965(4660254 to 5703859) |  | -74.4(-76.3 to -72.4) | - | -6.5(-82.5 to 680.7) | -73.9(-75.5 to -72.0) |  | -32.8(-39.5 to -25.4) | 4.6(-75.9 to 320.7) | | -32.3(-84.5 to 170.1) | -32.7(-39.1 to -26.5) |
| **China** | 7138850(5738497 to 8574712) | 88407(14983 to 264098) | 514571(93600 to 1489122) | 7741828(6624862 to 9092454) |  | -81.5(-84.5 to -78.2) | - | -68.3(-89.0 to 38.5) | -80.6(-83.0 to -77.8) |  | -42.7(-52.2 to -32.7) | -41.2(-85.8 to 54.5) | | -60.3(-90.6 to 3.4) | -44.3(-51.0 to -36.5) |
| **Colombia** | 5557037(4060853 to 7267656) | 75137(10866 to 230457) | 475473(75287 to 1425289) | 6107647(4784367 to 7757226) |  | -68.6(-72.6 to -65.8) | - | 408.1(4.8 to 4177.1) | -66.2(-68.4 to -63.8) |  | -32.2(-48.7 to -12.4) | 16.2(-71.6 to 179.8) | | -23.7(-81.4 to 82.7) | -31.2(-45.3 to -13.1) |
| **Comoros** | 172005748(109376264 to 235129950) | 383457(44395 to 1219528) | 28476212(3456564 to 85164369) | 200865417(141465142 to 255907820) |  | -57.1(-70.4 to -7.6) | - | 1666.6(103.3 to 23740.1) | -53.7(-66.6 to -0.6) |  | -15.3(-43.4 to 13.0) | 162.4(-41.2 to 1056.6) | | 74.1(-60.8 to 673.2) | -8.5(-30.3 to 15.5) |
| **Congo** | 197106029(139424903 to 264154596) | 126728(12016 to 513210) | 9334468(949118 to 38177658) | 206567225(149174522 to 272515801) |  | -52.4(-63.8 to -38.5) | - | 158.8(-75.2 to 3352.4) | -51.0(-62.4 to -37.2) |  | -32.2(-46.7 to -14.8) | 30.7(-70.5 to 515.4) | | -13.4(-80.4 to 315.1) | -31.5(-44.9 to -15.6) |
| **Cook Islands** | 5074247(4105406 to 6163281) | 4787(287 to 23468) | 27222(1939 to 132999) | 5106255(4133837 to 6191685) |  | -52.4(-59.9 to -43.5) | - | 37.6(-89.2 to 1585.8) | -52.2(-59.6 to -43.3) |  | -26.2(-36.6 to -14.5) | 32.1(-77.2 to 648.1) | | -13.3(-85.1 to 397.9) | -26.1(-36.7 to -14.5) |
| **Costa Rica** | 2818071(2156703 to 3608568) | 18446(2210 to 65002) | 115838(14297 to 420301) | 2952354(2283574 to 3763595) |  | -72.6(-75.9 to -69.4) | - | 533.1(0.0 to 7316.0) | -71.5(-74.4 to -68.5) |  | -30.7(-46.3 to -10.5) | 10.6(-75.9 to 228.3) | | -27.7(-84.4 to 111.7) | -30.4(-45.5 to -10.3) |
| **Croatia** | 1860759(1477517 to 2336201) | 5417(992 to 16751) | 12620(2392 to 38402) | 1878796(1496429 to 2353266) |  | -83.1(-84.9 to -81.2) | - | -67.7(-92.9 to 142.2) | -83.0(-84.8 to -81.0) |  | -40.9(-52.4 to -26.3) | -47.6(-87.7 to 65.8) | | -66.9(-92.2 to 4.6) | -41.2(-52.7 to -26.5) |
| **Cuba** | 1026127(805083 to 1283769) | 5508(946 to 16556) | 39265(6886 to 112246) | 1070900(847865 to 1329269) |  | -55.3(-60.7 to -49.0) | - | -65.1(-88.4 to 40.2) | -55.5(-60.6 to -49.4) |  | -29.3(-40.8 to -15.0) | 90.4(-61.2 to 518.6) | | 21.3(-75.4 to 294.5) | -27.9(-38.4 to -14.8) |
| **Cyprus** | 581024(488564 to 671771) | 4976(861 to 16224) | 20230(3557 to 64495) | 606229(521992 to 693985) |  | -79.0(-83.0 to -75.0) | - | 122.3(-48.3 to 2316.3) | -76.2(-79.4 to -72.3) |  | -10.9(-23.9 to 6.2) | -61.9(-91.7 to 11.8) | | -75.9(-94.7 to -28.9) | -19.1(-28.3 to -9.2) |
| **Czechia** | 878528(693507 to 1061831) | 12588(3743 to 31668) | 30281(9085 to 74543) | 921397(733892 to 1104221) |  | -74.5(-77.3 to -71.5) | - | -41.2(-83.1 to 255.9) | -73.7(-76.6 to -70.7) |  | -22.8(-36.9 to -7.8) | 13.5(-62.0 to 183.8) | | -29.6(-76.4 to 74.2) | -22.7(-36.0 to -7.9) |
| **C么te d'Ivoire** | 159500060(109816209 to 216837094) | 159606(19939 to 557759) | 11900460(1663201 to 40775981) | 171560126(123547456 to 229182100) |  | -28.8(-45.3 to -10.6) | - | 89.0(-60.3 to 1027.4) | -25.6(-41.6 to -7.0) |  | -28.5(-44.3 to -10.2) | 10.9(-71.0 to 237.6) | | -26.2(-80.8 to 124.9) | -28.3(-42.4 to -11.1) |
| **Democratic People's Republic of Korea** | 34980245(25733947 to 45885484) | 547438(142995 to 1339732) | 2980458(776047 to 7134763) | 38508141(29356955 to 49517749) |  | -28.6(-48.4 to -6.8) | - | 4122.2(756.3 to 47304.3) | -22.3(-43.1 to 0.0) |  | -24.3(-40.0 to -4.9) | 22.6(-69.1 to 381.5) | | -20.0(-79.7 to 215.2) | -23.6(-35.7 to -9.6) |
| **Democratic Republic of the Congo** | 322793909(237168432 to 445830258) | 245797(21567 to 890171) | 18241464(1838135 to 65985952) | 341281170(255291124 to 467580559) |  | -20.0(-38.0 to 2.2) | - | 406.0(-50.3 to 5447.5) | -17.2(-34.8 to 5.1) |  | -37.6(-50.3 to -24.2) | 30.6(-72.6 to 401.7) | | -13.1(-81.8 to 234.4) | -36.6(-48.6 to -23.2) |
| **Denmark** | 673877(571023 to 781450) | 4915(1104 to 13136) | 20464(4783 to 52904) | 699257(601492 to 808867) |  | -54.3(-58.9 to -48.9) | - | 53.6(-66.3 to 1037.8) | -53.3(-57.9 to -48.2) |  | -34.2(-42.6 to -25.8) | 31.0(-61.8 to 304.1) | | -14.6(-75.3 to 159.9) | -33.5(-41.1 to -25.4) |
| **Djibouti** | 169630292(117350966 to 243460209) | 366733(108186 to 859710) | 27316742(8761116 to 60530465) | 197313767(140630437 to 272006301) |  | -34.3(-52.8 to -10.1) | - | 6857.3(1540.4 to 67096.6) | -27.7(-46.3 to -2.6) |  | -38.8(-56.1 to -18.9) | 46.3(-56.7 to 415.6) | | -2.2(-71.2 to 243.2) | -35.3(-49.2 to -17.9) |
| **Dominica** | 13210297(10557447 to 16413895) | 26237(2133 to 128487) | 164820(14347 to 791177) | 13401355(10752940 to 16638856) |  | -54.3(-61.3 to -44.7) | - | -84.0(-98.6 to 165.1) | -55.0(-61.6 to -46.8) |  | -8.2(-23.7 to 10.1) | 111.0(-63.0 to 1126.0) | | 38.1(-75.7 to 698.9) | -7.7(-23.4 to 10.7) |
| **Dominican Republic** | 30890548(23461136 to 41256989) | 54995(4411 to 268325) | 335482(28817 to 1641454) | 31281025(23843252 to 41762199) |  | -65.2(-72.1 to -56.4) | - | -73.3(-97.7 to 221.4) | -65.2(-72.0 to -56.4) |  | -2.1(-28.5 to 35.5) | 141.7(-60.0 to 1483.9) | | 57.1(-73.8 to 934.5) | -1.6(-27.7 to 35.9) |
| **Ecuador** | 15147239(10557996 to 19606701) | 257008(40175 to 790576) | 1615509(260019 to 5037068) | 17019756(13657594 to 21495073) |  | -77.9(-82.0 to -74.6) | - | 371.4(-26.7 to 4447.5) | -75.2(-77.5 to -72.6) |  | -40.1(-54.8 to -23.2) | -15.5(-77.4 to 141.3) | | -44.2(-85.2 to 58.6) | -40.2(-52.2 to -25.4) |
| **Egypt** | 4724036(3514044 to 6706007) | 29978(6059 to 86655) | 436311(90420 to 1239979) | 5190325(3966834 to 7310681) |  | -64.6(-70.4 to -54.7) | - | 995.1(176.8 to 9421.5) | -60.6(-66.0 to -50.1) |  | -29.8(-45.4 to -11.2) | -13.0(-80.9 to 140.1) | | -41.9(-87.2 to 60.7) | -30.9(-44.6 to -14.8) |
| **El Salvador** | 4840373(3682838 to 6249965) | 29517(3294 to 109555) | 185388(21512 to 705375) | 5055278(3924774 to 6393437) |  | -85.3(-87.5 to -83.4) | - | 60.7(-83.8 to 1583.8) | -84.7(-86.2 to -83.0) |  | -18.9(-37.7 to 4.4) | 13.5(-71.1 to 471.5) | | -26.1(-81.3 to 272.2) | -19.1(-36.4 to 2.7) |
| **Equatorial Guinea** | 95794908(59833030 to 151712176) | 71792(6103 to 262586) | 5384928(542199 to 19837385) | 101251628(65973918 to 158830295) |  | -85.1(-90.7 to -76.4) | - | -9.1(-90.6 to 1001.8) | -84.6(-90.3 to -75.9) |  | -36.0(-52.2 to -14.8) | 32.6(-71.0 to 526.6) | | -10.7(-80.7 to 318.1) | -35.0(-50.1 to -14.6) |
| **Eritrea** | 418820087(288366296 to 588157530) | 495402(61462 to 1725800) | 36891116(4626694 to 127107660) | 456206605(321121369 to 626232556) |  | -38.1(-55.3 to -15.2) | - | 1922.5(156.5 to 29409.4) | -34.1(-50.2 to -10.1) |  | -30.2(-45.9 to -11.4) | 39.2(-64.0 to 471.8) | | -7.4(-76.1 to 280.6) | -28.8(-41.3 to -12.5) |
| **Estonia** | 3348670(2132609 to 4701127) | 521962(242116 to 901277) | 1223195(578609 to 2085335) | 5093827(4030748 to 6465311) |  | -62.7(-70.7 to -53.9) | - | 24.0(-34.1 to 215.9) | -46.2(-50.9 to -40.9) |  | -38.0(-56.2 to -15.6) | -32.2(-62.7 to 6.2) | | -57.4(-76.4 to -33.3) | -43.6(-55.0 to -30.4) |
| **Eswatini** | 259846753(159453259 to 370600822) | 831429(155140 to 2061172) | 61484722(13375495 to 140197545) | 322162904(226131479 to 434890621) |  | 8.7(-30.2 to 61.8) | - | 3191.1(904.1 to 17222.4) | 34.4(-8.5 to 97.7) |  | -44.3(-63.0 to -24.7) | -18.9(-77.0 to 77.4) | | -45.9(-84.6 to 18.7) | -44.6(-57.1 to -28.8) |
| **Ethiopia** | 172877668(134681045 to 207089932) | 162990(28329 to 513190) | 12264059(2120708 to 39364059) | 185304716(153981967 to 216382297) |  | -69.4(-75.9 to -63.2) | - | 974.5(47.8 to 13646.5) | -67.9(-74.4 to -61.5) |  | -46.2(-55.9 to -36.1) | 14.1(-68.1 to 250.9) | | -23.2(-78.7 to 137.0) | -45.1(-53.2 to -35.6) |
| **Fiji** | 18415856(14779109 to 22513449) | 12972(953 to 60164) | 70830(5251 to 304363) | 18499659(14837046 to 22586672) |  | -40.7(-52.2 to -27.3) | - | 94.8(-87.0 to 2680.4) | -40.6(-52.2 to -27.2) |  | -20.1(-34.9 to -2.6) | 90.9(-69.0 to 1018.7) | | 24.6(-79.8 to 625.7) | -20.0(-34.8 to -2.7) |
| **Finland** | 781841(655548 to 909890) | 12180(3147 to 31887) | 49512(13097 to 127289) | 843533(740190 to 959857) |  | -74.1(-76.8 to -71.2) | - | 88.3(-56.5 to 1414.0) | -73.0(-75.5 to -70.1) |  | -35.7(-44.5 to -26.9) | 49.7(-53.4 to 328.7) | | -2.5(-69.9 to 177.4) | -33.8(-40.9 to -26.2) |
| **France** | 1106279(953144 to 1272757) | 9911(2759 to 27919) | 39555(11509 to 106141) | 1155745(1014208 to 1316313) |  | -70.2(-72.7 to -67.6) | - | -10.2(-69.1 to 291.5) | -69.4(-71.8 to -67.0) |  | -31.5(-39.2 to -23.4) | 14.1(-60.4 to 167.4) | | -26.6(-74.5 to 72.4) | -31.1(-38.4 to -23.4) |
| **Gabon** | 124349353(85983282 to 162873909) | 109428(11698 to 429407) | 8136330(899077 to 29352215) | 132595111(94717444 to 171057075) |  | -46.7(-62.1 to -30.6) | - | 287.8(-60.8 to 4925.7) | -44.3(-58.4 to -27.7) |  | -39.7(-52.0 to -24.4) | 13.4(-74.2 to 369.3) | | -24.3(-82.9 to 220.2) | -38.9(-50.6 to -24.8) |
| **Gambia** | 192200956(143606031 to 245061958) | 133107(14330 to 497155) | 9938215(1123538 to 34851500) | 202272278(151892938 to 255742282) |  | -15.5(-36.7 to 10.9) | - | 548.5(-23.8 to 7734.5) | -11.1(-30.2 to 15.5) |  | -20.1(-36.6 to 1.7) | 4.5(-77.9 to 319.2) | | -30.5(-85.4 to 177.4) | -20.7(-36.0 to -2.4) |
| **Georgia** | 12601950(8310517 to 16661097) | 1966672(968451 to 3378608) | 4506666(2338968 to 7503383) | 19075288(16013684 to 22471756) |  | -53.6(-63.8 to -44.1) | - | 3523.8(890.3 to 32289.1) | -36.2(-43.5 to -28.0) |  | -34.1(-50.2 to -15.6) | 26.5(-20.9 to 92.8) | | -20.8(-51.0 to 20.2) | -27.6(-39.5 to -14.1) |
| **Germany** | 639935(542138 to 751117) | 10766(3598 to 25872) | 44775(16395 to 98723) | 695477(604556 to 793971) |  | -81.7(-83.5 to -79.6) | - | -47.0(-82.8 to 147.6) | -81.1(-82.8 to -79.0) |  | -18.6(-29.2 to -7.7) | 101.4(-18.0 to 322.9) | | 30.3(-47.3 to 175.0) | -15.8(-24.1 to -7.1) |
| **Ghana** | 165116429(127035129 to 206804382) | 133325(16298 to 429420) | 9859798(1274434 to 31615834) | 175109552(137053484 to 216301047) |  | -39.8(-53.9 to -21.4) | - | 319.8(-49.3 to 5622.4) | -36.7(-50.4 to -18.8) |  | -32.9(-45.3 to -17.0) | 2.3(-76.6 to 318.5) | | -32.6(-84.3 to 180.7) | -32.9(-43.9 to -20.7) |
| **Greece** | 1162460(967550 to 1326608) | 16379(2442 to 50922) | 65102(10433 to 208418) | 1243941(1106142 to 1384894) |  | -59.0(-64.4 to -54.3) | - | 384.7(5.8 to 4635.4) | -54.4(-57.9 to -50.7) |  | -26.9(-36.9 to -16.5) | -39.6(-85.9 to 63.1) | | -60.9(-91.0 to 5.0) | -30.2(-37.1 to -23.6) |
| **Greenland** | 10530093(8230207 to 12633216) | 107495(12725 to 368926) | 416873(53036 to 1541942) | 11054461(8973969 to 13198435) |  | -66.5(-72.7 to -59.2) | - | -65.7(-96.0 to 207.6) | -66.3(-72.2 to -60.2) |  | -29.7(-42.7 to -15.8) | 29.0(-71.3 to 537.4) | | -17.9(-81.5 to 309.5) | -29.0(-40.7 to -16.3) |
| **Grenada** | 2574279(2154287 to 3085823) | 4984(385 to 25054) | 34143(2661 to 166661) | 2613406(2199704 to 3117943) |  | -59.0(-64.3 to -53.3) | - | -67.8(-96.8 to 207.5) | -59.0(-64.3 to -53.6) |  | -28.9(-37.9 to -18.9) | 127.5(-68.2 to 1470.9) | | 51.1(-79.3 to 923.0) | -28.3(-37.3 to -18.5) |
| **Guam** | 7986077(6611824 to 9496641) | 13173(1266 to 53580) | 74880(7859 to 276072) | 8074130(6758709 to 9576544) |  | -46.9(-54.3 to -37.9) | - | 604.8(22.2 to 8048.8) | -44.9(-52.1 to -36.2) |  | -10.2(-23.4 to 4.7) | -64.6(-95.2 to 37.0) | | -76.7(-96.9 to -11.0) | -12.8(-25.2 to 1.2) |
| **Guatemala** | 11492935(8726916 to 14675368) | 98197(12807 to 334219) | 608589(73573 to 1969875) | 12199721(9582390 to 15372227) |  | -89.1(-90.9 to -87.1) | - | 73.4(-74.5 to 1775.2) | -88.4(-90.0 to -86.6) |  | -29.6(-47.1 to -7.2) | -5.1(-76.0 to 201.9) | | -37.6(-84.3 to 98.5) | -29.9(-47.6 to -8.9) |
| **Guinea** | 198884979(143684416 to 265793383) | 135036(16671 to 458469) | 10083522(1188223 to 34383971) | 209103537(153563741 to 278284058) |  | -33.3(-48.8 to -13.8) | - | 342.8(-50.9 to 5330.7) | -29.9(-44.7 to -11.0) |  | -19.4(-35.7 to 0.0) | 1.4(-73.6 to 319.4) | | -31.9(-82.5 to 180.5) | -20.1(-35.0 to -2.1) |
| **Guinea-Bissau** | 240815623(179165029 to 313129751) | 134505(18127 to 479151) | 10017053(1351129 to 34978063) | 250967181(191597232 to 328678494) |  | -45.5(-58.2 to -29.5) | - | 279.0(-49.7 to 4689.4) | -42.9(-55.7 to -26.8) |  | -28.3(-42.0 to -11.4) | -20.2(-82.0 to 234.1) | | -46.2(-88.0 to 122.0) | -29.3(-42.0 to -14.5) |
| **Guyana** | 33553532(25623713 to 43365271) | 79126(5976 to 409879) | 487129(38355 to 2704531) | 34119787(26239123 to 43798246) |  | -30.7(-42.0 to -16.6) | - | 7.8(-90.2 to 1226.8) | -30.4(-41.8 to -15.9) |  | -33.5(-50.0 to -12.6) | 68.1(-71.4 to 1020.7) | | 10.2(-81.3 to 630.4) | -33.1(-49.4 to -13.1) |
| **Haiti** | 56151837(41932146 to 75030621) | 80018(6753 to 367222) | 489912(42287 to 2033507) | 56721767(42346249 to 75638526) |  | -58.2(-67.3 to -47.6) | - | -61.4(-96.6 to 303.3) | -58.2(-66.9 to -47.8) |  | -24.7(-37.2 to -10.2) | 74.9(-72.4 to 1212.3) | | 14.4(-82.0 to 756.0) | -24.5(-36.6 to -9.9) |
| **Honduras** | 23998946(17832286 to 33815682) | 214647(26944 to 755504) | 1313435(172249 to 4428520) | 25527028(19655919 to 35268797) |  | -56.9(-66.9 to -42.7) | - | 1018.0(68.9 to 12767.4) | -54.4(-64.5 to -40.3) |  | -19.3(-36.2 to 4.8) | 27.4(-68.7 to 274.3) | | -16.8(-79.7 to 143.5) | -18.9(-35.0 to 3.8) |
| **Hungary** | 972168(776123 to 1187549) | 8830(1458 to 29902) | 21806(3516 to 74422) | 1002804(813012 to 1222402) |  | -84.1(-86.0 to -82.0) | - | 65.2(-63.6 to 1597.9) | -83.1(-85.0 to -80.9) |  | -40.7(-51.1 to -29.3) | -64.2(-92.6 to 19.0) | | -77.4(-95.4 to -25.6) | -43.0(-52.5 to -32.6) |
| **Iceland** | 544619(464744 to 629957) | 2233(256 to 7995) | 9254(1031 to 32193) | 556106(471312 to 643172) |  | -73.0(-77.6 to -69.2) | - | 167.5(-54.1 to 2355.5) | -70.5(-73.5 to -66.8) |  | -31.3(-39.8 to -20.0) | -79.8(-96.0 to -31.6) | | -86.9(-97.4 to -55.3) | -36.4(-43.4 to -28.9) |
| **India** | 104674284(79309308 to 126654493) | 753377(125684 to 1929433) | 14970782(2451276 to 37897038) | 120398442(103905905 to 139927328) |  | -61.1(-67.3 to -54.8) | - | 2453.1(347.6 to 38411.7) | -56.3(-60.7 to -51.0) |  | -32.0(-45.7 to -17.2) | 21.0(-66.6 to 190.4) | | -19.7(-77.7 to 93.7) | -30.5(-40.1 to -19.8) |
| **Indonesia** | 109854070(94317803 to 126871100) | 170673(14760 to 729952) | 906011(88263 to 3742835) | 110930755(96042595 to 127714542) |  | -53.8(-60.0 to -46.7) | - | 109.8(-83.7 to 3019.5) | -53.0(-58.6 to -46.2) |  | -37.9(-47.3 to -25.9) | -56.1(-91.9 to 167.8) | | -71.4(-94.7 to 74.4) | -38.6(-47.9 to -26.9) |
| **Iran (Islamic Republic of)** | 3243727(2932767 to 3595737) | 8501(1967 to 22804) | 119343(28807 to 321667) | 3371571(3125890 to 3730410) |  | -69.5(-74.0 to -63.1) | - | 432.3(5.7 to 7778.2) | -68.4(-72.7 to -62.0) |  | -32.1(-38.2 to -23.2) | 10.4(-74.8 to 340.3) | | -26.8(-83.1 to 192.6) | -31.9(-35.9 to -23.7) |
| **Iraq** | 11670705(9223616 to 14889042) | 51900(12733 to 141267) | 731688(177505 to 1923091) | 12454294(9843517 to 15550263) |  | -62.0(-69.7 to -50.4) | - | 894.5(128.0 to 13598.3) | -60.0(-68.1 to -47.6) |  | -44.3(-54.1 to -31.0) | -1.5(-74.6 to 244.4) | | -34.4(-83.0 to 130.0) | -43.7(-52.6 to -31.2) |
| **Ireland** | 917371(769178 to 1081305) | 5904(1180 to 18575) | 23797(5174 to 72362) | 947072(807032 to 1112444) |  | -67.4(-71.3 to -63.4) | - | -2.5(-75.3 to 824.0) | -66.5(-70.2 to -62.5) |  | -32.0(-39.8 to -23.4) | -21.7(-81.9 to 141.8) | | -49.6(-88.3 to 54.7) | -32.5(-39.5 to -24.4) |
| **Israel** | 516551(403806 to 609576) | 13570(3780 to 33970) | 55728(16350 to 136650) | 585849(517462 to 663722) |  | -68.8(-73.3 to -63.4) | - | -35.6(-82.0 to 302.5) | -66.8(-70.0 to -63.2) |  | -34.0(-47.4 to -22.6) | 10.3(-65.4 to 169.7) | | -28.8(-77.8 to 74.6) | -32.9(-38.8 to -26.3) |
| **Italy** | 673889(584106 to 756996) | 8367(2756 to 20157) | 33903(11500 to 76414) | 716158(645328 to 793692) |  | -72.5(-74.6 to -70.1) | - | -10.3(-72.4 to 406.3) | -70.9(-72.7 to -69.1) |  | -19.0(-25.2 to -13.0) | -8.5(-62.0 to 89.6) | | -41.4(-75.9 to 21.9) | -20.3(-24.1 to -16.2) |
| **Jamaica** | 1508815(1172381 to 1902611) | 6278(843 to 22926) | 41111(5898 to 145494) | 1556203(1225842 to 1949561) |  | -62.2(-66.4 to -56.6) | - | -30.1(-92.4 to 1046.0) | -61.8(-66.0 to -56.8) |  | -17.9(-34.3 to 1.1) | 119.3(-64.0 to 1109.2) | | 40.3(-76.4 to 664.7) | -16.8(-32.3 to 2.4) |
| **Japan** | 1312247(1142656 to 1448990) | 5234(524 to 22798) | 20909(2212 to 87392) | 1338390(1182245 to 1465597) |  | -72.8(-74.8 to -71.1) | - | -68.2(-95.2 to 100.0) | -72.7(-74.5 to -71.4) |  | -33.4(-37.0 to -30.1) | 13.5(-79.4 to 351.3) | | -27.3(-86.8 to 189.3) | -33.2(-35.5 to -30.6) |
| **Jordan** | 1245865(928851 to 1511331) | 7185(1102 to 24685) | 102043(16807 to 334121) | 1355093(1145316 to 1600989) |  | -78.1(-82.2 to -72.5) | - | 627.2(60.3 to 8332.3) | -75.3(-79.3 to -69.1) |  | -35.9(-49.7 to -20.7) | -38.3(-86.1 to 73.0) | | -59.1(-90.9 to 14.5) | -38.5(-47.5 to -27.3) |
| **Kazakhstan** | 11746528(7529134 to 16340525) | 2357217(1163179 to 3918785) | 5415850(2688714 to 8745342) | 19519595(16693247 to 22369900) |  | -57.1(-70.4 to -42.4) | - | 4429.5(1152.3 to 44396.7) | -12.0(-18.6 to -5.3) |  | -55.6(-67.8 to -39.3) | -61.3(-76.7 to -43.7) | | -75.5(-85.3 to -64.3) | -64.3(-69.5 to -59.0) |
| **Kenya** | 197066378(151144985 to 245953245) | 91481(21990 to 241821) | 6829820(1705311 to 17087788) | 203987678(156977890 to 253455349) |  | -7.3(-22.7 to 7.9) | - | 2955.4(678.8 to 42240.3) | -4.0(-19.4 to 11.0) |  | -30.4(-41.0 to -18.4) | -0.2(-75.7 to 282.4) | | -33.4(-83.8 to 155.6) | -30.5(-40.7 to -19.0) |
| **Kiribati** | 291556224(229263340 to 370568683) | 334698(28813 to 1454783) | 1787363(151039 to 7563703) | 293678285(230988274 to 373158777) |  | -31.9(-45.8 to -13.9) | - | 126.0(-81.1 to 2623.8) | -31.6(-45.6 to -13.8) |  | -18.5(-29.8 to -4.7) | 32.4(-76.7 to 740.9) | | -14.0(-84.9 to 446.5) | -18.5(-29.7 to -4.8) |
| **Kuwait** | 2888239(2380978 to 3472244) | 6517(1324 to 18707) | 94552(21497 to 254107) | 2989308(2481743 to 3588521) |  | -61.3(-66.1 to -55.8) | - | 300.6(-8.6 to 6108.7) | -60.2(-64.9 to -54.6) |  | -47.1(-55.0 to -37.6) | -15.6(-78.4 to 166.0) | | -43.6(-85.6 to 77.3) | -46.9(-54.7 to -37.2) |
| **Kyrgyzstan** | 17759954(9097617 to 27105172) | 4359146(1617628 to 7394199) | 9951098(3846664 to 16631873) | 32070198(28047412 to 36576343) |  | -39.6(-59.2 to -18.6) | - | 24670.2(5660.8 to 277990.5) | 14.4(5.9 to 24.1) |  | -43.9(-71.1 to -9.4) | -29.1(-70.8 to 30.7) | | -55.3(-81.4 to -17.7) | -46.6(-52.9 to -39.5) |
| **Lao People's Democratic Republic** | 137532833(99932108 to 175445602) | 261361(23736 to 1134073) | 1386723(140776 to 5569782) | 139180918(100913219 to 178005224) |  | -62.2(-70.8 to -52.1) | - | 92.8(-79.0 to 2610.5) | -61.6(-69.9 to -50.9) |  | -44.0(-54.2 to -32.2) | -56.0(-92.1 to 164.2) | | -71.3(-94.9 to 71.5) | -44.5(-54.5 to -33.8) |
| **Latvia** | 5394955(3962274 to 7000688) | 433222(170570 to 841061) | 1013961(418563 to 1996961) | 6842138(5548755 to 8423020) |  | -36.2(-47.9 to -13.9) | - | -50.5(-70.8 to -0.8) | -36.5(-41.8 to -30.4) |  | -31.6(-46.9 to -11.9) | -23.2(-60.3 to 32.3) | | -51.8(-75.3 to -16.7) | -35.2(-47.5 to -20.7) |
| **Lebanon** | 2842626(2031495 to 4543655) | 7128(698 to 30482) | 103420(10619 to 429522) | 2953174(2150979 to 4709003) |  | -72.9(-78.9 to -55.4) | - | 201.1(-61.0 to 3687.5) | -72.1(-77.9 to -53.8) |  | -26.2(-39.0 to -12.1) | 39.1(-72.1 to 415.2) | | -9.5(-81.8 to 234.0) | -25.6(-36.8 to -12.3) |
| **Lesotho** | 575692706(415162811 to 743852319) | 781036(200041 to 1986882) | 57749203(15243670 to 141288818) | 634222945(472944548 to 803333232) |  | 54.1(12.9 to 112.3) | - | 7644.0(1686.1 to 84308.5) | 67.0(24.7 to 127.4) |  | -26.3(-43.5 to -6.7) | 30.7(-69.6 to 395.6) | | -12.9(-79.7 to 231.2) | -25.2(-40.0 to -8.7) |
| **Liberia** | 129794074(94104369 to 178831036) | 78612(7732 to 330241) | 5798322(628745 to 23067860) | 135671009(100449614 to 183631814) |  | -59.2(-69.0 to -44.6) | - | 146.3(-75.6 to 3115.2) | -57.6(-67.1 to -42.4) |  | -23.7(-40.2 to -2.7) | 6.8(-75.8 to 358.3) | | -29.1(-83.8 to 205.6) | -23.9(-39.1 to -4.9) |
| **Libya** | 4938243(3504224 to 6382611) | 13755(1214 to 55162) | 195346(17837 to 753873) | 5147344(3661477 to 6562946) |  | -65.5(-73.3 to -53.0) | - | 208.7(-66.7 to 3712.8) | -64.3(-72.0 to -52.3) |  | -1.6(-21.2 to 20.3) | 62.7(-70.7 to 783.9) | | 5.8(-80.9 to 479.4) | -1.2(-18.8 to 20.2) |
| **Lithuania** | 9794841(6535961 to 13049076) | 1538064(705083 to 2695194) | 3522495(1743933 to 5799638) | 14855400(12118876 to 18173582) |  | -28.3(-44.2 to -12.3) | - | 176.0(5.3 to 990.3) | -3.5(-10.8 to 4.7) |  | -42.9(-57.1 to -26.2) | -12.9(-46.9 to 31.1) | | -45.1(-66.8 to -17.6) | -41.3(-52.3 to -27.5) |
| **Luxembourg** | 492392(397426 to 601177) | 5003(659 to 17966) | 21579(2995 to 76174) | 518974(435371 to 628012) |  | -69.9(-74.8 to -64.6) | - | 236.9(-35.8 to 3031.7) | -67.6(-71.2 to -63.1) |  | -25.8(-37.6 to -12.6) | -30.9(-88.2 to 160.5) | | -55.1(-92.4 to 68.7) | -27.8(-36.9 to -16.6) |
| **Madagascar** | 281443528(205363340 to 378417481) | 228942(32412 to 786702) | 17149454(2623347 to 58977812) | 298821925(222925652 to 394802162) |  | -42.0(-54.6 to -27.9) | - | 1497.5(174.3 to 16905.7) | -40.3(-53.1 to -26.2) |  | -22.2(-38.7 to -4.6) | 141.6(-46.2 to 618.7) | | 61.6(-64.4 to 379.0) | -19.8(-34.0 to -3.5) |
| **Malawi** | 269244079(207575130 to 342955609) | 160619(22991 to 508632) | 11942246(1812326 to 36329391) | 281346944(219514526 to 355132557) |  | -40.1(-51.4 to -26.8) | - | 1391.8(206.0 to 16719.1) | -38.8(-50.2 to -25.7) |  | -28.5(-43.0 to -12.5) | 116.9(-59.8 to 626.9) | | 44.7(-73.2 to 384.2) | -26.9(-40.6 to -11.3) |
| **Malaysia** | 22165541(18042645 to 27215276) | 68021(12690 to 223092) | 372074(77295 to 1160971) | 22605636(18425055 to 27756839) |  | -48.5(-54.5 to -41.7) | - | 840.1(59.7 to 9081.4) | -47.3(-52.7 to -41.1) |  | -25.7(-39.3 to -9.1) | -16.0(-84.5 to 345.0) | | -44.7(-89.7 to 188.6) | -26.1(-39.4 to -10.1) |
| **Maldives** | 12054091(10109687 to 14339119) | 18942(1737 to 81249) | 105407(9727 to 465459) | 12178440(10228042 to 14424019) |  | -88.6(-91.1 to -84.8) | - | -43.2(-94.9 to 847.3) | -88.4(-90.9 to -84.4) |  | -32.1(-42.6 to -18.9) | -53.4(-90.4 to 147.8) | | -69.5(-93.7 to 61.3) | -32.9(-43.1 to -21.0) |
| **Mali** | 154261426(114948799 to 208049963) | 126117(12664 to 418051) | 9384544(1044696 to 30713671) | 163772087(127102346 to 216933542) |  | -44.4(-56.5 to -30.0) | - | 301.6(-50.1 to 5158.3) | -41.4(-52.4 to -27.7) |  | -26.6(-41.7 to -7.3) | 5.7(-75.6 to 367.0) | | -29.7(-83.8 to 212.5) | -26.8(-39.6 to -11.3) |
| **Malta** | 478231(382870 to 598795) | 1336(148 to 5451) | 6005(692 to 22849) | 485572(388183 to 608039) |  | -62.5(-68.6 to -56.2) | - | 175.3(-60.5 to 3474.9) | -60.8(-65.9 to -54.8) |  | -18.0(-30.5 to -2.1) | -66.5(-93.6 to 14.5) | | -78.2(-95.9 to -25.4) | -21.0(-32.6 to -6.2) |
| **Marshall Islands** | 65268842(45891013 to 86051149) | 260323(31585 to 919073) | 1391960(191384 to 4906472) | 66921124(46943647 to 87857561) |  | -34.3(-48.4 to -17.7) | - | 2056.6(331.3 to 28783.4) | -30.7(-45.5 to -13.8) |  | -27.2(-39.8 to -12.4) | -52.0(-92.9 to 111.9) | | -68.7(-95.4 to 37.9) | -29.3(-40.8 to -15.4) |
| **Mauritania** | 60052396(40021917 to 86679703) | 42529(3836 to 149483) | 3172924(307111 to 10912146) | 63267850(43010118 to 90937989) |  | -61.3(-70.1 to -48.5) | - | 226.0(-63.5 to 3675.6) | -59.2(-67.5 to -45.7) |  | -38.2(-53.4 to -22.2) | -19.1(-81.5 to 204.1) | | -46.4(-87.7 to 102.7) | -38.6(-52.8 to -24.0) |
| **Mauritius** | 3858226(3134200 to 4771592) | 6127(1146 to 20656) | 35335(7150 to 113125) | 3899688(3152842 to 4828650) |  | -57.8(-62.5 to -52.9) | - | 490.4(4.6 to 5991.9) | -56.8(-61.4 to -51.9) |  | -17.6(-30.3 to -2.6) | -48.3(-87.6 to 93.8) | | -66.4(-91.8 to 25.4) | -18.7(-31.2 to -4.1) |
| **Mexico** | 7272115(5921034 to 8579534) | 70683(11828 to 212098) | 443338(76603 to 1355524) | 7786137(6716747 to 9009396) |  | -79.5(-80.9 to -78.3) | - | 772.4(93.6 to 7895.3) | -78.3(-79.2 to -77.2) |  | -21.7(-34.9 to -7.9) | 28.2(-68.6 to 270.9) | | -15.3(-79.5 to 142.5) | -21.1(-31.5 to -8.8) |
| **Micronesia (Federated States of)** | 51678899(34331521 to 67971916) | 63669(3074 to 336258) | 341880(16718 to 1690099) | 52084448(34808996 to 68620334) |  | -55.5(-66.0 to -42.1) | - | 44.8(-87.9 to 1857.4) | -55.3(-65.7 to -41.8) |  | -22.6(-39.4 to -5.7) | 38.6(-77.5 to 675.3) | | -10.5(-85.0 to 403.5) | -22.5(-39.0 to -5.6) |
| **Monaco** | 2042915(1624113 to 2495941) | 12765(1254 to 48746) | 50729(4893 to 187585) | 2106409(1704444 to 2574830) |  | -62.8(-70.6 to -52.2) | - | 3.0(-89.2 to 1048.6) | -62.0(-69.7 to -51.9) |  | -17.2(-32.2 to -0.3) | 24.9(-74.6 to 530.8) | | -19.7(-83.8 to 303.2) | -17.0(-31.6 to -0.2) |
| **Mongolia** | 41498850(24219884 to 57387495) | 3432049(744638 to 8824683) | 7777247(1621910 to 20025663) | 52708146(41714205 to 66750342) |  | -49.7(-62.7 to -35.4) | - | 1368.1(181.7 to 15798.7) | -41.3(-52.3 to -26.8) |  | -41.1(-61.1 to -20.8) | 29.6(-59.5 to 230.7) | | -18.5(-74.4 to 106.7) | -36.2(-48.8 to -20.8) |
| **Montenegro** | 1823745(1487501 to 2186947) | 13273(1667 to 48144) | 30736(4077 to 104871) | 1867754(1530202 to 2230156) |  | -40.7(-48.7 to -30.5) | - | 248.9(-29.6 to 3192.8) | -39.0(-46.8 to -28.7) |  | -28.7(-40.6 to -15.3) | -25.6(-87.6 to 168.6) | | -53.6(-92.2 to 71.7) | -29.3(-40.6 to -15.8) |
| **Morocco** | 36810529(27409677 to 64055074) | 120774(24421 to 348522) | 1666055(354168 to 4624874) | 38597359(29068843 to 69439580) |  | -57.9(-67.3 to -40.5) | - | 662.9(47.2 to 8863.3) | -56.3(-65.9 to -37.6) |  | -39.8(-51.6 to -18.6) | 10.1(-73.4 to 359.8) | | -27.2(-82.4 to 205.8) | -39.3(-50.4 to -17.0) |
| **Mozambique** | 432226507(309870347 to 582374607) | 696498(176587 to 1784862) | 51473570(13345131 to 124081898) | 484396574(366283112 to 628345411) |  | -29.8(-44.1 to -10.8) | - | 977.0(188.7 to 8149.3) | -23.4(-39.1 to -3.7) |  | -24.8(-43.0 to -1.5) | 39.3(-56.6 to 257.0) | | -7.3(-70.9 to 137.2) | -23.2(-39.5 to -3.7) |
| **Myanmar** | 87849642(62477756 to 117686112) | 2071741(551330 to 4953949) | 11010406(3325506 to 26630890) | 100931789(77498349 to 129796596) |  | -63.6(-72.8 to -52.6) | - | 1029.7(140.2 to 11204.9) | -59.3(-68.8 to -47.2) |  | -49.3(-61.1 to -35.1) | -10.6(-69.4 to 95.0) | | -41.7(-79.9 to 28.1) | -48.1(-57.7 to -35.8) |
| **Namibia** | 161912099(115577984 to 219401143) | 285722(87671 to 634477) | 21417260(7185371 to 46097587) | 183615080(133432528 to 238692264) |  | -27.7(-48.2 to 6.2) | - | 5031.6(953.5 to 50904.3) | -19.6(-40.0 to 15.2) |  | -40.3(-55.3 to -21.6) | 4.9(-71.3 to 286.1) | | -29.8(-80.6 to 159.1) | -39.2(-52.0 to -24.2) |
| **Nauru** | 37385050(29979880 to 48471000) | 41716(3226 to 193206) | 226741(19330 to 1104358) | 37653506(30110161 to 49260986) |  | -5.4(-24.0 to 17.2) | - | 177.8(-80.8 to 3673.6) | -5.0(-23.7 to 17.7) |  | -51.1(-58.4 to -42.4) | -16.4(-86.1 to 402.3) | | -44.8(-90.9 to 228.6) | -51.0(-58.4 to -42.6) |
| **Nepal** | 89745131(66103793 to 112728145) | 480862(102335 to 1387660) | 9468753(2068070 to 24781588) | 99694746(78342785 to 121180530) |  | -70.0(-77.3 to -60.8) | - | 40.1(-61.3 to 803.5) | -67.9(-75.5 to -58.1) |  | -33.6(-48.3 to -17.6) | 21.8(-68.5 to 201.8) | | -19.3(-78.8 to 99.9) | -32.3(-44.2 to -18.0) |
| **Netherlands** | 582670(500102 to 670821) | 5157(1457 to 12556) | 21260(6033 to 52034) | 609088(534981 to 693532) |  | -64.0(-67.8 to -60.1) | - | 33.6(-58.9 to 629.9) | -62.8(-66.4 to -58.8) |  | -23.2(-32.4 to -14.1) | 7.7(-57.9 to 150.7) | | -30.4(-73.0 to 59.7) | -23.3(-31.7 to -15.1) |
| **New Zealand** | 681065(579585 to 789212) | 5448(1014 to 17572) | 22635(4600 to 68432) | 709148(619080 to 811843) |  | -68.8(-72.1 to -65.1) | - | -33.3(-82.6 to 336.8) | -68.0(-70.9 to -64.3) |  | -16.8(-27.0 to -5.8) | 6.0(-74.1 to 224.0) | | -31.1(-82.8 to 109.1) | -17.2(-25.0 to -7.7) |
| **Nicaragua** | 9630395(7774776 to 11786547) | 58526(8071 to 188430) | 365931(54492 to 1161722) | 10054853(8274778 to 12138621) |  | -75.1(-77.9 to -71.8) | - | 33.9(-74.8 to 987.5) | -74.3(-76.8 to -71.4) |  | -32.9(-44.7 to -18.4) | 12.5(-73.8 to 234.8) | | -26.1(-82.9 to 119.1) | -32.5(-43.8 to -19.2) |
| **Niger** | 170995850(125590568 to 227464184) | 125488(15285 to 419620) | 9374580(1085577 to 31269185) | 180495919(137313211 to 236739576) |  | -57.6(-68.3 to -44.1) | - | 209.0(-65.2 to 4655.9) | -55.2(-65.5 to -41.9) |  | -11.6(-29.9 to 11.8) | 10.1(-72.7 to 395.3) | | -26.2(-81.8 to 228.4) | -12.5(-29.7 to 8.1) |
| **Nigeria** | 122755382(93332986 to 158222163) | 142252(30747 to 422658) | 10719147(2345645 to 30664303) | 133616781(105042188 to 168033134) |  | -53.1(-63.8 to -38.9) | - | 1994.2(346.6 to 23454.9) | -49.1(-60.5 to -34.2) |  | -27.1(-45.8 to -3.5) | 7.9(-71.6 to 178.3) | | -27.5(-81.1 to 86.4) | -27.1(-44.2 to -5.6) |
| **Niue** | 12251733(9669110 to 15771081) | 11833(1037 to 56652) | 64975(6210 to 283998) | 12328541(9726071 to 15908039) |  | -58.3(-66.0 to -48.3) | - | 32.3(-90.8 to 1366.6) | -58.2(-65.8 to -48.0) |  | -21.1(-31.7 to -8.9) | 27.5(-75.8 to 762.6) | | -17.6(-84.3 to 465.7) | -21.1(-31.6 to -9.3) |
| **North Macedonia** | 3768263(2993505 to 4667998) | 42336(10892 to 111594) | 96364(24373 to 244623) | 3906963(3131533 to 4803545) |  | -78.7(-81.3 to -75.5) | - | 97.5(-55.1 to 1857.7) | -77.6(-80.0 to -74.4) |  | -25.2(-40.6 to -6.7) | -31.5(-80.6 to 97.9) | | -57.3(-87.8 to 23.2) | -26.6(-41.0 to -9.5) |
| **Northern Mariana Islands** | 8406255(7029453 to 10029778) | 30244(3753 to 121113) | 170224(23519 to 724173) | 8606724(7238054 to 10139618) |  | -68.7(-75.0 to -61.5) | - | 5.3(-85.1 to 1478.6) | -68.2(-74.4 to -60.9) |  | -18.2(-29.2 to -6.1) | 10.0(-81.2 to 314.8) | | -28.1(-87.6 to 172.1) | -18.4(-28.3 to -6.9) |
| **Norway** | 695824(607160 to 769929) | 8090(1894 to 21587) | 32247(7918 to 82465) | 736161(668232 to 798820) |  | -56.6(-59.4 to -53.3) | - | -18.9(-75.0 to 268.4) | -55.1(-57.2 to -53.0) |  | -26.7(-33.6 to -20.4) | -2.1(-69.7 to 165.2) | | -37.3(-80.7 to 70.8) | -27.0(-30.6 to -22.7) |
| **Oman** | 3140547(2481613 to 3848192) | 8650(1492 to 28099) | 124616(22987 to 389293) | 3273813(2634585 to 3966942) |  | -71.3(-79.9 to -60.1) | - | -43.1(-84.9 to 261.4) | -70.7(-79.6 to -59.2) |  | -45.6(-53.2 to -37.3) | -11.7(-82.2 to 171.2) | | -41.0(-88.1 to 80.4) | -45.4(-52.4 to -37.3) |
| **Pakistan** | 147844445(113533803 to 189641497) | 900148(231128 to 2404994) | 17840963(4766734 to 45698994) | 166585555(136159848 to 210835182) |  | -38.2(-51.7 to -20.8) | - | 4901.5(970.8 to 53280.4) | -32.2(-46.3 to -13.8) |  | -33.0(-48.9 to -15.1) | 28.6(-67.0 to 369.9) | | -13.8(-78.2 to 210.8) | -31.1(-42.9 to -16.6) |
| **Palau** | 14046371(11279457 to 17190606) | 16068(1074 to 77178) | 87710(6884 to 434364) | 14150149(11399518 to 17329954) |  | -43.6(-55.9 to -27.4) | - | 87.4(-88.1 to 2087.0) | -43.4(-55.7 to -26.9) |  | -17.5(-30.5 to -2.3) | 43.9(-77.4 to 789.9) | | -7.0(-85.2 to 481.7) | -17.4(-30.3 to -2.2) |
| **Palestine** | 3088045(2277966 to 3781947) | 9276(871 to 40684) | 132008(14085 to 537461) | 3229329(2493591 to 3904901) |  | -66.0(-78.4 to -54.3) | - | 192.3(-69.1 to 3975.4) | -64.8(-77.3 to -52.9) |  | -32.4(-44.0 to -15.3) | 21.5(-75.1 to 511.6) | | -20.0(-83.7 to 305.6) | -31.8(-42.5 to -15.6) |
| **Panama** | 17307434(12640364 to 22822312) | 133039(13454 to 502240) | 830096(83575 to 3259129) | 18270569(13945686 to 23911421) |  | -47.2(-55.8 to -40.2) | - | 670.0(-7.6 to 7554.5) | -44.4(-49.7 to -38.5) |  | -29.2(-46.5 to -5.0) | 0.5(-78.4 to 343.6) | | -34.2(-86.0 to 191.1) | -29.2(-45.8 to -7.1) |
| **Papua New Guinea** | 83865231(55849990 to 115766157) | 1240666(290267 to 3220448) | 6607746(1596034 to 16615139) | 91713643(61737919 to 126956400) |  | -36.5(-46.5 to -24.8) | - | 1831.7(299.1 to 18383.9) | -34.7(-44.8 to -22.8) |  | -28.8(-41.3 to -14.6) | 221.8(-19.3 to 1169.6) | | 108.6(-48.1 to 726.0) | -24.4(-35.6 to -11.3) |
| **Paraguay** | 13515503(9783327 to 18009024) | 145665(15371 to 503412) | 905731(99651 to 3098009) | 14566898(11207298 to 18763252) |  | -43.8(-51.6 to -35.0) | - | 544.2(13.7 to 6290.0) | -41.0(-48.4 to -32.2) |  | -26.6(-45.0 to -4.4) | 54.2(-74.7 to 371.0) | | 0.6(-83.5 to 205.5) | -24.9(-41.9 to -3.5) |
| **Peru** | 18834419(13590060 to 25579473) | 488588(163577 to 1031385) | 3045261(1100618 to 6383477) | 22368267(16918045 to 29603247) |  | -85.2(-88.3 to -81.6) | - | 44.4(-51.6 to 531.6) | -82.3(-85.3 to -78.6) |  | -41.7(-59.0 to -19.9) | -27.6(-63.6 to 43.9) | | -52.5(-76.1 to -6.5) | -43.2(-59.2 to -23.8) |
| **Philippines** | 111486355(90958468 to 132592148) | 1144678(262431 to 3040721) | 6163968(1415436 to 16152428) | 118795001(100394108 to 140858529) |  | -48.1(-54.7 to -38.7) | - | 2173.0(476.1 to 22468.3) | -44.7(-50.7 to -35.3) |  | -19.7(-34.1 to -4.1) | 12.8(-70.8 to 223.4) | | -26.2(-81.0 to 111.3) | -19.8(-33.9 to -5.1) |
| **Poland** | 3000126(2471607 to 3597950) | 19250(5747 to 50126) | 43905(13423 to 112293) | 3063281(2538016 to 3658771) |  | -68.6(-70.0 to -67.0) | - | -41.3(-80.6 to 171.4) | -68.2(-69.6 to -66.8) |  | -33.0(-43.9 to -20.1) | -10.3(-68.1 to 133.4) | | -43.7(-79.9 to 43.8) | -33.0(-43.9 to -20.2) |
| **Portugal** | 2696150(2394073 to 2989340) | 19592(3636 to 61725) | 77318(15101 to 239914) | 2793060(2524649 to 3073108) |  | -67.6(-70.1 to -64.8) | - | -56.9(-84.4 to 50.0) | -67.1(-69.4 to -64.7) |  | -33.3(-39.7 to -26.3) | -8.4(-76.6 to 148.1) | | -40.9(-85.0 to 60.2) | -33.4(-39.0 to -26.9) |
| **Puerto Rico** | 1107967(866138 to 1396426) | 4175(619 to 13290) | 27370(4578 to 92389) | 1139512(898134 to 1435833) |  | -80.7(-83.1 to -78.1) | - | 84.0(-59.2 to 1395.9) | -79.9(-82.2 to -77.3) |  | -18.9(-34.4 to 2.4) | -23.4(-82.7 to 161.9) | | -50.6(-88.7 to 67.2) | -20.1(-34.8 to -0.3) |
| **Qatar** | 2882576(2275899 to 3632500) | 8449(1393 to 28838) | 121115(21191 to 393992) | 3012140(2415604 to 3741330) |  | -69.6(-75.6 to -60.5) | - | 116.1(-55.1 to 2367.7) | -68.7(-74.9 to -59.4) |  | -40.3(-51.9 to -26.4) | 16.3(-73.7 to 306.4) | | -22.5(-82.6 to 170.8) | -39.7(-50.9 to -26.5) |
| **Republic of Korea** | 6958917(6034082 to 7765286) | 48495(5817 to 199464) | 189810(22293 to 758743) | 7197222(6495182 to 7984757) |  | -83.7(-85.1 to -82.2) | - | -55.3(-91.4 to 219.7) | -83.3(-84.5 to -81.9) |  | -39.5(-45.6 to -33.7) | -23.5(-85.7 to 163.2) | | -50.9(-90.8 to 69.1) | -39.8(-44.6 to -34.7) |
| **Republic of Moldova** | 7371006(4186765 to 11207324) | 3265402(2064108 to 4632656) | 7604156(5152669 to 10378630) | 18240564(15713652 to 20851680) |  | -3.0(-33.6 to 29.1) | - | 3167.5(792.1 to 28614.3) | 89.7(73.7 to 106.6) |  | -66.8(-77.5 to -53.4) | -31.8(-50.1 to -8.5) | | -56.4(-68.2 to -41.4) | -59.0(-64.9 to -53.1) |
| **Romania** | 13587598(10947425 to 16524639) | 375493(114965 to 844562) | 848318(270086 to 1895436) | 14811408(12246714 to 17740089) |  | -29.2(-38.1 to -22.4) | - | 129.3(-35.6 to 792.1) | -24.8(-29.1 to -19.9) |  | -38.9(-50.9 to -23.7) | -7.0(-76.0 to 231.7) | | -41.5(-85.0 to 108.6) | -38.5(-48.9 to -26.3) |
| **Russian Federation** | 11370483(7105195 to 15609536) | 2438546(1173169 to 4004602) | 5554816(2812425 to 8898246) | 19363844(16610774 to 22384411) |  | 5.7(-29.8 to 40.1) | - | 2104.7(591.7 to 8948.6) | 70.0(63.9 to 85.9) |  | -57.6(-68.9 to -43.2) | -35.2(-57.5 to 3.3) | | -59.1(-73.4 to -35.3) | -56.1(-62.3 to -49.7) |
| **Rwanda** | 168590101(124169630 to 220886507) | 157156(44665 to 405902) | 11746287(3405372 to 29373565) | 180493544(134042381 to 235763906) |  | -71.2(-78.1 to -63.0) | - | 1492.5(246.6 to 15429.4) | -69.7(-76.6 to -61.6) |  | -36.7(-49.4 to -22.9) | 28.1(-67.3 to 415.1) | | -13.8(-78.2 to 247.4) | -35.5(-46.9 to -22.4) |
| **Saint Kitts and Nevis** | 5527632(3812500 to 7197870) | 9246(687 to 47542) | 58065(4860 to 267526) | 5594943(3871259 to 7280734) |  | -73.4(-79.1 to -68.2) | - | -81.7(-98.7 to 84.3) | -73.5(-79.1 to -68.5) |  | -26.7(-41.9 to -11.1) | 75.3(-67.9 to 792.2) | | 13.5(-79.3 to 478.6) | -26.3(-41.7 to -11.0) |
| **Saint Lucia** | 7660243(6444868 to 9206381) | 11245(1193 to 48116) | 70113(7187 to 294153) | 7741602(6491611 to 9299882) |  | -67.0(-70.4 to -62.5) | - | -87.2(-98.9 to 92.6) | -67.3(-70.5 to -63.6) |  | -21.8(-34.1 to -7.5) | 59.6(-71.2 to 638.9) | | 3.5(-81.3 to 379.2) | -21.6(-34.1 to -7.3) |
| **Saint Vincent and the Grenadines** | 7789517(6580312 to 9118559) | 13143(1105 to 54577) | 83574(7151 to 341511) | 7886234(6683530 to 9218466) |  | -59.8(-64.9 to -52.9) | - | -88.1(-98.9 to 117.6) | -60.4(-65.2 to -55.3) |  | -15.8(-29.3 to -1.3) | 87.2(-66.7 to 828.6) | | 22.2(-78.5 to 501.3) | -15.4(-27.6 to -0.9) |
| **Samoa** | 28723407(21526367 to 38337027) | 7132(589 to 28596) | 38647(3189 to 153843) | 28769186(21546313 to 38439179) |  | -43.5(-57.8 to -24.1) | - | 51.2(-86.2 to 1707.2) | -43.4(-57.7 to -24.1) |  | -17.5(-32.3 to -1.2) | -17.8(-84.0 to 426.0) | | -46.9(-89.6 to 239.6) | -17.6(-32.3 to -1.3) |
| **San Marino** | 670240(470230 to 931388) | 3502(372 to 13553) | 14207(1557 to 53570) | 687948(495496 to 950249) |  | -61.8(-71.9 to -50.2) | - | -6.7(-90.2 to 1051.6) | -61.1(-70.9 to -49.4) |  | -14.8(-34.8 to 10.7) | 26.1(-76.9 to 532.2) | | -20.2(-85.3 to 295.7) | -14.7(-33.7 to 10.7) |
| **Sao Tome and Principe** | 49041806(36249088 to 67094429) | 50433(5618 to 171641) | 3828450(418795 to 12568771) | 52920689(40654490 to 71179813) |  | -42.3(-56.5 to -21.1) | - | 448.2(-35.5 to 6928.7) | -38.5(-51.2 to -15.9) |  | -30.4(-44.4 to -11.9) | 12.8(-76.0 to 367.4) | | -25.0(-84.0 to 212.2) | -30.0(-41.2 to -15.1) |
| **Saudi Arabia** | 17553210(13490137 to 22499028) | 77060(11867 to 257456) | 1063666(187110 to 3480652) | 18693935(15077881 to 23690848) |  | -59.1(-75.5 to -33.6) | - | 932.5(139.6 to 12269.5) | -56.7(-73.9 to -30.2) |  | -48.5(-58.8 to -36.2) | -18.1(-83.0 to 129.9) | | -45.8(-88.7 to 53.7) | -48.3(-57.4 to -37.3) |
| **Senegal** | 130830635(97294228 to 165790597) | 92222(19892 to 269942) | 6840201(1575101 to 17293923) | 137763057(103085941 to 173543658) |  | -50.0(-59.9 to -37.4) | - | 1415.4(180.4 to 17873.0) | -47.5(-56.6 to -34.8) |  | -20.1(-37.9 to 0.8) | 23.2(-73.3 to 446.8) | | -18.6(-82.2 to 258.0) | -20.0(-36.1 to -1.7) |
| **Serbia** | 2654409(2124031 to 3282720) | 18912(3873 to 54754) | 43457(10111 to 122712) | 2716779(2163482 to 3377271) |  | -65.0(-69.3 to -57.9) | - | 48.2(-64.5 to 1200.5) | -64.2(-68.5 to -57.1) |  | -38.9(-50.9 to -24.7) | -27.9(-81.2 to 90.4) | | -54.7(-88.2 to 19.5) | -39.2(-50.8 to -25.3) |
| **Seychelles** | 10757786(9396508 to 12343011) | 8592(593 to 43008) | 47784(3286 to 239012) | 10814162(9442171 to 12402313) |  | -44.3(-50.8 to -37.2) | - | 253.2(-74.4 to 4732.9) | -43.9(-50.4 to -36.5) |  | -22.3(-31.2 to -11.3) | -27.9(-90.9 to 342.6) | | -52.8(-94.1 to 192.3) | -22.6(-31.4 to -12.1) |
| **Sierra Leone** | 176692379(126851375 to 243212752) | 119116(12399 to 429079) | 8949760(1036027 to 30930172) | 185761255(136646462 to 255178788) |  | -29.9(-47.9 to -9.3) | - | 205.0(-63.0 to 2523.3) | -27.1(-42.8 to -6.9) |  | -35.0(-48.6 to -16.7) | -3.0(-78.3 to 284.5) | | -35.5(-85.4 to 155.2) | -35.0(-47.3 to -18.2) |
| **Singapore** | 1953835(1708093 to 2241379) | 10225(3231 to 24964) | 41987(14818 to 97656) | 2006048(1763403 to 2291138) |  | -78.7(-80.6 to -76.8) | - | -41.0(-82.5 to 230.1) | -78.3(-80.1 to -76.5) |  | -42.4(-48.0 to -36.2) | -28.9(-70.7 to 57.5) | | -53.6(-81.0 to 3.5) | -42.7(-48.1 to -36.8) |
| **Slovakia** | 1176065(921002 to 1482226) | 6332(1067 to 21806) | 14919(2557 to 52702) | 1197316(942215 to 1498704) |  | -72.3(-76.0 to -68.5) | - | -48.2(-83.9 to 198.6) | -71.9(-75.5 to -68.2) |  | -34.7(-47.1 to -19.5) | -38.6(-87.0 to 89.2) | | -61.4(-91.9 to 19.0) | -35.3(-47.5 to -20.2) |
| **Slovenia** | 914557(723615 to 1141878) | 749(92 to 2808) | 1792(212 to 6834) | 917098(725231 to 1142933) |  | -77.2(-82.5 to -71.0) | - | -81.4(-96.7 to 48.5) | -77.2(-82.4 to -71.1) |  | -39.2(-51.2 to -24.6) | -74.7(-95.2 to -11.8) | | -84.0(-96.9 to -44.8) | -39.6(-51.5 to -25.1) |
| **Solomon Islands** | 41059794(33224530 to 49955892) | 51514(2998 to 204456) | 267104(16520 to 1077255) | 41378412(33601789 to 50109639) |  | -33.8(-48.1 to -16.1) | - | 118.6(-83.5 to 3147.1) | -33.4(-47.9 to -15.6) |  | -26.9(-38.5 to -13.3) | 24.7(-80.9 to 654.9) | | -19.6(-87.6 to 392.3) | -26.8(-38.3 to -13.1) |
| **Somalia** | 627318739(404504269 to 928125357) | 1371760(305778 to 4059202) | 101091759(23053545 to 277892335) | 729782258(527988935 to 1054009228) |  | -24.1(-42.0 to -0.1) | - | 12399.9(3003.6 to 125231.5) | -11.2(-29.5 to 15.5) |  | -15.0(-39.5 to 7.8) | 22.5(-64.4 to 256.2) | | -18.7(-76.3 to 137.1) | -15.5(-29.0 to 1.3) |
| **South Africa** | 149414407(130684393 to 167432487) | 111483(30918 to 301887) | 8454505(2508490 to 20826628) | 157980395(143514878 to 174045531) |  | -9.9(-23.6 to 2.9) | - | 494.9(73.0 to 3347.4) | -5.0(-18.0 to 7.9) |  | -45.8(-51.6 to -39.4) | -29.3(-80.1 to 135.0) | | -52.0(-86.6 to 58.0) | -46.1(-50.3 to -41.7) |
| **South Sudan** | 271558877(183712686 to 370381942) | 326427(38532 to 962987) | 24290575(3052869 to 68483251) | 296175880(213295092 to 398564302) |  | -34.0(-51.3 to -15.3) | - | 2462.9(218.1 to 30568.4) | -29.4(-44.4 to -10.7) |  | -16.1(-33.3 to 4.0) | 56.1(-60.5 to 545.0) | | 4.4(-73.6 to 329.9) | -14.7(-28.5 to 2.6) |
| **Spain** | 948992(836860 to 1046706) | 5591(595 to 23727) | 21937(2533 to 91227) | 976521(886991 to 1067024) |  | -79.0(-80.9 to -77.1) | - | -62.6(-93.4 to 144.0) | -78.7(-80.1 to -77.1) |  | -31.5(-38.2 to -24.6) | -18.5(-82.8 to 183.4) | | -48.2(-89.0 to 82.3) | -32.0(-37.1 to -26.6) |
| **Sri Lanka** | 12250574(9532506 to 15514611) | 16561(1190 to 75052) | 92001(7055 to 406464) | 12359136(9670008 to 15703495) |  | -66.0(-69.6 to -62.0) | - | 159.0(-74.6 to 3458.5) | -65.6(-69.3 to -61.8) |  | -35.4(-48.8 to -18.8) | -31.3(-88.4 to 271.1) | | -54.2(-92.3 to 145.1) | -35.6(-49.0 to -19.8) |
| **Sudan** | 16008132(9839019 to 22975172) | 48705(4720 to 214053) | 681280(65298 to 2922268) | 16738117(10376009 to 23840384) |  | -66.6(-75.6 to -55.1) | - | 205.2(-70.4 to 4143.2) | -65.3(-74.3 to -53.4) |  | -42.7(-52.7 to -29.0) | -9.7(-80.9 to 336.5) | | -40.0(-87.4 to 189.3) | -42.6(-51.9 to -29.8) |
| **Suriname** | 6861146(5599954 to 8205673) | 8571(812 to 31914) | 54113(5325 to 210569) | 6923829(5673748 to 8246533) |  | -56.5(-62.0 to -47.2) | - | -67.3(-97.1 to 262.0) | -56.6(-61.8 to -47.4) |  | -19.6(-33.3 to -4.1) | 59.8(-70.5 to 833.9) | | 4.5(-80.8 to 508.4) | -19.4(-33.5 to -3.7) |
| **Sweden** | 610422(518889 to 709221) | 8084(2434 to 20327) | 33665(10339 to 79906) | 652171(563133 to 747411) |  | -64.1(-68.0 to -60.0) | - | 66.9(-50.9 to 800.6) | -61.9(-65.6 to -58.1) |  | -24.4(-33.7 to -15.6) | -3.2(-66.1 to 124.9) | | -36.6(-78.0 to 47.2) | -25.0(-31.8 to -17.7) |
| **Switzerland** | 483857(396494 to 583488) | 7872(2415 to 17669) | 33434(10907 to 71426) | 525162(445503 to 618104) |  | -72.8(-75.8 to -69.1) | - | -7.1(-73.4 to 511.7) | -71.9(-75.0 to -68.3) |  | -26.8(-37.5 to -16.1) | 99.7(-24.2 to 340.8) | | 29.8(-51.3 to 186.5) | -23.9(-32.0 to -13.9) |
| **Syrian Arab Republic** | 2663975(2003690 to 3420850) | 9497(1145 to 38345) | 137610(17115 to 528322) | 2811083(2212323 to 3527133) |  | -76.4(-81.2 to -69.4) | - | 209.4(-57.6 to 4459.6) | -75.0(-79.7 to -68.1) |  | -15.1(-31.8 to 5.8) | 11.2(-76.0 to 316.9) | | -27.2(-84.4 to 172.9) | -15.7(-30.7 to 3.1) |
| **Taiwan (Province of China)** | 6427117(5106672 to 7779612) | 37738(3770 to 140851) | 230740(24594 to 866084) | 6695595(5396886 to 8145625) |  | -78.7(-82.0 to -76.0) | - | -46.4(-94.8 to 592.2) | -78.1(-80.3 to -76.0) |  | -20.6(-32.9 to -4.6) | 5.9(-78.5 to 426.7) | | -29.0(-85.5 to 257.8) | -20.8(-31.4 to -8.2) |
| **Tajikistan** | 31007744(20170613 to 41986113) | 4089057(1554065 to 7869653) | 9171653(3682264 to 16585833) | 44268455(36778320 to 53837350) |  | -10.4(-36.5 to 15.3) | - | 17974.7(4024.7 to 171025.1) | 20.1(3.9 to 38.7) |  | -31.8(-55.5 to -0.2) | 20.3(-53.4 to 200.9) | | -24.6(-71.1 to 88.2) | -27.5(-41.0 to -10.2) |
| **Thailand** | 18313478(14008831 to 23364172) | 159908(33934 to 446834) | 879077(194191 to 2438979) | 19352462(15208318 to 24392809) |  | -76.5(-80.3 to -72.0) | - | -11.6(-73.8 to 434.7) | -75.4(-79.1 to -70.8) |  | -31.3(-46.8 to -12.4) | -9.4(-77.9 to 157.7) | | -40.1(-85.4 to 68.0) | -31.6(-46.6 to -12.7) |
| **Timor-Leste** | 106747223(64357806 to 143396514) | 202763(18548 to 958956) | 1072886(102331 to 5013319) | 108022872(66249512 to 144887344) |  | -64.6(-76.6 to -51.5) | - | 68.6(-85.5 to 2381.1) | -63.9(-76.0 to -50.8) |  | -9.3(-25.4 to 13.2) | -30.1(-86.8 to 256.2) | | -54.5(-91.4 to 130.5) | -10.3(-25.8 to 10.9) |
| **Togo** | 151319841(108307052 to 208502608) | 113641(13557 to 363326) | 8473566(1065825 to 26300910) | 159907047(115349946 to 218602984) |  | -24.1(-44.4 to -2.1) | - | 450.8(-27.0 to 7238.0) | -19.9(-38.3 to 2.7) |  | -32.3(-46.2 to -15.2) | -11.8(-78.6 to 278.3) | | -41.4(-85.8 to 152.1) | -32.9(-45.1 to -18.9) |
| **Tokelau** | 18478245(14378006 to 23853656) | 20746(1532 to 96702) | 114582(8713 to 585729) | 18613574(14503470 to 24061690) |  | -63.1(-71.3 to -53.0) | - | 14.9(-90.1 to 1905.5) | -63.0(-71.1 to -52.3) |  | -32.8(-42.7 to -20.7) | 16.9(-79.7 to 517.0) | | -22.8(-86.5 to 302.2) | -32.7(-42.4 to -20.8) |
| **Tonga** | 18166124(14376718 to 22427393) | 19338(1576 to 85036) | 107463(9373 to 482814) | 18292925(14530804 to 22590739) |  | -40.6(-53.3 to -24.1) | - | 86.1(-85.0 to 2674.8) | -40.4(-53.2 to -24.1) |  | -20.8(-32.8 to -5.9) | 39.6(-76.8 to 653.5) | | -7.8(-84.9 to 388.8) | -20.7(-32.5 to -6.3) |
| **Trinidad and Tobago** | 4085834(3116891 to 5253886) | 6741(547 to 31551) | 42439(3803 to 173157) | 4135014(3158052 to 5286134) |  | -47.6(-53.2 to -41.4) | - | -62.0(-96.2 to 316.2) | -47.7(-53.2 to -41.7) |  | -24.3(-41.7 to -3.6) | 64.9(-71.2 to 803.7) | | 6.1(-81.2 to 485.5) | -24.0(-41.4 to -3.7) |
| **Tunisia** | 3158354(2381207 to 4092444) | 5714(1289 to 17287) | 83167(20830 to 243164) | 3247235(2455726 to 4248499) |  | -66.2(-73.6 to -56.9) | - | 347.7(-30.9 to 5125.5) | -65.4(-72.8 to -55.8) |  | -33.3(-45.3 to -18.4) | 13.4(-71.6 to 397.8) | | -25.2(-81.1 to 227.5) | -33.1(-45.0 to -18.6) |
| **Turkey** | 3464822(2810840 to 4208836) | 17925(6227 to 40731) | 264907(99320 to 571548) | 3747655(3089864 to 4502580) |  | -85.5(-88.1 to -82.3) | - | 371.1(3.7 to 4452.4) | -84.5(-87.2 to -81.1) |  | -41.5(-51.6 to -29.7) | -3.3(-62.9 to 148.7) | | -35.3(-75.2 to 65.6) | -41.0(-50.8 to -29.6) |
| **Turkmenistan** | 28924816(16275787 to 40726415) | 4181259(1413484 to 8333985) | 9390034(3414916 to 18061326) | 42496110(33737558 to 52793888) |  | -35.3(-54.4 to -20.8) | - | 12077.8(2950.6 to 125759.6) | -11.2(-19.3 to -2.2) |  | -35.9(-63.3 to -1.4) | 13.9(-59.9 to 186.6) | | -28.7(-75.2 to 78.8) | -31.4(-46.0 to -13.5) |
| **Tuvalu** | 34959682(26456372 to 45097935) | 39866(2892 to 184716) | 215484(16240 to 922182) | 35215032(26603295 to 45397534) |  | -63.0(-70.6 to -53.7) | - | 15.9(-91.9 to 1641.5) | -62.9(-70.3 to -53.5) |  | -27.5(-40.1 to -13.6) | 20.4(-79.2 to 768.2) | | -20.5(-86.4 to 463.5) | -27.4(-39.8 to -13.8) |
| **Uganda** | 225256998(162690955 to 298394279) | 226845(36853 to 694135) | 16853020(2853796 to 48193138) | 242336863(182558433 to 312525195) |  | -36.1(-50.4 to -16.8) | - | 3453.6(756.5 to 40649.8) | -32.9(-47.8 to -12.7) |  | -29.9(-44.4 to -11.8) | 52.1(-68.6 to 315.8) | | 1.7(-79.2 to 177.0) | -28.3(-41.6 to -12.4) |
| **Ukraine** | 15577494(8476990 to 23870090) | 4711926(2284894 to 7440640) | 10687939(5459742 to 16596348) | 30977359(25456908 to 37829181) |  | 9.8(-32.2 to 48.5) | - | 3200.0(784.7 to 28751.2) | 77.7(66.5 to 88.7) |  | -18.6(-57.8 to 40.8) | 75.9(-13.4 to 275.3) | | 10.6(-46.2 to 135.4) | -1.6(-19.7 to 21.1) |
| **United Arab Emirates** | 7180906(4150810 to 10276825) | 20690(1874 to 79488) | 296305(27141 to 1109345) | 7497901(4321550 to 10536208) |  | -55.4(-66.4 to -43.0) | - | 278.0(-62.7 to 4280.3) | -54.0(-64.6 to -41.4) |  | -35.7(-48.5 to -21.2) | 13.7(-78.7 to 500.1) | | -24.1(-85.7 to 295.7) | -35.2(-46.6 to -21.6) |
| **United Kingdom** | 892648(821556 to 972759) | 5255(1624 to 12990) | 21219(6911 to 48816) | 919121(849464 to 999563) |  | -51.6(-53.8 to -49.1) | - | -37.9(-75.1 to 68.7) | -51.1(-53.1 to -49.0) |  | -21.6(-25.4 to -17.7) | 14.9(-56.6 to 153.7) | | -27.0(-72.6 to 59.9) | -21.6(-24.5 to -18.0) |
| **United Republic of Tanzania** | 206182077(156317451 to 267514729) | 162075(20975 to 608840) | 12099440(1662127 to 44058707) | 218443592(168408104 to 281165458) |  | -42.3(-53.6 to -28.6) | - | 1673.3(208.9 to 23425.5) | -40.3(-52.0 to -26.3) |  | -26.9(-41.1 to -10.2) | 86.8(-56.2 to 419.1) | | 24.6(-70.8 to 248.6) | -25.2(-38.6 to -9.4) |
| **United States of America** | 531184(483071 to 580979) | 3470(1068 to 8689) | 13823(4445 to 33964) | 548477(503110 to 596436) |  | -71.2(-72.8 to -69.1) | - | -89.2(-93.6 to -79.9) | -72.3(-73.5 to -70.8) |  | -8.7(-13.6 to -4.4) | 45.6(-41.3 to 211.9) | | -7.2(-62.5 to 99.0) | -8.5(-12.3 to -4.7) |
| **United States Virgin Islands** | 2140289(1695304 to 2693357) | 2988(307 to 12064) | 19341(2033 to 79735) | 2162619(1718152 to 2708417) |  | -57.3(-65.1 to -47.1) | - | -72.4(-97.3 to 236.4) | -57.5(-65.0 to -47.5) |  | -10.5(-25.3 to 6.1) | 93.3(-64.7 to 915.4) | | 23.6(-77.3 to 552.5) | -10.2(-24.6 to 6.6) |
| **Uruguay** | 3936715(3462060 to 4390040) | 6366(1174 to 20481) | 25207(4839 to 80026) | 3968289(3509019 to 4429666) |  | -66.8(-69.7 to -64.0) | - | -14.2(-87.1 to 694.4) | -66.7(-69.4 to -63.8) |  | -12.3(-22.2 to -2.5) | 53.9(-70.0 to 560.6) | | -1.1(-80.7 to 322.6) | -12.1(-21.9 to -2.6) |
| **Uzbekistan** | 20903019(10394587 to 31812945) | 4733819(1820452 to 8760311) | 10633869(4214369 to 18048059) | 36270707(30346691 to 42949543) |  | -33.2(-55.2 to -11.8) | - | 23747.1(6400.0 to 316284.0) | 23.7(14.4 to 33.2) |  | -35.1(-65.4 to 1.1) | -21.1(-66.1 to 32.8) | | -50.7(-79.1 to -16.7) | -39.3(-49.1 to -27.9) |
| **Vanuatu** | 70441813(50440220 to 92767546) | 50618(3197 to 229497) | 271293(18715 to 1248051) | 70763724(50748714 to 93124701) |  | -32.3(-49.6 to -10.3) | - | 145.8(-85.1 to 2404.6) | -32.1(-49.5 to -10.3) |  | -16.8(-30.6 to -0.5) | 86.8(-68.1 to 1141.6) | | 21.1(-79.3 to 707.2) | -16.7(-30.7 to -0.5) |
| **Venezuela (Bolivarian Republic of)** | 9168142(6724079 to 12009448) | 56847(5869 to 202333) | 353951(39279 to 1259734) | 9578941(7306116 to 12522697) |  | -67.7(-71.7 to -64.5) | - | 263.8(-53.9 to 4859.2) | -66.3(-68.6 to -63.9) |  | -3.7(-28.0 to 27.4) | 35.0(-73.1 to 517.5) | | -12.3(-82.4 to 303.4) | -3.9(-27.2 to 25.6) |
| **Viet Nam** | 50081583(38782613 to 61567093) | 772824(185858 to 1957067) | 4138649(1041643 to 10226147) | 54993056(44508399 to 66321165) |  | -59.3(-70.0 to -43.6) | - | 306.2(9.6 to 2353.8) | -55.0(-66.5 to -38.6) |  | -35.0(-47.7 to -20.7) | -20.7(-78.5 to 105.9) | | -48.3(-85.9 to 35.0) | -36.1(-46.1 to -24.3) |
| **Yemen** | 20309632(14494517 to 29356379) | 68378(11396 to 234574) | 955316(152226 to 3290933) | 21333327(15464572 to 30684176) |  | -66.9(-77.0 to -54.9) | - | 395.2(-2.2 to 5849.0) | -65.2(-76.1 to -52.7) |  | -13.6(-29.1 to 5.4) | 16.2(-74.1 to 314.5) | | -23.1(-82.9 to 173.7) | -14.0(-27.9 to 2.9) |
| **Zambia** | 244570992(172574369 to 320935384) | 261144(36386 to 870850) | 19395725(2867396 to 63807764) | 264227861(194329738 to 341874406) |  | -33.7(-45.4 to -18.0) | - | 546.5(38.6 to 5891.2) | -31.5(-43.6 to -14.2) |  | -46.8(-59.5 to -32.7) | 76.6(-61.0 to 399.1) | | 17.9(-74.0 to 233.7) | -44.5(-55.0 to -31.4) |
| **Zimbabwe** | 363350688(254331217 to 472354784) | 540715(67963 to 1796885) | 39934595(5144987 to 122157457) | 403825998(308810954 to 504650550) |  | 83.9(31.9 to 138.8) | - | 2600.4(197.4 to 37341.4) | 101.0(56.3 to 156.0) |  | -35.1(-50.5 to -14.7) | 6.9(-73.3 to 350.1) | | -28.3(-82.3 to 200.1) | -34.5(-45.7 to -21.5) |

| **S6** | | | | | | | | | | | | | | | |
| --- | --- | --- | --- | --- | --- | --- | --- | --- | --- | --- | --- | --- | --- | --- | --- |
| **Group** | **Number of Desths (age-standardized, both sexes) with 95% uncertainty intervals (2019)** | | | |  | **Annualized rate of change of age-standardized rate (%) with 95% uncertainty intervals** | | | | | | | | | |
|  |  |  |  |  |  | **1990-2010** | | | |  | **2010-2019** | | | | |
|  | **Drug-susceptible tuberculosis** | **Extensively drug-resistant tuberculosis** | **Multidrug-resistant tuberculosis without extensive drug resistance** | **Tuberculosis** |  | **Drug-susceptible tuberculosis** | **Extensively drug-resistant tuberculosis** | **Multidrug-resistant tuberculosis without extensive drug resistance** | **Tuberculosis** |  | **Drug-susceptible tuberculosis** | **Extensively drug-resistant tuberculosis** | **Multidrug-resistant tuberculosis without extensive drug resistance** | | **Tuberculosis** |
| **Global** | 1317296(1147331 to 1473409) | 10419(4901 to 18734) | 135906(54012 to 258625) | 1463621(1339240 to 1602711) |  | -53.0(-57.3 to -48.3) | - | 348.4(114.3 to 929.2) | -48.9(-52.0 to -44.4) |  | -29.4(-36.3 to -22.3) | -5.6(-32.8 to 37.6) | | -21.6(-52.4 to 27.5) | -28.6(-34.4 to -22.2) |
| **Afghanistan** | 1973937(1149543 to 2739992) | 15080(1969 to 53058) | 202245(30596 to 654914) | 2191262(1344133 to 2977873) |  | -50.2(-62.0 to -33.7) | - | 2556.2(475.9 to 31887.6) | -42.4(-54.1 to -26.3) |  | -31.9(-49.7 to -14.1) | -30.2(-86.7 to 126.1) | | -54.2(-91.2 to 49.0) | -34.8(-45.4 to -21.5) |
| **Albania** | 26914(20466 to 35149) | 187(28 to 561) | 411(62 to 1242) | 27511(21329 to 35740) |  | -86.0(-88.4 to -82.7) | - | -5.9(-80.6 to 752.2) | -85.5(-87.9 to -82.1) |  | -13.1(-33.4 to 11.7) | -34.4(-87.0 to 106.1) | | -58.9(-91.9 to 28.1) | -14.7(-34.5 to 9.5) |
| **Algeria** | 146041(112009 to 182977) | 510(39 to 2398) | 6908(530 to 31548) | 153459(124667 to 187584) |  | -70.5(-77.3 to -61.9) | - | -61.4(-94.6 to 103.6) | -70.2(-76.4 to -61.7) |  | -34.9(-47.9 to -17.7) | 23.0(-77.5 to 320.4) | | -18.8(-85.2 to 177.0) | -34.2(-46.8 to -18.0) |
| **American Samoa** | 149790(125578 to 176797) | 192(15 to 834) | 1012(82 to 4490) | 150994(126165 to 177925) |  | -57.9(-64.3 to -49.7) | - | 28.7(-91.6 to 1649.0) | -57.7(-64.2 to -49.6) |  | -16.6(-29.3 to -1.1) | 48.2(-73.6 to 775.8) | | -3.3(-82.9 to 466.3) | -16.4(-29.1 to -1.4) |
| **Andorra** | 18185(13904 to 22882) | 25(2 to 112) | 93(9 to 465) | 18303(14005 to 23042) |  | -62.3(-73.5 to -48.2) | - | -44.9(-95.2 to 502.8) | -62.2(-73.2 to -48.2) |  | -11.3(-28.8 to 9.8) | 3.5(-84.2 to 521.8) | | -33.5(-89.9 to 297.6) | -11.4(-28.6 to 9.8) |
| **Angola** | 7371286(5613571 to 9342051) | 6856(670 to 25500) | 504844(49540 to 1734339) | 7882985(6404055 to 9866391) |  | -45.1(-60.2 to -26.1) | - | 423.4(-45.6 to 6493.4) | -42.0(-55.0 to -22.4) |  | -39.4(-51.4 to -24.0) | -0.4(-78.4 to 349.3) | | -33.7(-85.6 to 198.9) | -39.0(-49.0 to -25.9) |
| **Antigua and Barbuda** | 38514(31981 to 46748) | 88(7 to 423) | 533(41 to 2552) | 39135(32750 to 47353) |  | -62.4(-66.2 to -58.1) | - | -78.5(-98.0 to 134.9) | -62.7(-66.1 to -58.9) |  | -21.9(-35.5 to -5.7) | 66.2(-68.9 to 990.0) | | 7.7(-79.8 to 607.7) | -21.6(-34.8 to -5.3) |
| **Argentina** | 136515(119557 to 152249) | 1081(126 to 4105) | 4109(464 to 15603) | 141705(129572 to 155656) |  | -67.7(-70.6 to -65.1) | - | 61.8(-66.4 to 1194.4) | -66.4(-68.7 to -64.1) |  | -16.9(-26.9 to -6.7) | -7.3(-80.4 to 176.5) | | -40.4(-87.5 to 76.9) | -17.8(-25.5 to -9.1) |
| **Armenia** | 132359(65530 to 191809) | 19517(4306 to 41607) | 42677(9827 to 90346) | 194554(161717 to 232790) |  | -23.6(-48.5 to -2.1) | - | 12667.3(2799.2 to 161881.4) | 13.1(3.8 to 24.2) |  | -49.5(-69.5 to -28.9) | -30.7(-76.7 to 31.9) | | -56.8(-85.5 to -18.0) | -50.0(-58.4 to -40.2) |
| **Australia** | 19560(14681 to 25373) | 357(101 to 870) | 1359(411 to 3176) | 21277(16583 to 27200) |  | -56.8(-62.7 to -51.0) | - | 75.7(-61.8 to 969.5) | -55.2(-60.5 to -49.6) |  | -11.0(-28.2 to 7.6) | 140.0(-32.8 to 706.8) | | 53.7(-57.0 to 416.2) | -7.5(-22.7 to 10.9) |
| **Austria** | 25634(22018 to 28821) | 459(134 to 1131) | 1744(517 to 4210) | 27837(25119 to 30622) |  | -80.1(-82.4 to -77.6) | - | 66.2(-55.0 to 1334.7) | -78.1(-80.1 to -75.8) |  | -17.1(-28.0 to -6.4) | -8.5(-64.7 to 95.4) | | -41.3(-77.4 to 25.0) | -19.0(-26.4 to -10.8) |
| **Azerbaijan** | 383019(210043 to 559662) | 68350(20582 to 133658) | 148467(46902 to 278945) | 599837(493599 to 735783) |  | -55.6(-70.1 to -42.0) | - | 10970.1(2500.1 to 125638.6) | -36.0(-44.4 to -25.8) |  | -38.2(-65.8 to 0.9) | 12.6(-64.5 to 176.4) | | -30.4(-78.0 to 71.7) | -32.9(-44.1 to -19.3) |
| **Bahamas** | 179834(140335 to 222870) | 1008(115 to 3956) | 6079(710 to 23667) | 186920(149576 to 230009) |  | -63.0(-68.2 to -57.2) | - | 332.4(-22.0 to 4531.5) | -62.1(-67.3 to -56.2) |  | -16.3(-34.8 to 3.0) | 79.9(-73.1 to 751.4) | | 17.0(-82.5 to 453.0) | -15.3(-31.8 to 4.3) |
| **Bahrain** | 153733(120298 to 191053) | 770(99 to 2667) | 10332(1447 to 32510) | 164836(132944 to 200259) |  | -62.7(-69.7 to -53.8) | - | 710.4(73.9 to 9148.8) | -60.3(-66.7 to -51.6) |  | -42.6(-54.9 to -29.2) | -9.5(-87.0 to 231.0) | | -40.5(-91.4 to 118.1) | -42.3(-52.5 to -31.1) |
| **Bangladesh** | 2071387(1541282 to 2868296) | 9221(1879 to 26699) | 178447(36635 to 476332) | 2259056(1741160 to 3078066) |  | -71.0(-76.0 to -63.2) | - | 1428.9(205.4 to 16683.9) | -69.0(-74.2 to -60.9) |  | -47.1(-58.5 to -33.9) | 0.9(-72.9 to 171.3) | | -33.3(-82.1 to 79.4) | -46.1(-55.8 to -34.1) |
| **Barbados** | 40349(32994 to 48885) | 52(3 to 258) | 309(19 to 1564) | 40709(33345 to 49166) |  | -62.6(-67.0 to -56.8) | - | -91.1(-99.3 to 58.5) | -63.2(-67.2 to -58.9) |  | -14.0(-29.9 to 5.6) | 93.4(-76.0 to 1302.4) | | 24.5(-84.4 to 810.8) | -13.7(-29.9 to 6.2) |
| **Belarus** | 82737(37918 to 140758) | 43464(24436 to 66671) | 94812(56234 to 140444) | 221013(166991 to 290575) |  | -22.1(-51.3 to 8.9) | - | 2872.5(540.5 to 28070.9) | 72.6(59.8 to 86.4) |  | -64.5(-78.6 to -45.6) | -34.0(-55.4 to -6.0) | | -58.9(-72.3 to -41.6) | -58.2(-68.4 to -45.8) |
| **Belgium** | 31910(27549 to 36203) | 316(78 to 912) | 1193(302 to 3493) | 33419(29555 to 37385) |  | -53.4(-57.7 to -48.3) | - | 6.6(-68.2 to 522.1) | -51.8(-55.6 to -47.5) |  | -33.7(-41.7 to -25.4) | -19.0(-73.6 to 100.3) | | -48.3(-83.1 to 28.2) | -34.2(-41.2 to -26.4) |
| **Belize** | 447376(378568 to 521176) | 777(72 to 3247) | 4688(438 to 19764) | 452841(385987 to 527764) |  | -49.1(-55.1 to -42.1) | - | -64.7(-96.4 to 282.2) | -49.2(-54.9 to -42.4) |  | -23.1(-34.8 to -8.9) | 78.6(-66.9 to 922.2) | | 16.3(-78.5 to 564.0) | -22.8(-34.2 to -8.8) |
| **Benin** | 4188464(3085964 to 5633294) | 2628(368 to 8612) | 192141(27234 to 605485) | 4383234(3246577 to 5934089) |  | -43.8(-56.7 to -27.5) | - | 415.0(15.7 to 4087.1) | -41.9(-55.0 to -25.7) |  | -23.1(-37.2 to -8.4) | 45.8(-73.4 to 334.2) | | -3.5(-82.3 to 188.8) | -22.4(-35.0 to -7.4) |
| **Bermuda** | 6383(5140 to 7975) | 12(1 to 76) | 71(4 to 432) | 6466(5254 to 8100) |  | -77.5(-80.4 to -74.4) | - | -89.5(-99.4 to 29.1) | -77.6(-80.4 to -74.6) |  | -27.9(-40.6 to -12.0) | 138.4(-74.0 to 1781.7) | | 58.2(-83.1 to 1122.6) | -27.4(-39.8 to -11.8) |
| **Bhutan** | 1712773(1026495 to 3116939) | 8492(922 to 28659) | 163906(16727 to 529016) | 1885171(1220015 to 3372434) |  | -72.8(-81.7 to -58.7) | - | 228.0(-65.9 to 5042.5) | -70.4(-78.6 to -55.7) |  | -33.8(-48.9 to -15.4) | 2.0(-73.7 to 337.6) | | -32.6(-82.6 to 189.4) | -33.6(-45.1 to -19.3) |
| **Bolivia (Plurinational State of)** | 1478576(974637 to 1976192) | 26063(3726 to 84785) | 157767(22284 to 509745) | 1662406(1243299 to 2118777) |  | -76.9(-83.0 to -70.2) | - | 7.7(-87.5 to 1039.8) | -75.5(-80.6 to -69.3) |  | -37.7(-53.4 to -19.9) | 48.4(-57.3 to 533.0) | | -2.5(-72.3 to 311.2) | -34.9(-47.4 to -20.6) |
| **Bosnia and Herzegovina** | 206301(164226 to 256004) | 883(173 to 2755) | 1924(371 to 5643) | 209107(166803 to 258995) |  | -68.5(-71.8 to -64.7) | - | -36.6(-84.6 to 317.6) | -68.1(-71.5 to -64.4) |  | -30.6(-45.1 to -12.7) | -19.6(-78.6 to 126.0) | | -50.0(-86.7 to 40.4) | -30.8(-45.6 to -12.7) |
| **Botswana** | 4181572(2791710 to 5800459) | 6205(797 to 19279) | 456972(69709 to 1428205) | 4644750(3286480 to 6417785) |  | -28.9(-49.7 to 1.0) | - | 881.2(148.4 to 7933.2) | -24.1(-45.9 to 7.0) |  | -40.3(-56.7 to -22.2) | 32.8(-67.5 to 251.3) | | -11.3(-78.4 to 133.7) | -38.2(-52.0 to -21.7) |
| **Brazil** | 215935(184947 to 236391) | 2225(314 to 6503) | 13415(2096 to 38241) | 231575(219393 to 244926) |  | -64.6(-67.1 to -62.5) | - | 1511.1(243.0 to 19359.3) | -62.7(-64.2 to -61.0) |  | -33.0(-40.6 to -27.0) | 34.7(-66.9 to 231.0) | | -12.4(-78.5 to 115.0) | -31.7(-34.9 to -27.4) |
| **Brunei Darussalam** | 939075(810380 to 1077145) | 1759(213 to 6189) | 6635(794 to 23638) | 947469(819381 to 1083159) |  | -62.1(-67.9 to -55.2) | - | -38.3(-92.3 to 583.7) | -62.0(-67.8 to -55.1) |  | -15.6(-26.6 to -1.8) | 105.6(-69.8 to 1037.7) | | 31.5(-80.7 to 627.9) | -15.3(-26.0 to -1.3) |
| **Bulgaria** | 89926(68002 to 116570) | 1950(447 to 5366) | 4277(962 to 11484) | 96153(74951 to 122778) |  | -43.0(-51.0 to -34.6) | - | 832.4(127.8 to 8502.1) | -36.1(-43.0 to -28.7) |  | -27.0(-44.8 to -4.6) | -43.5(-85.0 to 40.0) | | -64.6(-90.7 to -13.0) | -30.7(-46.6 to -10.5) |
| **Burkina Faso** | 6002821(4654487 to 7371918) | 5550(636 to 17560) | 410079(51719 to 1308035) | 6418451(5253302 to 7807610) |  | -32.5(-46.8 to -17.3) | - | 500.5(-13.6 to 7690.1) | -28.0(-39.3 to -13.5) |  | -9.2(-25.6 to 11.9) | 23.0(-72.1 to 498.9) | | -17.9(-81.4 to 298.4) | -9.8(-22.6 to 7.3) |
| **Burundi** | 14388965(9762703 to 20004475) | 14483(1600 to 53423) | 1063941(122587 to 3813508) | 15467389(10796503 to 21227196) |  | -46.5(-61.3 to -30.4) | - | 1406.6(98.6 to 20189.8) | -43.5(-57.7 to -27.2) |  | -16.4(-33.2 to 1.6) | 65.1(-58.7 to 536.5) | | 9.6(-72.5 to 323.4) | -14.9(-28.0 to 1.3) |
| **Cabo Verde** | 1236952(918905 to 1829056) | 1352(131 to 4595) | 98528(10602 to 323170) | 1336832(1028810 to 2017553) |  | -55.1(-66.0 to -41.5) | - | 297.7(-51.1 to 4899.0) | -52.1(-61.7 to -38.9) |  | -24.9(-39.9 to -0.4) | 19.1(-73.2 to 459.1) | | -21.2(-82.1 to 271.9) | -24.6(-37.0 to -5.8) |
| **Cambodia** | 4938598(3625903 to 6450173) | 15403(1584 to 61360) | 80682(8465 to 305535) | 5034684(3723613 to 6616769) |  | -50.3(-61.9 to -34.2) | - | 732.0(53.8 to 8898.0) | -48.8(-60.2 to -31.7) |  | -39.3(-50.0 to -26.9) | -47.0(-90.1 to 64.7) | | -65.7(-93.6 to 6.6) | -40.1(-50.2 to -27.9) |
| **Cameroon** | 4391277(2920645 to 6205337) | 3358(406 to 12163) | 245085(32364 to 893540) | 4639721(3144773 to 6480761) |  | -22.0(-44.0 to 2.2) | - | 473.6(-38.0 to 6989.2) | -17.9(-38.1 to 5.8) |  | -37.0(-50.3 to -20.7) | -12.0(-78.0 to 268.6) | | -41.8(-85.4 to 145.2) | -37.3(-49.0 to -23.3) |
| **Canada** | 20242(17459 to 23023) | 144(39 to 360) | 545(142 to 1381) | 20932(18269 to 23765) |  | -66.3(-69.3 to -62.7) | - | -56.8(-83.7 to 43.1) | -65.9(-68.6 to -62.5) |  | -3.5(-15.1 to 9.8) | 43.9(-48.5 to 243.5) | | -7.6(-67.0 to 119.8) | -3.4(-14.4 to 9.6) |
| **Central African Republic** | 27131177(19686980 to 35825781) | 12658(1510 to 45447) | 941111(116965 to 3392508) | 28084945(20714705 to 36556692) |  | -6.5(-26.9 to 18.8) | - | 354.2(-44.2 to 4220.0) | -4.2(-24.3 to 20.7) |  | -10.2(-25.5 to 7.8) | 53.0(-66.4 to 570.2) | | 2.6(-77.7 to 345.8) | -9.8(-25.0 to 7.7) |
| **Chad** | 8478256(6494826 to 10681683) | 7402(837 to 25641) | 546049(67477 to 1806506) | 9031708(7205615 to 11265134) |  | -12.5(-34.7 to 9.4) | - | 580.6(-35.2 to 9983.4) | -7.1(-28.5 to 15.2) |  | -23.1(-36.8 to -4.8) | 5.6(-77.2 to 324.4) | | -29.9(-84.8 to 182.3) | -23.5(-35.7 to -9.4) |
| **Chile** | 188508(167584 to 209146) | 1170(313 to 3159) | 4457(1283 to 11750) | 194135(173986 to 215142) |  | -70.0(-72.3 to -67.6) | - | 12.2(-78.8 to 842.2) | -69.4(-71.4 to -67.2) |  | -33.9(-40.8 to -26.1) | 4.1(-75.7 to 321.3) | | -32.9(-84.5 to 169.6) | -33.7(-40.3 to -26.8) |
| **China** | 179104(138744 to 215554) | 2922(481 to 8757) | 15283(2591 to 45539) | 197309(168265 to 229367) |  | -82.5(-85.6 to -79.0) | - | -67.1(-88.7 to 41.1) | -81.4(-83.9 to -78.4) |  | -46.0(-57.3 to -33.9) | -40.3(-85.2 to 53.2) | | -61.2(-90.4 to -0.9) | -47.5(-55.8 to -37.7) |
| **Colombia** | 157191(110848 to 208961) | 2421(343 to 7456) | 14680(2342 to 44908) | 174291(132849 to 223239) |  | -69.8(-73.4 to -66.9) | - | 406.1(5.9 to 4134.7) | -67.2(-69.1 to -65.1) |  | -31.4(-49.8 to -9.9) | 19.4(-70.7 to 184.9) | | -22.1(-80.9 to 85.1) | -30.3(-46.0 to -9.6) |
| **Comoros** | 5511669(3542776 to 7528333) | 13070(1502 to 40546) | 953996(115625 to 2906749) | 6478735(4851590 to 8327758) |  | -54.2(-67.7 to -18.8) | - | 1814.0(125.2 to 24005.9) | -50.4(-63.2 to -12.2) |  | -19.0(-44.3 to 5.9) | 152.2(-39.5 to 1004.6) | | 67.0(-59.7 to 634.8) | -12.2(-28.6 to 7.3) |
| **Congo** | 6093955(4336387 to 7966816) | 4172(382 to 16693) | 303031(31216 to 1218070) | 6401158(4685054 to 8268708) |  | -49.7(-61.6 to -35.6) | - | 178.2(-73.1 to 3607.4) | -48.1(-59.9 to -33.6) |  | -29.2(-43.5 to -12.0) | 38.2(-69.7 to 537.5) | | -8.6(-79.8 to 324.1) | -28.5(-41.0 to -13.2) |
| **Cook Islands** | 133770(110540 to 162761) | 166(9 to 832) | 862(56 to 4236) | 134798(111138 to 163801) |  | -56.4(-63.8 to -47.2) | - | 34.9(-89.7 to 1506.1) | -56.3(-63.6 to -47.2) |  | -29.5(-41.1 to -15.8) | 30.1(-77.9 to 630.6) | | -15.4(-85.7 to 372.7) | -29.3(-40.9 to -15.6) |
| **Costa Rica** | 86542(63300 to 115420) | 652(76 to 2337) | 3907(455 to 14326) | 91101(68265 to 120006) |  | -76.3(-79.3 to -73.2) | - | 476.0(-6.7 to 6595.6) | -75.2(-77.9 to -72.2) |  | -31.1(-48.6 to -8.2) | 9.9(-76.0 to 227.2) | | -28.4(-84.4 to 112.5) | -30.8(-47.3 to -8.0) |
| **Croatia** | 62148(48725 to 78553) | 212(38 to 661) | 465(83 to 1486) | 62825(49400 to 79956) |  | -83.6(-85.3 to -81.8) | - | -67.0(-92.8 to 141.8) | -83.5(-85.2 to -81.6) |  | -41.5(-54.2 to -25.7) | -47.6(-87.7 to 71.3) | | -67.2(-92.3 to 6.4) | -41.9(-54.6 to -25.6) |
| **Cuba** | 21503(16643 to 26688) | 181(30 to 551) | 1090(174 to 3314) | 22774(18219 to 28125) |  | -66.1(-70.0 to -61.8) | - | -71.0(-90.5 to 12.3) | -66.2(-69.5 to -62.5) |  | -21.3(-39.2 to -0.9) | 95.3(-60.2 to 531.1) | | 27.4(-74.2 to 310.0) | -19.4(-35.7 to -0.2) |
| **Cyprus** | 26538(22130 to 30990) | 277(47 to 922) | 1050(169 to 3355) | 27865(24070 to 32273) |  | -80.6(-85.1 to -75.5) | - | 123.2(-48.1 to 2312.6) | -77.5(-81.0 to -72.0) |  | -4.2(-20.8 to 17.9) | -60.5(-91.2 to 12.8) | | -74.8(-94.3 to -27.8) | -14.4(-26.0 to -2.0) |
| **Czechia** | 25881(20492 to 31573) | 476(130 to 1252) | 1040(286 to 2733) | 27398(22078 to 33101) |  | -79.3(-81.5 to -77.3) | - | -48.3(-85.1 to 214.0) | -78.5(-80.4 to -76.5) |  | -18.0(-34.8 to 0.3) | 17.6(-61.4 to 199.5) | | -26.8(-76.0 to 86.1) | -17.9(-34.6 to -0.4) |
| **C么te d'Ivoire** | 5088718(3427475 to 6878885) | 5428(667 to 18897) | 399054(52727 to 1363655) | 5493201(3938798 to 7196790) |  | -26.9(-43.8 to -7.6) | - | 94.9(-57.6 to 1074.6) | -23.5(-40.5 to -4.1) |  | -27.2(-42.4 to -9.2) | 13.2(-70.4 to 235.3) | | -24.8(-80.3 to 123.0) | -27.0(-41.0 to -10.5) |
| **Democratic People's Republic of Korea** | 935340(673845 to 1234486) | 16726(4161 to 41652) | 87384(22071 to 216547) | 1039450(769644 to 1346452) |  | -24.7(-48.0 to 0.1) | - | 4490.3(805.5 to 50547.5) | -17.4(-42.0 to 8.6) |  | -24.0(-39.9 to -3.3) | 25.5(-68.0 to 391.4) | | -18.7(-79.3 to 218.0) | -23.1(-35.4 to -9.0) |
| **Democratic Republic of the Congo** | 9740449(7081073 to 13936413) | 7798(665 to 28699) | 572091(57145 to 2122275) | 10320337(7614452 to 14685384) |  | -12.9(-31.6 to 11.6) | - | 453.6(-45.1 to 5681.9) | -9.8(-27.7 to 14.0) |  | -34.7(-49.0 to -18.9) | 38.2(-71.5 to 427.0) | | -8.3(-81.0 to 250.6) | -33.6(-46.8 to -19.2) |
| **Denmark** | 22433(19106 to 25489) | 207(44 to 553) | 794(169 to 2092) | 23434(20552 to 26454) |  | -54.5(-59.2 to -49.3) | - | 58.0(-63.8 to 1053.2) | -53.4(-57.9 to -48.2) |  | -34.9(-43.4 to -26.3) | 31.8(-61.7 to 305.1) | | -14.9(-75.5 to 159.2) | -34.1(-41.5 to -25.7) |
| **Djibouti** | 5381535(3782230 to 7543714) | 12328(3625 to 28370) | 905249(290576 to 2005664) | 6299112(4744602 to 8349629) |  | -34.1(-52.6 to -9.2) | - | 6938.6(1513.5 to 68870.9) | -27.2(-46.2 to -1.2) |  | -37.1(-53.4 to -16.7) | 50.9(-55.3 to 426.2) | | 0.6(-70.3 to 250.1) | -33.5(-45.7 to -16.7) |
| **Dominica** | 343340(274128 to 427864) | 761(59 to 3646) | 4621(393 to 21409) | 348721(277442 to 434046) |  | -56.3(-63.4 to -46.0) | - | -84.3(-98.6 to 153.7) | -56.9(-63.8 to -48.1) |  | -6.5(-23.2 to 13.6) | 113.7(-62.5 to 1207.6) | | 40.3(-75.7 to 749.3) | -5.9(-22.8 to 14.3) |
| **Dominican Republic** | 728949(543002 to 992530) | 1405(109 to 7043) | 8359(702 to 39740) | 738713(555208 to 999634) |  | -62.1(-70.0 to -51.5) | - | -70.2(-97.5 to 260.9) | -62.1(-70.1 to -51.6) |  | -3.5(-31.2 to 34.7) | 137.2(-60.2 to 1474.8) | | 54.0(-74.2 to 922.9) | -3.0(-30.9 to 35.0) |
| **Ecuador** | 417307(286800 to 553134) | 7845(1217 to 24052) | 47703(7298 to 150330) | 472856(377136 to 609210) |  | -77.9(-82.4 to -74.5) | - | 386.2(-23.5 to 4509.8) | -75.0(-77.4 to -72.5) |  | -38.4(-54.8 to -19.0) | -12.3(-76.5 to 151.9) | | -42.4(-84.7 to 63.6) | -38.5(-51.9 to -21.3) |
| **Egypt** | 110687(75692 to 170762) | 877(170 to 2461) | 11823(2340 to 34086) | 123386(89769 to 190102) |  | -65.1(-71.7 to -51.9) | - | 1055.5(198.9 to 10106.9) | -60.5(-66.8 to -47.0) |  | -30.4(-50.0 to -6.7) | -12.2(-81.0 to 141.2) | | -41.9(-87.5 to 58.9) | -31.6(-48.2 to -11.6) |
| **El Salvador** | 140035(105162 to 182064) | 961(102 to 3601) | 5798(665 to 21773) | 146793(112195 to 190909) |  | -85.7(-87.8 to -83.9) | - | 64.8(-83.5 to 1613.7) | -85.1(-86.5 to -83.6) |  | -16.7(-38.1 to 10.0) | 16.3(-70.7 to 487.3) | | -24.4(-81.0 to 281.5) | -16.9(-36.1 to 6.7) |
| **Equatorial Guinea** | 3047647(1883798 to 4902318) | 2505(211 to 8975) | 183579(18036 to 658239) | 3233730(2075952 to 5187289) |  | -83.4(-89.6 to -71.9) | - | 5.8(-88.5 to 1207.5) | -82.8(-89.2 to -71.5) |  | -32.7(-49.5 to -11.7) | 41.5(-69.0 to 550.8) | | -5.3(-79.4 to 332.9) | -31.5(-47.6 to -11.9) |
| **Eritrea** | 12864559(8791234 to 18044637) | 16009(1951 to 55928) | 1176044(146755 to 4015988) | 14056611(9845335 to 19365602) |  | -35.6(-54.2 to -11.8) | - | 2021.7(163.5 to 30643.0) | -31.2(-48.4 to -7.3) |  | -27.9(-43.4 to -11.2) | 44.1(-63.0 to 492.2) | | -4.4(-75.4 to 293.9) | -26.4(-38.7 to -11.2) |
| **Estonia** | 74212(43808 to 110029) | 14047(6112 to 24377) | 30626(13551 to 52806) | 118884(89670 to 153808) |  | -64.5(-74.1 to -54.3) | - | 22.3(-35.0 to 209.4) | -46.2(-51.4 to -40.4) |  | -37.2(-58.1 to -8.7) | -31.4(-63.2 to 7.4) | | -57.3(-77.1 to -33.2) | -43.5(-57.3 to -26.8) |
| **Eswatini** | 6650977(4089364 to 9539322) | 22069(4045 to 55126) | 1611459(346023 to 3662954) | 8284506(5866842 to 11058157) |  | 3.8(-32.8 to 55.2) | - | 2999.8(835.7 to 16256.3) | 29.0(-12.1 to 88.8) |  | -42.6(-61.6 to -23.2) | -16.1(-76.4 to 80.8) | | -44.4(-84.3 to 20.3) | -42.9(-55.1 to -28.1) |
| **Ethiopia** | 5663620(4404862 to 6821648) | 5672(940 to 17637) | 420244(71308 to 1321198) | 6089537(5044285 to 7149581) |  | -67.3(-74.4 to -59.9) | - | 1065.3(60.1 to 14677.3) | -65.6(-72.7 to -58.2) |  | -45.4(-54.7 to -35.4) | 17.6(-67.2 to 256.5) | | -21.3(-78.2 to 137.1) | -44.1(-51.9 to -35.0) |
| **Fiji** | 540211(423063 to 671925) | 433(31 to 1974) | 2257(166 to 9758) | 542901(426146 to 673946) |  | -42.0(-54.1 to -26.4) | - | 94.4(-86.9 to 2598.5) | -41.8(-54.0 to -26.1) |  | -21.1(-37.7 to -0.6) | 90.3(-68.6 to 1036.0) | | 23.5(-79.7 to 635.0) | -21.0(-37.5 to -0.8) |
| **Finland** | 36487(29200 to 43079) | 685(170 to 1822) | 2610(675 to 6916) | 39782(34019 to 45994) |  | -73.8(-76.8 to -70.6) | - | 100.9(-54.2 to 1522.7) | -72.5(-75.3 to -69.4) |  | -36.3(-46.8 to -26.1) | 51.3(-51.9 to 332.2) | | -2.6(-69.2 to 176.5) | -34.2(-42.3 to -25.0) |
| **France** | 57136(47155 to 67302) | 608(162 to 1728) | 2292(644 to 6285) | 60035(49927 to 69767) |  | -68.8(-71.9 to -65.4) | - | -3.6(-67.6 to 324.4) | -67.8(-70.9 to -64.7) |  | -31.5(-41.3 to -20.7) | 16.4(-60.4 to 169.2) | | -25.8(-74.6 to 72.2) | -31.0(-40.3 to -20.9) |
| **Gabon** | 3871293(2617797 to 5062403) | 3668(376 to 14637) | 267891(29556 to 948810) | 4142853(2929981 to 5314713) |  | -44.1(-60.0 to -26.3) | - | 314.9(-58.9 to 5419.3) | -41.5(-56.7 to -24.0) |  | -37.8(-50.0 to -22.3) | 18.7(-73.3 to 404.4) | | -21.2(-82.2 to 235.5) | -36.9(-47.6 to -23.4) |
| **Gambia** | 6515553(4839515 to 8392086) | 4931(528 to 17817) | 360463(40231 to 1268635) | 6880947(5237785 to 8701925) |  | -10.1(-33.3 to 18.4) | - | 594.6(-20.3 to 8836.9) | -5.1(-26.2 to 21.4) |  | -17.5(-34.5 to 4.8) | 7.0(-77.6 to 328.6) | | -28.7(-85.1 to 185.1) | -18.2(-33.3 to 0.2) |
| **Georgia** | 245254(149860 to 332420) | 43699(20828 to 76432) | 95517(47654 to 161562) | 384470(316001 to 459289) |  | -55.0(-66.1 to -44.6) | - | 3518.1(906.0 to 31722.4) | -36.5(-44.5 to -27.5) |  | -32.4(-51.3 to -9.8) | 30.1(-19.4 to 101.6) | | -18.9(-49.9 to 25.3) | -25.2(-39.5 to -8.6) |
| **Germany** | 20857(17602 to 23536) | 455(137 to 1106) | 1724(566 to 3868) | 23036(20780 to 25212) |  | -83.4(-84.8 to -82.0) | - | -47.3(-83.5 to 147.5) | -82.7(-83.8 to -81.4) |  | -18.0(-29.3 to -7.6) | 105.6(-17.6 to 337.9) | | 31.6(-47.3 to 180.2) | -14.6(-21.9 to -6.5) |
| **Ghana** | 5485257(4224444 to 6935336) | 4716(548 to 15387) | 343822(43196 to 1141590) | 5833794(4641037 to 7266829) |  | -39.7(-53.8 to -20.7) | - | 324.4(-48.5 to 5622.4) | -36.5(-50.5 to -17.2) |  | -33.2(-45.2 to -19.1) | 2.2(-76.4 to 309.5) | | -32.8(-84.3 to 172.4) | -33.2(-43.3 to -21.2) |
| **Greece** | 47567(38306 to 54958) | 760(111 to 2392) | 2895(460 to 9392) | 51222(44565 to 57217) |  | -58.6(-64.6 to -52.8) | - | 396.7(7.3 to 4723.9) | -53.5(-57.9 to -49.2) |  | -30.2(-40.6 to -19.7) | -41.7(-87.0 to 55.3) | | -62.5(-91.7 to -0.7) | -33.6(-40.7 to -26.5) |
| **Greenland** | 471521(368358 to 566586) | 5146(587 to 17961) | 19480(2484 to 73303) | 496147(402450 to 586626) |  | -65.2(-71.5 to -58.2) | - | -63.7(-95.8 to 227.7) | -65.0(-70.6 to -59.0) |  | -29.7(-42.9 to -15.8) | 30.0(-71.7 to 546.3) | | -17.7(-81.9 to 313.5) | -28.9(-40.8 to -16.5) |
| **Grenada** | 51909(44806 to 59768) | 139(10 to 679) | 849(65 to 4389) | 52898(45857 to 60485) |  | -65.0(-69.5 to -59.3) | - | -69.4(-97.0 to 179.4) | -65.0(-69.5 to -59.8) |  | -27.7(-38.0 to -16.8) | 127.1(-68.2 to 1473.3) | | 50.6(-79.4 to 922.1) | -27.0(-37.3 to -16.2) |
| **Guam** | 200188(161971 to 241441) | 408(37 to 1698) | 2159(219 to 8226) | 202755(165041 to 245902) |  | -59.4(-66.8 to -50.8) | - | 467.9(0.0 to 6495.2) | -57.7(-64.7 to -49.0) |  | -10.8(-27.8 to 7.9) | -64.6(-95.2 to 37.4) | | -76.8(-96.9 to -11.1) | -13.6(-29.4 to 4.1) |
| **Guatemala** | 295970(221861 to 383202) | 2712(352 to 9366) | 16424(1962 to 52730) | 315106(243417 to 402574) |  | -89.1(-91.0 to -86.9) | - | 79.1(-73.7 to 1824.3) | -88.4(-90.0 to -86.3) |  | -29.3(-48.0 to -4.9) | -4.3(-75.4 to 206.5) | | -37.3(-84.0 to 99.1) | -29.6(-48.0 to -7.2) |
| **Guinea** | 6381502(4593281 to 8494756) | 4584(559 to 15553) | 337826(38983 to 1098346) | 6723912(4955721 to 8906369) |  | -30.5(-46.7 to -11.3) | - | 370.0(-46.4 to 5682.7) | -26.8(-42.1 to -7.1) |  | -15.8(-31.7 to 5.3) | 5.7(-72.4 to 331.0) | | -29.1(-81.7 to 186.7) | -16.6(-31.7 to 2.2) |
| **Guinea-Bissau** | 7786939(5776136 to 10173356) | 4563(612 to 16047) | 336809(44389 to 1159333) | 8128311(6203951 to 10586237) |  | -43.8(-56.6 to -26.3) | - | 297.4(-49.1 to 4896.1) | -41.0(-53.8 to -24.3) |  | -25.9(-40.4 to -9.0) | -17.5(-81.1 to 248.7) | | -44.5(-87.4 to 132.0) | -26.9(-39.5 to -12.3) |
| **Guyana** | 870629(652285 to 1125360) | 2191(163 to 11555) | 13203(1029 to 72205) | 886023(676303 to 1139611) |  | -37.0(-47.8 to -23.6) | - | -1.3(-91.0 to 1092.8) | -36.7(-47.4 to -23.3) |  | -33.9(-51.0 to -14.0) | 68.0(-71.1 to 1006.8) | | 9.8(-81.2 to 619.0) | -33.4(-50.3 to -13.6) |
| **Haiti** | 1435822(1032617 to 1962204) | 2163(184 to 9770) | 13019(1142 to 56424) | 1451005(1050600 to 1966993) |  | -55.4(-64.4 to -43.7) | - | -58.1(-96.3 to 321.3) | -55.3(-64.1 to -43.7) |  | -25.0(-36.7 to -11.1) | 75.0(-72.5 to 1263.7) | | 14.0(-82.2 to 785.8) | -24.7(-36.3 to -10.8) |
| **Honduras** | 860374(652416 to 1186536) | 8143(989 to 28601) | 48920(6426 to 162936) | 917438(741754 to 1251853) |  | -49.3(-61.3 to -31.7) | - | 1232.5(101.2 to 15487.9) | -46.3(-58.0 to -28.5) |  | -12.2(-29.4 to 11.8) | 38.5(-65.6 to 313.8) | | -9.7(-77.6 to 168.8) | -11.8(-27.5 to 10.4) |
| **Hungary** | 30155(23739 to 37242) | 365(57 to 1265) | 806(120 to 2842) | 31326(25517 to 38431) |  | -87.7(-89.1 to -86.5) | - | 42.8(-67.6 to 1354.8) | -86.7(-87.9 to -85.5) |  | -40.7(-52.6 to -26.1) | -64.0(-92.6 to 18.6) | | -77.4(-95.4 to -26.3) | -43.5(-54.2 to -30.8) |
| **Iceland** | 20328(16998 to 23835) | 107(11 to 382) | 405(42 to 1414) | 20839(17623 to 24257) |  | -74.4(-79.1 to -70.0) | - | 169.5(-54.3 to 2340.5) | -71.7(-75.0 to -67.9) |  | -36.4(-46.0 to -22.9) | -80.6(-96.2 to -34.3) | | -87.6(-97.6 to -57.9) | -41.7(-49.1 to -32.9) |
| **India** | 3117774(2300991 to 3857611) | 24179(4004 to 61675) | 468876(75833 to 1198253) | 3610829(3072574 to 4260159) |  | -62.9(-69.3 to -56.0) | - | 2374.8(338.6 to 37236.8) | -58.1(-62.8 to -52.1) |  | -30.8(-45.7 to -14.8) | 24.7(-64.8 to 201.5) | | -17.7(-76.7 to 99.4) | -29.2(-40.0 to -17.3) |
| **Indonesia** | 3776695(3223152 to 4264266) | 6286(547 to 26900) | 32772(3172 to 142602) | 3815753(3274090 to 4301897) |  | -47.0(-54.4 to -37.9) | - | 144.0(-80.7 to 3895.5) | -46.1(-52.9 to -37.2) |  | -36.7(-46.6 to -25.7) | -54.5(-91.6 to 171.9) | | -70.6(-94.6 to 75.9) | -37.3(-47.2 to -27.4) |
| **Iran (Islamic Republic of)** | 112382(100220 to 127558) | 340(78 to 910) | 4571(1070 to 12759) | 117293(107126 to 132384) |  | -67.2(-72.7 to -58.2) | - | 501.1(20.4 to 8561.6) | -65.9(-71.5 to -57.0) |  | -28.7(-36.0 to -17.5) | 19.0(-72.4 to 378.5) | | -21.8(-81.8 to 215.3) | -28.4(-33.1 to -17.4) |
| **Iraq** | 360741(282102 to 458189) | 1827(428 to 4923) | 24639(5703 to 64656) | 387207(307105 to 484292) |  | -60.4(-69.7 to -45.9) | - | 957.2(144.6 to 14528.9) | -58.1(-67.1 to -42.9) |  | -40.9(-52.5 to -25.1) | 6.4(-71.9 to 276.1) | | -29.7(-81.5 to 147.8) | -40.1(-50.7 to -25.3) |
| **Ireland** | 37975(31139 to 45540) | 293(56 to 921) | 1111(227 to 3408) | 39378(32865 to 47094) |  | -68.9(-72.8 to -64.4) | - | -2.6(-74.9 to 845.5) | -67.8(-71.8 to -63.4) |  | -30.1(-39.5 to -20.6) | -18.7(-81.3 to 151.0) | | -48.0(-88.0 to 60.6) | -30.7(-38.8 to -21.2) |
| **Israel** | 18631(14044 to 22107) | 617(160 to 1567) | 2338(629 to 5953) | 21585(18901 to 24187) |  | -72.1(-76.4 to -66.9) | - | -38.3(-83.5 to 284.0) | -69.9(-73.1 to -66.2) |  | -35.1(-49.8 to -22.3) | 9.9(-65.1 to 168.5) | | -29.7(-77.7 to 71.8) | -33.8(-41.2 to -25.6) |
| **Italy** | 25286(21700 to 28257) | 375(118 to 910) | 1427(458 to 3311) | 27089(24059 to 29684) |  | -72.3(-74.9 to -69.9) | - | -5.0(-71.5 to 424.6) | -70.4(-72.4 to -68.7) |  | -16.0(-23.3 to -8.6) | -6.2(-60.5 to 94.2) | | -39.8(-74.7 to 24.3) | -17.6(-22.1 to -12.7) |
| **Jamaica** | 33367(25253 to 42381) | 178(24 to 669) | 1070(139 to 3795) | 34615(26953 to 44213) |  | -70.4(-74.2 to -66.5) | - | -40.6(-93.2 to 857.5) | -70.1(-73.8 to -66.3) |  | -12.8(-33.8 to 11.2) | 120.5(-63.3 to 1114.0) | | 42.8(-76.2 to 688.6) | -11.4(-31.5 to 12.6) |
| **Japan** | 75099(61163 to 83391) | 342(34 to 1492) | 1301(133 to 5551) | 76742(63169 to 84458) |  | -67.5(-71.2 to -65.1) | - | -61.1(-94.2 to 147.3) | -67.3(-70.9 to -65.2) |  | -29.7(-34.5 to -25.6) | 19.8(-77.9 to 379.4) | | -23.5(-85.8 to 206.7) | -29.5(-32.8 to -25.9) |
| **Jordan** | 48467(35372 to 60558) | 330(49 to 1139) | 4431(696 to 14793) | 53228(44453 to 64183) |  | -78.7(-83.3 to -71.4) | - | 643.4(59.4 to 8810.2) | -75.6(-79.8 to -68.0) |  | -34.7(-49.6 to -17.2) | -36.6(-85.6 to 79.9) | | -58.2(-90.5 to 18.5) | -37.7(-48.1 to -24.6) |
| **Kazakhstan** | 228753(134645 to 326604) | 52516(25316 to 87150) | 114951(55572 to 187334) | 396219(334821 to 458889) |  | -60.7(-74.8 to -45.3) | - | 4137.5(1089.0 to 41981.7) | -15.9(-22.0 to -9.4) |  | -54.9(-68.4 to -36.3) | -59.7(-75.8 to -41.2) | | -74.8(-85.0 to -63.4) | -63.7(-69.5 to -57.7) |
| **Kenya** | 6289409(4713249 to 7808128) | 3116(745 to 8227) | 228988(56843 to 588167) | 6521513(4936924 to 8134237) |  | -12.6(-27.9 to 1.3) | - | 2770.6(640.9 to 41361.8) | -9.4(-24.7 to 4.4) |  | -28.9(-39.0 to -16.9) | 3.6(-74.9 to 295.2) | | -31.1(-83.3 to 162.9) | -29.0(-38.6 to -17.9) |
| **Kiribati** | 8896590(6881489 to 11514083) | 10625(917 to 45674) | 55999(4705 to 236930) | 8963214(6946347 to 11522028) |  | -28.1(-43.0 to -8.5) | - | 140.2(-79.9 to 2800.0) | -27.7(-42.8 to -8.0) |  | -18.8(-31.0 to -4.6) | 32.8(-76.9 to 746.3) | | -14.0(-85.0 to 447.5) | -18.7(-30.8 to -4.8) |
| **Kuwait** | 113838(90882 to 140405) | 323(62 to 960) | 4340(899 to 12368) | 118501(95001 to 146054) |  | -60.0(-65.9 to -52.9) | - | 329.9(-0.8 to 6487.6) | -58.7(-64.7 to -51.4) |  | -48.2(-57.1 to -37.0) | -13.9(-77.9 to 172.0) | | -43.3(-85.4 to 79.3) | -48.0(-56.9 to -36.7) |
| **Kyrgyzstan** | 369032(177855 to 591350) | 101691(37100 to 174523) | 221997(84185 to 375562) | 692720(602049 to 797423) |  | -37.6(-59.5 to -14.0) | - | 25684.4(5799.5 to 291627.5) | 22.5(13.0 to 33.2) |  | -44.1(-72.0 to -6.4) | -28.2(-70.1 to 31.3) | | -55.2(-81.4 to -18.4) | -46.6(-53.8 to -38.2) |
| **Lao People's Democratic Republic** | 4444849(3278797 to 5614836) | 8908(791 to 38443) | 46528(4628 to 186827) | 4500285(3317246 to 5705620) |  | -58.9(-67.9 to -46.6) | - | 112.5(-76.8 to 2902.8) | -58.1(-67.4 to -45.5) |  | -41.3(-51.7 to -28.5) | -53.0(-91.8 to 168.9) | | -69.5(-94.7 to 74.0) | -41.9(-52.0 to -30.3) |
| **Latvia** | 128400(88873 to 172418) | 12294(4764 to 24382) | 26918(10326 to 53153) | 167612(132000 to 211475) |  | -38.0(-50.3 to -13.9) | - | -51.6(-71.1 to -4.4) | -38.0(-43.6 to -31.0) |  | -29.4(-48.3 to -4.2) | -21.6(-59.5 to 35.4) | | -51.1(-74.8 to -15.9) | -33.6(-48.3 to -15.5) |
| **Lebanon** | 76777(50163 to 145060) | 245(23 to 1066) | 3267(320 to 13986) | 80289(54604 to 148130) |  | -75.2(-82.3 to -50.4) | - | 192.6(-64.0 to 3631.3) | -74.4(-81.6 to -48.2) |  | -29.3(-44.1 to -12.2) | 32.0(-73.7 to 378.4) | | -14.0(-82.7 to 215.2) | -28.6(-41.8 to -13.1) |
| **Lesotho** | 15108961(11009054 to 19689081) | 21011(5429 to 53797) | 1541526(399003 to 3770433) | 16671499(12415465 to 21222228) |  | 48.9(7.4 to 102.7) | - | 7285.3(1594.2 to 80418.5) | 61.7(19.0 to 117.9) |  | -25.3(-42.7 to -5.4) | 32.3(-69.0 to 405.3) | | -12.0(-79.4 to 236.2) | -24.2(-38.7 to -7.6) |
| **Liberia** | 4374505(3217796 to 6190706) | 2840(278 to 11863) | 205541(21798 to 807344) | 4582886(3474343 to 6391347) |  | -53.8(-66.1 to -35.4) | - | 186.1(-71.0 to 3560.5) | -51.9(-62.8 to -33.6) |  | -23.6(-39.9 to -2.0) | 7.0(-75.4 to 362.8) | | -29.1(-83.7 to 207.9) | -23.8(-39.0 to -6.1) |
| **Libya** | 155473(104760 to 206208) | 512(44 to 2082) | 6867(607 to 27069) | 162852(109337 to 211874) |  | -66.7(-75.8 to -50.9) | - | 209.0(-67.3 to 3603.4) | -65.4(-74.7 to -49.5) |  | 4.6(-19.0 to 32.1) | 68.7(-69.6 to 815.9) | | 10.4(-80.0 to 503.5) | 5.0(-16.5 to 31.8) |
| **Lithuania** | 225178(142152 to 312625) | 40371(17622 to 71073) | 88138(40937 to 149607) | 353687(285147 to 440732) |  | -28.5(-46.3 to -11.2) | - | 172.4(4.2 to 977.4) | -1.7(-9.6 to 6.7) |  | -42.8(-58.7 to -23.8) | -11.3(-45.7 to 33.2) | | -44.7(-66.2 to -17.2) | -40.9(-53.2 to -26.0) |
| **Luxembourg** | 15719(12195 to 19052) | 226(28 to 851) | 857(110 to 3103) | 16802(14127 to 19844) |  | -73.4(-78.8 to -68.8) | - | 229.7(-36.2 to 2897.5) | -70.9(-74.1 to -67.1) |  | -27.2(-40.2 to -12.0) | -31.1(-88.0 to 157.9) | | -55.8(-92.3 to 65.0) | -29.6(-39.5 to -17.1) |
| **Madagascar** | 8638782(6250111 to 11674300) | 7430(1024 to 26569) | 548160(81103 to 1889566) | 9194373(6807039 to 12219524) |  | -35.5(-49.2 to -18.9) | - | 1689.7(212.1 to 19074.7) | -33.6(-47.6 to -16.8) |  | -21.1(-38.8 to -2.9) | 145.7(-44.8 to 623.2) | | 64.1(-63.3 to 381.1) | -18.5(-34.0 to -1.8) |
| **Malawi** | 8753347(6768509 to 11115367) | 5541(818 to 17329) | 407596(60875 to 1242472) | 9166484(7309690 to 11535432) |  | -37.7(-49.1 to -24.5) | - | 1457.0(218.0 to 17611.5) | -36.3(-48.2 to -23.0) |  | -26.2(-39.4 to -11.4) | 125.1(-57.0 to 644.1) | | 50.0(-71.4 to 395.0) | -24.5(-37.2 to -10.5) |
| **Malaysia** | 785734(628867 to 971881) | 2782(490 to 9222) | 14500(2822 to 45709) | 803017(643656 to 993317) |  | -47.9(-55.0 to -39.9) | - | 879.3(67.8 to 9778.5) | -46.6(-53.0 to -38.7) |  | -28.2(-42.8 to -9.5) | -16.7(-84.5 to 348.4) | | -45.8(-90.0 to 190.1) | -28.6(-42.7 to -11.0) |
| **Maldives** | 506178(413954 to 615243) | 922(80 to 4092) | 4878(412 to 22355) | 511979(419518 to 622412) |  | -87.4(-90.1 to -83.5) | - | -33.3(-94.0 to 1008.9) | -87.1(-89.7 to -83.3) |  | -33.9(-46.8 to -17.4) | -53.4(-90.4 to 143.1) | | -69.8(-93.8 to 57.3) | -34.7(-47.2 to -19.3) |
| **Mali** | 5058654(3781433 to 6916736) | 4389(463 to 14692) | 321804(36244 to 1112692) | 5384846(4189565 to 7280033) |  | -48.4(-59.7 to -33.2) | - | 278.4(-50.0 to 4881.9) | -45.5(-56.0 to -31.0) |  | -21.5(-37.2 to -0.5) | 13.1(-73.5 to 418.4) | | -24.9(-82.4 to 244.9) | -21.7(-35.2 to -6.6) |
| **Malta** | 12395(10583 to 14404) | 54(6 to 225) | 205(21 to 838) | 12654(10881 to 14620) |  | -71.8(-76.8 to -67.3) | - | 132.5(-67.0 to 2842.1) | -69.9(-73.6 to -66.0) |  | -20.7(-32.7 to -5.8) | -66.8(-93.8 to 8.1) | | -78.7(-96.0 to -30.9) | -24.5(-34.7 to -13.6) |
| **Marshall Islands** | 1953474(1318689 to 2602980) | 8374(1027 to 30055) | 43660(5851 to 150887) | 2005508(1359223 to 2655757) |  | -36.6(-51.3 to -19.9) | - | 1996.4(313.7 to 28001.9) | -33.1(-47.9 to -15.9) |  | -27.5(-39.9 to -12.4) | -51.5(-92.7 to 112.0) | | -68.6(-95.3 to 37.2) | -29.6(-41.5 to -15.5) |
| **Mauritania** | 2165321(1393849 to 3168303) | 1670(151 to 6025) | 121745(11360 to 416472) | 2288736(1514614 to 3346176) |  | -59.8(-69.1 to -44.5) | - | 249.5(-62.1 to 3924.6) | -57.5(-66.4 to -42.5) |  | -34.8(-49.4 to -17.7) | -13.7(-79.4 to 219.0) | | -43.0(-86.3 to 112.2) | -35.3(-48.7 to -20.4) |
| **Mauritius** | 86681(68869 to 109582) | 182(33 to 596) | 950(179 to 3279) | 87813(69519 to 110594) |  | -65.6(-69.5 to -61.4) | - | 424.0(-9.6 to 5336.3) | -64.7(-68.5 to -60.4) |  | -17.2(-34.1 to 4.3) | -47.8(-87.3 to 99.3) | | -66.2(-91.8 to 29.0) | -18.6(-34.7 to 2.5) |
| **Mexico** | 199813(161897 to 239677) | 2129(356 to 6302) | 12935(2212 to 39615) | 214877(182219 to 253018) |  | -81.6(-82.9 to -80.6) | - | 709.8(79.4 to 7348.5) | -80.5(-81.1 to -79.7) |  | -25.3(-38.6 to -10.6) | 23.9(-69.9 to 258.6) | | -18.7(-80.5 to 133.0) | -24.6(-35.5 to -11.8) |
| **Micronesia (Federated States of)** | 1545196(1063197 to 2022432) | 2068(101 to 10973) | 10821(535 to 53492) | 1558085(1087229 to 2053982) |  | -55.7(-66.2 to -42.8) | - | 47.4(-87.8 to 1852.6) | -55.4(-66.1 to -42.5) |  | -24.1(-39.5 to -6.7) | 37.5(-77.0 to 642.0) | | -11.5(-85.1 to 380.1) | -23.9(-39.1 to -6.7) |
| **Monaco** | 83245(65305 to 100661) | 588(55 to 2280) | 2256(223 to 8019) | 86089(69004 to 103155) |  | -62.8(-71.2 to -51.8) | - | 6.2(-88.8 to 1093.1) | -61.9(-69.9 to -50.9) |  | -15.5(-31.9 to 3.0) | 26.9(-74.7 to 537.4) | | -18.3(-83.8 to 307.8) | -15.4(-30.7 to 1.3) |
| **Mongolia** | 883602(486539 to 1258576) | 79696(17247 to 201952) | 174943(35955 to 462629) | 1138242(885503 to 1477937) |  | -47.8(-62.8 to -31.7) | - | 1443.5(196.6 to 16068.2) | -38.6(-50.9 to -22.6) |  | -41.5(-63.1 to -18.5) | 30.9(-59.3 to 237.1) | | -18.4(-74.7 to 109.4) | -36.3(-49.9 to -19.3) |
| **Montenegro** | 46674(37883 to 56918) | 403(51 to 1450) | 875(112 to 3082) | 47951(39193 to 58518) |  | -36.3(-45.6 to -23.5) | - | 277.5(-26.2 to 3494.0) | -34.2(-43.7 to -21.2) |  | -26.5(-39.5 to -10.5) | -22.9(-87.1 to 176.8) | | -52.2(-92.0 to 72.0) | -27.2(-40.0 to -11.6) |
| **Morocco** | 1295919(953242 to 2423754) | 4623(919 to 13620) | 62217(12671 to 177354) | 1362758(1011521 to 2556087) |  | -57.2(-66.9 to -37.3) | - | 686.3(50.0 to 9278.3) | -55.5(-65.4 to -35.0) |  | -35.7(-49.0 to -11.8) | 19.4(-72.5 to 402.4) | | -21.4(-81.9 to 231.0) | -35.0(-47.4 to -10.9) |
| **Mozambique** | 13443338(9804295 to 17988845) | 22538(5689 to 56922) | 1654015(437859 to 3927490) | 15119891(11636803 to 19561138) |  | -29.8(-43.6 to -10.6) | - | 991.5(196.7 to 8159.2) | -23.2(-37.8 to -3.3) |  | -24.0(-41.5 to -1.8) | 40.3(-57.0 to 258.0) | | -6.8(-71.4 to 138.2) | -22.4(-38.2 to -3.9) |
| **Myanmar** | 2689409(1894384 to 3530600) | 68340(18443 to 162030) | 356042(103699 to 846949) | 3113791(2438266 to 3892389) |  | -61.7(-71.2 to -50.8) | - | 1121.7(159.3 to 12086.1) | -57.0(-66.9 to -45.1) |  | -47.8(-60.1 to -34.0) | -5.9(-66.9 to 101.1) | | -39.2(-78.6 to 30.1) | -46.4(-54.7 to -34.9) |
| **Namibia** | 4386904(3147672 to 5846755) | 8327(2541 to 18140) | 611178(204760 to 1313243) | 5006410(3783823 to 6405276) |  | -27.1(-47.5 to 7.6) | - | 5076.7(952.6 to 50393.0) | -18.5(-39.0 to 17.3) |  | -41.1(-55.6 to -22.5) | 4.1(-71.7 to 282.7) | | -30.7(-81.2 to 154.6) | -39.9(-51.6 to -26.1) |
| **Nauru** | 1069497(857186 to 1354437) | 1327(104 to 6306) | 6983(585 to 34653) | 1077807(863271 to 1363268) |  | -8.1(-27.9 to 15.7) | - | 170.1(-81.0 to 3672.6) | -7.7(-27.1 to 15.9) |  | -51.2(-59.3 to -41.2) | -13.5(-85.4 to 415.8) | | -43.6(-90.5 to 233.6) | -51.1(-59.3 to -41.3) |
| **Nepal** | 3052378(2261124 to 3840373) | 17551(3623 to 50274) | 339196(71745 to 915324) | 3409125(2680721 to 4151541) |  | -66.1(-74.2 to -55.4) | - | 60.4(-57.0 to 937.0) | -63.6(-72.2 to -52.3) |  | -30.1(-47.1 to -11.9) | 28.3(-66.2 to 220.9) | | -15.1(-77.7 to 112.2) | -28.7(-42.4 to -13.6) |
| **Netherlands** | 25680(21555 to 29171) | 285(76 to 733) | 1089(293 to 2800) | 27055(23427 to 30515) |  | -65.6(-69.3 to -60.9) | - | 34.8(-58.3 to 617.1) | -64.1(-67.9 to -59.7) |  | -21.8(-32.5 to -10.8) | 9.4(-57.6 to 152.7) | | -29.4(-72.9 to 61.7) | -21.9(-31.6 to -11.4) |
| **New Zealand** | 21720(18465 to 24451) | 221(39 to 694) | 837(167 to 2556) | 22778(20195 to 25298) |  | -70.6(-73.7 to -67.3) | - | -31.5(-80.8 to 295.2) | -69.7(-72.6 to -66.5) |  | -17.3(-28.7 to -6.3) | 8.5(-72.6 to 220.7) | | -30.5(-82.5 to 105.2) | -17.7(-26.5 to -8.0) |
| **Nicaragua** | 292849(235062 to 352975) | 1961(265 to 6356) | 11873(1730 to 38071) | 306682(251499 to 367395) |  | -70.8(-74.1 to -66.6) | - | 62.1(-69.7 to 1215.7) | -69.8(-72.8 to -66.0) |  | -32.9(-45.3 to -18.5) | 13.5(-73.0 to 241.6) | | -25.7(-82.4 to 121.9) | -32.4(-44.5 to -19.5) |
| **Niger** | 5574131(4182420 to 7340783) | 4326(528 to 14606) | 318623(37350 to 1041637) | 5897080(4567356 to 7661680) |  | -51.4(-63.6 to -35.7) | - | 262.2(-60.4 to 5856.2) | -48.5(-58.9 to -32.7) |  | -12.1(-29.1 to 9.6) | 9.2(-73.0 to 399.0) | | -26.8(-82.1 to 232.0) | -13.0(-28.4 to 4.6) |
| **Nigeria** | 4090585(3074657 to 5511020) | 5218(1110 to 15007) | 386151(79506 to 1088070) | 4481953(3502624 to 6009472) |  | -51.7(-62.8 to -35.2) | - | 2156.7(382.9 to 24584.9) | -47.2(-59.3 to -29.6) |  | -25.6(-44.5 to -3.3) | 11.3(-70.5 to 188.9) | | -25.4(-80.4 to 92.2) | -25.6(-42.8 to -5.0) |
| **Niue** | 338478(265199 to 437885) | 390(32 to 1856) | 2016(184 to 8978) | 340884(266566 to 439844) |  | -62.1(-69.5 to -52.0) | - | 26.3(-90.9 to 1227.9) | -61.9(-69.4 to -52.0) |  | -21.4(-33.0 to -7.1) | 28.7(-76.1 to 802.8) | | -17.4(-84.6 to 484.3) | -21.3(-32.8 to -7.5) |
| **North Macedonia** | 121590(95546 to 152013) | 1525(383 to 4127) | 3329(802 to 8563) | 126444(99790 to 156602) |  | -76.9(-80.1 to -73.0) | - | 122.2(-48.4 to 2085.8) | -75.6(-78.6 to -71.8) |  | -26.3(-42.3 to -7.6) | -32.5(-80.8 to 97.3) | | -58.0(-88.0 to 22.6) | -27.8(-42.9 to -10.1) |
| **Northern Mariana Islands** | 238556(197562 to 280947) | 1060(128 to 4382) | 5543(745 to 24302) | 245159(206534 to 288530) |  | -72.8(-78.3 to -66.4) | - | -1.4(-86.5 to 1371.6) | -72.2(-77.6 to -65.3) |  | -20.2(-32.4 to -6.8) | 9.1(-81.4 to 308.3) | | -29.3(-87.9 to 164.2) | -20.4(-31.7 to -7.7) |
| **Norway** | 37727(32202 to 41803) | 503(115 to 1349) | 1908(430 to 5002) | 40137(35941 to 43609) |  | -53.5(-57.1 to -49.5) | - | -10.4(-72.4 to 320.9) | -51.8(-54.2 to -49.2) |  | -22.1(-30.8 to -14.2) | 2.6(-68.3 to 178.2) | | -34.3(-79.7 to 78.0) | -22.6(-27.3 to -17.1) |
| **Oman** | 117677(89836 to 153688) | 399(66 to 1354) | 5367(897 to 17638) | 123442(95297 to 157862) |  | -71.7(-81.9 to -57.0) | - | -41.2(-85.2 to 272.0) | -71.0(-81.5 to -56.0) |  | -40.5(-51.0 to -28.1) | 0.0(-80.2 to 209.7) | | -34.1(-87.0 to 104.1) | -40.2(-49.3 to -28.1) |
| **Pakistan** | 4083032(3100274 to 5311416) | 26737(6878 to 71840) | 519370(135740 to 1348755) | 4629138(3707725 to 6016976) |  | -35.7(-50.8 to -15.4) | - | 5085.4(1009.5 to 54920.8) | -29.2(-44.6 to -6.1) |  | -34.2(-50.0 to -16.2) | 28.2(-66.9 to 371.1) | | -14.5(-78.1 to 211.5) | -32.2(-44.4 to -17.1) |
| **Palau** | 399884(311918 to 499346) | 537(35 to 2611) | 2784(209 to 13896) | 403204(313001 to 503767) |  | -47.0(-59.8 to -28.7) | - | 81.8(-88.4 to 2007.5) | -46.8(-59.8 to -28.4) |  | -17.9(-32.9 to 0.1) | 44.7(-77.9 to 795.5) | | -6.8(-85.7 to 479.5) | -17.8(-33.0 to 0.4) |
| **Palestine** | 108425(76699 to 134542) | 395(37 to 1737) | 5245(547 to 21075) | 114065(84858 to 140313) |  | -63.9(-78.8 to -47.3) | - | 225.6(-66.3 to 4260.7) | -62.5(-77.9 to -45.5) |  | -30.9(-46.0 to -6.3) | 22.8(-74.9 to 513.3) | | -19.1(-83.5 to 304.1) | -30.3(-44.2 to -7.6) |
| **Panama** | 427441(306927 to 575198) | 3565(360 to 13688) | 21673(2157 to 86440) | 452678(335831 to 604829) |  | -47.6(-56.8 to -40.9) | - | 671.6(-7.9 to 7602.3) | -44.7(-49.6 to -39.2) |  | -30.5(-49.0 to -6.5) | -0.1(-78.3 to 341.7) | | -34.9(-85.9 to 186.9) | -30.6(-48.2 to -6.7) |
| **Papua New Guinea** | 2355633(1467189 to 3304740) | 37197(8593 to 96643) | 194550(45165 to 486156) | 2587380(1618984 to 3585343) |  | -36.6(-47.9 to -23.3) | - | 1851.5(304.4 to 18839.0) | -34.7(-45.8 to -20.8) |  | -28.1(-41.4 to -13.5) | 228.2(-17.6 to 1174.4) | | 112.2(-46.7 to 724.5) | -23.4(-34.7 to -9.9) |
| **Paraguay** | 377796(265464 to 507781) | 4446(465 to 15366) | 26854(2971 to 91843) | 409096(310607 to 530910) |  | -41.6(-50.1 to -31.2) | - | 582.0(22.6 to 6761.2) | -38.5(-46.6 to -28.0) |  | -27.3(-47.3 to -2.3) | 53.2(-74.6 to 367.4) | | -0.4(-83.5 to 203.6) | -25.5(-44.2 to -2.5) |
| **Peru** | 487435(337310 to 682996) | 14087(4690 to 30110) | 84874(29052 to 184180) | 586396(432153 to 784164) |  | -83.9(-87.6 to -79.7) | - | 63.2(-46.2 to 613.1) | -80.5(-84.3 to -76.1) |  | -40.9(-60.1 to -16.1) | -25.6(-63.0 to 48.5) | | -51.6(-76.0 to -3.6) | -42.5(-60.3 to -21.0) |
| **Philippines** | 3339506(2648630 to 4015710) | 37600(8698 to 98454) | 196701(43593 to 508921) | 3573807(2926108 to 4270886) |  | -47.0(-54.3 to -36.2) | - | 2249.9(483.3 to 23055.6) | -43.3(-50.1 to -32.5) |  | -23.0(-38.9 to -6.4) | 11.0(-71.3 to 214.2) | | -28.1(-81.4 to 103.3) | -23.1(-37.8 to -7.5) |
| **Poland** | 87124(71989 to 105702) | 623(183 to 1623) | 1363(396 to 3528) | 89111(73109 to 107738) |  | -72.7(-73.9 to -71.6) | - | -47.9(-82.9 to 140.4) | -72.4(-73.4 to -71.3) |  | -32.6(-44.5 to -17.2) | -9.3(-67.5 to 132.4) | | -43.4(-79.8 to 44.4) | -32.7(-44.6 to -18.3) |
| **Portugal** | 92114(80231 to 102824) | 758(141 to 2357) | 2868(546 to 9095) | 95740(86396 to 105495) |  | -65.3(-68.0 to -62.4) | - | -52.9(-83.0 to 59.9) | -64.8(-67.2 to -62.3) |  | -31.3(-39.4 to -23.1) | -4.4(-76.0 to 158.7) | | -38.8(-84.7 to 65.5) | -31.4(-38.2 to -24.1) |
| **Puerto Rico** | 29644(22663 to 38803) | 145(21 to 486) | 876(135 to 3001) | 30665(23832 to 40081) |  | -85.1(-86.7 to -83.5) | - | 58.4(-64.1 to 1187.5) | -84.4(-85.8 to -82.9) |  | -16.7(-36.2 to 9.7) | -23.2(-82.6 to 166.8) | | -50.1(-88.7 to 73.3) | -18.3(-36.5 to 5.6) |
| **Qatar** | 132789(100668 to 171891) | 467(75 to 1601) | 6288(1022 to 21459) | 139544(108072 to 178855) |  | -66.5(-74.6 to -53.6) | - | 143.8(-49.4 to 2764.4) | -65.4(-73.7 to -52.4) |  | -32.0(-47.3 to -13.9) | 34.7(-69.0 to 382.8) | | -11.0(-79.6 to 218.1) | -31.2(-46.5 to -13.7) |
| **Republic of Korea** | 369756(307055 to 424616) | 2812(325 to 11322) | 10649(1169 to 41671) | 383217(329911 to 435759) |  | -78.2(-80.6 to -75.9) | - | -38.8(-88.0 to 331.7) | -77.6(-79.9 to -75.4) |  | -35.8(-43.0 to -28.2) | -18.3(-84.5 to 182.9) | | -47.8(-90.1 to 81.0) | -36.1(-41.9 to -29.9) |
| **Republic of Moldova** | 139501(69817 to 222707) | 73499(45304 to 106965) | 160184(102344 to 221135) | 373183(317792 to 430703) |  | 5.8(-33.2 to 45.4) | - | 3387.3(851.3 to 30308.5) | 115.7(96.6 to 137.2) |  | -68.9(-80.4 to -55.1) | -32.4(-50.8 to -9.4) | | -58.0(-69.5 to -43.7) | -60.2(-67.1 to -53.1) |
| **Romania** | 308768(243304 to 383615) | 9277(2776 to 21045) | 20310(6097 to 46871) | 338355(272706 to 412659) |  | -19.4(-30.1 to -12.0) | - | 162.0(-26.5 to 937.7) | -14.1(-19.0 to -8.9) |  | -38.7(-52.0 to -21.3) | -5.3(-75.9 to 238.3) | | -40.9(-85.0 to 110.2) | -38.3(-49.9 to -24.1) |
| **Russian Federation** | 235712(138620 to 331956) | 56215(26526 to 92992) | 122712(61199 to 198834) | 414639(349858 to 481019) |  | -4.1(-38.9 to 29.2) | - | 1891.1(516.9 to 8221.8) | 57.9(52.5 to 73.2) |  | -57.6(-69.7 to -41.7) | -33.9(-56.6 to 4.5) | | -58.8(-73.0 to -35.1) | -55.8(-62.8 to -48.6) |
| **Rwanda** | 5387169(3975553 to 7098131) | 5291(1499 to 13509) | 390699(112368 to 978918) | 5783158(4322233 to 7482276) |  | -68.9(-75.9 to -59.7) | - | 1649.5(284.6 to 16903.3) | -67.2(-74.6 to -58.2) |  | -36.0(-48.4 to -21.6) | 29.6(-65.8 to 417.9) | | -13.0(-77.2 to 244.5) | -34.8(-45.5 to -21.4) |
| **Saint Kitts and Nevis** | 150358(112534 to 189000) | 296(22 to 1512) | 1765(136 to 8320) | 152420(114307 to 191429) |  | -73.4(-78.2 to -68.3) | - | -80.8(-98.6 to 95.3) | -73.4(-78.3 to -68.6) |  | -27.2(-41.1 to -11.9) | 74.4(-68.4 to 798.6) | | 12.7(-79.5 to 483.0) | -26.8(-40.6 to -11.5) |
| **Saint Lucia** | 204345(170925 to 244311) | 337(35 to 1451) | 2027(202 to 8337) | 206709(173519 to 246895) |  | -70.5(-73.6 to -66.5) | - | -88.2(-99.0 to 73.3) | -70.8(-73.8 to -67.4) |  | -17.1(-31.2 to -0.6) | 68.8(-69.4 to 680.7) | | 9.5(-80.1 to 407.1) | -16.8(-31.1 to -0.7) |
| **Saint Vincent and the Grenadines** | 206254(175336 to 242617) | 396(31 to 1654) | 2423(202 to 9823) | 209074(178911 to 245438) |  | -61.2(-66.4 to -53.8) | - | -88.1(-98.9 to 115.8) | -61.9(-66.7 to -56.6) |  | -11.8(-26.2 to 5.0) | 94.0(-65.8 to 878.3) | | 27.1(-77.8 to 535.4) | -11.4(-25.0 to 5.5) |
| **Samoa** | 859107(638699 to 1157782) | 241(20 to 967) | 1255(99 to 5058) | 860603(638833 to 1161415) |  | -45.4(-59.0 to -26.4) | - | 49.9(-86.4 to 1759.8) | -45.3(-59.0 to -26.4) |  | -17.8(-32.9 to -1.2) | -17.3(-83.7 to 413.0) | | -46.9(-89.5 to 231.9) | -17.9(-32.7 to -1.3) |
| **San Marino** | 27842(18640 to 39575) | 180(19 to 721) | 684(73 to 2612) | 28706(19280 to 40712) |  | -64.1(-75.1 to -49.5) | - | -6.8(-90.5 to 1049.1) | -63.3(-74.2 to -48.5) |  | -13.1(-36.3 to 19.1) | 25.8(-76.0 to 505.9) | | -19.8(-84.6 to 287.8) | -13.1(-36.8 to 18.2) |
| **Sao Tome and Principe** | 1652411(1212308 to 2251641) | 1875(212 to 6460) | 138541(15140 to 454890) | 1792827(1392771 to 2416447) |  | -40.7(-55.7 to -16.7) | - | 478.0(-32.5 to 7376.1) | -36.5(-50.3 to -11.9) |  | -27.8(-42.2 to -9.0) | 17.1(-75.0 to 387.5) | | -22.4(-83.4 to 224.3) | -27.4(-39.4 to -14.1) |
| **Saudi Arabia** | 669556(506720 to 878146) | 3232(485 to 10592) | 43247(7503 to 143124) | 716036(572862 to 923980) |  | -63.6(-78.7 to -40.8) | - | 834.7(119.9 to 11220.3) | -61.3(-77.4 to -37.1) |  | -48.6(-59.6 to -35.0) | -16.8(-82.7 to 132.1) | | -45.3(-88.6 to 52.9) | -48.4(-57.5 to -36.4) |
| **Senegal** | 4419883(3382321 to 5648091) | 3331(730 to 9682) | 242867(55184 to 623392) | 4666081(3574217 to 5922739) |  | -46.1(-57.0 to -33.0) | - | 1565.4(219.9 to 19633.4) | -43.2(-53.5 to -30.0) |  | -19.7(-36.0 to -0.6) | 23.6(-73.5 to 449.3) | | -18.4(-82.4 to 265.4) | -19.7(-34.6 to -2.3) |
| **Serbia** | 86217(68512 to 106398) | 710(141 to 2053) | 1547(336 to 4514) | 88474(70713 to 109189) |  | -62.2(-67.0 to -52.6) | - | 65.6(-61.1 to 1342.4) | -61.2(-66.1 to -52.0) |  | -39.5(-52.2 to -24.2) | -27.5(-80.9 to 88.5) | | -54.9(-88.1 to 17.1) | -39.8(-52.4 to -24.9) |
| **Seychelles** | 374485(326682 to 439034) | 357(24 to 1768) | 1864(130 to 9461) | 376706(329287 to 441657) |  | -44.9(-52.3 to -36.7) | - | 263.3(-74.2 to 4888.3) | -44.5(-51.8 to -36.3) |  | -24.4(-34.5 to -11.5) | -28.4(-91.2 to 327.7) | | -53.7(-94.3 to 176.7) | -24.7(-34.7 to -12.3) |
| **Sierra Leone** | 5368182(3913427 to 7328339) | 3848(404 to 14273) | 284356(32772 to 997970) | 5656386(4297561 to 7785508) |  | -26.8(-44.3 to -5.0) | - | 222.9(-61.0 to 2685.7) | -23.8(-38.6 to -3.1) |  | -34.0(-46.8 to -15.2) | -1.3(-77.7 to 285.8) | | -34.6(-85.2 to 156.7) | -34.0(-46.0 to -18.3) |
| **Singapore** | 76722(66061 to 87774) | 500(152 to 1237) | 1895(628 to 4611) | 79117(68604 to 89941) |  | -79.5(-81.3 to -77.5) | - | -39.6(-81.9 to 234.2) | -79.0(-80.8 to -77.1) |  | -44.9(-51.3 to -37.7) | -28.6(-70.9 to 55.7) | | -54.4(-81.4 to -0.4) | -45.1(-51.4 to -37.9) |
| **Slovakia** | 35009(27034 to 44058) | 229(38 to 833) | 502(81 to 1847) | 35740(27706 to 45295) |  | -75.2(-78.2 to -71.7) | - | -51.1(-85.3 to 181.8) | -74.7(-77.7 to -71.3) |  | -33.7(-48.2 to -16.4) | -37.0(-86.7 to 91.0) | | -60.7(-91.7 to 18.6) | -34.4(-48.3 to -17.4) |
| **Slovenia** | 29067(22531 to 37744) | 30(3 to 117) | 65(7 to 253) | 29163(22631 to 37879) |  | -77.3(-82.7 to -70.5) | - | -80.4(-96.6 to 48.4) | -77.3(-82.7 to -70.5) |  | -40.2(-53.8 to -22.9) | -74.6(-95.1 to -12.0) | | -84.1(-97.0 to -45.3) | -40.7(-54.0 to -23.3) |
| **Solomon Islands** | 1192867(977129 to 1439995) | 1617(95 to 6424) | 8169(509 to 32427) | 1202652(986383 to 1450463) |  | -35.3(-49.1 to -17.3) | - | 115.7(-83.7 to 3144.6) | -35.0(-48.8 to -17.2) |  | -27.0(-38.3 to -12.9) | 26.0(-81.2 to 677.9) | | -19.2(-87.8 to 403.4) | -26.9(-37.8 to -13.1) |
| **Somalia** | 19389538(12224838 to 30321065) | 43574(9720 to 129013) | 3189812(711501 to 8858708) | 22622924(16009345 to 33772467) |  | -23.2(-41.4 to 0.0) | - | 12627.3(3101.1 to 127194.6) | -9.9(-28.6 to 15.7) |  | -13.2(-38.2 to 9.9) | 25.0(-62.8 to 261.4) | | -17.1(-75.3 to 140.4) | -13.8(-28.2 to 3.1) |
| **South Africa** | 3779936(3322988 to 4159167) | 3106(841 to 8147) | 229059(63308 to 570511) | 4012102(3677527 to 4376222) |  | 3.3(-13.0 to 21.7) | - | 590.9(98.4 to 3915.1) | 9.2(-5.6 to 28.2) |  | -46.2(-52.4 to -39.9) | -27.8(-79.7 to 138.3) | | -51.6(-86.5 to 58.5) | -46.5(-51.0 to -42.3) |
| **South Sudan** | 8579808(5723749 to 11544553) | 10863(1274 to 31851) | 798757(99070 to 2304645) | 9389428(6713925 to 12548639) |  | -32.0(-49.8 to -12.4) | - | 2574.5(216.1 to 31654.7) | -27.0(-43.0 to -7.0) |  | -14.2(-32.4 to 7.6) | 60.0(-59.3 to 569.9) | | 6.9(-72.9 to 345.6) | -12.7(-27.2 to 4.0) |
| **Spain** | 35523(30526 to 39643) | 242(25 to 1043) | 905(102 to 3811) | 36670(32869 to 40401) |  | -76.3(-78.6 to -74.0) | - | -56.4(-92.3 to 188.4) | -75.8(-77.6 to -74.1) |  | -30.0(-37.5 to -21.8) | -15.8(-82.3 to 185.9) | | -46.7(-88.7 to 82.9) | -30.4(-36.6 to -23.9) |
| **Sri Lanka** | 358543(268969 to 468422) | 591(41 to 2538) | 3067(225 to 13120) | 362201(270449 to 475985) |  | -69.2(-72.4 to -65.4) | - | 148.8(-75.8 to 3197.2) | -68.8(-72.0 to -65.0) |  | -41.5(-56.6 to -23.5) | -34.0(-89.1 to 258.6) | | -57.0(-93.0 to 132.0) | -41.7(-56.7 to -23.9) |
| **Sudan** | 496402(283090 to 734665) | 1690(153 to 7525) | 22803(2100 to 103602) | 520895(303355 to 764548) |  | -64.4(-74.0 to -52.5) | - | 234.4(-67.2 to 4381.1) | -63.0(-72.9 to -50.4) |  | -39.0(-50.3 to -25.0) | -1.8(-79.1 to 383.1) | | -35.1(-86.2 to 218.3) | -38.8(-49.0 to -26.0) |
| **Suriname** | 171227(138640 to 207319) | 242(22 to 920) | 1472(139 to 5845) | 172941(141157 to 208762) |  | -58.2(-63.6 to -51.3) | - | -67.4(-97.1 to 258.6) | -58.3(-63.5 to -51.4) |  | -17.8(-33.3 to -0.3) | 62.7(-70.0 to 849.2) | | 6.3(-80.5 to 516.6) | -17.6(-33.1 to -0.2) |
| **Sweden** | 29249(24229 to 34119) | 496(142 to 1319) | 1884(537 to 4699) | 31629(27121 to 35901) |  | -64.5(-68.7 to -59.8) | - | 76.3(-48.5 to 843.2) | -62.0(-65.5 to -57.9) |  | -28.5(-38.3 to -17.9) | -4.5(-66.1 to 120.9) | | -38.7(-78.3 to 41.4) | -28.9(-36.8 to -20.5) |
| **Switzerland** | 17711(14573 to 20525) | 394(109 to 906) | 1495(422 to 3255) | 19600(17082 to 22171) |  | -75.0(-77.7 to -72.1) | - | -6.5(-74.7 to 486.2) | -74.0(-76.5 to -71.2) |  | -28.0(-38.8 to -17.0) | 101.3(-22.8 to 335.3) | | 29.1(-50.6 to 178.6) | -24.5(-32.4 to -15.4) |
| **Syrian Arab Republic** | 66354(48592 to 88276) | 299(35 to 1229) | 3988(486 to 15339) | 70641(53459 to 91627) |  | -74.5(-81.1 to -63.7) | - | 260.3(-51.1 to 4755.4) | -72.8(-78.7 to -61.4) |  | -13.5(-34.6 to 11.7) | 11.0(-76.3 to 311.8) | | -27.2(-84.4 to 171.4) | -14.3(-32.8 to 8.8) |
| **Taiwan (Province of China)** | 213949(164164 to 271300) | 1903(176 to 7072) | 9994(908 to 38260) | 225846(177871 to 282048) |  | -82.0(-85.3 to -79.8) | - | -47.3(-94.8 to 574.7) | -81.3(-82.9 to -79.8) |  | -26.5(-42.6 to -5.0) | 3.7(-79.9 to 419.7) | | -32.4(-87.0 to 236.3) | -26.6(-42.1 to -8.8) |
| **Tajikistan** | 704490(429780 to 979997) | 100292(37641 to 191914) | 218897(86603 to 411925) | 1023680(836221 to 1261014) |  | 5.0(-27.3 to 37.0) | - | 21117.9(4757.5 to 198722.9) | 42.7(22.3 to 64.6) |  | -30.4(-56.7 to 6.4) | 23.8(-51.9 to 209.9) | | -22.8(-70.1 to 92.5) | -25.6(-40.3 to -6.5) |
| **Thailand** | 749233(550607 to 978749) | 7678(1583 to 21565) | 40209(8516 to 110932) | 797120(603871 to 1026122) |  | -74.9(-79.4 to -69.3) | - | -0.5(-70.7 to 494.3) | -73.6(-78.0 to -68.1) |  | -35.1(-52.2 to -13.4) | -10.9(-78.3 to 152.2) | | -42.0(-86.0 to 63.2) | -35.3(-51.3 to -14.9) |
| **Timor-Leste** | 3630840(2353132 to 4931606) | 7387(656 to 35201) | 38167(3536 to 172053) | 3676394(2366162 to 4970844) |  | -59.1(-70.9 to -44.5) | - | 100.2(-82.3 to 2740.0) | -58.3(-69.9 to -44.1) |  | -9.9(-25.5 to 11.6) | -30.1(-87.1 to 253.5) | | -54.7(-91.7 to 128.7) | -10.9(-26.5 to 8.7) |
| **Togo** | 5073194(3598462 to 7169893) | 4087(477 to 13298) | 299711(37691 to 957554) | 5376992(3853005 to 7526884) |  | -22.4(-43.9 to 0.6) | - | 472.3(-24.5 to 7411.2) | -17.8(-37.1 to 6.0) |  | -31.9(-45.4 to -15.0) | -10.8(-78.4 to 291.6) | | -40.9(-85.6 to 160.5) | -32.5(-43.8 to -18.0) |
| **Tokelau** | 551085(427007 to 711130) | 712(53 to 3474) | 3748(283 to 20267) | 555544(429114 to 713410) |  | -64.6(-72.1 to -54.4) | - | 13.8(-90.0 to 1756.2) | -64.5(-72.0 to -53.9) |  | -33.8(-44.3 to -20.6) | 17.8(-79.4 to 522.4) | | -22.9(-86.7 to 303.2) | -33.7(-44.0 to -21.1) |
| **Tonga** | 563816(437585 to 693546) | 686(55 to 2975) | 3640(295 to 16240) | 568142(441343 to 699341) |  | -43.9(-57.2 to -26.7) | - | 80.9(-85.4 to 2451.1) | -43.6(-57.0 to -26.7) |  | -21.7(-34.9 to -5.4) | 39.5(-76.5 to 656.2) | | -8.4(-84.8 to 389.0) | -21.6(-34.8 to -5.7) |
| **Trinidad and Tobago** | 110927(82319 to 146704) | 216(16 to 1013) | 1292(106 to 5187) | 112435(83213 to 148080) |  | -54.0(-59.2 to -48.0) | - | -65.9(-96.5 to 269.7) | -54.1(-59.0 to -48.4) |  | -22.3(-42.6 to 2.3) | 69.0(-70.4 to 814.6) | | 8.5(-80.8 to 493.9) | -22.0(-42.1 to 2.3) |
| **Tunisia** | 100551(69327 to 140977) | 231(50 to 702) | 3107(705 to 9403) | 103888(72008 to 145999) |  | -65.7(-75.1 to -52.5) | - | 378.2(-28.4 to 5433.9) | -64.8(-74.4 to -51.5) |  | -31.8(-47.2 to -11.8) | 17.7(-70.8 to 423.3) | | -22.7(-80.8 to 244.8) | -31.5(-46.8 to -12.2) |
| **Turkey** | 88782(68801 to 110772) | 600(191 to 1415) | 8084(2768 to 18568) | 97466(77710 to 119558) |  | -84.9(-87.8 to -81.3) | - | 445.2(16.3 to 5087.5) | -83.7(-86.6 to -80.0) |  | -43.4(-56.6 to -27.1) | -3.7(-63.4 to 150.1) | | -36.5(-75.9 to 64.8) | -42.8(-55.2 to -27.1) |
| **Turkmenistan** | 592096(322837 to 858682) | 92472(30456 to 183467) | 202080(70999 to 396783) | 886648(690973 to 1135247) |  | -34.0(-53.8 to -18.9) | - | 12399.8(2979.7 to 127959.3) | -8.2(-15.7 to -0.8) |  | -36.1(-64.9 to 1.0) | 14.5(-59.9 to 187.6) | | -28.6(-75.1 to 78.7) | -31.3(-47.3 to -11.6) |
| **Tuvalu** | 1049228(779302 to 1364051) | 1331(94 to 6265) | 6953(492 to 29854) | 1057511(783054 to 1388617) |  | -61.5(-69.9 to -50.5) | - | 24.7(-91.6 to 1831.8) | -61.3(-69.6 to -50.3) |  | -27.8(-40.7 to -12.8) | 21.1(-78.4 to 770.2) | | -20.5(-86.0 to 463.2) | -27.8(-40.3 to -12.9) |
| **Uganda** | 7340516(5344257 to 9463655) | 7776(1322 to 23722) | 572256(101026 to 1650310) | 7920548(6058906 to 10019285) |  | -38.3(-51.3 to -21.3) | - | 3334.4(729.9 to 38226.3) | -35.0(-48.9 to -16.4) |  | -31.2(-45.1 to -14.9) | 49.6(-69.2 to 295.8) | | -0.3(-79.5 to 163.3) | -29.5(-41.6 to -15.2) |
| **Ukraine** | 329388(164775 to 527585) | 109732(51946 to 174222) | 240290(119938 to 375885) | 679411(546198 to 838456) |  | -4.0(-43.9 to 33.3) | - | 2767.1(676.6 to 24903.6) | 59.6(49.9 to 69.8) |  | -21.2(-61.7 to 42.7) | 70.4(-16.7 to 259.2) | | 6.7(-48.2 to 123.2) | -4.0(-23.6 to 20.6) |
| **United Arab Emirates** | 227832(110058 to 337946) | 782(67 to 3073) | 10535(958 to 41071) | 239149(116955 to 348340) |  | -54.4(-67.5 to -36.6) | - | 298.6(-60.7 to 4219.5) | -52.8(-66.1 to -33.8) |  | -43.5(-57.6 to -27.6) | 1.9(-80.7 to 430.2) | | -32.5(-87.3 to 249.3) | -43.0(-56.2 to -28.2) |
| **United Kingdom** | 30237(27734 to 32097) | 211(63 to 525) | 801(239 to 1909) | 31249(29041 to 32938) |  | -55.8(-57.7 to -53.8) | - | -40.4(-76.0 to 61.2) | -55.3(-56.9 to -53.8) |  | -14.4(-19.2 to -8.9) | 19.5(-54.8 to 161.6) | | -23.2(-71.1 to 67.4) | -14.5(-18.7 to -9.7) |
| **United Republic of Tanzania** | 6453043(4890940 to 8269618) | 5357(703 to 20326) | 395104(53572 to 1380050) | 6853504(5263391 to 8658122) |  | -41.9(-53.2 to -28.5) | - | 1688.4(209.2 to 24122.7) | -39.9(-51.5 to -25.7) |  | -26.0(-39.4 to -10.3) | 88.8(-54.4 to 427.1) | | 25.7(-69.7 to 250.6) | -24.2(-37.1 to -9.3) |
| **United States of America** | 17451(15960 to 18645) | 133(39 to 337) | 504(151 to 1272) | 18088(16823 to 19112) |  | -72.3(-73.7 to -70.3) | - | -89.1(-93.7 to -80.0) | -73.3(-74.3 to -72.3) |  | -6.1(-11.5 to -0.9) | 46.9(-40.7 to 215.8) | | -6.1(-62.0 to 102.1) | -5.8(-10.1 to -1.2) |
| **United States Virgin Islands** | 53318(42901 to 65855) | 93(9 to 367) | 561(56 to 2367) | 53972(43623 to 66430) |  | -62.0(-68.6 to -51.5) | - | -73.7(-97.5 to 219.1) | -62.1(-68.6 to -51.6) |  | -3.5(-19.4 to 14.1) | 100.3(-62.4 to 945.5) | | 29.9(-75.6 to 579.3) | -3.1(-19.4 to 14.5) |
| **Uruguay** | 111574(98590 to 125317) | 205(38 to 664) | 777(144 to 2456) | 112555(99416 to 126264) |  | -72.3(-74.8 to -69.7) | - | -25.8(-88.9 to 581.0) | -72.2(-74.7 to -69.6) |  | -10.5(-21.8 to 0.1) | 56.6(-69.3 to 565.7) | | 0.6(-80.4 to 325.9) | -10.4(-21.3 to 0.2) |
| **Uzbekistan** | 478840(218552 to 744482) | 118305(44542 to 220483) | 257351(98674 to 435840) | 854495(706087 to 1024990) |  | -29.1(-54.5 to -3.8) | - | 25331.0(6792.8 to 338083.7) | 35.3(26.1 to 45.4) |  | -34.4(-65.7 to 6.9) | -19.8(-65.6 to 35.0) | | -50.2(-78.7 to -16.2) | -38.7(-49.7 to -25.8) |
| **Vanuatu** | 2177212(1537936 to 2858190) | 1668(105 to 7523) | 8753(608 to 40866) | 2187633(1543511 to 2884801) |  | -34.9(-51.1 to -13.8) | - | 136.3(-85.9 to 2384.1) | -34.8(-51.1 to -13.6) |  | -16.1(-30.2 to 0.4) | 91.2(-67.5 to 1168.4) | | 23.6(-78.9 to 720.6) | -15.9(-30.1 to 0.9) |
| **Venezuela (Bolivarian Republic of)** | 277196(199954 to 372663) | 1906(194 to 6819) | 11476(1237 to 42301) | 290578(215125 to 389042) |  | -69.9(-74.0 to -66.9) | - | 249.2(-56.4 to 4578.9) | -68.6(-70.8 to -66.1) |  | -4.6(-31.1 to 29.8) | 33.6(-73.3 to 517.5) | | -13.3(-82.6 to 301.1) | -4.8(-29.4 to 26.7) |
| **Viet Nam** | 2015239(1551072 to 2510167) | 33868(8449 to 89783) | 176966(43335 to 441483) | 2226073(1826024 to 2685301) |  | -58.9(-69.1 to -42.4) | - | 322.2(15.8 to 2409.6) | -54.3(-65.3 to -36.7) |  | -33.9(-48.0 to -19.1) | -17.9(-77.4 to 111.3) | | -46.9(-85.4 to 36.7) | -35.0(-44.5 to -22.5) |
| **Yemen** | 656302(451040 to 953548) | 2436(380 to 8255) | 32938(5379 to 113867) | 691676(480911 to 1004494) |  | -65.9(-76.7 to -52.9) | - | 427.7(0.9 to 6371.5) | -64.0(-75.4 to -50.6) |  | -9.8(-25.9 to 11.0) | 20.5(-73.2 to 327.2) | | -20.2(-82.3 to 181.5) | -10.3(-24.9 to 8.9) |
| **Zambia** | 7528909(5413870 to 9844920) | 8453(1160 to 28468) | 620946(92479 to 2015470) | 8158308(6084819 to 10366435) |  | -33.6(-46.0 to -16.7) | - | 548.7(40.2 to 5844.6) | -31.3(-43.9 to -14.2) |  | -45.8(-58.7 to -32.2) | 80.5(-60.4 to 411.9) | | 20.1(-73.6 to 240.5) | -43.4(-53.4 to -30.8) |
| **Zimbabwe** | 10890741(7703629 to 14007706) | 16592(2103 to 56251) | 1216495(157282 to 3729422) | 12123829(9322830 to 14894582) |  | 70.4(20.5 to 119.9) | - | 2363.2(168.0 to 33779.2) | 86.7(46.4 to 132.8) |  | -32.9(-48.2 to -13.1) | 9.6(-72.8 to 357.7) | | -26.7(-81.9 to 204.5) | -32.3(-43.6 to -19.5) |

| **S7** | | | |
| --- | --- | --- | --- |
| **Group** | **Incidence** | **DALYs** | **Deaths** |
|  |  | **(Disability-Adjusted Life Years)** |  |
| **Global** | -38.16(-40.73 to -35.26) | -62.75(-66.61 to -57.98) | -63.49(-67.62 to -57.85) |
| **Afghanistan** | -50.20(-54.66 to -44.63) | -66.75(-74.12 to -56.14) | -62.43(-71.37 to -51.63) |
| **Albania** | -62.50(-65.67 to -58.55) | -85.77(-88.91 to -81.84) | -87.60(-90.67 to -83.28) |
| **Algeria** | -63.99(-66.68 to -61.47) | -81.88(-85.72 to -75.40) | -80.39(-84.83 to -72.04) |
| **American Samoa** | -24.42(-28.77 to -19.30) | -58.19(-65.76 to -49.45) | -64.67(-71.90 to -56.24) |
| **Andorra** | -46.96(-50.59 to -43.08) | -64.98(-75.12 to -53.47) | -66.54(-77.75 to -52.72) |
| **Angola** | -27.06(-32.21 to -21.56) | -69.05(-77.46 to -55.94) | -64.66(-73.84 to -49.91) |
| **Antigua and Barbuda** | -45.04(-49.73 to -39.77) | -64.54(-69.80 to -58.41) | -70.72(-75.64 to -64.25) |
| **Argentina** | -44.23(-50.48 to -36.79) | -73.03(-75.72 to -69.93) | -72.38(-75.08 to -69.09) |
| **Armenia** | -50.35(-55.88 to -44.11) | -49.94(-57.52 to -41.46) | -43.45(-53.88 to -32.00) |
| **Australia** | -34.98(-41.39 to -27.69) | -58.94(-66.20 to -49.80) | -58.53(-67.50 to -46.98) |
| **Austria** | -55.99(-60.32 to -51.43) | -81.64(-83.49 to -79.57) | -82.28(-84.17 to -80.18) |
| **Azerbaijan** | -26.74(-34.96 to -16.79) | -60.53(-68.20 to -50.75) | -57.06(-65.57 to -45.85) |
| **Bahamas** | -48.15(-52.01 to -43.22) | -66.13(-72.90 to -57.69) | -67.85(-74.89 to -58.85) |
| **Bahrain** | -60.05(-63.67 to -56.63) | -77.56(-81.80 to -71.60) | -77.11(-82.22 to -69.49) |
| **Bangladesh** | -70.00(-73.47 to -66.44) | -84.92(-88.20 to -79.37) | -83.30(-87.10 to -76.60) |
| **Barbados** | -47.68(-51.94 to -42.81) | -65.14(-71.14 to -58.54) | -68.25(-74.28 to -60.96) |
| **Belarus** | -53.09(-58.46 to -47.20) | -28.00(-43.31 to -8.30) | -27.95(-45.68 to -6.04) |
| **Belgium** | -39.09(-45.09 to -32.55) | -68.00(-71.17 to -64.53) | -68.27(-72.08 to -64.24) |
| **Belize** | -38.43(-44.47 to -31.48) | -60.25(-66.58 to -52.45) | -60.78(-67.70 to -52.94) |
| **Benin** | -40.34(-46.34 to -33.91) | -57.62(-68.74 to -42.82) | -54.95(-66.74 to -40.21) |
| **Bermuda** | -52.95(-57.42 to -47.91) | -63.64(-69.84 to -57.11) | -83.76(-87.03 to -79.54) |
| **Bhutan** | -70.64(-74.00 to -66.94) | -82.69(-88.71 to -71.24) | -80.35(-86.58 to -67.30) |
| **Bolivia (Plurinational State of)** | -62.63(-66.53 to -58.47) | -86.24(-89.92 to -81.56) | -84.04(-88.07 to -78.82) |
| **Bosnia and Herzegovina** | -55.61(-59.61 to -51.49) | -77.13(-81.87 to -71.58) | -77.94(-82.50 to -72.25) |
| **Botswana** | -31.99(-39.80 to -22.94) | -51.26(-66.32 to -32.14) | -53.09(-67.36 to -33.64) |
| **Brazil** | -42.86(-46.45 to -38.33) | -75.46(-76.99 to -73.80) | -74.55(-76.05 to -72.96) |
| **Brunei Darussalam** | -44.62(-50.44 to -37.72) | -71.52(-76.19 to -65.69) | -67.84(-73.41 to -60.88) |
| **Bulgaria** | -35.22(-41.57 to -28.13) | -53.11(-62.63 to -41.62) | -55.74(-65.76 to -43.17) |
| **Burkina Faso** | -13.40(-22.15 to -4.36) | -34.59(-48.11 to -17.40) | -35.02(-48.23 to -17.63) |
| **Burundi** | -28.65(-35.67 to -21.13) | -54.71(-66.52 to -38.75) | -51.97(-64.33 to -36.54) |
| **Cabo Verde** | -52.53(-57.41 to -47.67) | -69.05(-76.12 to -58.96) | -63.86(-72.93 to -46.22) |
| **Cambodia** | -44.17(-48.70 to -39.55) | -72.02(-79.39 to -62.01) | -69.31(-77.53 to -57.65) |
| **Cameroon** | -37.08(-43.88 to -29.56) | -48.23(-63.04 to -29.93) | -48.51(-62.71 to -30.84) |
| **Canada** | -26.10(-31.38 to -20.96) | -64.00(-67.82 to -60.34) | -67.07(-71.12 to -62.48) |
| **Central African Republic** | -12.70(-17.35 to -7.47) | -16.16(-37.13 to 11.95) | -13.61(-34.44 to 14.82) |
| **Chad** | -22.59(-30.19 to -13.70) | -31.59(-48.77 to -11.52) | -28.99(-48.23 to -7.90) |
| **Chile** | -65.21(-68.42 to -61.59) | -82.41(-84.21 to -80.53) | -79.71(-82.05 to -77.19) |
| **China** | -60.73(-62.36 to -58.99) | -89.17(-90.97 to -87.07) | -90.23(-91.98 to -87.88) |
| **Colombia** | -50.51(-55.87 to -44.83) | -76.72(-81.66 to -70.42) | -77.10(-82.70 to -70.63) |
| **Comoros** | -36.92(-44.25 to -28.73) | -57.62(-70.69 to -6.36) | -56.48(-68.38 to -21.52) |
| **Congo** | -25.04(-29.65 to -20.18) | -66.44(-75.23 to -55.07) | -62.86(-71.87 to -51.30) |
| **Cook Islands** | -30.06(-34.00 to -25.69) | -64.66(-72.15 to -55.36) | -69.09(-75.93 to -60.14) |
| **Costa Rica** | -61.39(-64.84 to -57.43) | -80.17(-84.85 to -74.28) | -82.82(-87.27 to -77.08) |
| **Croatia** | -70.09(-72.88 to -67.15) | -89.98(-92.11 to -87.36) | -90.39(-92.55 to -87.59) |
| **Cuba** | -60.52(-64.70 to -56.08) | -67.93(-73.01 to -62.41) | -72.74(-78.54 to -66.23) |
| **Cyprus** | -61.02(-63.68 to -57.81) | -80.71(-83.40 to -77.35) | -80.75(-83.98 to -75.93) |
| **Czechia** | -65.97(-69.20 to -62.35) | -79.68(-83.39 to -75.81) | -82.35(-86.03 to -78.23) |
| **C么te d'Ivoire** | -28.07(-34.67 to -20.43) | -46.67(-60.83 to -30.60) | -44.11(-59.36 to -27.60) |
| **Democratic People's Republic of Korea** | -33.85(-37.53 to -29.45) | -40.61(-59.16 to -20.06) | -36.42(-58.31 to -13.24) |
| **Democratic Republic of the Congo** | -6.44(-12.02 to -0.83) | -47.54(-59.43 to -31.88) | -40.11(-54.04 to -22.27) |
| **Denmark** | -52.79(-56.88 to -47.98) | -68.97(-72.91 to -65.06) | -69.30(-73.32 to -64.68) |
| **Djibouti** | -46.56(-52.52 to -39.83) | -53.24(-66.57 to -33.43) | -51.57(-64.64 to -33.23) |
| **Dominica** | -38.18(-42.85 to -32.77) | -58.44(-67.59 to -47.05) | -59.46(-68.45 to -48.29) |
| **Dominican Republic** | -36.56(-43.18 to -28.68) | -65.78(-74.37 to -53.57) | -63.28(-72.79 to -49.50) |
| **Ecuador** | -68.80(-71.80 to -65.65) | -85.19(-88.05 to -81.29) | -84.65(-87.70 to -80.42) |
| **Egypt** | -42.41(-48.02 to -35.91) | -72.77(-78.99 to -61.09) | -73.00(-80.71 to -58.02) |
| **El Salvador** | -59.57(-63.55 to -55.01) | -87.62(-90.60 to -84.16) | -87.60(-90.65 to -83.89) |
| **Equatorial Guinea** | -59.23(-63.12 to -54.59) | -90.00(-93.90 to -82.68) | -88.25(-93.07 to -78.38) |
| **Eritrea** | -30.96(-38.71 to -22.34) | -53.08(-66.41 to -33.40) | -49.36(-63.34 to -28.59) |
| **Estonia** | -68.07(-71.30 to -64.16) | -69.66(-76.06 to -62.07) | -69.61(-76.88 to -60.40) |
| **Eswatini** | -47.06(-54.02 to -38.53) | -25.49(-49.70 to 8.61) | -26.37(-49.84 to 6.25) |
| **Ethiopia** | -45.13(-48.01 to -42.06) | -82.36(-87.16 to -76.90) | -80.81(-85.90 to -74.30) |
| **Fiji** | -22.43(-26.70 to -17.90) | -52.43(-64.70 to -35.96) | -54.05(-67.26 to -35.26) |
| **Finland** | -61.53(-65.00 to -57.75) | -82.11(-84.33 to -79.70) | -81.93(-84.51 to -79.12) |
| **France** | -54.20(-58.37 to -50.02) | -78.93(-81.45 to -76.07) | -77.81(-81.41 to -74.04) |
| **Gabon** | -27.02(-31.73 to -21.83) | -66.01(-75.66 to -55.31) | -63.08(-73.65 to -51.54) |
| **Gambia** | -37.84(-44.13 to -30.73) | -29.50(-49.59 to -2.40) | -22.37(-45.51 to 7.34) |
| **Georgia** | -21.76(-29.92 to -12.53) | -53.84(-61.52 to -44.81) | -52.53(-61.73 to -42.03) |
| **Germany** | -47.29(-53.33 to -40.56) | -84.09(-85.93 to -81.93) | -85.20(-86.59 to -83.70) |
| **Ghana** | -32.54(-39.77 to -23.98) | -57.54(-68.65 to -43.96) | -57.58(-68.48 to -42.85) |
| **Greece** | -54.26(-57.82 to -50.18) | -68.20(-71.66 to -64.69) | -69.16(-73.12 to -65.02) |
| **Greenland** | -3.72(-9.58 to 2.37) | -76.05(-81.49 to -69.82) | -75.10(-80.56 to -69.10) |
| **Grenada** | -47.75(-52.19 to -42.42) | -70.62(-75.17 to -65.78) | -74.47(-78.53 to -69.25) |
| **Guam** | -44.77(-48.47 to -41.29) | -51.90(-59.84 to -42.31) | -63.45(-70.65 to -54.11) |
| **Guatemala** | -63.20(-66.79 to -59.10) | -91.89(-93.71 to -89.40) | -91.80(-93.71 to -89.30) |
| **Guinea** | -25.75(-33.56 to -16.94) | -44.03(-59.18 to -25.89) | -38.89(-54.74 to -19.23) |
| **Guinea-Bissau** | -42.01(-48.07 to -35.86) | -59.59(-70.47 to -44.90) | -56.91(-68.25 to -40.08) |
| **Guyana** | -25.98(-34.19 to -16.05) | -53.40(-65.16 to -38.23) | -57.83(-68.62 to -43.89) |
| **Haiti** | -45.17(-50.24 to -39.97) | -68.45(-76.78 to -57.53) | -66.37(-75.00 to -54.81) |
| **Honduras** | -25.72(-32.30 to -18.48) | -63.05(-71.48 to -47.43) | -52.64(-62.88 to -33.09) |
| **Hungary** | -69.96(-72.91 to -66.52) | -90.35(-92.09 to -88.44) | -92.50(-93.92 to -90.77) |
| **Iceland** | -48.15(-51.89 to -43.92) | -81.23(-83.60 to -78.49) | -83.48(-85.95 to -80.68) |
| **India** | -42.85(-47.15 to -37.80) | -69.62(-75.01 to -63.76) | -70.33(-76.24 to -63.62) |
| **Indonesia** | -70.60(-72.58 to -68.39) | -71.13(-75.99 to -64.67) | -66.24(-72.51 to -59.02) |
| **Iran (Islamic Republic of)** | -40.99(-44.04 to -37.86) | -78.51(-81.40 to -70.95) | -75.60(-79.65 to -64.75) |
| **Iraq** | -56.81(-60.73 to -52.89) | -77.46(-83.46 to -66.62) | -74.94(-82.34 to -61.17) |
| **Ireland** | -55.48(-59.44 to -51.50) | -77.35(-80.77 to -73.60) | -77.70(-81.41 to -73.30) |
| **Israel** | -59.09(-62.44 to -55.00) | -77.73(-80.01 to -74.81) | -80.08(-82.61 to -77.04) |
| **Italy** | -50.94(-54.20 to -47.19) | -76.84(-78.63 to -75.06) | -75.62(-77.78 to -73.47) |
| **Jamaica** | -55.40(-58.76 to -51.28) | -68.24(-74.33 to -60.69) | -73.52(-79.62 to -65.91) |
| **Japan** | -71.59(-73.91 to -68.95) | -81.78(-83.24 to -80.67) | -76.94(-79.98 to -75.01) |
| **Jordan** | -67.29(-69.45 to -65.11) | -84.80(-87.71 to -79.41) | -84.81(-88.14 to -77.48) |
| **Kazakhstan** | -44.25(-50.10 to -38.02) | -68.57(-72.82 to -63.69) | -69.51(-73.98 to -64.25) |
| **Kenya** | -13.37(-16.21 to -10.46) | -33.30(-47.98 to -18.91) | -35.65(-51.11 to -21.85) |
| **Kiribati** | -8.07(-12.13 to -3.62) | -44.26(-58.03 to -25.51) | -41.25(-55.70 to -21.03) |
| **Kuwait** | -55.85(-59.48 to -51.35) | -78.86(-82.59 to -75.04) | -78.51(-82.40 to -73.79) |
| **Kyrgyzstan** | -16.26(-25.86 to -5.74) | -38.91(-46.92 to -30.01) | -34.60(-43.78 to -23.90) |
| **Lao People's Democratic Republic** | -51.05(-54.07 to -47.61) | -78.67(-84.20 to -71.10) | -75.65(-81.67 to -66.40) |
| **Latvia** | -56.14(-60.99 to -50.53) | -58.82(-66.74 to -49.06) | -58.81(-67.86 to -47.35) |
| **Lebanon** | -64.82(-67.79 to -61.71) | -79.25(-84.39 to -61.24) | -81.73(-87.27 to -57.47) |
| **Lesotho** | -13.67(-23.47 to 0.06) | 24.86(-12.56 to 75.48) | 22.61(-15.82 to 72.73) |
| **Liberia** | -40.89(-46.91 to -34.21) | -67.72(-76.17 to -54.18) | -63.36(-72.70 to -46.91) |
| **Libya** | -49.60(-53.90 to -44.90) | -64.74(-73.84 to -49.78) | -63.72(-74.83 to -46.26) |
| **Lithuania** | -48.25(-54.10 to -41.29) | -43.37(-53.63 to -30.98) | -41.87(-53.69 to -27.44) |
| **Luxembourg** | -45.44(-49.59 to -40.58) | -76.60(-79.74 to -72.73) | -79.48(-82.70 to -75.81) |
| **Madagascar** | -35.44(-41.83 to -28.68) | -52.16(-63.90 to -37.66) | -45.94(-59.26 to -27.75) |
| **Malawi** | -49.32(-55.58 to -42.55) | -55.29(-65.68 to -41.95) | -51.89(-62.64 to -38.69) |
| **Malaysia** | -10.77(-17.00 to -3.95) | -61.06(-68.93 to -51.44) | -61.87(-70.37 to -49.90) |
| **Maldives** | -63.02(-65.90 to -59.69) | -92.20(-94.06 to -89.14) | -91.57(-93.53 to -88.21) |
| **Mali** | -37.18(-43.66 to -29.67) | -57.08(-67.18 to -42.45) | -57.30(-66.83 to -40.04) |
| **Malta** | -25.65(-32.45 to -17.41) | -69.02(-73.65 to -63.65) | -77.31(-80.86 to -73.36) |
| **Marshall Islands** | -39.66(-43.02 to -36.23) | -51.03(-63.68 to -35.00) | -52.91(-65.47 to -36.66) |
| **Mauritania** | -45.13(-50.51 to -39.18) | -74.97(-82.12 to -63.61) | -72.47(-79.60 to -58.71) |
| **Mauritius** | -39.22(-43.75 to -34.78) | -64.87(-71.39 to -57.54) | -71.22(-77.56 to -63.54) |
| **Mexico** | -37.13(-42.24 to -31.49) | -82.85(-85.26 to -80.09) | -85.26(-87.53 to -82.68) |
| **Micronesia (Federated States of)** | -30.52(-34.54 to -26.24) | -65.34(-76.67 to -52.49) | -66.12(-76.02 to -53.54) |
| **Monaco** | -49.63(-52.65 to -46.08) | -68.47(-75.70 to -59.02) | -67.78(-74.94 to -57.95) |
| **Mongolia** | -26.86(-35.25 to -16.41) | -62.53(-71.25 to -51.28) | -60.83(-70.61 to -48.27) |
| **Montenegro** | -42.02(-47.10 to -36.64) | -56.88(-64.74 to -47.47) | -52.09(-61.68 to -39.02) |
| **Morocco** | -50.98(-55.55 to -45.63) | -73.48(-80.28 to -51.34) | -71.12(-78.60 to -44.46) |
| **Mozambique** | -35.51(-42.55 to -27.50) | -41.19(-56.73 to -20.79) | -40.37(-55.02 to -19.31) |
| **Myanmar** | -61.87(-64.36 to -59.13) | -78.90(-84.90 to -70.96) | -76.93(-82.70 to -68.86) |
| **Namibia** | -38.83(-45.26 to -30.45) | -51.12(-66.51 to -25.80) | -51.04(-65.27 to -26.69) |
| **Nauru** | -19.31(-23.75 to -14.33) | -53.50(-61.67 to -41.99) | -54.90(-63.54 to -42.92) |
| **Nepal** | -67.87(-71.19 to -63.90) | -78.29(-84.07 to -70.14) | -74.06(-81.37 to -62.57) |
| **Netherlands** | -45.17(-49.92 to -39.46) | -71.45(-74.63 to -67.88) | -71.95(-75.74 to -67.96) |
| **New Zealand** | -44.89(-48.49 to -41.06) | -73.48(-76.88 to -70.12) | -75.04(-78.28 to -71.40) |
| **Nicaragua** | -52.07(-57.02 to -47.19) | -82.69(-85.84 to -78.97) | -79.63(-83.39 to -75.17) |
| **Niger** | -36.29(-42.87 to -29.09) | -60.83(-71.44 to -45.27) | -55.26(-65.98 to -38.38) |
| **Nigeria** | -43.00(-46.58 to -38.88) | -62.87(-72.81 to -50.78) | -60.74(-71.72 to -45.79) |
| **Niue** | -29.48(-33.48 to -25.09) | -67.00(-74.75 to -55.93) | -70.01(-77.44 to -59.37) |
| **North Macedonia** | -60.55(-64.11 to -56.53) | -83.56(-87.01 to -79.18) | -82.38(-86.12 to -77.19) |
| **Northern Mariana Islands** | -66.66(-68.64 to -64.61) | -74.00(-78.98 to -68.02) | -77.83(-82.51 to -72.37) |
| **Norway** | -42.89(-47.76 to -38.14) | -67.25(-69.31 to -65.09) | -62.68(-65.69 to -59.74) |
| **Oman** | -65.99(-68.63 to -62.94) | -84.03(-88.96 to -76.57) | -82.67(-89.06 to -70.78) |
| **Pakistan** | -47.25(-51.36 to -42.67) | -53.35(-63.14 to -39.77) | -52.06(-63.03 to -35.38) |
| **Palau** | -16.86(-21.58 to -11.59) | -53.19(-65.29 to -38.36) | -56.23(-68.70 to -39.21) |
| **Palestine** | -62.79(-65.84 to -59.37) | -76.02(-86.10 to -62.66) | -73.88(-86.33 to -52.65) |
| **Panama** | -34.59(-41.00 to -27.56) | -60.64(-70.40 to -48.04) | -61.61(-71.55 to -48.64) |
| **Papua New Guinea** | -37.50(-40.55 to -34.23) | -50.61(-61.64 to -36.93) | -49.98(-61.58 to -35.60) |
| **Paraguay** | -29.34(-36.27 to -20.48) | -55.74(-66.44 to -42.67) | -54.17(-66.13 to -38.96) |
| **Peru** | -71.29(-73.98 to -68.42) | -89.95(-92.66 to -85.90) | -88.81(-92.02 to -83.92) |
| **Philippines** | -48.35(-51.84 to -44.84) | -55.64(-64.05 to -45.84) | -56.37(-65.63 to -44.82) |
| **Poland** | -60.12(-63.61 to -56.34) | -78.69(-82.19 to -74.58) | -81.39(-84.60 to -77.45) |
| **Portugal** | -59.79(-63.32 to -55.64) | -78.11(-80.27 to -75.86) | -75.86(-78.41 to -73.20) |
| **Puerto Rico** | -64.55(-67.96 to -61.05) | -83.95(-87.10 to -79.94) | -87.23(-90.18 to -83.22) |
| **Qatar** | -65.60(-68.71 to -62.60) | -81.12(-85.64 to -74.51) | -76.22(-82.79 to -66.08) |
| **Republic of Korea** | -68.98(-72.50 to -64.56) | -89.92(-90.87 to -88.72) | -85.69(-87.45 to -83.68) |
| **Republic of Moldova** | -17.61(-27.68 to -5.80) | -22.20(-33.17 to -10.45) | -14.23(-28.06 to 0.93) |
| **Romania** | -19.62(-28.51 to -9.70) | -53.77(-61.72 to -44.56) | -46.99(-57.14 to -35.47) |
| **Russian Federation** | -26.50(-33.41 to -17.90) | -25.43(-35.35 to -14.93) | -30.29(-40.63 to -18.93) |
| **Rwanda** | -53.97(-58.88 to -48.39) | -80.46(-85.53 to -73.65) | -78.63(-83.89 to -70.03) |
| **Saint Kitts and Nevis** | -48.40(-52.84 to -42.96) | -80.45(-86.38 to -73.96) | -80.53(-85.66 to -75.01) |
| **Saint Lucia** | -52.78(-56.75 to -48.16) | -74.36(-78.60 to -68.89) | -75.69(-80.00 to -70.37) |
| **Saint Vincent and the Grenadines** | -45.08(-49.44 to -40.07) | -66.54(-72.09 to -59.70) | -66.23(-71.77 to -58.89) |
| **Samoa** | -16.44(-21.18 to -11.83) | -53.35(-66.63 to -35.69) | -55.10(-67.31 to -38.83) |
| **San Marino** | -53.01(-56.06 to -49.84) | -66.87(-76.85 to -53.77) | -68.14(-79.36 to -53.40) |
| **Sao Tome and Principe** | -39.12(-44.92 to -32.21) | -56.99(-67.73 to -39.34) | -53.89(-65.42 to -34.84) |
| **Saudi Arabia** | -56.47(-61.45 to -50.29) | -77.63(-87.10 to -59.63) | -80.04(-88.92 to -63.29) |
| **Senegal** | -37.94(-44.14 to -30.71) | -58.01(-69.44 to -42.49) | -54.40(-66.04 to -39.10) |
| **Serbia** | -53.55(-57.70 to -49.05) | -78.24(-82.87 to -72.32) | -76.65(-82.05 to -69.04) |
| **Seychelles** | -15.23(-21.05 to -9.10) | -56.56(-62.07 to -49.96) | -58.18(-64.07 to -51.24) |
| **Sierra Leone** | -22.69(-30.56 to -13.63) | -52.61(-65.70 to -33.54) | -49.75(-62.54 to -29.09) |
| **Singapore** | -53.62(-58.41 to -47.88) | -87.56(-88.98 to -86.07) | -88.45(-89.94 to -86.72) |
| **Slovakia** | -62.86(-65.94 to -59.77) | -81.83(-85.73 to -77.01) | -83.39(-87.41 to -78.63) |
| **Slovenia** | -68.35(-71.21 to -65.16) | -86.23(-90.07 to -81.51) | -86.51(-90.62 to -81.18) |
| **Solomon Islands** | -16.95(-21.01 to -12.67) | -51.29(-62.92 to -35.40) | -52.53(-63.61 to -37.03) |
| **Somalia** | -12.93(-19.88 to -4.79) | -24.99(-43.17 to 1.18) | -22.30(-41.20 to 4.06) |
| **South Africa** | -12.00(-19.60 to -3.69) | -48.84(-55.53 to -42.59) | -41.61(-49.06 to -33.83) |
| **South Sudan** | -27.49(-34.53 to -20.11) | -39.71(-54.28 to -18.27) | -36.33(-52.10 to -14.20) |
| **Spain** | -66.33(-69.39 to -62.65) | -85.48(-86.67 to -84.14) | -83.15(-84.80 to -81.41) |
| **Sri Lanka** | -9.13(-15.13 to -2.90) | -77.86(-82.92 to -71.70) | -81.82(-86.68 to -75.66) |
| **Sudan** | -52.39(-57.13 to -46.86) | -80.09(-85.75 to -71.90) | -77.32(-83.66 to -68.67) |
| **Suriname** | -39.27(-45.13 to -32.63) | -64.97(-71.83 to -55.54) | -65.60(-72.68 to -56.93) |
| **Sweden** | -0.22(-9.40 to 9.31) | -71.39(-74.51 to -67.82) | -72.99(-76.43 to -69.30) |
| **Switzerland** | -43.87(-49.44 to -37.44) | -78.65(-81.37 to -75.51) | -80.36(-82.68 to -77.66) |
| **Syrian Arab Republic** | -60.95(-64.57 to -56.87) | -78.95(-84.02 to -70.03) | -76.67(-83.52 to -63.84) |
| **Taiwan (Province of China)** | -39.91(-44.56 to -34.46) | -82.69(-85.57 to -79.60) | -86.30(-89.18 to -82.95) |
| **Tajikistan** | -0.97(-10.83 to 9.45) | -12.85(-29.04 to 8.15) | 6.10(-13.85 to 31.43) |
| **Thailand** | -57.82(-60.85 to -54.62) | -83.18(-87.10 to -77.80) | -82.95(-87.43 to -76.95) |
| **Timor-Leste** | -19.73(-24.32 to -14.64) | -67.62(-78.07 to -54.87) | -62.83(-73.12 to -47.81) |
| **Togo** | -33.42(-40.44 to -26.01) | -46.23(-59.41 to -28.09) | -44.50(-59.24 to -26.21) |
| **Tokelau** | -39.12(-42.97 to -34.93) | -75.09(-81.66 to -66.61) | -76.44(-82.70 to -68.08) |
| **Tonga** | -18.84(-23.16 to -14.28) | -52.73(-63.90 to -37.82) | -55.80(-66.90 to -40.69) |
| **Trinidad and Tobago** | -43.29(-48.92 to -36.89) | -60.29(-69.90 to -48.52) | -64.21(-73.88 to -51.82) |
| **Tunisia** | -57.14(-60.43 to -53.52) | -76.86(-82.30 to -67.90) | -75.90(-82.89 to -61.84) |
| **Turkey** | -66.97(-70.35 to -62.99) | -90.85(-92.76 to -88.55) | -90.66(-92.94 to -87.69) |
| **Turkmenistan** | -31.71(-38.70 to -23.40) | -39.08(-51.35 to -24.06) | -36.94(-50.90 to -20.05) |
| **Tuvalu** | -36.03(-39.53 to -32.13) | -73.05(-79.49 to -63.54) | -72.07(-79.01 to -62.07) |
| **Uganda** | -10.59(-20.14 to -0.55) | -51.87(-64.30 to -34.77) | -54.19(-65.81 to -36.77) |
| **Ukraine** | 10.76(-0.12 to 24.57) | 74.85(42.95 to 113.92) | 53.26(22.00 to 94.57) |
| **United Arab Emirates** | -48.34(-53.76 to -42.52) | -70.17(-78.57 to -59.10) | -73.07(-82.85 to -60.05) |
| **United Kingdom** | -44.22(-47.17 to -40.86) | -61.66(-63.06 to -60.22) | -61.75(-63.67 to -59.99) |
| **United Republic of Tanzania** | -46.11(-51.19 to -40.50) | -55.35(-65.60 to -42.43) | -54.42(-65.34 to -40.34) |
| **United States of America** | -72.07(-73.90 to -70.28) | -74.60(-76.16 to -73.04) | -74.87(-76.38 to -73.34) |
| **United States Virgin Islands** | -45.66(-50.44 to -40.22) | -61.81(-69.93 to -49.20) | -63.28(-71.51 to -50.58) |
| **Uruguay** | -31.89(-38.55 to -24.50) | -70.72(-74.27 to -67.00) | -75.10(-78.23 to -71.55) |
| **Uzbekistan** | -32.25(-39.59 to -24.34) | -24.94(-37.76 to -9.37) | -17.13(-32.59 to 0.74) |
| **Vanuatu** | -16.08(-20.46 to -11.32) | -43.46(-58.80 to -20.83) | -45.14(-60.30 to -23.02) |
| **Venezuela (Bolivarian Republic of)** | -39.79(-45.89 to -33.03) | -67.63(-75.60 to -57.24) | -70.06(-77.88 to -59.72) |
| **Viet Nam** | -27.87(-32.50 to -23.47) | -71.27(-79.68 to -59.00) | -70.31(-78.96 to -55.87) |
| **Yemen** | -44.26(-49.56 to -38.52) | -70.07(-79.96 to -56.83) | -67.67(-78.85 to -53.39) |
| **Zambia** | -42.89(-49.82 to -34.92) | -62.01(-71.01 to -49.80) | -61.13(-70.49 to -48.22) |
| **Zimbabwe** | -26.93(-36.77 to -15.89) | 31.76(-2.88 to 73.77) | 26.35(-5.39 to 64.68) |

| **S8** | | | | | | | | |
| --- | --- | --- | --- | --- | --- | --- | --- | --- |
| **Group** | **Incidence** | |  | **DALYs (Disability-Adjusted Life Years)** | |  | **Deaths** | |
|  | **Male** | **Female** |  | **Male** | **Female** |  | **Male** | **Female** |
| **1990** | 18248.26(15984.99 to 20828.62) | 16330.13(14154.87 to 18793.97) |  | 189956.24(175483.13 to 203721.52) | 127274.45(107600.13 to 147485.86) |  | 5298.99(4862.93 to 5726.17) | 2880.34(2381.89 to 3410.67) |
| **1991** | 17596.87(15395.24 to 20128.40) | 15802.25(13691.41 to 18173.44) |  | 186358.62(172269.37 to 200734.84) | 124545.67(105607.31 to 145347.26) |  | 5200.07(4774.15 to 5662.83) | 2818.29(2338.33 to 3361.34) |
| **1992** | 16987.16(14877.98 to 19437.69) | 15309.58(13287.33 to 17588.44) |  | 182966.68(169543.83 to 197287.46) | 123326.44(103695.77 to 143457.81) |  | 5111.60(4676.82 to 5534.09) | 2796.17(2285.18 to 3326.25) |
| **1993** | 16455.76(14427.58 to 18823.37) | 14878.09(12922.96 to 17097.35) |  | 176004.95(162353.73 to 189575.41) | 117274.68(99939.55 to 135524.33) |  | 4913.06(4510.88 to 5298.13) | 2653.96(2208.29 to 3138.06) |
| **1994** | 16036.05(14075.00 to 18354.05) | 14533.15(12627.26 to 16670.41) |  | 170333.97(157905.95 to 183011.57) | 112422.73(97625.61 to 128803.43) |  | 4738.85(4369.94 to 5069.91) | 2535.05(2150.52 to 2960.70) |
| **1995** | 15761.21(13827.58 to 18051.11) | 14296.60(12405.65 to 16398.63) |  | 165092.59(152084.17 to 177188.70) | 108439.07(94032.02 to 124678.72) |  | 4576.43(4187.78 to 4898.43) | 2438.76(2067.56 to 2842.66) |
| **1996** | 15582.84(13682.30 to 17837.08) | 14130.51(12262.89 to 16202.75) |  | 159568.62(145741.75 to 171740.89) | 104365.59(90794.15 to 119584.14) |  | 4411.42(4003.29 to 4747.25) | 2347.88(2006.63 to 2735.47) |
| **1997** | 15417.37(13536.06 to 17609.79) | 13974.47(12109.86 to 16025.99) |  | 156318.83(143162.45 to 168430.37) | 101594.03(88474.93 to 115695.72) |  | 4319.94(3918.28 to 4643.97) | 2290.00(1947.01 to 2626.99) |
| **1998** | 15266.10(13407.20 to 17476.72) | 13827.07(11982.46 to 15857.87) |  | 152492.95(139618.38 to 164884.66) | 98813.81(85731.13 to 112511.94) |  | 4208.48(3824.70 to 4523.16) | 2228.74(1889.16 to 2554.07) |
| **1999** | 15129.28(13278.98 to 17310.98) | 13686.02(11844.44 to 15694.52) |  | 148630.84(135389.01 to 160637.28) | 96279.82(83071.53 to 109276.84) |  | 4074.03(3701.44 to 4400.44) | 2165.40(1821.56 to 2469.42) |
| **2000** | 15003.50(13183.90 to 17164.01) | 13547.39(11724.77 to 15514.46) |  | 145021.35(131974.07 to 156625.36) | 93528.04(81176.06 to 106045.66) |  | 3967.83(3616.49 to 4265.12) | 2097.91(1786.58 to 2372.62) |
| **2001** | 14863.61(13051.43 to 17003.79) | 13346.36(11545.58 to 15307.30) |  | 138975.81(126636.59 to 149972.61) | 89550.31(77477.96 to 101792.54) |  | 3792.91(3464.41 to 4091.04) | 2009.46(1713.80 to 2276.51) |
| **2002** | 14688.63(12904.50 to 16782.18) | 13050.87(11289.08 to 14968.06) |  | 133257.13(121008.49 to 143265.49) | 85253.97(74631.28 to 96931.87) |  | 3630.87(3296.19 to 3904.70) | 1918.17(1649.21 to 2175.58) |
| **2003** | 14498.94(12733.68 to 16547.59) | 12714.90(11010.91 to 14568.18) |  | 127694.65(116353.50 to 138128.97) | 81070.52(70738.04 to 91971.95) |  | 3474.18(3156.17 to 3740.33) | 1821.43(1571.63 to 2065.24) |
| **2004** | 14309.46(12558.06 to 16338.71) | 12390.31(10763.08 to 14210.62) |  | 121475.13(111212.77 to 131276.95) | 76896.25(67630.82 to 87173.18) |  | 3278.32(3001.62 to 3550.74) | 1717.76(1507.54 to 1946.02) |
| **2005** | 14134.98(12402.74 to 16136.77) | 12127.77(10565.03 to 13948.49) |  | 117046.74(106173.17 to 127129.57) | 73722.52(65267.18 to 82820.47) |  | 3146.45(2868.09 to 3405.48) | 1648.31(1447.80 to 1858.14) |
| **2006** | 13929.20(12249.31 to 15920.33) | 11877.77(10344.52 to 13633.96) |  | 112456.14(102630.29 to 121870.63) | 70398.27(62386.86 to 79587.26) |  | 3009.36(2746.49 to 3250.75) | 1576.88(1395.58 to 1771.51) |
| **2007** | 13653.30(11992.20 to 15563.50) | 11574.81(10063.55 to 13312.12) |  | 108411.60(99253.79 to 117527.65) | 66961.24(59284.82 to 75241.85) |  | 2889.59(2645.20 to 3121.86) | 1503.03(1326.10 to 1687.58) |
| **2008** | 13350.05(11707.36 to 15205.36) | 11254.24(9793.35 to 12954.05) |  | 104787.67(95528.30 to 113588.83) | 64119.79(57212.07 to 72013.92) |  | 2787.83(2544.86 to 3017.21) | 1439.38(1280.42 to 1611.79) |
| **2009** | 13062.44(11462.90 to 14878.50) | 10950.46(9547.90 to 12608.30) |  | 100009.33(90848.37 to 108625.04) | 60881.97(54394.69 to 67853.48) |  | 2652.68(2411.86 to 2875.06) | 1361.81(1215.15 to 1519.72) |
| **2010** | 12829.81(11249.86 to 14631.81) | 10696.37(9301.03 to 12319.69) |  | 94962.06(85681.69 to 103444.07) | 58149.76(51892.81 to 64694.04) |  | 2510.74(2270.37 to 2721.82) | 1300.03(1165.78 to 1452.51) |
| **2011** | 12609.38(11069.60 to 14344.79) | 10447.18(9114.00 to 12007.37) |  | 90627.61(81657.91 to 98845.41) | 55467.26(49179.63 to 61871.90) |  | 2387.58(2152.92 to 2591.78) | 1244.96(1107.83 to 1396.93) |
| **2012** | 12349.31(10864.96 to 14069.52) | 10157.44(8868.33 to 11632.71) |  | 88211.43(79295.93 to 96611.70) | 52862.41(46694.54 to 59201.05) |  | 2318.43(2086.11 to 2530.11) | 1191.92(1053.82 to 1328.95) |
| **2013** | 12086.10(10658.06 to 13756.23) | 9864.25(8595.88 to 11270.01) |  | 85513.74(76126.96 to 93825.70) | 50918.16(45189.67 to 57309.26) |  | 2255.76(1995.93 to 2467.71) | 1152.99(1021.81 to 1304.91) |
| **2014** | 11857.01(10438.17 to 13436.18) | 9606.39(8353.27 to 10974.64) |  | 82884.20(73693.92 to 90494.09) | 49081.67(42927.71 to 55204.88) |  | 2190.98(1946.09 to 2379.18) | 1117.11(977.53 to 1261.77) |
| **2015** | 11696.36(10248.57 to 13248.64) | 9422.51(8161.47 to 10779.04) |  | 79159.40(70091.19 to 87519.99) | 47303.66(41031.22 to 53382.32) |  | 2081.77(1847.48 to 2291.17) | 1080.24(942.38 to 1224.33) |
| **2016** | 11589.43(10177.38 to 13100.67) | 9265.18(8067.05 to 10614.81) |  | 76362.38(67076.19 to 84586.47) | 45243.43(39198.56 to 50999.36) |  | 2007.28(1768.90 to 2216.03) | 1035.99(894.89 to 1186.84) |
| **2017** | 11519.06(10129.92 to 13000.68) | 9154.77(7981.49 to 10498.84) |  | 73689.08(65109.52 to 81827.73) | 43225.38(37109.31 to 49704.62) |  | 1938.71(1706.29 to 2155.62) | 992.03(851.64 to 1155.63) |
| **2018** | 11428.71(9970.54 to 12960.15) | 9079.87(7887.45 to 10429.68) |  | 70696.89(61542.96 to 79638.88) | 41376.07(35680.37 to 48123.29) |  | 1854.39(1616.92 to 2090.02) | 948.28(818.78 to 1129.08) |
| **2019** | 11281.35(9794.19 to 12887.24) | 8979.85(7766.86 to 10325.23) |  | 67808.96(58247.38 to 76286.90) | 39640.13(33770.03 to 46100.38) |  | 1773.95(1536.06 to 1995.87) | 908.12(766.83 to 1093.71) |

| **S9** | | | | | | | | |
| --- | --- | --- | --- | --- | --- | --- | --- | --- |
| **Group** | **Incidence** | |  | **DALYs (Disability-Adjusted Life Years)** | |  | **Deaths** | |
|  | **Male** | **Female** |  | **Male** | **Female** |  | **Male** | **Female** |
| **1990** | 880.26(779.40 to 994.73) | 1019.81(902.59 to 1147.81) |  | 12152.77(9372.21 to 15582.33) | 14822.08(11318.00 to 19439.32) |  | 206.47(153.63 to 272.18) | 241.15(178.58 to 321.85) |
| **1991** | 1059.49(937.26 to 1196.31) | 1224.28(1080.10 to 1380.26) |  | 14779.26(11481.93 to 18952.91) | 18382.43(14075.51 to 23958.70) |  | 251.52(187.96 to 329.91) | 300.99(223.09 to 400.58) |
| **1992** | 1229.08(1083.67 to 1387.49) | 1421.36(1251.81 to 1606.42) |  | 17696.68(13701.46 to 22774.66) | 22283.14(17065.90 to 29131.72) |  | 302.48(226.24 to 396.15) | 367.43(272.95 to 487.49) |
| **1993** | 1385.02(1218.83 to 1569.05) | 1606.36(1408.50 to 1818.64) |  | 20820.90(16044.02 to 26666.15) | 26432.27(20256.40 to 34250.19) |  | 357.81(267.12 to 467.71) | 439.09(329.31 to 580.36) |
| **1994** | 1525.06(1336.77 to 1731.65) | 1778.12(1555.49 to 2016.10) |  | 23966.69(18337.87 to 30663.22) | 30640.00(23394.67 to 39796.61) |  | 414.11(310.47 to 540.76) | 512.70(383.45 to 674.22) |
| **1995** | 1644.36(1435.03 to 1870.96) | 1931.40(1685.51 to 2193.51) |  | 26980.13(20572.80 to 34542.36) | 34691.12(26355.84 to 44947.88) |  | 468.57(351.50 to 614.16) | 584.39(436.96 to 768.31) |
| **1996** | 1758.39(1530.54 to 2003.54) | 2083.49(1814.63 to 2370.20) |  | 29612.05(22382.24 to 38251.11) | 38314.40(28882.77 to 49700.47) |  | 516.58(383.82 to 679.73) | 649.52(482.25 to 852.65) |
| **1997** | 1876.97(1631.37 to 2143.42) | 2244.45(1953.40 to 2553.39) |  | 32089.83(24233.51 to 41537.15) | 41830.86(31358.00 to 54241.11) |  | 561.84(415.77 to 741.72) | 713.22(525.36 to 941.49) |
| **1998** | 1989.17(1727.63 to 2273.62) | 2398.97(2083.53 to 2733.05) |  | 34556.49(25876.87 to 44796.99) | 45264.27(33773.39 to 58772.07) |  | 607.06(444.66 to 801.38) | 775.36(569.88 to 1021.58) |
| **1999** | 2085.68(1811.67 to 2389.08) | 2533.55(2202.03 to 2890.47) |  | 36646.32(27236.89 to 47427.38) | 48161.01(35598.79 to 62689.66) |  | 646.50(468.31 to 850.07) | 828.53(600.36 to 1090.84) |
| **2000** | 2155.06(1869.44 to 2472.84) | 2633.23(2291.16 to 3006.23) |  | 38316.84(28212.88 to 49693.46) | 50707.57(37266.02 to 65844.00) |  | 679.14(488.94 to 890.77) | 876.53(630.46 to 1151.04) |
| **2001** | 2203.13(1908.09 to 2527.00) | 2705.12(2347.63 to 3087.64) |  | 39070.49(28484.36 to 50597.86) | 52134.13(37654.39 to 67532.79) |  | 695.07(493.94 to 910.10) | 904.93(642.34 to 1188.11) |
| **2002** | 2240.84(1938.95 to 2571.13) | 2762.91(2393.25 to 3155.58) |  | 39443.44(28126.18 to 51111.93) | 52964.37(37535.50 to 68599.59) |  | 703.93(496.17 to 918.40) | 922.86(644.40 to 1207.75) |
| **2003** | 2264.51(1961.63 to 2596.93) | 2800.74(2426.26 to 3204.10) |  | 39442.14(27899.21 to 50869.55) | 53290.74(37450.69 to 69057.95) |  | 706.02(491.52 to 916.78) | 932.11(642.36 to 1217.80) |
| **2004** | 2271.28(1969.39 to 2600.76) | 2813.90(2432.38 to 3220.82) |  | 38709.53(27282.82 to 49641.55) | 52604.95(36599.65 to 68054.61) |  | 693.69(478.51 to 897.90) | 922.24(630.32 to 1193.77) |
| **2005** | 2258.21(1958.08 to 2585.17) | 2798.27(2415.74 to 3203.82) |  | 37184.42(26153.21 to 47684.55) | 50744.08(35302.31 to 65170.57) |  | 666.29(458.02 to 861.86) | 890.29(603.76 to 1145.54) |
| **2006** | 2213.16(1921.52 to 2533.65) | 2738.00(2370.89 to 3133.17) |  | 34954.31(24688.39 to 44769.39) | 47914.11(33542.03 to 61210.07) |  | 626.18(431.93 to 805.93) | 840.81(573.24 to 1073.33) |
| **2007** | 2135.70(1857.39 to 2444.11) | 2634.39(2283.50 to 3012.48) |  | 32347.04(23051.97 to 41433.35) | 44245.58(31278.49 to 56273.10) |  | 578.21(401.92 to 742.41) | 774.92(534.50 to 985.90) |
| **2008** | 2041.08(1777.19 to 2335.28) | 2508.68(2176.11 to 2868.78) |  | 29715.32(21424.60 to 37855.43) | 40448.40(28978.12 to 51488.42) |  | 529.13(372.13 to 676.08) | 705.87(492.24 to 899.21) |
| **2009** | 1946.09(1697.29 to 2223.81) | 2382.55(2066.78 to 2720.95) |  | 27101.67(19561.51 to 34425.40) | 36825.15(26543.29 to 46922.60) |  | 481.47(340.03 to 615.05) | 640.97(449.92 to 819.23) |
| **2010** | 1867.13(1631.30 to 2129.73) | 2277.44(1975.09 to 2596.17) |  | 24908.92(18004.04 to 31633.25) | 33471.53(24104.12 to 42640.59) |  | 442.80(313.31 to 567.54) | 581.52(407.64 to 746.56) |
| **2011** | 1793.77(1570.12 to 2044.87) | 2177.53(1895.60 to 2483.59) |  | 22966.79(16593.61 to 29324.97) | 30140.93(21736.69 to 38431.10) |  | 409.45(289.45 to 527.06) | 522.82(363.00 to 667.31) |
| **2012** | 1710.16(1497.76 to 1948.16) | 2061.11(1792.86 to 2351.06) |  | 21310.53(15546.44 to 27248.15) | 27113.78(19627.29 to 34597.50) |  | 381.18(270.67 to 491.68) | 469.46(328.32 to 599.54) |
| **2013** | 1624.15(1425.02 to 1848.50) | 1940.99(1690.30 to 2215.68) |  | 19828.42(14534.45 to 25312.56) | 24496.12(17799.20 to 31299.24) |  | 355.74(253.70 to 458.14) | 423.62(300.44 to 541.17) |
| **2014** | 1544.12(1354.80 to 1754.34) | 1830.98(1599.64 to 2093.96) |  | 18537.49(13695.97 to 23604.28) | 22389.34(16453.65 to 28484.18) |  | 333.69(237.66 to 428.61) | 387.49(277.81 to 495.43) |
| **2015** | 1478.43(1299.34 to 1678.04) | 1745.11(1524.97 to 1987.58) |  | 17331.14(12799.29 to 22132.30) | 20587.00(15189.09 to 26259.45) |  | 313.55(224.18 to 403.47) | 357.12(254.34 to 457.00) |
| **2016** | 1422.74(1252.88 to 1615.65) | 1678.96(1471.88 to 1909.51) |  | 16264.51(11990.61 to 20791.50) | 18991.12(13952.80 to 24321.92) |  | 295.84(210.37 to 383.53) | 330.33(234.53 to 426.85) |
| **2017** | 1372.76(1215.70 to 1561.26) | 1619.99(1426.12 to 1842.32) |  | 15060.46(11090.06 to 19296.79) | 17254.39(12617.80 to 22173.68) |  | 274.97(194.66 to 357.52) | 300.44(212.84 to 391.25) |
| **2018** | 1321.44(1162.66 to 1502.72) | 1556.14(1362.19 to 1777.70) |  | 13723.47(10096.03 to 17604.12) | 15581.54(11422.48 to 20091.77) |  | 251.02(178.14 to 326.93) | 271.63(192.38 to 354.09) |
| **2019** | 1262.75(1111.81 to 1439.88) | 1481.57(1291.31 to 1696.41) |  | 12848.13(9413.32 to 16560.04) | 14573.47(10694.68 to 18811.11) |  | 235.49(166.84 to 307.32) | 254.85(180.48 to 332.96) |

| **S10** | | | | | | | | | | | | | | | | | | |
| --- | --- | --- | --- | --- | --- | --- | --- | --- | --- | --- | --- | --- | --- | --- | --- | --- | --- | --- |
| **Group** | **Incidence** | | | | |  | **DALYs (Disability-Adjusted Life Years)** | | | | |  | **Deaths** | | | | | |
|  | **High SDI** | **High-middle SDI** | **Middle SDI** | **Low-middle SDI** | **Low SDI** |  | **High SDI** | **High-middle SDI** | **Middle SDI** | **Low-middle SDI** | **Low SDI** |  | **High SDI** | **High-middle SDI** | **Middle SDI** | **Low-middle SDI** | **Low SDI** |  |
| **1990** | 2139.07(1881.26 to 2418.93) | 7985.21(6993.99 to 9064.27) | 19155.87(16843.03 to 21797.68) | 34132.08(29255.79 to 39708.31) | 36392.93(32074.55 to 41365.24) |  | 8403.17(7868.84 to 9020.57) | 40836.63(37875.00 to 43881.87) | 132280.50(122574.93 to 140921.25) | 356691.17(324158.80 to 391581.31) | 513857.49(455664.27 to 575339.15) |  | 275.87(259.76 to 292.65) | 1032.12(954.95 to 1108.23) | 3914.63(3598.02 to 4288.50) | 10385.98(9318.49 to 11509.52) | 14944.48(13130.76 to 17079.58) |  |
| **1991** | 2059.65(1813.82 to 2327.46) | 7792.68(6829.26 to 8881.75) | 17324.38(15232.42 to 19752.70) | 32780.30(28112.28 to 38122.15) | 33582.83(29622.21 to 38195.69) |  | 7719.43(7233.52 to 8296.36) | 39385.87(36205.04 to 42162.68) | 126309.48(117338.80 to 135624.29) | 350729.08(319836.79 to 384790.07) | 510241.35(452140.55 to 572662.15) |  | 256.05(240.01 to 270.68) | 990.41(896.31 to 1066.82) | 3749.79(3440.95 to 4109.29) | 10237.23(9241.85 to 11383.62) | 14866.00(13010.01 to 17050.23) |  |
| **1992** | 1981.12(1742.86 to 2241.88) | 7665.10(6709.13 to 8756.48) | 16339.67(14384.21 to 18639.73) | 31783.42(27243.52 to 36927.34) | 32986.29(29134.25 to 37434.09) |  | 7105.55(6632.97 to 7597.35) | 38657.68(35466.34 to 41639.10) | 121776.16(112581.96 to 130572.04) | 346497.07(313220.57 to 380959.07) | 505924.17(446341.28 to 565156.12) |  | 239.01(223.90 to 252.48) | 969.40(881.69 to 1045.92) | 3627.41(3303.38 to 3944.19) | 10152.40(9047.26 to 11293.77) | 14764.20(12865.39 to 16848.24) |  |
| **1993** | 1905.54(1680.81 to 2159.05) | 7551.42(6594.97 to 8633.37) | 15432.46(13605.99 to 17627.99) | 30898.07(26563.04 to 35910.38) | 32454.22(28674.25 to 36801.37) |  | 6627.50(6198.12 to 7092.52) | 37990.27(34702.02 to 40874.93) | 113993.73(105327.17 to 122420.72) | 326687.00(297099.00 to 357084.04) | 495596.13(440551.88 to 551811.20) |  | 225.77(210.75 to 238.52) | 949.28(861.58 to 1020.03) | 3410.40(3122.62 to 3721.52) | 9582.81(8662.75 to 10586.97) | 14476.50(12649.08 to 16596.82) |  |
| **1994** | 1836.54(1617.97 to 2079.95) | 7456.35(6486.49 to 8519.54) | 14758.77(13015.21 to 16839.93) | 30155.99(25954.50 to 35042.87) | 32004.44(28250.05 to 36272.43) |  | 6241.22(5843.23 to 6682.16) | 37599.47(34141.80 to 40465.70) | 107676.70(99474.01 to 115414.05) | 309824.03(282343.55 to 337740.24) | 486920.12(433567.37 to 545320.94) |  | 214.66(200.17 to 226.98) | 933.49(838.68 to 1008.06) | 3222.44(2952.84 to 3519.27) | 9064.81(8169.74 to 9982.27) | 14218.29(12532.36 to 16248.06) |  |
| **1995** | 1776.24(1561.06 to 2012.51) | 7386.57(6416.05 to 8477.61) | 14307.12(12620.10 to 16307.61) | 29588.22(25503.81 to 34370.23) | 31635.39(27888.14 to 35835.87) |  | 5869.36(5472.84 to 6297.77) | 36415.75(32509.54 to 39520.81) | 101878.45(93884.16 to 109637.46) | 296728.23(268706.41 to 323166.14) | 478121.09(423850.70 to 535607.35) |  | 204.85(190.32 to 216.06) | 897.49(800.01 to 977.25) | 3047.02(2775.84 to 3327.34) | 8669.58(7781.32 to 9538.70) | 13967.43(12264.04 to 15995.37) |  |
| **1996** | 1715.65(1514.02 to 1948.14) | 7328.71(6381.63 to 8416.43) | 13983.29(12342.83 to 15900.16) | 29159.30(25167.79 to 33847.78) | 31318.03(27591.71 to 35464.97) |  | 5456.48(5061.23 to 5842.65) | 34347.26(30658.58 to 37299.94) | 96449.48(88634.29 to 103667.80) | 285698.79(256588.24 to 310366.30) | 465899.13(414492.19 to 523261.94) |  | 192.63(178.62 to 203.46) | 839.35(736.93 to 915.96) | 2882.93(2619.08 to 3132.75) | 8351.38(7508.28 to 9186.58) | 13626.87(11990.84 to 15588.43) |  |
| **1997** | 1648.52(1451.91 to 1873.79) | 7267.84(6341.49 to 8336.22) | 13705.53(12079.58 to 15568.70) | 28792.52(24866.65 to 33398.04) | 30992.53(27294.61 to 35152.02) |  | 5099.97(4716.45 to 5484.15) | 32897.17(29030.92 to 35990.86) | 92682.13(84939.68 to 99771.74) | 281731.15(251249.58 to 305994.85) | 452908.32(399830.12 to 509108.62) |  | 181.93(167.77 to 191.96) | 799.31(695.71 to 876.28) | 2766.23(2506.38 to 3011.16) | 8261.90(7311.68 to 8991.16) | 13282.46(11690.13 to 15150.35) |  |
| **1998** | 1582.12(1388.86 to 1799.38) | 7208.97(6285.67 to 8258.63) | 13450.29(11861.35 to 15261.60) | 28456.82(24589.47 to 32980.11) | 30664.74(26990.95 to 34830.53) |  | 4835.58(4465.87 to 5193.38) | 31722.68(27624.40 to 34895.90) | 90246.33(82318.07 to 97648.11) | 272942.86(243979.16 to 296886.56) | 440472.72(388226.32 to 495223.81) |  | 174.55(161.23 to 184.40) | 766.05(660.51 to 844.63) | 2689.25(2442.28 to 2938.46) | 8026.09(7182.17 to 8749.71) | 12930.56(11335.68 to 14808.36) |  |
| **1999** | 1525.84(1338.59 to 1736.43) | 7152.78(6196.23 to 8212.25) | 13221.23(11636.10 to 14975.60) | 28121.61(24228.40 to 32558.02) | 30335.03(26730.49 to 34489.76) |  | 4571.92(4220.00 to 4901.89) | 31571.50(27637.54 to 34842.82) | 87606.74(79860.83 to 94450.52) | 263625.46(236496.64 to 285477.83) | 426825.51(376581.47 to 477947.58) |  | 167.12(153.46 to 176.18) | 758.49(649.39 to 842.82) | 2599.97(2353.31 to 2820.95) | 7710.50(6850.70 to 8423.92) | 12505.30(10969.11 to 14282.21) |  |
| **2000** | 1488.51(1301.33 to 1695.69) | 7101.43(6157.33 to 8154.99) | 13022.08(11453.19 to 14759.57) | 27753.76(23958.42 to 32159.98) | 29997.39(26457.22 to 34176.37) |  | 4292.74(3965.95 to 4630.97) | 30895.51(26895.95 to 34220.70) | 85019.41(76853.93 to 91665.97) | 254194.11(229260.00 to 275619.50) | 416084.49(366709.16 to 469468.95) |  | 156.99(143.86 to 166.00) | 741.18(636.94 to 821.85) | 2518.26(2260.04 to 2736.16) | 7435.47(6669.52 to 8100.90) | 12215.12(10681.95 to 13955.35) |  |
| **2001** | 1465.20(1284.04 to 1671.41) | 7019.62(6094.14 to 8054.26) | 12792.41(11240.58 to 14512.78) | 27281.38(23526.75 to 31565.81) | 29609.00(26128.37 to 33702.36) |  | 4007.45(3692.29 to 4320.70) | 29489.89(25448.89 to 32666.26) | 80825.84(73505.32 to 87133.45) | 241133.11(217190.17 to 261202.67) | 400624.58(353247.17 to 450332.43) |  | 146.22(133.49 to 154.91) | 706.16(602.36 to 783.06) | 2391.43(2161.14 to 2600.29) | 7071.72(6335.85 to 7662.88) | 11770.39(10353.85 to 13461.44) |  |
| **2002** | 1445.08(1265.64 to 1652.06) | 6884.52(5990.34 to 7887.02) | 12521.22(11026.78 to 14202.74) | 26692.99(23024.15 to 30820.01) | 29143.28(25704.36 to 33172.41) |  | 3748.48(3455.06 to 4050.31) | 27987.87(24010.72 to 31084.23) | 77568.43(70162.31 to 83826.22) | 228362.77(206348.91 to 246827.83) | 382640.11(337880.00 to 431299.10) |  | 136.58(124.71 to 144.61) | 671.66(572.29 to 751.06) | 2295.55(2066.36 to 2486.49) | 6716.46(6023.12 to 7303.91) | 11266.93(9963.90 to 12901.70) |  |
| **2003** | 1426.37(1247.97 to 1633.64) | 6722.96(5843.71 to 7691.77) | 12210.21(10748.40 to 13837.16) | 26047.24(22562.07 to 30037.35) | 28641.97(25263.72 to 32578.60) |  | 3545.91(3256.90 to 3837.04) | 26397.86(22534.61 to 29535.49) | 74399.06(67276.45 to 80259.99) | 215288.43(194597.71 to 233806.14) | 366566.39(324141.67 to 413613.59) |  | 128.45(116.63 to 136.40) | 633.83(538.42 to 707.56) | 2194.98(1983.79 to 2372.52) | 6334.86(5716.60 to 6878.22) | 10807.17(9546.24 to 12413.39) |  |
| **2004** | 1408.57(1230.64 to 1614.16) | 6559.95(5724.18 to 7494.68) | 11918.46(10507.13 to 13511.36) | 25402.49(22025.16 to 29314.36) | 28134.82(24821.38 to 32092.89) |  | 3336.59(3053.32 to 3620.05) | 24750.00(21149.60 to 27716.33) | 71034.22(64616.29 to 76642.19) | 201135.84(183043.55 to 217955.94) | 348275.69(308328.47 to 394338.33) |  | 119.86(108.87 to 127.35) | 590.58(500.83 to 661.22) | 2085.52(1888.80 to 2260.19) | 5860.69(5313.84 to 6345.33) | 10266.40(9167.22 to 11797.98) |  |
| **2005** | 1390.11(1214.04 to 1596.01) | 6419.93(5606.96 to 7340.95) | 11669.90(10290.48 to 13222.57) | 24816.53(21507.48 to 28609.70) | 27655.85(24363.82 to 31697.72) |  | 3227.92(2959.87 to 3496.93) | 23696.74(19940.48 to 26744.86) | 68069.26(61863.98 to 73408.45) | 193204.77(175469.95 to 209143.26) | 331277.58(293292.04 to 375694.00) |  | 115.24(104.72 to 122.71) | 562.63(476.03 to 635.14) | 1992.73(1807.59 to 2150.99) | 5624.66(5086.43 to 6090.15) | 9786.19(8718.71 to 11258.70) |  |
| **2006** | 1365.87(1192.34 to 1571.51) | 6270.32(5467.75 to 7178.78) | 11450.17(10123.36 to 12982.62) | 24193.54(20981.16 to 27937.41) | 27138.91(23974.80 to 31062.66) |  | 3084.34(2840.51 to 3343.56) | 21608.86(18255.35 to 24404.02) | 64887.04(59323.25 to 69971.22) | 184779.12(168201.32 to 200422.55) | 317346.19(282498.45 to 359275.11) |  | 109.26(99.58 to 116.52) | 508.62(429.94 to 573.88) | 1896.85(1730.97 to 2039.55) | 5376.72(4886.38 to 5832.85) | 9397.69(8365.51 to 10684.09) |  |
| **2007** | 1334.60(1164.17 to 1537.73) | 6073.25(5286.25 to 6956.57) | 11203.35(9884.02 to 12710.86) | 23455.61(20317.36 to 27101.72) | 26516.45(23424.87 to 30366.54) |  | 2953.47(2714.67 to 3198.21) | 20014.36(17033.90 to 22659.95) | 61509.57(56015.61 to 66432.99) | 176732.40(160951.31 to 191684.13) | 306349.66(273809.48 to 345161.00) |  | 103.43(93.98 to 110.41) | 468.75(395.57 to 529.66) | 1796.25(1638.95 to 1937.22) | 5134.99(4672.83 to 5582.65) | 9088.86(8127.79 to 10325.19) |  |
| **2008** | 1299.92(1132.91 to 1499.27) | 5854.39(5092.22 to 6706.62) | 10920.72(9625.18 to 12389.44) | 22684.02(19683.08 to 26196.45) | 26011.16(22980.36 to 29780.48) |  | 2830.37(2600.86 to 3074.05) | 18748.48(15926.70 to 21150.50) | 58577.09(53461.94 to 63276.54) | 171095.94(154839.33 to 185972.38) | 292881.93(260178.98 to 330096.67) |  | 98.10(89.06 to 104.66) | 438.84(371.47 to 494.09) | 1711.45(1557.56 to 1844.74) | 4955.76(4477.50 to 5413.21) | 8712.72(7771.33 to 9950.38) |  |
| **2009** | 1266.33(1101.26 to 1462.04) | 5637.34(4898.00 to 6466.07) | 10650.08(9395.59 to 12092.89) | 21962.81(19027.17 to 25374.66) | 25370.13(22390.76 to 29051.40) |  | 2707.29(2485.02 to 2947.14) | 17109.45(14651.26 to 19354.50) | 55742.31(51008.50 to 60189.41) | 161468.19(145525.01 to 176008.65) | 279827.76(248167.26 to 315979.37) |  | 92.89(84.09 to 98.98) | 399.54(342.76 to 451.18) | 1627.87(1492.64 to 1749.93) | 4636.26(4174.63 to 5060.50) | 8328.75(7406.03 to 9432.97) |  |
| **2010** | 1237.15(1075.82 to 1430.51) | 5443.16(4724.89 to 6257.72) | 10418.06(9177.69 to 11812.62) | 21374.53(18518.75 to 24689.80) | 24808.73(21853.18 to 28451.07) |  | 2595.57(2377.23 to 2842.07) | 15851.37(13607.27 to 17893.57) | 52677.43(47950.82 to 57020.77) | 153549.83(136605.00 to 167728.81) | 264932.69(233432.70 to 298277.56) |  | 88.91(80.37 to 94.82) | 368.25(315.75 to 414.52) | 1539.46(1403.94 to 1665.80) | 4374.99(3890.43 to 4783.59) | 7875.03(6967.42 to 8939.66) |  |
| **2011** | 1207.71(1050.71 to 1393.14) | 5228.25(4523.56 to 6006.75) | 10200.82(8977.58 to 11574.20) | 20857.05(18065.91 to 24055.83) | 24236.56(21395.40 to 27737.45) |  | 2469.05(2264.87 to 2701.65) | 14649.96(12579.19 to 16619.33) | 49509.13(45311.13 to 53629.87) | 146540.86(130296.78 to 160027.07) | 253020.86(223166.88 to 285289.42) |  | 85.33(76.77 to 91.23) | 340.23(292.19 to 384.12) | 1447.31(1323.64 to 1563.24) | 4157.81(3690.76 to 4565.41) | 7527.65(6625.07 to 8530.10) |  |
| **2012** | 1172.05(1018.93 to 1350.52) | 4958.98(4303.94 to 5693.70) | 9966.79(8759.03 to 11274.24) | 20318.25(17639.91 to 23393.73) | 23563.76(20760.12 to 26872.37) |  | 2338.77(2134.00 to 2567.53) | 13742.35(11804.20 to 15533.66) | 46885.28(42571.93 to 50735.21) | 142015.84(124442.38 to 155428.79) | 244929.38(215000.79 to 274863.00) |  | 81.39(73.11 to 87.03) | 319.99(272.71 to 359.63) | 1367.23(1251.40 to 1480.96) | 4030.50(3517.11 to 4429.26) | 7303.81(6438.20 to 8225.47) |  |
| **2013** | 1134.27(987.74 to 1304.57) | 4681.45(4070.57 to 5360.98) | 9736.25(8561.05 to 11012.02) | 19790.57(17206.22 to 22736.31) | 22868.46(20159.01 to 25888.77) |  | 2224.56(2022.28 to 2457.08) | 12926.28(11059.15 to 14517.22) | 44834.42(41011.27 to 48352.17) | 137148.26(118771.52 to 152129.06) | 236851.03(210419.88 to 267333.24) |  | 77.45(69.51 to 83.09) | 301.95(256.57 to 339.57) | 1310.88(1193.16 to 1412.40) | 3935.04(3387.97 to 4356.86) | 7081.37(6264.96 to 7980.29) |  |
| **2014** | 1099.82(957.88 to 1264.04) | 4439.59(3863.11 to 5097.18) | 9532.61(8409.11 to 10774.20) | 19307.89(16733.37 to 22233.60) | 22236.05(19575.28 to 25054.01) |  | 2112.77(1910.92 to 2345.86) | 12120.77(10403.19 to 13639.82) | 42958.04(38867.79 to 46480.59) | 132286.27(114402.05 to 145833.40) | 229177.59(202674.17 to 257756.38) |  | 73.68(66.05 to 79.37) | 283.86(241.94 to 317.97) | 1258.35(1147.89 to 1354.78) | 3835.65(3287.18 to 4255.16) | 6855.19(6081.33 to 7703.04) |  |
| **2015** | 1073.62(934.91 to 1234.61) | 4272.93(3714.08 to 4907.07) | 9378.53(8277.91 to 10577.27) | 18907.10(16305.37 to 21847.72) | 21746.36(19158.97 to 24514.82) |  | 2024.38(1825.78 to 2255.43) | 11356.37(9718.60 to 12832.73) | 40783.81(37015.35 to 44312.14) | 126104.58(107256.43 to 140477.98) | 219843.19(193019.48 to 249128.68) |  | 70.97(63.50 to 76.90) | 267.00(228.75 to 301.01) | 1190.71(1087.48 to 1286.71) | 3639.69(3096.51 to 4077.39) | 6570.35(5790.00 to 7447.20) |  |
| **2016** | 1053.21(918.73 to 1207.34) | 4165.18(3619.14 to 4770.60) | 9268.24(8208.48 to 10435.89) | 18450.15(15884.74 to 21254.57) | 21304.60(18864.09 to 24047.88) |  | 1958.01(1757.03 to 2192.00) | 10774.06(9184.74 to 12207.36) | 38991.02(35181.73 to 42333.43) | 121236.35(102471.07 to 135941.08) | 209707.02(182761.87 to 238864.01) |  | 69.20(61.55 to 75.15) | 256.44(218.22 to 289.61) | 1140.61(1034.68 to 1238.50) | 3488.05(2931.73 to 3943.75) | 6262.92(5512.93 to 7097.14) |  |
| **2017** | 1036.60(903.72 to 1186.35) | 4092.15(3550.36 to 4678.27) | 9192.45(8116.46 to 10324.66) | 18062.91(15570.33 to 20855.81) | 20959.19(18524.58 to 23671.50) |  | 1912.17(1716.74 to 2146.77) | 10195.55(8688.46 to 11584.54) | 37226.22(33616.77 to 40632.37) | 116805.64(98160.50 to 132428.44) | 200145.62(173685.73 to 229472.15) |  | 68.30(60.72 to 74.72) | 242.88(205.61 to 275.96) | 1084.82(976.03 to 1186.15) | 3368.42(2811.12 to 3843.00) | 5992.06(5240.74 to 6901.05) |  |
| **2018** | 1014.56(882.18 to 1167.27) | 4050.53(3501.66 to 4638.00) | 9113.67(8070.38 to 10274.35) | 17795.52(15394.00 to 20473.89) | 20645.29(18245.60 to 23301.80) |  | 1882.63(1674.74 to 2128.39) | 9900.69(8301.83 to 11369.91) | 35562.66(31668.55 to 39118.53) | 110825.08(91895.69 to 126666.98) | 190857.66(165843.40 to 219150.23) |  | 67.62(59.94 to 74.42) | 234.09(196.17 to 267.79) | 1027.54(919.48 to 1134.17) | 3197.80(2647.01 to 3715.49) | 5724.63(4991.75 to 6596.77) |  |
| **2019** | 982.37(851.39 to 1134.43) | 4024.96(3443.85 to 4629.60) | 9015.10(7936.89 to 10194.21) | 17500.17(14921.31 to 20233.35) | 20228.73(17704.87 to 22958.49) |  | 1864.03(1657.62 to 2112.64) | 9656.43(8011.62 to 11122.82) | 34089.01(30391.15 to 37561.25) | 105034.56(87174.67 to 121173.83) | 181931.29(157033.17 to 209942.48) |  | 67.26(59.26 to 74.32) | 226.85(186.85 to 259.47) | 976.87(877.62 to 1087.63) | 3027.31(2490.85 to 3494.54) | 5472.99(4760.87 to 6280.80) |  |

| **S11** | | | | | | | | | | | | | | | | | |
| --- | --- | --- | --- | --- | --- | --- | --- | --- | --- | --- | --- | --- | --- | --- | --- | --- | --- |
| **Group** | **Incidence** | | | | |  | **DALYs (Disability-Adjusted Life Years)** | | | | |  | **Deaths** | | | | |
|  | **High SDI** | **High-middle SDI** | **Middle SDI** | **Low-middle SDI** | **Low SDI** |  | **High SDI** | **High-middle SDI** | **Middle SDI** | **Low-middle SDI** | **Low SDI** |  | **High SDI** | **High-middle SDI** | **Middle SDI** | **Low-middle SDI** | **Low SDI** |
| **1990** | 119.84(101.64 to 142.29) | 208.81(178.90 to 243.52) | 532.69(467.21 to 606.41) | 1280.36(1109.94 to 1470.12) | 5715.64(5102.54 to 6372.34) |  | 1453.43(976.18 to 2123.65) | 1746.52(1267.93 to 2287.39) | 2380.72(1907.40 to 3024.50) | 12414.01(9087.44 to 17556.36) | 101519.93(74391.96 to 132759.62) |  | 27.69(17.98 to 40.97) | 30.44(21.82 to 40.33) | 38.06(28.59 to 50.78) | 205.01(143.86 to 302.41) | 1853.15(1310.37 to 2466.89) |
| **1991** | 126.27(107.00 to 148.59) | 258.65(222.31 to 300.71) | 692.00(614.12 to 776.62) | 1518.23(1306.28 to 1749.09) | 5870.22(5225.42 to 6563.99) |  | 1536.62(1020.78 to 2283.29) | 2018.41(1454.72 to 2633.41) | 3449.70(2746.17 to 4466.05) | 16703.68(12319.56 to 23146.69) | 120197.49(88624.53 to 155820.70) |  | 29.18(18.74 to 44.03) | 34.91(24.71 to 46.28) | 55.49(41.47 to 75.43) | 280.00(197.22 to 400.37) | 2209.25(1580.17 to 2913.66) |
| **1992** | 128.99(109.49 to 151.62) | 304.89(261.86 to 354.59) | 876.08(777.28 to 981.30) | 1782.71(1525.64 to 2061.25) | 6468.63(5735.19 to 7259.56) |  | 1558.78(1027.10 to 2305.81) | 2413.80(1752.30 to 3150.81) | 4907.71(3835.06 to 6413.37) | 21734.09(16133.34 to 30181.04) | 138838.37(102604.00 to 179807.09) |  | 29.65(18.94 to 44.73) | 41.60(29.49 to 55.30) | 80.27(59.73 to 109.29) | 369.92(264.45 to 522.60) | 2571.61(1859.54 to 3372.85) |
| **1993** | 129.61(110.46 to 152.19) | 346.59(296.93 to 403.93) | 1045.39(926.90 to 1171.20) | 2034.45(1736.26 to 2358.83) | 6970.05(6162.40 to 7827.74) |  | 1589.45(1038.11 to 2359.92) | 2901.12(2106.87 to 3834.77) | 6705.55(5179.52 to 8639.12) | 27342.46(20462.67 to 37639.13) | 156965.93(116504.54 to 203697.41) |  | 30.30(19.23 to 45.69) | 50.11(35.66 to 67.17) | 111.60(83.22 to 150.10) | 472.43(339.83 to 668.80) | 2929.23(2125.54 to 3837.41) |
| **1994** | 128.55(109.76 to 150.58) | 382.50(326.76 to 446.50) | 1212.93(1069.56 to 1371.63) | 2267.10(1929.06 to 2628.10) | 7391.86(6518.14 to 8311.70) |  | 1579.18(1031.01 to 2342.30) | 3421.96(2491.07 to 4531.07) | 8737.03(6764.93 to 11204.50) | 33153.00(24862.38 to 45007.70) | 173880.31(130130.65 to 226717.19) |  | 30.31(19.26 to 45.58) | 59.41(42.26 to 80.24) | 147.36(110.30 to 197.37) | 580.76(422.08 to 806.79) | 3267.34(2379.40 to 4279.73) |
| **1995** | 126.10(107.91 to 147.43) | 410.90(349.31 to 479.27) | 1358.91(1191.22 to 1548.46) | 2473.23(2108.80 to 2869.80) | 7723.46(6812.91 to 8700.93) |  | 1547.66(1009.86 to 2291.49) | 3882.75(2791.62 to 5184.40) | 11003.75(8373.74 to 14238.26) | 38957.70(29154.87 to 52677.67) | 188200.35(141512.02 to 246120.84) |  | 29.87(18.87 to 44.78) | 67.43(47.31 to 92.00) | 187.56(138.80 to 248.94) | 691.07(503.84 to 957.46) | 3559.62(2613.03 to 4676.38) |
| **1996** | 118.41(101.43 to 138.39) | 435.31(369.00 to 508.96) | 1503.23(1316.88 to 1710.54) | 2683.72(2288.64 to 3111.99) | 8008.60(7054.94 to 9012.95) |  | 1308.27(864.02 to 1924.33) | 4189.33(2950.39 to 5665.76) | 13452.22(9887.50 to 17440.86) | 44466.73(33195.91 to 60316.08) | 198959.16(148981.21 to 258341.51) |  | 25.25(16.10 to 37.87) | 72.51(49.69 to 99.34) | 231.28(165.00 to 306.83) | 797.32(583.98 to 1103.64) | 3786.03(2805.22 to 4931.89) |
| **1997** | 103.99(89.14 to 121.27) | 458.03(386.43 to 537.12) | 1669.09(1457.89 to 1901.45) | 2914.32(2478.75 to 3370.02) | 8270.14(7280.92 to 9318.85) |  | 899.40(603.29 to 1233.97) | 4351.66(2989.83 to 5971.45) | 16465.86(11821.73 to 21466.87) | 50103.40(37185.10 to 68110.86) | 206940.29(154339.22 to 267838.66) |  | 17.31(11.22 to 24.25) | 74.62(50.03 to 103.85) | 285.41(200.53 to 379.31) | 906.61(663.55 to 1248.69) | 3959.36(2929.15 to 5153.55) |
| **1998** | 87.21(75.09 to 101.71) | 478.31(400.39 to 560.80) | 1836.01(1602.83 to 2091.30) | 3135.35(2668.94 to 3620.01) | 8481.54(7448.87 to 9570.62) |  | 758.97(517.32 to 1014.54) | 4421.74(2913.40 to 6166.17) | 19857.54(13909.07 to 26003.96) | 55971.85(41594.06 to 74887.08) | 212093.44(158677.57 to 275204.57) |  | 14.76(9.71 to 20.03) | 75.53(48.67 to 107.75) | 346.65(236.39 to 465.53) | 1020.15(740.62 to 1381.99) | 4074.72(3010.95 to 5294.17) |
| **1999** | 72.40(62.67 to 84.30) | 494.80(411.57 to 582.62) | 1990.28(1733.41 to 2272.59) | 3317.29(2818.98 to 3826.43) | 8626.60(7550.59 to 9745.03) |  | 704.93(478.55 to 947.52) | 4649.43(3043.97 to 6707.02) | 22781.99(15487.39 to 30097.98) | 61140.38(45095.24 to 81736.86) | 214819.28(159621.96 to 277143.96) |  | 13.90(9.09 to 18.99) | 79.89(51.18 to 117.18) | 401.16(265.13 to 541.42) | 1120.78(813.04 to 1515.26) | 4141.42(3039.00 to 5342.34) |
| **2000** | 63.87(55.46 to 74.34) | 505.45(420.37 to 594.41) | 2115.70(1839.56 to 2413.56) | 3430.48(2916.64 to 3955.13) | 8682.90(7593.95 to 9804.02) |  | 632.74(426.25 to 865.15) | 4701.43(3063.78 to 6865.65) | 25661.37(17286.47 to 34115.86) | 65275.92(47829.97 to 86648.95) | 216120.29(159239.34 to 277657.58) |  | 12.60(8.21 to 17.50) | 81.30(51.57 to 120.37) | 456.51(297.84 to 618.59) | 1203.31(870.54 to 1608.99) | 4180.34(3050.74 to 5376.95) |
| **2001** | 60.75(52.78 to 70.71) | 510.57(426.93 to 601.95) | 2228.24(1930.43 to 2540.50) | 3492.05(2976.58 to 4032.17) | 8634.07(7533.58 to 9753.20) |  | 593.91(400.36 to 813.73) | 4601.31(3027.18 to 6787.70) | 27782.51(18194.63 to 37508.84) | 67546.37(49320.44 to 88972.70) | 213358.89(156386.25 to 273648.34) |  | 11.88(7.76 to 16.60) | 79.95(51.84 to 120.19) | 498.28(316.90 to 681.06) | 1250.89(897.88 to 1669.01) | 4138.32(3015.23 to 5298.48) |
| **2002** | 58.91(51.17 to 68.54) | 511.31(430.15 to 604.09) | 2345.62(2027.98 to 2676.83) | 3535.31(3008.62 to 4087.15) | 8498.89(7400.48 to 9614.33) |  | 562.55(378.68 to 779.53) | 4399.38(2899.65 to 6517.34) | 30172.37(19629.90 to 40589.66) | 68719.48(50069.40 to 89080.25) | 207068.55(149423.27 to 266111.35) |  | 11.29(7.37 to 15.96) | 76.70(49.79 to 115.57) | 545.05(342.94 to 740.12) | 1277.57(907.00 to 1677.16) | 4024.83(2881.92 to 5148.13) |
| **2003** | 57.80(50.20 to 67.25) | 508.21(430.15 to 601.30) | 2444.16(2114.15 to 2795.37) | 3555.83(3017.90 to 4124.19) | 8305.54(7238.17 to 9412.88) |  | 534.93(357.99 to 749.54) | 4178.35(2746.82 to 6199.42) | 32358.88(20998.10 to 43160.44) | 68650.01(50268.05 to 88700.75) | 199785.80(143495.72 to 256743.32) |  | 10.74(6.98 to 15.24) | 73.17(47.12 to 110.74) | 588.53(371.15 to 794.40) | 1279.77(912.50 to 1658.29) | 3891.74(2743.34 to 4979.32) |
| **2004** | 56.90(49.38 to 66.19) | 501.95(425.21 to 593.02) | 2511.85(2168.86 to 2872.63) | 3551.02(3013.35 to 4117.91) | 8072.55(7015.17 to 9159.03) |  | 486.87(326.44 to 698.57) | 3872.98(2545.44 to 5760.46) | 33399.77(21555.78 to 44335.27) | 66945.00(49166.24 to 85895.86) | 190935.74(134401.51 to 243667.90) |  | 9.77(6.30 to 14.33) | 67.89(43.33 to 103.13) | 610.07(385.49 to 819.23) | 1248.54(900.85 to 1603.17) | 3725.50(2567.35 to 4767.97) |
| **2005** | 55.64(48.29 to 64.71) | 493.02(419.32 to 579.62) | 2530.79(2182.93 to 2891.99) | 3518.91(2984.70 to 4095.93) | 7822.29(6797.34 to 8871.29) |  | 457.21(306.63 to 657.77) | 3717.75(2453.37 to 5538.52) | 33418.01(21575.40 to 44092.41) | 63819.71(47196.29 to 80963.03) | 178137.65(123218.19 to 226874.34) |  | 9.22(5.95 to 13.59) | 65.17(41.52 to 99.13) | 612.54(386.44 to 816.25) | 1188.54(868.04 to 1516.67) | 3475.84(2353.02 to 4428.52) |
| **2006** | 53.67(46.63 to 62.38) | 477.39(405.44 to 561.69) | 2500.65(2158.70 to 2860.55) | 3428.25(2919.97 to 3974.47) | 7516.46(6518.89 to 8524.22) |  | 424.24(285.67 to 616.28) | 3435.44(2285.72 to 5085.96) | 32487.60(21146.84 to 42390.19) | 59779.15(44706.56 to 75327.67) | 162938.86(112921.42 to 207100.29) |  | 8.60(5.57 to 12.83) | 60.05(38.46 to 90.42) | 597.29(381.55 to 787.51) | 1110.31(821.16 to 1407.15) | 3176.40(2154.14 to 4026.64) |
| **2007** | 51.28(44.57 to 59.53) | 453.40(385.52 to 531.05) | 2436.97(2110.51 to 2783.67) | 3270.68(2789.15 to 3785.09) | 7128.63(6181.82 to 8086.46) |  | 400.59(270.50 to 581.27) | 3214.33(2148.95 to 4724.74) | 30439.76(19734.19 to 39684.65) | 54603.76(41035.83 to 68808.54) | 147439.42(103442.43 to 186131.54) |  | 8.13(5.29 to 12.11) | 56.20(36.05 to 83.98) | 559.68(353.92 to 739.03) | 1010.35(758.72 to 1276.65) | 2867.23(1978.09 to 3613.63) |
| **2008** | 48.73(42.36 to 56.56) | 425.92(361.55 to 500.44) | 2351.86(2048.08 to 2683.38) | 3078.83(2624.74 to 3565.75) | 6734.57(5843.15 to 7645.55) |  | 372.68(251.62 to 540.35) | 3025.04(2006.99 to 4421.64) | 28039.21(18217.75 to 36599.09) | 49067.30(37375.71 to 61644.62) | 132789.20(94305.52 to 166548.85) |  | 7.57(4.92 to 11.19) | 52.96(33.93 to 79.45) | 514.50(324.10 to 678.16) | 905.96(680.44 to 1137.34) | 2568.99(1784.89 to 3209.86) |
| **2009** | 46.34(40.28 to 53.82) | 399.67(339.00 to 469.47) | 2265.96(1979.16 to 2587.29) | 2886.26(2466.63 to 3348.59) | 6322.99(5494.76 to 7192.92) |  | 344.37(232.48 to 495.19) | 2753.50(1806.94 to 3967.44) | 25303.89(16356.46 to 33278.54) | 43760.88(33550.76 to 54958.30) | 120466.63(85427.01 to 151068.14) |  | 7.02(4.59 to 10.38) | 48.19(30.92 to 71.54) | 464.38(290.08 to 612.52) | 807.53(611.03 to 1015.22) | 2320.32(1617.94 to 2896.47) |
| **2010** | 44.38(38.54 to 51.60) | 378.94(320.77 to 444.98) | 2195.24(1913.72 to 2510.70) | 2726.80(2328.77 to 3162.60) | 5969.11(5181.62 to 6778.15) |  | 322.52(219.08 to 459.20) | 2498.40(1675.26 to 3586.00) | 23040.41(14886.74 to 30570.79) | 38886.99(29755.53 to 48911.76) | 110418.90(78387.23 to 138501.73) |  | 6.60(4.35 to 9.65) | 43.59(27.82 to 64.14) | 422.62(264.49 to 562.10) | 715.83(543.26 to 906.66) | 2127.34(1478.98 to 2661.16) |
| **2011** | 42.45(36.94 to 49.22) | 361.81(307.26 to 424.86) | 2122.92(1849.93 to 2416.41) | 2587.58(2221.39 to 2995.23) | 5642.57(4910.81 to 6397.23) |  | 301.34(205.73 to 429.07) | 2279.85(1536.22 to 3235.81) | 20728.42(13393.63 to 27714.43) | 34635.58(26491.52 to 43712.33) | 101767.47(72296.52 to 127765.16) |  | 6.19(4.07 to 9.01) | 39.84(25.75 to 58.12) | 377.90(231.96 to 508.96) | 637.16(483.31 to 809.63) | 1967.69(1366.49 to 2468.85) |
| **2012** | 40.17(34.92 to 46.39) | 344.26(294.53 to 403.76) | 2029.93(1772.65 to 2308.39) | 2439.07(2101.14 to 2819.80) | 5295.49(4629.89 to 5994.93) |  | 285.43(194.26 to 408.90) | 2139.24(1457.52 to 3018.02) | 18509.45(11982.64 to 24846.50) | 31442.40(24101.92 to 39731.95) | 93232.10(66960.39 to 116631.43) |  | 5.89(3.87 to 8.60) | 37.39(24.37 to 54.15) | 335.48(206.00 to 455.38) | 579.76(439.75 to 735.67) | 1806.91(1266.43 to 2259.23) |
| **2013** | 37.85(32.94 to 43.57) | 327.47(280.74 to 383.88) | 1930.54(1691.11 to 2190.02) | 2290.91(1980.57 to 2643.22) | 4952.36(4325.60 to 5614.17) |  | 271.09(184.06 to 392.63) | 2004.53(1389.27 to 2830.45) | 16765.55(10880.71 to 22479.54) | 28978.57(22052.46 to 36672.95) | 84236.26(61317.52 to 104439.38) |  | 5.65(3.69 to 8.37) | 35.10(23.16 to 50.90) | 303.18(186.81 to 415.35) | 536.39(402.61 to 683.26) | 1633.70(1168.62 to 2022.71) |
| **2014** | 35.83(31.27 to 41.16) | 312.44(266.23 to 365.56) | 1839.53(1610.65 to 2084.55) | 2153.20(1859.14 to 2479.51) | 4639.41(4049.96 to 5268.68) |  | 253.60(172.03 to 363.36) | 1918.92(1328.66 to 2689.91) | 15643.32(10217.00 to 21113.33) | 26971.62(20572.13 to 34202.71) | 75731.17(56461.78 to 92900.61) |  | 5.27(3.43 to 7.72) | 33.64(22.29 to 48.79) | 283.23(174.92 to 389.21) | 501.75(375.28 to 638.74) | 1469.80(1073.66 to 1799.45) |
| **2015** | 34.47(30.08 to 39.70) | 299.91(254.73 to 350.77) | 1772.06(1554.36 to 2004.19) | 2036.13(1763.44 to 2348.45) | 4381.64(3821.64 to 4986.41) |  | 250.35(169.67 to 364.98) | 1825.93(1264.51 to 2549.06) | 14759.10(9635.17 to 20182.21) | 24802.88(18804.23 to 31574.74) | 68592.41(51438.19 to 84001.86) |  | 5.22(3.36 to 7.76) | 32.08(21.15 to 46.42) | 269.50(166.07 to 373.48) | 463.41(344.48 to 590.98) | 1332.27(977.83 to 1632.19) |
| **2016** | 33.69(29.42 to 38.76) | 287.29(246.39 to 333.41) | 1720.84(1511.67 to 1945.35) | 1933.49(1686.96 to 2225.56) | 4174.63(3658.54 to 4746.27) |  | 250.29(169.93 to 363.74) | 1747.78(1218.01 to 2441.93) | 14107.61(9135.05 to 19419.58) | 22600.98(17100.18 to 28653.42) | 62416.19(46663.00 to 76671.51) |  | 5.22(3.38 to 7.76) | 30.76(20.39 to 44.23) | 260.38(159.03 to 367.21) | 423.61(314.32 to 541.49) | 1213.48(895.51 to 1494.18) |
| **2017** | 32.98(28.89 to 37.84) | 276.84(238.58 to 320.08) | 1676.78(1480.95 to 1894.32) | 1844.03(1607.12 to 2122.82) | 3977.51(3493.38 to 4523.01) |  | 245.65(166.60 to 357.59) | 1644.38(1161.05 to 2280.30) | 13276.28(8640.52 to 18449.51) | 20540.95(15574.04 to 26136.91) | 55687.86(42147.21 to 68277.55) |  | 5.13(3.33 to 7.60) | 28.73(19.28 to 41.04) | 246.11(150.30 to 352.53) | 385.72(286.26 to 493.59) | 1084.01(800.35 to 1341.47) |
| **2018** | 31.99(27.94 to 36.84) | 270.11(231.23 to 312.46) | 1635.57(1445.35 to 1848.66) | 1759.68(1530.63 to 2026.75) | 3745.90(3278.48 to 4270.96) |  | 244.36(166.28 to 353.10) | 1591.18(1125.79 to 2172.79) | 12118.45(7945.60 to 16829.09) | 18654.85(14186.13 to 23852.74) | 49485.92(37657.61 to 60742.70) |  | 5.11(3.32 to 7.56) | 27.59(18.56 to 39.02) | 223.96(137.12 to 322.18) | 351.36(260.75 to 452.93) | 965.74(718.26 to 1188.98) |
| **2019** | 30.72(26.67 to 35.58) | 264.39(224.63 to 307.81) | 1590.45(1397.25 to 1801.79) | 1669.93(1450.05 to 1935.40) | 3476.76(3037.45 to 3977.41) |  | 242.14(164.86 to 348.22) | 1556.48(1106.66 to 2114.40) | 11418.31(7498.60 to 15874.34) | 17384.52(13251.51 to 22316.43) | 45495.49(34473.84 to 56412.05) |  | 5.07(3.31 to 7.43) | 26.88(18.23 to 37.91) | 210.40(129.46 to 302.36) | 328.71(243.79 to 424.24) | 891.66(658.69 to 1106.21) |

| **S12** | | | | | | | | | | | |
| --- | --- | --- | --- | --- | --- | --- | --- | --- | --- | --- | --- |
| **Group** | **HIV/AIDS - Drug-susceptible Tuberculosis** | | |  | **HIV/AIDS - Extensively drug-resistant Tuberculosis** | | |  | **HIV/AIDS - Multidrug-resistant Tuberculosis without extensive drug resistance** | | |
|  | **Number of incident cases, 2019** | **Annualised rate of change of age-standardized incidence** | |  | **Number of incident cases, 2019** | **Annualised rate of change of age-standardized incidence** | |  | **Number of incident cases, 2019** | **Annualised rate of change of age-standardized incidence** | |
|  |  | **1990-2010** | **2010-2019** |  |  | **1990-2010** | **2010-2019** |  |  | **1990-2010** | **2010-2019** |
| **Global** | 1370314(1199744 to 1564779) | 118.2(108.2 to 128.0) | -33.8(-36.0 to -31.4) |  | 2104(1506 to 2902) | - | 3.3(-23.0 to 38.9) |  | 59225(41552 to 83609) | 2294.7(1303.9 to 3839.2) | -30.1(-50.3 to -1.2) |
| **High SDI** | 30717(26673 to 35576) | -63.0(-64.9 to -59.8) | -30.8(-32.8 to -28.8) |  | 104(72 to 145) | - | -2.9(-27.6 to 31.6) |  | 862(562 to 1271) | -47.4(-66.7 to -16.8) | -31.6(-51.8 to 0.3) |
| **High-middle SDI** | 264392(224629 to 307813) | 81.5(66.8 to 94.3) | -30.2(-35.4 to -24.8) |  | 5344(3408 to 7769) | - | 11.4(-23.7 to 74.2) |  | 33619(20874 to 52969) | 3131.5(1315.4 to 7375.5) | -34.6(-60.8 to 10.5) |
| **Middle SDI** | 1590454(1397254 to 1801788) | 312.1(289.7 to 336.6) | -27.6(-30.7 to -24.3) |  | 1410(852 to 2320) | - | -1.5(-43.1 to 64.8) |  | 48527(28863 to 81554) | 1778.9(746.8 to 4546.9) | -38.5(-68.5 to 24.4) |
| **Low-middle SDI** | 1669933(1450047 to 1935401) | 113.0(99.3 to 125.9) | -38.8(-41.5 to -35.8) |  | 1764(996 to 2989) | - | -12.4(-49.8 to 50.4) |  | 82872(47291 to 138412) | 3462.9(1102.1 to 10035.2) | -34.9(-61.9 to 11.1) |
| **Low SDI** | 3476761(3037449 to 3977413) | 4.4(-1.3 to 10.3) | -41.8(-44.2 to -39.1) |  | 1239(755 to 1965) | - | 26.2(-19.6 to 103.2) |  | 148378(92047 to 230778) | 1869.1(874.1 to 3831.0) | -18.8(-47.8 to 28.6) |
| **High-income** | 70653(61225 to 82135) | -52.3(-55.5 to -48.7) | -13.8(-18.1 to -9.4) |  | 167(82 to 405) | - | 21.5(-34.0 to 163.1) |  | 1328(650 to 3226) | -29.9(-60.4 to 26.5) | -22.3(-57.8 to 68.3) |
| **High-income North America** | 19686(17004 to 23007) | -78.3(-79.8 to -76.6) | -35.8(-38.0 to -33.5) |  | 41(18 to 84) | - | 5.9(-51.1 to 107.1) |  | 327(147 to 669) | -90.1(-94.4 to -81.2) | -32.2(-68.7 to 32.4) |
| **Australasia** | 14003(12054 to 16406) | -77.0(-78.5 to -75.2) | -21.2(-26.0 to -16.3) |  | 62(27 to 127) | - | 91.7(-32.8 to 426.4) |  | 491(214 to 1009) | -15.7(-71.9 to 207.0) | 22.7(-57.0 to 236.7) |
| **High-income Asia Pacific** | 31845(27226 to 37291) | -6.3(-24.5 to 255.4) | -21.9(-27.0 to -16.6) |  | 66(16 to 226) | - | 6.5(-67.8 to 195.3) |  | 521(124 to 1800) | 168.2(-35.5 to 1959.1) | -31.9(-79.4 to 88.8) |
| **Western Europe** | 34994(30245 to 40746) | -68.5(-69.9 to -67.1) | -37.5(-39.8 to -35.3) |  | 110(71 to 166) | - | 3.1(-29.7 to 47.8) |  | 875(564 to 1318) | -11.8(-50.6 to 62.7) | -34.0(-55.0 to -5.4) |
| **Southern Latin America** | 683877(588869 to 799082) | -16.2(-26.9 to -5.2) | -1.1(-8.2 to 7.3) |  | 1480(326 to 4978) | - | 28.3(-62.1 to 259.5) |  | 11770(2594 to 39537) | 336.4(-1.1 to 2452.4) | -17.9(-75.7 to 130.1) |
| **Central Europe, Eastern Europe, and Central Asia** | 163449(122330 to 209084) | 71.0(43.3 to 98.1) | -34.7(-47.0 to -21.7) |  | 18219(11415 to 26546) | - | 16.0(-22.7 to 85.7) |  | 83150(52093 to 121152) | 5777.6(2278.7 to 14148.0) | -27.9(-52.0 to 15.4) |
| **Eastern Europe** | 280319(203531 to 364223) | 92.1(58.0 to 124.1) | -35.1(-49.3 to -20.3) |  | 33637(20568 to 49714) | - | 18.9(-21.8 to 95.8) |  | 153519(93869 to 226955) | 6041.7(2288.6 to 15730.7) | -26.1(-51.5 to 21.7) |
| **Central Europe** | 29780(25432 to 35007) | 10.6(-3.8 to 45.5) | -22.7(-27.5 to -18.0) |  | 182(91 to 339) | - | 23.8(-56.1 to 226.2) |  | 829(414 to 1545) | 382.7(92.5 to 1094.8) | -23.1(-72.7 to 102.6) |
| **Central Asia** | 59321(46744 to 72970) | 5.0(-6.7 to 17.9) | -27.3(-39.7 to -16.2) |  | 4862(3072 to 6924) | - | -5.1(-37.0 to 37.8) |  | 22189(14021 to 31612) | 24865.1(8739.9 to 78345.0) | -41.1(-60.9 to -14.4) |
| **Latin America and Caribbean** | 389963(333390 to 452797) | -11.7(-16.6 to -5.9) | -17.6(-21.6 to -13.9) |  | 1221(606 to 2410) | - | 49.7(-21.0 to 176.9) |  | 15462(7670 to 30512) | 1134.6(503.0 to 2414.2) | -2.8(-48.7 to 79.9) |
| **Central Latin America** | 205475(178386 to 237484) | -12.2(-18.7 to -5.6) | -11.2(-15.3 to -7.2) |  | 605(262 to 1238) | - | 49.8(-28.2 to 194.9) |  | 7662(3317 to 15677) | 1876.9(642.2 to 5616.6) | -2.7(-53.4 to 91.5) |
| **Andean Latin America** | 431652(365779 to 507366) | -24.3(-31.9 to -15.7) | -29.3(-34.9 to -24.3) |  | 2834(1459 to 5292) | - | -2.4(-45.6 to 66.6) |  | 35884(18485 to 66990) | 665.7(178.3 to 2614.1) | -36.6(-64.7 to 8.2) |
| **Caribbean** | 609473(528049 to 699091) | 12.6(5.0 to 20.5) | -27.0(-31.2 to -22.8) |  | 300(100 to 833) | - | 73.6(-37.2 to 443.6) |  | 3795(1272 to 10540) | 15.5(-69.2 to 359.3) | 12.7(-59.2 to 253.1) |
| **Tropical Latin America** | 525028(439492 to 620785) | -12.6(-18.5 to -5.5) | -14.6(-21.6 to -8.8) |  | 1615(337 to 4378) | - | 87.3(-55.1 to 376.1) |  | 20444(4274 to 55427) | 4211.0(808.5 to 35773.3) | 21.7(-70.8 to 209.1) |
| **Southeast Asia, East Asia, and Oceania** | 934676(818461 to 1061739) | 354.1(331.1 to 377.6) | -11.6(-14.3 to -8.7) |  | 1552(853 to 2999) | - | -6.6(-50.8 to 76.1) |  | 17016(9355 to 32889) | 664.6(192.9 to 2823.8) | -39.5(-68.1 to 13.9) |
| **East Asia** | 133858(112802 to 154249) | 45.5(28.8 to 56.6) | 3.1(-7.9 to 11.2) |  | 642(137 to 1888) | - | 21.1(-69.4 to 224.3) |  | 7037(1507 to 20701) | 159.0(-23.5 to 1046.8) | -21.6(-80.2 to 109.8) |
| **Southeast Asia** | 2621828(2290731 to 2977603) | 391.2(364.6 to 417.7) | -19.7(-22.2 to -16.9) |  | 3531(1920 to 6204) | - | -19.5(-61.4 to 43.9) |  | 38723(21049 to 68041) | 1625.7(465.5 to 7175.4) | -47.9(-75.1 to -6.9) |
| **Oceania** | 1131739(989229 to 1279222) | 1884.6(1786.9 to 1988.7) | -16.8(-23.1 to -11.3) |  | 4852(1773 to 11122) | - | 295.3(-3.5 to 1386.8) |  | 53214(19436 to 121953) | 47499.9(14941.5 to 153016.8) | 155.8(-37.6 to 862.2) |
| **North Africa and Middle East** | 81222(69951 to 94214) | 37.0(26.5 to 49.6) | -20.8(-24.3 to -16.6) |  | 73(39 to 135) | - | 29.0(-34.9 to 164.6) |  | 2062(1106 to 3824) | 1717.6(697.5 to 3958.5) | -15.0(-57.1 to 74.4) |
| **South Asia** | 452390(367514 to 539611) | 729.4(656.7 to 803.0) | -52.6(-58.2 to -48.5) |  | 1036(227 to 2676) | - | -13.2(-75.1 to 129.7) |  | 42255(9271 to 109181) | 68475.2(11859.6 to 659236.8) | -42.6(-83.6 to 51.9) |
| **Sub-Saharan Africa** | 9272978(8075394 to 10609055) | 54.0(45.3 to 62.3) | -40.9(-43.2 to -38.3) |  | 2396(1602 to 3522) | - | 1.7(-32.6 to 61.1) |  | 371376(248338 to 545915) | 2474.2(1204.6 to 4961.7) | -32.3(-55.2 to 7.2) |
| **Southern Sub-Saharan Africa** | 34622889(30342243 to 39355851) | 231.5(204.1 to 259.3) | -39.8(-43.5 to -35.4) |  | 9180(4701 to 17250) | - | -14.3(-60.9 to 95.2) |  | 1422882(728577 to 2674308) | 3619.3(1030.2 to 14305.0) | -43.0(-74.0 to 29.9) |
| **Western Sub-Saharan Africa** | 4109920(3542616 to 4787274) | 59.2(47.4 to 72.5) | -33.9(-37.6 to -30.4) |  | 1101(483 to 2297) | - | -0.8(-52.1 to 108.6) |  | 170610(74917 to 356008) | 1570.0(662.0 to 3934.3) | -34.0(-68.1 to 38.8) |
| **Eastern Sub-Saharan Africa** | 9994133(8649686 to 11580732) | 7.5(0.4 to 15.2) | -41.3(-43.8 to -38.5) |  | 2593(1507 to 4279) | - | 35.1(-18.5 to 115.6) |  | 401921(233526 to 663221) | 3244.1(1367.6 to 8537.7) | -10.2(-45.8 to 43.4) |
| **Central Sub-Saharan Africa** | 4230570(3685727 to 4812765) | 34.4(26.0 to 41.4) | -47.1(-50.2 to -43.8) |  | 785(280 to 1915) | - | 3.7(-59.5 to 165.3) |  | 121695(43336 to 296676) | 815.7(72.7 to 4455.0) | -31.0(-73.1 to 76.4) |

| **S13** | | | | | | | | | | | |
| --- | --- | --- | --- | --- | --- | --- | --- | --- | --- | --- | --- |
| **Group** | **HIV/AIDS - Drug-susceptible Tuberculosis** | | |  | **HIV/AIDS - Extensively drug-resistant Tuberculosis** | | |  | **HIV/AIDS - Multidrug-resistant Tuberculosis without extensive drug resistance** | | |
|  | **Number of DALYs, 2019** | **Annualised rate of change of age-standardized DALYs** | |  | **Number of DALYs, 2019** | **Annualised rate of change of age-standardized DALYs** | |  | **Number of DALYs, 2019** | **Annualised rate of change of age-standardized DALYs** | |
|  |  | **1990-2010** | **2010-2019** |  |  | **1990-2010** | **2010-2019** |  |  | **1990-2010** | **2010-2019** |
| **Global** | 13697119(10071811 to 17569090) | 116.5(73.1 to 174.5) | -53.0(-55.9 to -49.9) |  | 52003(22943 to 93846) | — | -21.2(-36.0 to -1.0) |  | 1341461(582207 to 2456710) | 3244.3(1842.2 to 5372.4) | -45.1(-61.1 to -23.1) |
| **High SDI** | 242143(164861 to 348224) | -77.8(-80.2 to -74.9) | -24.9(-28.9 to -20.8) |  | 3350(1362 to 6870) | — | 2.6(-22.2 to 32.6) |  | 13292(5541 to 27289) | -63.3(-76.4 to -41.6) | -31.0(-50.6 to -3.7) |
| **High-middle SDI** | 1556479(1106655 to 2114403) | 43.1(15.3 to 68.2) | -37.7(-45.6 to -27.1) |  | 85310(40716 to 146906) | — | -17.5(-37.7 to 16.8) |  | 298788(132503 to 556807) | 2553.7(1162.0 to 5798.8) | -48.9(-68.6 to -16.1) |
| **Middle SDI** | 11418310(7498600 to 15874340) | 867.8(533.2 to 1153.8) | -50.4(-55.5 to -44.3) |  | 39107(14733 to 79697) | — | -16.3(-46.2 to 26.2) |  | 901008(327495 to 2028682) | 5425.0(1993.7 to 15304.6) | -53.7(-78.3 to -2.3) |
| **Low-middle SDI** | 17384519(13251507 to 22316430) | 213.3(136.8 to 316.3) | -55.3(-58.8 to -51.6) |  | 55987(21740 to 108244) | — | -39.7(-62.3 to -7.8) |  | 1750776(736856 to 3306041) | 5275.9(1824.6 to 15806.7) | -52.2(-70.6 to -24.2) |
| **Low SDI** | 45495491(34473837 to 56412047) | 8.8(-13.0 to 41.9) | -58.8(-62.4 to -55.3) |  | 70776(26222 to 138773) | — | -7.3(-39.3 to 43.0) |  | 4499354(1771049 to 8649153) | 2034.1(902.0 to 4687.6) | -40.6(-62.6 to -6.6) |
| **High-income** | 650009(491217 to 767850) | -58.6(-65.4 to -50.8) | -10.8(-18.4 to -6.3) |  | 6076(1967 to 15722) | — | 18.0(-34.9 to 134.0) |  | 23415(7985 to 59270) | -43.1(-69.0 to 12.5) | -24.4(-58.4 to 50.1) |
| **High-income North America** | 172318(112532 to 260328) | -85.7(-87.2 to -84.0) | -9.6(-14.9 to -4.7) |  | 1486(412 to 3833) | — | 43.7(-32.6 to 181.8) |  | 5715(1751 to 13822) | -93.9(-96.4 to -88.8) | -8.2(-57.0 to 79.8) |
| **Australasia** | 68633(41240 to 110105) | -80.5(-83.3 to -77.6) | -16.9(-29.9 to -2.3) |  | 1346(361 to 3616) | — | 103.7(-28.3 to 442.5) |  | 5200(1417 to 12792) | -31.8(-79.2 to 155.6) | 30.3(-54.2 to 245.7) |
| **High-income Asia Pacific** | 198406(158700 to 235196) | 52.6(29.0 to 86.7) | -18.7(-24.4 to -13.3) |  | 1456(275 to 4766) | — | 13.2(-65.0 to 174.3) |  | 5592(1128 to 17302) | 232.3(-0.8 to 1119.2) | -27.8(-77.6 to 75.0) |
| **Western Europe** | 348144(226150 to 512162) | -81.8(-83.4 to -80.2) | -28.1(-33.5 to -22.4) |  | 4517(1522 to 10463) | — | 14.3(-25.5 to 69.7) |  | 17295(6332 to 37175) | -50.2(-73.5 to -5.8) | -27.0(-52.4 to 8.1) |
| **Southern Latin America** | 6558230(5155709 to 7053122) | 96.6(71.8 to 109.0) | -9.8(-22.2 to -3.2) |  | 54556(10598 to 185395) | — | 8.0(-64.4 to 180.4) |  | 210984(42611 to 705300) | 900.1(142.4 to 5223.6) | -30.5(-77.2 to 79.8) |
| **Central Europe, Eastern Europe, and Central Asia** | 963615(527124 to 1587462) | 71.3(12.6 to 132.3) | -49.6(-61.7 to -34.6) |  | 287426(137686 to 486382) | — | -16.2(-36.2 to 18.5) |  | 643789(329399 to 1065385) | 3873.9(1697.8 to 9399.6) | -47.3(-60.3 to -26.1) |
| **Eastern Europe** | 1533551(793282 to 2592264) | 105.9(29.7 to 185.2) | -51.9(-65.9 to -34.6) |  | 509083(241606 to 877776) | — | -15.4(-37.3 to 25.2) |  | 1140182(571734 to 1899985) | 4083.2(1643.4 to 10861.4) | -46.8(-60.9 to -21.7) |
| **Central Europe** | 302524(192988 to 461955) | -18.4(-32.1 to 1.0) | -29.2(-37.2 to -15.0) |  | 6976(2266 to 15916) | — | 5.2(-61.8 to 171.2) |  | 15485(5063 to 35643) | 163.6(-1.9 to 645.9) | -34.3(-76.2 to 67.9) |
| **Central Asia** | 480749(272449 to 798100) | -9.6(-39.1 to 19.4) | -38.3(-54.5 to -20.8) |  | 121183(56340 to 218536) | — | -23.9(-46.5 to 2.6) |  | 271821(134432 to 459279) | 15925.1(5645.6 to 63518.1) | -52.4(-66.6 to -36.0) |
| **Latin America and Caribbean** | 3365912(2177363 to 5026168) | -35.7(-44.8 to -23.0) | -33.9(-38.3 to -29.5) |  | 40179(12381 to 94975) | — | 14.4(-31.6 to 88.9) |  | 246889(83556 to 569211) | 875.0(362.2 to 1941.6) | -25.2(-55.6 to 23.3) |
| **Central Latin America** | 2134563(1397266 to 3127112) | -42.0(-52.2 to -28.6) | -24.0(-30.7 to -16.9) |  | 25114(7142 to 59231) | — | 19.2(-39.0 to 116.7) |  | 154840(48783 to 355201) | 1252.1(429.2 to 3768.4) | -21.9(-60.3 to 41.3) |
| **Andean Latin America** | 3687592(2317302 to 5581767) | 18.5(-24.5 to 91.7) | -41.7(-49.9 to -33.7) |  | 98264(28826 to 232358) | — | -19.9(-54.4 to 32.8) |  | 603987(203287 to 1367812) | 926.5(260.2 to 3910.1) | -47.6(-70.3 to -13.6) |
| **Caribbean** | 7498387(4675485 to 11344076) | -18.6(-31.2 to -0.7) | -43.3(-48.3 to -37.0) |  | 15688(2881 to 55029) | — | 36.6(-56.8 to 372.2) |  | 95806(18691 to 322477) | -20.1(-82.1 to 282.0) | -10.8(-71.9 to 206.8) |
| **Tropical Latin America** | 3716747(2362745 to 5564448) | -44.8(-51.6 to -33.6) | -32.3(-40.8 to -26.5) |  | 44245(7084 to 133629) | — | 37.2(-61.7 to 218.3) |  | 271525(44486 to 827112) | 2442.2(469.6 to 24060.0) | -10.5(-75.1 to 108.5) |
| **Southeast Asia, East Asia, and Oceania** | 3096133(2390691 to 3865469) | 549.6(393.0 to 938.7) | -30.7(-36.5 to -22.4) |  | 35905(13529 to 74459) | — | -27.2(-57.9 to 21.1) |  | 192744(78112 to 391331) | 1192.5(359.4 to 5469.0) | -52.5(-72.7 to -21.4) |
| **East Asia** | 628473(401459 to 924372) | 37.3(-14.1 to 316.1) | -13.5(-27.9 to -0.3) |  | 10713(2016 to 33240) | — | -6.2(-73.1 to 135.0) |  | 58843(11712 to 198028) | 153.4(-33.2 to 1881.7) | -38.1(-82.4 to 54.9) |
| **Southeast Asia** | 8136574(6429410 to 9957239) | 1085.3(760.4 to 1388.0) | -37.2(-42.8 to -28.9) |  | 87839(32427 to 185368) | — | -36.6(-65.0 to 8.0) |  | 468841(192764 to 968128) | 7639.7(2541.3 to 31514.8) | -58.7(-77.2 to -29.8) |
| **Oceania** | 12806293(7124790 to 20763899) | 4687.7(2474.9 to 8990.1) | -34.7(-44.7 to -25.0) |  | 226556(51038 to 572777) | — | 194.5(-24.1 to 1030.2) |  | 1204532(280932 to 3220341) | 100098.3(24572.0 to 371311.5) | 91.4(-50.6 to 636.1) |
| **North Africa and Middle East** | 519210(338472 to 760844) | 72.8(19.1 to 172.2) | -33.4(-41.5 to -22.8) |  | 2034(653 to 4863) | — | 2.7(-48.6 to 105.5) |  | 28103(9631 to 65852) | 1752.3(568.2 to 4963.7) | -32.1(-65.8 to 35.5) |
| **South Asia** | 3300086(2324645 to 4367293) | 3372.6(1909.3 to 5856.6) | -64.2(-71.7 to -57.1) |  | 27519(5457 to 69967) | — | -36.6(-80.1 to 54.9) |  | 547198(101319 to 1318904) | 249733.7(44451.8 to 2740404.8) | -57.8(-86.7 to 3.2) |
| **Sub-Saharan Africa** | 101821145(73412218 to 131444960) | 73.1(33.8 to 135.3) | -58.4(-61.2 to -55.6) |  | 124616(47590 to 249340) | — | -25.3(-48.9 to 12.0) |  | 9281623(3736410 to 17280748) | 3304.7(1665.9 to 7151.1) | -50.0(-65.8 to -25.3) |
| **Southern Sub-Saharan Africa** | 347786723(246032444 to 460739958) | 824.8(442.9 to 1553.3) | -59.5(-63.7 to -53.4) |  | 429140(143683 to 1012008) | — | -40.7(-70.9 to 23.9) |  | 31947653(11584959 to 65820526) | 10503.2(2390.7 to 55209.3) | -60.2(-80.6 to -17.0) |
| **Western Sub-Saharan Africa** | 46906722(30778893 to 65458386) | 99.7(49.2 to 174.0) | -46.7(-51.4 to -42.1) |  | 53365(16142 to 129352) | — | -21.2(-57.0 to 43.2) |  | 3996197(1322832 to 9060583) | 1361.9(506.6 to 4177.5) | -47.2(-71.3 to -4.0) |
| **Eastern Sub-Saharan Africa** | 117000650(85069685 to 146573611) | 6.1(-17.5 to 44.4) | -58.7(-62.4 to -55.2) |  | 154480(54312 to 328232) | — | -3.5(-42.6 to 59.2) |  | 11481133(4223483 to 22976990) | 4152.0(1670.4 to 12668.5) | -35.5(-61.6 to 6.1) |
| **Central Sub-Saharan Africa** | 51170554(39799232 to 64268175) | 27.9(-2.7 to 68.5) | -66.3(-70.4 to -60.6) |  | 43766(11054 to 111313) | — | -35.4(-74.8 to 66.3) |  | 3265626(906394 to 8161874) | 747.6(56.0 to 4898.0) | -56.8(-83.2 to 10.9) |

| **S14** | | | | | | | | | | | |
| --- | --- | --- | --- | --- | --- | --- | --- | --- | --- | --- | --- |
| **Group** | **HIV/AIDS - Drug-susceptible Tuberculosis** | | |  | **HIV/AIDS - Extensively drug-resistant Tuberculosis** | | |  | **HIV/AIDS - Multidrug-resistant Tuberculosis without extensive drug resistance** | | |
|  | **Number of Deaths, 2019** | **Annualised rate of change of age-standardised Deaths** | |  | **Number of Deaths, 2019** | **Annualised rate of change of age-standardised Deaths** | |  | **Number of Deaths, 2019** | **Annualised rate of change of age-standardised Deaths** | |
|  |  | **1990-2010** | **2010-2019** |  |  | **1990-2010** | **2010-2019** |  |  | **1990-2010** | **2010-2019** |
| **Global** | 244923(173251 to 319002) | 129.0(78.1 to 203.7) | -52.1(-55.2 to -48.9) |  | 998(429 to 1825) | — | -19.0(-34.5 to 1.6) |  | 24454(10229 to 45371) | 3334.7(1888.3 to 5538.6) | -44.2(-59.9 to -21.9) |
| **High SDI** | 5068(3312 to 7433) | -76.2(-78.8 to -73.0) | -23.2(-27.5 to -18.9) |  | 72(29 to 150) | — | 5.6(-20.7 to 38.4) |  | 279(110 to 571) | -61.6(-75.5 to -39.0) | -28.9(-49.4 to -1.5) |
| **High-middle SDI** | 26882(18229 to 37908) | 43.2(13.7 to 70.5) | -38.3(-46.2 to -27.6) |  | 1628(749 to 2828) | — | -14.2(-35.0 to 21.3) |  | 5522(2364 to 10462) | 2498.3(1107.6 to 5799.4) | -47.6(-67.3 to -14.3) |
| **Middle SDI** | 210401(129459 to 302360) | 1010.5(598.3 to 1421.7) | -50.2(-55.7 to -43.3) |  | 770(282 to 1595) | — | -13.4(-44.8 to 30.9) |  | 17466(6058 to 39854) | 5735.3(2019.6 to 17658.4) | -52.6(-77.9 to 0.8) |
| **Low-middle SDI** | 328708(243790 to 424237) | 249.2(156.8 to 387.7) | -54.1(-58.1 to -50.1) |  | 1114(426 to 2154) | — | -38.8(-61.7 to -7.4) |  | 33923(14063 to 64303) | 5767.8(2111.4 to 17655.1) | -51.0(-69.8 to -22.8) |
| **Low SDI** | 891665(658694 to 1106214) | 14.8(-10.1 to 58.6) | -58.1(-61.6 to -54.4) |  | 1421(525 to 2790) | — | -5.5(-37.8 to 43.3) |  | 89204(34639 to 173041) | 2142.4(922.6 to 4977.6) | -39.6(-61.8 to -6.2) |
| **High-income** | 13087(9629 to 15734) | -55.9(-62.9 to -47.6) | -9.3(-17.3 to -4.4) |  | 128(42 to 328) | — | 20.4(-32.3 to 132.9) |  | 487(166 to 1206) | -39.6(-67.3 to 17.0) | -22.8(-56.7 to 49.0) |
| **High-income North America** | 3717(2329 to 5723) | -84.0(-85.7 to -82.0) | -8.5(-14.2 to -3.4) |  | 34(9 to 88) | — | 43.8(-32.9 to 183.4) |  | 128(38 to 315) | -93.0(-95.9 to -87.3) | -8.0(-57.1 to 81.3) |
| **Australasia** | 1420(810 to 2377) | -79.4(-82.6 to -76.0) | -15.3(-29.5 to 0.8) |  | 30(8 to 80) | — | 107.8(-26.9 to 465.9) |  | 112(30 to 281) | -27.9(-78.1 to 167.8) | 33.1(-53.2 to 262.1) |
| **High-income Asia Pacific** | 4370(3466 to 5197) | 77.1(50.8 to 115.1) | -16.4(-22.6 to -10.6) |  | 33(6 to 109) | — | 16.6(-63.8 to 184.3) |  | 126(25 to 394) | 266.8(8.0 to 1198.9) | -25.5(-76.9 to 81.9) |
| **Western Europe** | 7378(4637 to 11063) | -79.6(-81.4 to -77.7) | -27.1(-33.0 to -20.8) |  | 99(33 to 232) | — | 15.3(-25.7 to 71.6) |  | 374(134 to 812) | -43.6(-69.6 to 6.7) | -26.3(-52.5 to 9.8) |
| **Southern Latin America** | 132758(102029 to 142142) | 116.1(90.0 to 127.0) | -7.0(-20.7 to 0.0) |  | 1157(226 to 3902) | — | 11.1(-63.2 to 187.5) |  | 4399(868 to 14732) | 936.7(156.2 to 5534.1) | -28.6(-76.4 to 84.0) |
| **Central Europe, Eastern Europe, and Central Asia** | 17144(8910 to 29309) | 70.0(8.7 to 133.7) | -48.5(-61.4 to -32.7) |  | 5465(2557 to 9353) | — | -13.0(-33.8 to 23.3) |  | 11926(5831 to 19998) | 3810.8(1680.0 to 9191.6) | -45.7(-58.9 to -23.4) |
| **Eastern Europe** | 27140(13048 to 48254) | 101.8(22.9 to 183.4) | -50.9(-66.1 to -32.6) |  | 9640(4464 to 16794) | — | -12.0(-34.8 to 31.0) |  | 21042(10086 to 35671) | 3976.6(1634.3 to 10494.9) | -45.1(-59.5 to -18.6) |
| **Central Europe** | 5348(3294 to 8283) | -11.3(-27.2 to 11.2) | -26.9(-35.3 to -11.5) |  | 126(41 to 289) | — | 9.6(-58.9 to 171.8) |  | 276(88 to 645) | 216.9(24.1 to 743.6) | -31.6(-74.4 to 68.9) |
| **Central Asia** | 8618(4425 to 14787) | -10.1(-41.6 to 21.5) | -37.7(-54.5 to -18.8) |  | 2338(1072 to 4281) | — | -22.5(-45.5 to 3.7) |  | 5091(2479 to 8903) | 15562.9(5484.2 to 65435.8) | -51.8(-66.1 to -35.6) |
| **Latin America and Caribbean** | 64423(39804 to 98193) | -33.4(-43.7 to -19.4) | -31.9(-37.0 to -27.2) |  | 793(237 to 1888) | — | 18.9(-30.3 to 100.6) |  | 4784(1518 to 11340) | 897.4(370.4 to 1992.0) | -22.5(-54.7 to 30.3) |
| **Central Latin America** | 40810(25801 to 61316) | -42.8(-54.3 to -28.5) | -23.8(-30.6 to -16.1) |  | 508(141 to 1208) | — | 19.9(-38.9 to 118.8) |  | 3078(934 to 7204) | 1247.9(432.8 to 3792.4) | -21.6(-60.3 to 42.1) |
| **Andean Latin America** | 64005(38388 to 98694) | 16.2(-28.5 to 96.1) | -40.9(-49.3 to -32.1) |  | 1771(508 to 4257) | — | -17.9(-53.7 to 38.6) |  | 10709(3431 to 24780) | 866.8(219.7 to 3681.9) | -46.3(-70.0 to -10.0) |
| **Caribbean** | 143951(86444 to 220875) | -14.9(-28.5 to 5.6) | -39.2(-44.8 to -32.4) |  | 319(58 to 1122) | — | 46.5(-51.9 to 387.7) |  | 1911(359 to 6495) | -14.5(-80.2 to 287.3) | -4.6(-68.7 to 216.8) |
| **Tropical Latin America** | 72511(44138 to 111826) | -39.9(-48.2 to -26.9) | -30.2(-39.6 to -23.8) |  | 911(143 to 2804) | — | 42.2(-61.2 to 231.2) |  | 5486(859 to 17137) | 2704.2(528.4 to 25947.7) | -7.6(-74.8 to 115.1) |
| **Southeast Asia, East Asia, and Oceania** | 48022(35335 to 62715) | 576.4(369.8 to 1219.9) | -34.1(-40.7 to -25.2) |  | 694(254 to 1425) | — | -25.0(-56.8 to 23.3) |  | 3622(1426 to 7477) | 1195.8(343.3 to 6363.3) | -51.4(-72.0 to -20.2) |
| **East Asia** | 11596(6851 to 17924) | 40.9(-19.1 to 462.3) | -10.2(-27.0 to 5.2) |  | 225(40 to 710) | — | 0.3(-71.8 to 148.7) |  | 1177(218 to 3756) | 159.7(-31.6 to 2149.6) | -34.8(-81.7 to 60.9) |
| **Southeast Asia** | 127266(95085 to 162234) | 1384.2(811.3 to 2034.5) | -41.8(-48.2 to -33.1) |  | 1717(618 to 3658) | — | -36.3(-65.3 to 9.9) |  | 8957(3608 to 18582) | 9559.5(2926.4 to 41943.3) | -58.8(-77.6 to -28.9) |
| **Oceania** | 257047(134574 to 428823) | 6152.6(3022.5 to 13602.3) | -32.4(-43.1 to -22.8) |  | 4788(1066 to 12189) | — | 209.3(-20.2 to 1068.5) |  | 25037(5682 to 67217) | 123580.9(30449.4 to 472405.0) | 100.0(-48.4 to 656.0) |
| **North Africa and Middle East** | 9297(5784 to 13927) | 83.8(21.7 to 217.9) | -31.8(-40.4 to -20.0) |  | 40(13 to 96) | — | 5.9(-46.6 to 114.2) |  | 534(177 to 1259) | 1825.7(588.4 to 5332.6) | -30.3(-64.8 to 41.2) |
| **South Asia** | 59284(40713 to 79600) | 5185.5(2472.9 to 12902.0) | -64.7(-72.4 to -57.2) |  | 531(104 to 1347) | — | -36.2(-80.1 to 55.6) |  | 10284(1912 to 24589) | 337122.6(62417.5 to 4275906.7) | -57.9(-86.8 to 2.9) |
| **Sub-Saharan Africa** | 2081533(1453313 to 2722437) | 89.8(43.2 to 171.9) | -57.1(-60.2 to -54.1) |  | 2629(990 to 5242) | — | -23.0(-47.3 to 15.5) |  | 193307(75963 to 369192) | 3656.8(1810.3 to 8112.6) | -48.6(-65.0 to -23.2) |
| **Southern Sub-Saharan Africa** | 7018526(4789423 to 9499937) | 1054.6(538.4 to 2125.7) | -58.1(-62.7 to -51.2) |  | 8928(2935 to 20984) | — | -38.1(-69.6 to 30.2) |  | 656549(234008 to 1384386) | 12725.8(2932.9 to 68458.4) | -58.6(-79.8 to -13.4) |
| **Western Sub-Saharan Africa** | 970284(610476 to 1389372) | 117.2(58.5 to 207.6) | -44.0(-49.2 to -39.0) |  | 1164(344 to 2847) | — | -16.5(-54.8 to 52.1) |  | 85753(27788 to 194140) | 1509.2(552.9 to 4604.7) | -44.3(-69.9 to 1.2) |
| **Eastern Sub-Saharan Africa** | 2331897(1651682 to 2925724) | 12.6(-14.3 to 63.8) | -57.9(-61.7 to -54.2) |  | 3139(1101 to 6654) | — | -1.4(-41.2 to 63.5) |  | 230647(81935 to 464310) | 4422.2(1785.1 to 13828.5) | -34.4(-60.9 to 8.8) |
| **Central Sub-Saharan Africa** | 1038968(808374 to 1321928) | 36.1(1.0 to 87.9) | -65.8(-70.4 to -59.8) |  | 930(231 to 2392) | — | -33.3(-74.1 to 70.6) |  | 68372(18430 to 167854) | 799.6(62.5 to 5359.1) | -55.7(-82.7 to 13.5) |

| **S15** | | | | | | | | | | | |
| --- | --- | --- | --- | --- | --- | --- | --- | --- | --- | --- | --- |
| **Group** | **Number of incident cases (age-standardized, both sexes) with 95% uncertainty intervals (2019)** | | |  | **Annualized rate of change of age-standardized rate (%) with 95% uncertainty intervals** | | | | | | |
|  |  |  |  |  | **1990-2010** | | |  | **2010-2019** | | |
|  | **HIV/AIDS - Drug-susceptible Tuberculosis** | **HIV/AIDS - Extensively drug-resistant Tuberculosis** | **HIV/AIDS - Multidrug-resistant Tuberculosis without extensive drug resistance** |  | **HIV/AIDS - Drug-susceptible Tuberculosis** | **HIV/AIDS - Extensively drug-resistant Tuberculosis** | **HIV/AIDS - Multidrug-resistant Tuberculosis without extensive drug resistance** |  | **HIV/AIDS - Drug-susceptible Tuberculosis** | **HIV/AIDS - Drug-susceptible Tuberculosis** | **HIV/AIDS - Multidrug-resistant Tuberculosis without extensive drug resistance** |
| **Global** | 1370314(1199744 to 1564779) | 2104(1506 to 2902) | 59225(41552 to 83609) |  | 118.2(108.2 to 128.0) | — | 2294.7(1303.9 to 3839.2) |  | -33.8(-36.0 to -31.4) | 3.3(-23.0 to 38.9) | -30.1(-50.3 to -1.2) |
| **Afghanistan** | 7354(5853 to 8856) | 15(3 to 48) | 421(73 to 1358) |  | -24.6(-31.5 to -17.4) | — | 3942.7(850.2 to 50810.0) |  | -18.9(-30.7 to -7.6) | -13.6(-83.5 to 208.0) | -43.1(-89.1 to 103.1) |
| **Albania** | 6146(5246 to 7199) | 13(2 to 38) | 58(11 to 172) |  | -40.6(-46.6 to -33.4) | — | 342.1(-2.7 to 4071.7) |  | -14.0(-20.9 to -6.7) | -29.4(-86.2 to 133.7) | -56.2(-91.4 to 45.4) |
| **Algeria** | 23515(19641 to 27586) | 21(2 to 97) | 599(56 to 2746) |  | -9.8(-18.4 to -1.3) | — | 22.1(-85.0 to 567.6) |  | -26.0(-32.7 to -19.8) | 45.5(-73.4 to 408.9) | -4.1(-82.5 to 235.6) |
| **American Samoa** | 17733(15205 to 20640) | 6(1 to 26) | 66(6 to 280) |  | 34.8(25.8 to 44.2) | — | 249.3(-75.6 to 5137.4) |  | -22.0(-26.7 to -16.7) | 36.3(-74.3 to 696.7) | -11.8(-83.4 to 415.6) |
| **Andorra** | 44689(37802 to 53548) | 17(2 to 70) | 137(15 to 560) |  | -25.2(-30.7 to -19.6) | — | 18.3(-89.0 to 1103.0) |  | -15.6(-20.8 to -10.3) | -4.6(-85.0 to 473.9) | -39.0(-90.4 to 267.2) |
| **Angola** | 4245346(3569768 to 4914670) | 981(131 to 3520) | 152063(20252 to 545553) |  | 411.3(367.1 to 449.2) | — | 4975.9(381.4 to 59713.9) |  | -13.3(-21.8 to -4.8) | 38.9(-70.6 to 542.2) | -7.6(-80.4 to 327.1) |
| **Antigua and Barbuda** | 264864(220946 to 316291) | 183(18 to 794) | 2312(228 to 10054) |  | -40.3(-46.7 to -33.6) | — | -58.9(-96.3 to 395.1) |  | -33.9(-38.8 to -29.2) | 37.9(-75.0 to 807.8) | -10.4(-83.7 to 489.3) |
| **Argentina** | 841614(715773 to 990530) | 1949(270 to 7298) | 15499(2151 to 58017) |  | -17.6(-29.5 to -4.1) | — | 346.9(-8.7 to 3530.5) |  | 1.2(-7.5 to 11.4) | 25.2(-75.8 to 278.2) | -19.9(-84.5 to 141.7) |
| **Armenia** | 86424(53480 to 111738) | 4436(925 to 10414) | 20248(4223 to 47548) |  | 131.9(0.0 to 138.2) | — | 60876.9(0.0 to 597179.9) |  | -20.8(-47.4 to -2.2) | 0.5(-72.5 to 110.0) | -37.6(-82.9 to 30.5) |
| **Australia** | 12194(10548 to 14289) | 61(25 to 126) | 483(196 to 999) |  | -75.9(-77.6 to -73.9) | — | 11.4(-75.7 to 579.4) |  | -21.6(-28.1 to -14.9) | 122.6(-38.4 to 736.2) | 42.4(-60.6 to 435.2) |
| **Austria** | 22103(18945 to 25712) | 110(41 to 232) | 878(323 to 1847) |  | -64.5(-68.5 to 0.0) | — | 239.6(-10.8 to 2910.1) |  | -32.9(-38.3 to -27.5) | -23.5(-72.2 to 74.0) | -51.0(-82.2 to 11.4) |
| **Azerbaijan** | 35440(23649 to 46389) | 2730(841 to 5381) | 12460(3838 to 24570) |  | -15.1(-30.6 to -0.9) | — | 32035.1(7261.2 to 347546.2) |  | -33.7(-56.7 to -9.3) | 42.5(-65.2 to 305.2) | -11.5(-78.4 to 151.4) |
| **Bahamas** | 317583(268586 to 375754) | 474(64 to 1784) | 5999(804 to 22595) |  | -32.0(-38.4 to -24.6) | — | 746.3(47.2 to 9434.7) |  | -20.7(-27.1 to -13.3) | 80.2(-74.8 to 792.5) | 17.0(-83.6 to 479.8) |
| **Bahrain** | 35502(29546 to 42734) | 46(7 to 142) | 1291(198 to 4011) |  | -45.3(-51.0 to -39.2) | — | 1141.2(174.4 to 13978.3) |  | -28.6(-36.7 to -20.4) | 16.4(-83.0 to 353.0) | -23.3(-88.8 to 198.7) |
| **Bangladesh** | 150526(128973 to 176805) | 171(41 to 409) | 6957(1691 to 16703) |  | 0.0(0.0 to 0.0) | — | 0.0(0.0 to 0.0) |  | -16.7(-25.0 to -7.7) | 63.8(-59.4 to 343.2) | 8.3(-73.1 to 193.2) |
| **Barbados** | 84760(71523 to 100296) | 28(2 to 134) | 356(26 to 1698) |  | -45.4(-50.5 to -39.0) | — | -86.7(-99.0 to 164.0) |  | -35.5(-39.5 to -31.0) | 51.1(-82.4 to 987.9) | -1.8(-88.6 to 606.2) |
| **Belarus** | 41098(22871 to 57503) | 8837(5756 to 12660) | 40333(26282 to 57759) |  | 6.6(-16.5 to 23.5) | — | 5292.8(0.0 to 46024.3) |  | -59.0(-75.7 to -44.1) | -12.1(-40.2 to 23.2) | -45.4(-62.9 to -23.5) |
| **Belgium** | 31843(27491 to 37016) | 89(30 to 219) | 709(235 to 1742) |  | -56.5(-59.6 to -53.1) | — | 7.3(-68.4 to 545.4) |  | -32.6(-37.1 to -28.2) | -15.0(-73.0 to 115.7) | -45.6(-82.7 to 38.0) |
| **Belize** | 571469(494481 to 668930) | 276(32 to 1178) | 3489(399 to 14925) |  | -25.0(-33.3 to -16.2) | — | -44.1(-94.1 to 543.6) |  | -28.2(-33.8 to -21.6) | 81.5(-67.4 to 945.8) | 17.9(-78.8 to 579.7) |
| **Benin** | 2098072(1815894 to 2436716) | 314(59 to 881) | 48732(9127 to 136512) |  | 305.3(259.0 to 354.6) | — | 3422.6(712.6 to 31507.4) |  | -21.3(-27.4 to -14.6) | 52.7(-72.0 to 354.1) | 1.6(-81.3 to 202.0) |
| **Bermuda** | 274352(230042 to 329546) | 139(8 to 828) | 1758(97 to 10479) |  | -52.1(-56.9 to -46.2) | — | -75.4(-98.6 to 220.8) |  | -31.1(-36.4 to -25.5) | 143.0(-73.0 to 1696.5) | 57.8(-82.5 to 1066.8) |
| **Bhutan** | 54697(44682 to 66366) | 70(10 to 228) | 2866(388 to 9317) |  | -25.6(-36.6 to -14.3) | — | 871.5(-6.7 to 14494.4) |  | -24.9(-34.3 to -14.4) | 17.6(-71.7 to 445.0) | -22.2(-81.3 to 260.1) |
| **Bolivia (Plurinational State of)** | 239198(195505 to 284354) | 1171(174 to 3717) | 14832(2206 to 47107) |  | -36.5(-45.7 to -27.8) | — | 208.7(-63.2 to 3252.0) |  | -37.9(-46.0 to -31.2) | 55.8(-57.4 to 586.3) | 1.2(-72.3 to 345.8) |
| **Bosnia and Herzegovina** | 6503(5488 to 7682) | 8(2 to 22) | 37(10 to 103) |  | -63.5(-66.9 to -59.7) | — | -17.8(-79.7 to 464.7) |  | -20.1(-25.8 to -13.9) | -2.5(-74.1 to 175.4) | -39.4(-83.9 to 71.0) |
| **Botswana** | 39305856(31952411 to 47440098) | 14724(2754 to 43416) | 2282186(426844 to 6732229) |  | 31.4(14.6 to 48.9) | — | 1876.2(417.9 to 16555.1) |  | -40.4(-48.0 to -32.6) | 33.6(-70.9 to 249.9) | -11.1(-80.7 to 132.8) |
| **Brazil** | 526040(440187 to 622524) | 1614(307 to 4450) | 20430(3886 to 56359) |  | -13.8(-19.7 to -6.7) | — | 4324.9(778.6 to 51469.3) |  | -14.5(-21.7 to -8.6) | 87.4(-57.7 to 385.2) | 21.7(-72.5 to 215.3) |
| **Brunei Darussalam** | 42340(36555 to 49415) | 21(3 to 71) | 171(27 to 562) |  | 44.4(30.2 to 60.6) | — | 156.6(-68.0 to 2842.7) |  | 1.2(-6.8 to 9.4) | 160.5(-64.4 to 1318.6) | 66.7(-77.2 to 809.5) |
| **Bulgaria** | 4138(3534 to 4818) | 28(7 to 67) | 126(33 to 306) |  | 48.3(0.0 to 52.6) | — | 2908.1(0.0 to 25943.1) |  | -35.8(-41.1 to -30.4) | -49.0(-86.9 to 24.6) | -68.3(-91.8 to -22.5) |
| **Burkina Faso** | 3137317(2678332 to 3633899) | 731(110 to 2431) | 113303(17110 to 376918) |  | -53.2(-58.5 to -47.7) | — | 350.3(-39.1 to 6299.5) |  | -49.6(-54.2 to -44.2) | -30.7(-85.7 to 239.3) | -53.9(-90.5 to 125.6) |
| **Burundi** | 7210624(5969033 to 8483240) | 1777(237 to 6186) | 275398(36686 to 957886) |  | -21.2(-29.9 to -11.6) | — | 2200.1(173.6 to 30718.0) |  | -59.2(-63.1 to -55.6) | -17.2(-79.7 to 236.8) | -44.9(-86.5 to 124.1) |
| **Cabo Verde** | 602966(503059 to 710763) | 164(22 to 512) | 25488(3450 to 79444) |  | -7.3(-19.6 to 4.6) | — | 696.7(-8.6 to 9921.2) |  | -53.5(-58.3 to -48.5) | -26.7(-84.3 to 242.7) | -51.2(-89.6 to 128.0) |
| **Cambodia** | 1873423(1634689 to 2170665) | 1509(213 to 5377) | 16553(2339 to 58899) |  | 1435.3(1326.2 to 1546.0) | — | 26992.3(4521.9 to 280634.7) |  | -52.4(-55.8 to -48.5) | -57.5(-91.7 to 33.3) | -72.5(-94.6 to -13.7) |
| **Cameroon** | 7139320(6058050 to 8412317) | 1266(178 to 3929) | 196280(27587 to 608885) |  | 192.6(159.0 to 229.2) | — | 2088.3(119.7 to 26162.2) |  | -39.9(-45.6 to -33.5) | -17.6(-80.0 to 300.3) | -45.2(-86.7 to 166.6) |
| **Canada** | 40861(35544 to 47088) | 77(28 to 179) | 609(220 to 1426) |  | -58.2(-61.0 to -54.2) | — | -40.7(-77.5 to 100.5) |  | -21.1(-27.1 to -16.0) | 25.3(-57.5 to 194.8) | -19.8(-72.8 to 88.7) |
| **Central African Republic** | 22427469(19991428 to 25160081) | 2515(388 to 8217) | 389878(60106 to 1273951) |  | 89.0(75.3 to 103.3) | — | 877.3(18.4 to 9118.3) |  | -33.8(-38.1 to -29.3) | 15.6(-74.4 to 408.2) | -23.1(-83.0 to 238.2) |
| **Chad** | 5877487(5044327 to 6826667) | 1236(174 to 3730) | 191647(27025 to 578191) |  | 51.8(36.0 to 67.5) | — | 1103.4(6.9 to 17996.7) |  | -12.4(-20.3 to -2.8) | 19.8(-73.3 to 412.5) | -20.3(-82.2 to 241.0) |
| **Chile** | 376300(326166 to 436096) | 602(202 to 1375) | 4791(1609 to 10940) |  | -18.1(-25.6 to -10.5) | — | 236.6(-35.3 to 2706.1) |  | -15.5(-21.6 to -9.3) | 39.1(-69.9 to 471.5) | -11.0(-80.7 to 265.9) |
| **China** | 130924(109813 to 151208) | 628(107 to 1938) | 6889(1173 to 21248) |  | 42.7(25.5 to 54.1) | — | 151.0(-26.2 to 1028.2) |  | 2.2(-9.3 to 10.5) | 18.4(-74.7 to 226.9) | -23.4(-83.7 to 111.5) |
| **Colombia** | 212401(177659 to 246646) | 915(158 to 2474) | 11591(2007 to 31342) |  | 17.4(4.9 to 30.5) | — | 2035.9(346.5 to 17849.3) |  | -18.9(-27.2 to -11.6) | 52.9(-64.4 to 278.0) | -0.7(-76.9 to 145.8) |
| **Comoros** | 566843(438289 to 688057) | 499(60 to 1632) | 77286(9298 to 252935) |  | 109.7(82.3 to 139.1) | — | 9436.3(1016.6 to 116234.9) |  | -3.1(-21.6 to 10.0) | 324.1(-12.1 to 1899.3) | 182.1(-41.6 to 1231.7) |
| **Congo** | 5187580(4425072 to 6017385) | 844(98 to 2950) | 130749(15146 to 457216) |  | -14.9(-21.2 to -9.0) | — | 359.1(-56.0 to 6298.7) |  | -32.2(-38.3 to -25.5) | 36.0(-71.6 to 584.6) | -9.6(-81.1 to 355.8) |
| **Cook Islands** | 107277(91680 to 125995) | 33(3 to 138) | 360(32 to 1508) |  | 228.0(206.1 to 250.2) | — | 949.9(-20.1 to 13099.9) |  | 51.1(41.6 to 61.1) | 175.9(-49.9 to 1504.5) | 78.5(-67.6 to 938.0) |
| **Costa Rica** | 66400(56521 to 76956) | 129(18 to 450) | 1627(233 to 5701) |  | -19.7(-27.0 to -12.2) | — | 1811.0(218.5 to 22863.1) |  | -35.5(-40.9 to -30.2) | 5.4(-76.2 to 223.8) | -31.6(-84.5 to 110.4) |
| **Croatia** | 8064(6989 to 9421) | 8(2 to 23) | 36(8 to 105) |  | -26.4(-33.0 to -19.9) | — | 64.8(-64.4 to 1090.9) |  | -52.4(-55.8 to -49.1) | -55.5(-88.9 to 34.2) | -72.4(-93.1 to -16.6) |
| **Cuba** | 32500(27859 to 38326) | 76(14 to 218) | 957(184 to 2762) |  | 6.4(-5.5 to 20.0) | — | -6.4(-70.5 to 289.2) |  | -17.3(-24.4 to -9.7) | 122.8(-55.2 to 652.4) | 44.7(-70.9 to 388.7) |
| **Cyprus** | 11727(9852 to 13920) | 35(7 to 108) | 281(52 to 861) |  | -4.7(-13.4 to 3.7) | — | 1168.0(169.1 to 14032.9) |  | -5.3(-13.2 to 4.0) | -60.0(-91.5 to 21.7) | -74.4(-94.6 to -22.1) |
| **Czechia** | 12453(10691 to 14561) | 68(24 to 157) | 312(111 to 718) |  | -18.7(-25.7 to -11.0) | — | 141.1(-32.9 to 1380.8) |  | -22.3(-28.0 to -16.1) | 14.2(-61.8 to 181.8) | -29.1(-76.3 to 75.2) |
| **C么te d'Ivoire** | 4436340(3734829 to 5194052) | 1125(176 to 3785) | 174304(27282 to 586538) |  | 15.8(2.6 to 30.3) | — | 204.1(-34.6 to 1776.0) |  | -47.1(-52.2 to -42.4) | -14.7(-78.9 to 163.7) | -43.2(-85.9 to 75.4) |
| **Democratic People's Republic of Korea** | 360707(313291 to 407778) | 1850(587 to 4284) | 20290(6437 to 46987) |  | 129.4(101.8 to 144.7) | — | 14344.7(1986.9 to 137906.9) |  | 27.1(14.4 to 40.8) | 108.9(-51.1 to 749.6) | 35.2(-68.3 to 449.5) |
| **Democratic Republic of the Congo** | 2889865(2464428 to 3305433) | 570(73 to 2101) | 88305(11248 to 325662) |  | 11.2(2.3 to 19.6) | — | 641.7(-24.2 to 7472.8) |  | -58.7(-62.3 to -54.8) | -13.0(-82.9 to 241.9) | -42.1(-88.6 to 127.1) |
| **Denmark** | 14437(12393 to 16893) | 41(12 to 108) | 323(94 to 859) |  | 128.4(113.3 to 146.0) | — | 803.2(100.4 to 6601.4) |  | -45.1(-48.7 to -41.2) | 19.8(-66.4 to 271.4) | -23.3(-78.5 to 137.8) |
| **Djibouti** | 3598780(2948263 to 4306789) | 2012(858 to 4150) | 311873(133048 to 643369) |  | 2196.7(1916.3 to 2523.3) | — | 249047.0(55839.4 to 2383913.3) |  | -42.8(-50.9 to -34.8) | 41.5(-62.4 to 417.3) | -5.9(-75.0 to 244.3) |
| **Dominica** | 341026(292570 to 397840) | 225(21 to 956) | 2855(269 to 12119) |  | -37.4(-43.3 to -30.4) | — | -71.9(-97.5 to 399.6) |  | -18.4(-23.8 to -12.6) | 76.2(-67.0 to 927.1) | 14.5(-78.5 to 567.7) |
| **Dominican Republic** | 765021(642460 to 907362) | 394(39 to 1823) | 4991(491 to 23088) |  | 33.8(18.2 to 52.3) | — | 3.5(-91.5 to 1058.9) |  | -23.6(-29.1 to -18.0) | 100.2(-67.9 to 1144.4) | 30.0(-79.2 to 708.8) |
| **Ecuador** | 623748(492607 to 745068) | 3241(586 to 10114) | 41039(7431 to 127999) |  | 17.3(2.4 to 33.6) | — | 2778.0(306.8 to 28788.2) |  | -23.0(-32.8 to -14.4) | 12.7(-73.4 to 249.2) | -26.8(-82.7 to 126.5) |
| **Egypt** | 9383(7931 to 11027) | 20(4 to 51) | 556(126 to 1432) |  | -51.9(-56.7 to -47.1) | — | 1552.8(319.7 to 14367.7) |  | -27.3(-35.7 to -19.4) | -5.0(-80.7 to 174.3) | -37.4(-87.3 to 80.7) |
| **El Salvador** | 122162(104063 to 141372) | 220(28 to 883) | 2787(355 to 11178) |  | 2.9(-9.5 to 14.5) | — | 1008.4(9.3 to 11237.6) |  | -25.0(-31.0 to -18.3) | 7.5(-73.9 to 447.8) | -30.2(-83.0 to 255.9) |
| **Equatorial Guinea** | 6256854(5224641 to 7429523) | 1259(161 to 3992) | 195190(24946 to 618745) |  | 194.6(165.8 to 228.3) | — | 1753.3(99.5 to 21355.5) |  | -25.4(-32.8 to -17.7) | 65.5(-66.0 to 604.1) | 10.1(-77.4 to 368.6) |
| **Eritrea** | 3183058(2620383 to 3775909) | 1007(149 to 3306) | 156027(23046 to 512401) |  | 52.3(34.0 to 71.1) | — | 5193.0(516.7 to 76188.0) |  | -43.9(-49.8 to -37.5) | 14.1(-73.2 to 410.1) | -24.1(-82.1 to 239.3) |
| **Estonia** | 116770(87575 to 147565) | 7312(3775 to 12318) | 33369(17248 to 56263) |  | 334.7(269.9 to 399.3) | — | 1735.5(678.6 to 4644.6) |  | -42.2(-54.5 to -31.1) | -36.6(-66.6 to 8.7) | -60.6(-79.2 to -32.5) |
| **Eswatini** | 50761974(38611250 to 61398953) | 45166(10692 to 114374) | 7000845(1657589 to 17734437) |  | 537.2(447.3 to 629.6) | — | 20153.3(6009.5 to 105306.1) |  | -41.4(-53.0 to -32.0) | -12.1(-78.1 to 104.5) | -41.6(-85.4 to 36.0) |
| **Ethiopia** | 3564191(2986049 to 4155638) | 946(176 to 3262) | 146612(27297 to 505472) |  | 13.9(6.2 to 21.7) | — | 4095.8(463.3 to 53857.2) |  | -45.7(-50.4 to -42.6) | 27.6(-66.8 to 311.9) | -15.1(-77.9 to 174.1) |
| **Fiji** | 61027(53375 to 69139) | 13(1 to 57) | 147(13 to 620) |  | 69.9(58.8 to 81.1) | — | 515.2(-60.8 to 8080.7) |  | -19.9(-24.5 to -14.9) | 106.2(-66.1 to 1084.4) | 33.4(-78.0 to 667.1) |
| **Finland** | 8583(7371 to 9981) | 46(14 to 115) | 363(112 to 911) |  | -56.9(-59.8 to -54.0) | — | 258.0(-16.7 to 2852.1) |  | -52.3(-56.3 to -49.0) | 21.0(-64.0 to 255.8) | -22.6(-77.0 to 127.7) |
| **France** | 41028(35718 to 47323) | 120(42 to 262) | 956(331 to 2086) |  | -82.3(-83.6 to -80.9) | — | -40.2(-80.7 to 167.2) |  | -30.5(-34.9 to -26.4) | 23.6(-58.1 to 199.3) | -20.9(-73.2 to 91.6) |
| **Gabon** | 8417993(7097087 to 9931713) | 2061(263 to 7404) | 319460(40786 to 1147570) |  | 157.0(133.1 to 177.3) | — | 1988.4(109.0 to 26079.0) |  | -42.1(-47.5 to -36.2) | 13.4(-73.2 to 410.6) | -24.5(-82.1 to 239.6) |
| **Gambia** | 4160021(3544853 to 4875392) | 740(100 to 2431) | 114749(15549 to 376767) |  | 336.2(279.6 to 389.4) | — | 3129.9(263.5 to 40082.4) |  | -34.1(-38.8 to -28.2) | -15.3(-82.8 to 273.6) | -43.7(-88.6 to 148.3) |
| **Georgia** | 150819(118607 to 186117) | 9934(5585 to 16339) | 45338(25511 to 74591) |  | 94.9(78.1 to 113.5) | — | 21055.1(5775.6 to 178112.8) |  | 17.4(-3.3 to 38.3) | 133.0(35.0 to 263.9) | 44.8(-16.1 to 126.1) |
| **Germany** | 22336(19030 to 26106) | 137(54 to 280) | 1089(429 to 2230) |  | -79.9(-81.3 to -78.1) | — | -29.4(-77.5 to 242.3) |  | -12.5(-20.5 to -3.5) | 129.1(-10.8 to 403.8) | 46.6(-42.9 to 222.4) |
| **Ghana** | 5323549(4508690 to 6296318) | 1068(162 to 3163) | 165544(25099 to 490347) |  | 67.8(47.0 to 88.1) | — | 1110.0(40.3 to 15346.5) |  | -35.7(-42.1 to -28.4) | -1.8(-78.3 to 334.0) | -34.7(-85.6 to 188.6) |
| **Greece** | 23745(20490 to 27287) | 114(19 to 353) | 908(148 to 2809) |  | -65.3(-67.6 to -62.6) | — | 372.8(4.2 to 4652.0) |  | -6.6(-14.7 to 1.2) | -17.3(-81.0 to 119.7) | -47.1(-87.8 to 40.5) |
| **Greenland** | 398454(331937 to 478045) | 1261(171 to 4259) | 10032(1363 to 33869) |  | 0.3(-9.4 to 9.7) | — | 15.5(-87.6 to 998.0) |  | -18.2(-25.7 to -10.3) | 61.7(-64.7 to 727.4) | 3.5(-77.4 to 430.0) |
| **Grenada** | 130199(110043 to 153362) | 99(8 to 473) | 1250(106 to 5990) |  | -51.5(-56.7 to -45.4) | — | -51.7(-95.5 to 321.1) |  | -32.6(-37.5 to -27.5) | 117.7(-71.6 to 1341.8) | 41.4(-81.5 to 836.9) |
| **Guam** | 70716(60690 to 81953) | 38(4 to 133) | 414(42 to 1460) |  | -23.9(-29.5 to -17.8) | — | 1017.7(105.1 to 13088.1) |  | -20.0(-25.2 to -14.7) | -67.3(-95.5 to 25.5) | -78.9(-97.1 to -18.9) |
| **Guatemala** | 324403(282215 to 372222) | 805(120 to 2566) | 10192(1521 to 32488) |  | -55.6(-60.9 to -50.2) | — | 697.3(9.7 to 8991.1) |  | -29.8(-35.7 to -23.5) | 0.3(-73.4 to 213.5) | -34.8(-82.7 to 103.6) |
| **Guinea** | 4877472(4160318 to 5644967) | 828(113 to 2713) | 128286(17508 to 420482) |  | 169.3(138.8 to 199.8) | — | 1688.5(90.4 to 23429.6) |  | -19.7(-26.6 to -11.2) | 2.6(-74.4 to 359.7) | -31.7(-83.0 to 205.8) |
| **Guinea-Bissau** | 8906372(7525665 to 10469795) | 1242(190 to 3990) | 192457(29510 to 618284) |  | 161.0(129.9 to 192.9) | — | 1756.1(134.4 to 23399.0) |  | -26.8(-33.5 to -18.6) | -16.1(-81.9 to 256.9) | -44.2(-87.9 to 137.5) |
| **Guyana** | 1027980(871452 to 1208754) | 774(70 to 4187) | 9800(888 to 53044) |  | 35.2(21.5 to 50.5) | — | 149.6(-76.5 to 2763.9) |  | -21.9(-29.2 to -14.5) | 104.8(-67.0 to 1182.9) | 33.0(-78.6 to 734.3) |
| **Haiti** | 1545262(1342211 to 1779988) | 598(61 to 2379) | 7573(770 to 30134) |  | -12.5(-20.5 to -4.5) | — | -13.3(-92.3 to 800.8) |  | -37.7(-42.9 to -32.6) | 50.8(-75.7 to 976.5) | -2.0(-84.3 to 599.4) |
| **Honduras** | 303801(261154 to 348861) | 749(119 to 2475) | 9486(1505 to 31323) |  | -42.1(-48.3 to -36.8) | — | 1547.9(145.5 to 19742.7) |  | -13.3(-20.3 to -6.0) | 40.9(-65.2 to 318.1) | -8.5(-77.4 to 171.7) |
| **Hungary** | 7964(6856 to 9377) | 29(5 to 99) | 134(25 to 453) |  | -69.2(-71.9 to -66.4) | — | 332.2(-1.9 to 4414.0) |  | -59.0(-62.6 to -55.2) | -74.9(-95.0 to -13.5) | -84.4(-96.9 to -46.2) |
| **Iceland** | 18649(15640 to 22137) | 28(4 to 106) | 225(32 to 844) |  | -61.2(-65.2 to -57.5) | — | 395.2(-13.6 to 4279.1) |  | -31.7(-36.0 to -25.7) | -79.1(-96.0 to -29.8) | -86.6(-97.4 to -55.1) |
| **India** | 529986(427223 to 635758) | 1247(231 to 3332) | 50892(9421 to 135915) |  | 803.9(718.1 to 890.9) | — | 73173.0(11901.4 to 1134546.8) |  | -54.3(-60.1 to -50.1) | -15.3(-78.1 to 130.6) | -44.0(-85.5 to 52.5) |
| **Indonesia** | 4980350(4334920 to 5707500) | 2021(241 to 7564) | 22166(2646 to 82929) |  | 0.0(0.0 to 0.0) | — | 0.0(0.0 to 0.0) |  | -7.8(-11.0 to -3.6) | -35.6(-87.7 to 300.8) | -58.3(-92.1 to 159.3) |
| **Iran (Islamic Republic of)** | 248141(213728 to 287396) | 186(50 to 455) | 5264(1408 to 12890) |  | 53.5(40.2 to 67.7) | — | 3047.5(460.8 to 42203.8) |  | -2.2(-9.0 to 4.7) | 61.9(-67.1 to 612.4) | 6.7(-78.3 to 369.5) |
| **Iraq** | 701(592 to 823) | 1(0 to 2) | 26(8 to 59) |  | 24.5(12.7 to 38.1) | — | 3531.7(733.6 to 50100.2) |  | -39.7(-45.1 to -34.8) | 8.6(-72.8 to 285.5) | -28.4(-82.0 to 154.0) |
| **Ireland** | 47238(40618 to 55085) | 102(27 to 299) | 814(213 to 2376) |  | -53.6(-57.2 to -50.1) | — | 68.5(-60.3 to 1474.3) |  | -39.6(-43.8 to -35.1) | -27.8(-83.6 to 126.6) | -53.8(-89.5 to 45.0) |
| **Israel** | 12966(10810 to 15339) | 120(39 to 276) | 956(310 to 2199) |  | -60.5(-63.7 to -56.2) | — | -7.8(-75.2 to 551.6) |  | -34.3(-42.1 to -27.4) | 19.1(-64.7 to 214.2) | -23.8(-77.4 to 100.8) |
| **Italy** | 16378(13776 to 19610) | 73(28 to 151) | 580(221 to 1200) |  | -70.4(-72.0 to -68.7) | — | 28.7(-63.8 to 625.8) |  | -36.5(-40.0 to -33.4) | -30.3(-72.8 to 51.1) | -55.4(-82.6 to -3.3) |
| **Jamaica** | 115735(97935 to 136539) | 169(27 to 579) | 2140(337 to 7330) |  | -5.5(-14.5 to 4.3) | — | 102.6(-78.2 to 3255.4) |  | -46.2(-50.4 to -41.7) | 41.5(-74.3 to 637.7) | -8.1(-83.3 to 379.3) |
| **Japan** | 6756(5591 to 7971) | 9(1 to 37) | 69(8 to 291) |  | -24.3(-30.5 to -17.2) | — | -9.8(-87.1 to 470.3) |  | -35.2(-38.5 to -31.9) | 16.7(-79.1 to 376.9) | -25.3(-86.6 to 205.2) |
| **Jordan** | 2307(1861 to 2781) | 4(1 to 15) | 120(21 to 421) |  | -32.3(-38.8 to -26.5) | — | 2391.1(443.9 to 31265.5) |  | -23.1(-32.2 to -15.7) | -22.3(-83.9 to 135.7) | -48.8(-89.4 to 55.4) |
| **Kazakhstan** | 46694(33885 to 59280) | 3724(1987 to 6019) | 16996(9065 to 27470) |  | 13.4(-4.4 to 31.2) | — | 17360.0(4407.2 to 145813.5) |  | -42.6(-56.0 to -27.7) | -54.0(-74.1 to -26.3) | -71.4(-83.9 to -54.2) |
| **Kenya** | 10866988(9250027 to 12785919) | 1308(413 to 3026) | 202697(63965 to 469028) |  | 48.2(41.0 to 55.3) | — | 5593.2(1381.4 to 74982.1) |  | -31.6(-34.7 to -28.3) | -4.9(-79.2 to 296.0) | -36.8(-86.1 to 163.5) |
| **Kiribati** | 155682(137309 to 174736) | 50(5 to 199) | 546(53 to 2181) |  | -32.0(-35.4 to -28.4) | — | 134.2(-80.1 to 2878.7) |  | -9.7(-14.9 to -4.6) | 53.8(-73.8 to 884.5) | -0.5(-83.0 to 536.8) |
| **Kuwait** | 5172(4315 to 6200) | 4(1 to 9) | 104(28 to 269) |  | -36.3(-42.1 to -30.0) | — | 605.3(66.3 to 10113.5) |  | -42.2(-47.5 to -36.7) | -2.2(-76.6 to 220.7) | -35.5(-84.6 to 111.4) |
| **Kyrgyzstan** | 122411(64389 to 170068) | 13337(4721 to 25301) | 60870(21536 to 115451) |  | 2.0(-16.1 to 20.5) | — | 59225.9(14159.8 to 615952.5) |  | -1.5(-48.2 to 43.3) | 44.2(-51.2 to 204.8) | -10.4(-69.7 to 89.4) |
| **Lao People's Democratic Republic** | 2102696(1800267 to 2489161) | 1090(124 to 4015) | 11953(1362 to 44047) |  | 0.0(0.0 to 0.0) | — | 0.0(0.0 to 0.0) |  | -24.3(-29.4 to -18.5) | -37.5(-88.6 to 257.9) | -59.6(-92.6 to 131.6) |
| **Latvia** | 206315(169727 to 249420) | 6109(2953 to 11038) | 27880(13482 to 50413) |  | 41.0(20.8 to 73.1) | — | 9.4(-41.5 to 138.1) |  | -29.7(-39.2 to -20.8) | -16.4(-57.8 to 51.5) | -48.1(-73.7 to -5.9) |
| **Lebanon** | 30577(25627 to 36192) | 25(3 to 100) | 706(88 to 2832) |  | -49.9(-54.5 to -45.2) | — | 510.7(-24.1 to 7166.5) |  | -32.9(-38.6 to -28.3) | 28.7(-74.3 to 373.5) | -15.2(-83.0 to 212.0) |
| **Lesotho** | 77828407(65784420 to 91494815) | 27273(9627 to 57784) | 4227319(1491758 to 8957650) |  | 170.0(137.5 to 206.2) | — | 13693.2(2942.4 to 156703.0) |  | -16.5(-25.3 to -6.2) | 42.7(-68.8 to 462.3) | -5.1(-79.2 to 274.2) |
| **Liberia** | 1094349(924360 to 1280820) | 170(20 to 688) | 26360(3085 to 106750) |  | 94.4(69.7 to 121.0) | — | 975.2(-3.4 to 13473.6) |  | -36.6(-42.3 to -30.3) | -11.4(-79.8 to 297.6) | -41.1(-86.5 to 164.3) |
| **Libya** | 132109(110640 to 156979) | 114(13 to 421) | 3218(366 to 11935) |  | 9.3(-3.8 to 22.1) | — | 970.9(8.0 to 13405.6) |  | -0.4(-8.8 to 8.1) | 61.9(-72.5 to 786.2) | 6.7(-81.8 to 483.8) |
| **Lithuania** | 52117(41563 to 62992) | 3187(1822 to 4925) | 14545(8320 to 22478) |  | 22.8(0.0 to 38.7) | — | 477.8(0.0 to 2195.8) |  | -39.0(-49.4 to -28.7) | 0.4(-40.4 to 51.8) | -37.6(-63.0 to -5.7) |
| **Luxembourg** | 22806(18903 to 27300) | 94(14 to 325) | 749(108 to 2582) |  | -68.7(-71.4 to -65.6) | — | 342.3(-22.3 to 4131.5) |  | -23.3(-30.2 to -15.7) | -23.9(-87.9 to 200.9) | -51.3(-92.3 to 92.7) |
| **Madagascar** | 185259(154974 to 221827) | 40(6 to 129) | 6239(939 to 20054) |  | 2879.8(2586.9 to 3195.6) | — | 81995.7(13893.9 to 840014.0) |  | -17.5(-24.6 to -10.0) | 163.8(-39.8 to 723.4) | 75.5(-60.0 to 447.5) |
| **Malawi** | 23027143(19536135 to 26983125) | 3584(683 to 10525) | 555584(105910 to 1631621) |  | -2.0(-16.0 to 12.3) | — | 2445.0(399.3 to 30095.8) |  | -48.0(-53.2 to -42.0) | 63.1(-68.8 to 488.7) | 8.5(-79.2 to 291.5) |
| **Malaysia** | 796098(678228 to 933415) | 738(174 to 2074) | 8088(1910 to 22741) |  | 65.7(53.4 to 78.6) | — | 2599.7(357.2 to 27268.2) |  | -16.2(-21.8 to -10.4) | 0.4(-82.0 to 424.7) | -35.1(-88.4 to 239.6) |
| **Maldives** | 37202(31334 to 44113) | 18(2 to 73) | 196(21 to 804) |  | 13.3(1.3 to 25.0) | — | 562.9(-41.3 to 10590.3) |  | -26.5(-32.3 to -19.8) | -48.6(-89.4 to 180.1) | -66.7(-93.1 to 81.1) |
| **Mali** | 2511260(2140441 to 2916187) | 537(70 to 1654) | 83227(10909 to 256280) |  | 89.3(67.8 to 112.2) | — | 1309.0(56.4 to 18138.4) |  | -34.3(-40.3 to -26.7) | -3.8(-78.4 to 366.4) | -36.0(-85.6 to 210.2) |
| **Malta** | 29448(24434 to 35492) | 35(5 to 131) | 280(40 to 1040) |  | -53.7(-57.6 to -49.8) | — | 335.8(-39.1 to 5348.0) |  | -15.5(-21.7 to -8.2) | -65.2(-93.5 to 23.6) | -77.7(-95.9 to -20.8) |
| **Marshall Islands** | 185841(163134 to 209069) | 219(38 to 678) | 2400(416 to 7433) |  | 46.8(37.2 to 57.0) | — | 5230.8(903.6 to 70787.8) |  | 12.9(5.0 to 21.0) | -21.9(-87.4 to 252.8) | -49.5(-91.9 to 128.4) |
| **Mauritania** | 842224(714596 to 985567) | 154(19 to 491) | 23798(2895 to 76065) |  | -66.5(-70.2 to -62.3) | — | 190.3(-70.9 to 3090.2) |  | -38.0(-43.1 to -32.2) | -20.1(-80.5 to 198.2) | -46.8(-87.0 to 98.4) |
| **Mauritius** | 89568(77465 to 104421) | 49(11 to 151) | 532(119 to 1657) |  | 85.2(70.2 to 101.2) | — | 2709.7(383.8 to 29400.2) |  | -9.1(-14.7 to -2.8) | -40.6(-84.7 to 128.0) | -61.5(-90.1 to 47.5) |
| **Mexico** | 168562(140563 to 198918) | 509(96 to 1577) | 6450(1211 to 19957) |  | -2.8(-9.5 to 3.7) | — | 4662.1(1002.9 to 43447.5) |  | -7.8(-15.9 to -1.9) | 63.1(-63.7 to 413.5) | 5.9(-76.4 to 233.6) |
| **Micronesia (Federated States of)** | 808363(693939 to 922085) | 262(15 to 1310) | 2877(167 to 14359) |  | 164.0(148.1 to 181.2) | — | 680.5(-43.5 to 10204.7) |  | 62.0(51.8 to 72.2) | 175.1(-49.8 to 1415.8) | 78.0(-67.5 to 880.4) |
| **Monaco** | 109233(94070 to 126955) | 207(23 to 787) | 1649(186 to 6255) |  | -25.9(-31.3 to -20.9) | — | 134.2(-75.4 to 2384.7) |  | -14.7(-20.0 to -9.2) | 29.3(-75.3 to 569.9) | -17.3(-84.2 to 328.9) |
| **Mongolia** | 14358(10174 to 17923) | 436(84 to 1241) | 1991(382 to 5662) |  | 4529.3(0.0 to 0.0) | — | 234230.3(0.0 to 0.0) |  | 22.7(-8.9 to 43.8) | 202.2(-20.4 to 700.2) | 87.8(-50.6 to 396.5) |
| **Montenegro** | 6413(5428 to 7622) | 16(3 to 54) | 74(12 to 247) |  | 3.5(-5.5 to 13.3) | — | 608.3(43.2 to 6800.7) |  | -30.3(-35.2 to -25.3) | -23.9(-87.0 to 189.8) | -52.7(-91.9 to 80.1) |
| **Morocco** | 188459(158801 to 224699) | 169(51 to 404) | 4778(1433 to 11459) |  | 51.9(36.5 to 70.3) | — | 2834.0(474.3 to 35162.2) |  | -41.4(-46.0 to -35.9) | 9.9(-74.5 to 385.6) | -27.6(-83.2 to 220.0) |
| **Mozambique** | 31645990(26501118 to 37319667) | 13030(4217 to 28805) | 2019649(653686 to 4466062) |  | 221.7(186.0 to 263.5) | — | 4808.5(1213.2 to 36837.0) |  | -20.0(-28.2 to -11.2) | 53.3(-54.9 to 270.5) | 2.0(-70.0 to 146.5) |
| **Myanmar** | 2254430(1948407 to 2557790) | 15394(5349 to 31622) | 168831(58654 to 346756) |  | 1051.7(975.9 to 1134.9) | — | 37417.3(7638.5 to 405082.2) |  | -62.5(-66.4 to -59.2) | -29.0(-76.0 to 64.8) | -54.0(-84.5 to 6.6) |
| **Namibia** | 27483341(23041223 to 32563712) | 13170(5141 to 25436) | 2041296(797200 to 3942730) |  | 145.8(111.1 to 182.1) | — | 18426.2(3427.7 to 172190.5) |  | -39.7(-46.5 to -32.1) | 4.9(-73.4 to 310.4) | -30.2(-82.3 to 172.9) |
| **Nauru** | 300784(256293 to 351428) | 96(10 to 416) | 1053(108 to 4557) |  | 287.9(260.6 to 317.7) | — | 1148.6(-20.9 to 15710.3) |  | 29.4(19.6 to 40.3) | 130.6(-60.3 to 1296.3) | 49.2(-74.3 to 803.8) |
| **Nepal** | 243952(205433 to 287182) | 367(90 to 989) | 14969(3673 to 40346) |  | 0.0(0.0 to 0.0) | — | 0.0(0.0 to 0.0) |  | -45.3(-52.1 to -38.9) | 6.8(-74.3 to 178.5) | -29.4(-83.0 to 84.3) |
| **Netherlands** | 28706(24634 to 33346) | 86(33 to 189) | 686(266 to 1507) |  | -77.4(-79.0 to -75.7) | — | 0.6(-70.9 to 448.2) |  | -31.6(-36.1 to -26.8) | -0.2(-63.0 to 143.1) | -36.2(-76.3 to 55.5) |
| **New Zealand** | 25018(20973 to 29746) | 70(16 to 201) | 556(124 to 1597) |  | -79.0(-80.7 to -77.0) | — | -45.7(-85.0 to 222.4) |  | -15.5(-21.9 to -9.2) | 20.3(-72.3 to 244.5) | -23.0(-82.3 to 120.6) |
| **Nicaragua** | 76522(65131 to 88602) | 139(23 to 393) | 1760(297 to 4975) |  | 28.8(15.2 to 42.8) | — | 643.2(30.0 to 5975.7) |  | -11.8(-18.4 to -5.3) | 53.0(-64.7 to 364.9) | -0.6(-77.1 to 202.0) |
| **Niger** | 1506190(1287335 to 1749969) | 292(41 to 930) | 45201(6403 to 144138) |  | 49.7(30.2 to 68.2) | — | 1047.9(23.9 to 16925.1) |  | -37.5(-43.1 to -30.5) | -20.1(-80.8 to 278.5) | -46.9(-87.2 to 151.7) |
| **Nigeria** | 4303517(3613581 to 5047093) | 1457(311 to 3910) | 225806(48189 to 606006) |  | 78.0(65.3 to 92.3) | — | 8684.9(1910.1 to 98687.8) |  | -30.4(-36.7 to -26.2) | 6.7(-76.2 to 201.9) | -29.1(-84.1 to 100.8) |
| **Niue** | 211241(183298 to 245312) | 62(7 to 281) | 685(75 to 3086) |  | 204.7(184.8 to 224.6) | — | 928.6(-19.6 to 11056.8) |  | 45.2(37.2 to 53.8) | 147.1(-54.8 to 1556.1) | 59.9(-70.7 to 969.7) |
| **North Macedonia** | 6417(5416 to 7625) | 23(8 to 57) | 107(35 to 260) |  | -25.6(-32.6 to -18.3) | — | 712.9(86.9 to 7713.3) |  | -26.4(-31.4 to -20.7) | -32.1(-81.3 to 97.1) | -57.8(-88.4 to 22.4) |
| **Northern Mariana Islands** | 56452(47611 to 66867) | 68(9 to 247) | 744(102 to 2713) |  | -18.6(-26.0 to -9.4) | — | 215.1(-55.6 to 4893.3) |  | -19.3(-26.8 to -10.9) | 12.8(-81.5 to 338.4) | -27.0(-88.0 to 183.3) |
| **Norway** | 19105(15944 to 23044) | 79(20 to 225) | 624(156 to 1791) |  | -48.4(-53.7 to -42.9) | — | 10.8(-70.0 to 452.4) |  | -43.3(-47.2 to -39.9) | -19.7(-78.7 to 131.9) | -48.6(-86.4 to 48.4) |
| **Oman** | 26853(22006 to 32625) | 22(5 to 64) | 633(132 to 1805) |  | 3.6(-6.5 to 15.5) | — | 116.3(-40.6 to 1182.4) |  | -47.6(-51.8 to -43.0) | -11.3(-81.8 to 191.5) | -41.5(-88.0 to 92.0) |
| **Pakistan** | 149638(124822 to 177492) | 265(81 to 626) | 10805(3313 to 25530) |  | -8.3(-15.1 to -1.1) | — | 7730.8(1611.1 to 80262.4) |  | 27.0(11.3 to 40.7) | 158.8(-40.1 to 931.3) | 71.1(-60.4 to 582.2) |
| **Palau** | 220407(186824 to 260780) | 74(7 to 327) | 812(79 to 3588) |  | 249.2(221.5 to 278.2) | — | 1110.6(-23.5 to 14176.5) |  | 66.9(55.5 to 79.1) | 194.4(-52.4 to 1814.3) | 90.5(-69.2 to 1140.2) |
| **Palestine** | 3615(2971 to 4304) | 3(0 to 14) | 98(12 to 400) |  | -35.6(-42.6 to -28.4) | — | 539.0(-34.9 to 7511.7) |  | -16.6(-24.1 to -8.8) | 52.1(-70.9 to 709.6) | 0.3(-80.8 to 433.6) |
| **Panama** | 511250(437234 to 590345) | 1161(145 to 4426) | 14698(1835 to 56085) |  | 9.7(-1.7 to 20.6) | — | 1679.7(94.0 to 18348.7) |  | -17.2(-24.4 to -8.7) | 17.0(-74.8 to 443.6) | -24.0(-83.6 to 253.2) |
| **Papua New Guinea** | 1433307(1251726 to 1622635) | 6344(2306 to 14562) | 69583(25294 to 159671) |  | 2859.9(2705.2 to 3025.8) | — | 97698.5(20198.2 to 991871.3) |  | -21.1(-27.2 to -15.8) | 278.7(-8.1 to 1347.2) | 145.0(-40.5 to 836.6) |
| **Paraguay** | 480549(398054 to 572722) | 1622(183 to 5312) | 20534(2321 to 67252) |  | 92.3(74.8 to 112.3) | — | 1999.8(274.7 to 19841.9) |  | -17.7(-26.9 to -9.3) | 85.0(-71.2 to 473.0) | 20.2(-81.3 to 272.2) |
| **Peru** | 398832(337316 to 468801) | 3178(1559 to 5717) | 40244(19738 to 72356) |  | -36.0(-44.0 to -27.5) | — | 564.9(128.7 to 2840.7) |  | -32.0(-39.3 to -25.0) | -12.5(-55.3 to 62.0) | -43.2(-71.0 to 5.3) |
| **Philippines** | 53903(46850 to 61580) | 177(49 to 407) | 1939(535 to 4459) |  | 0.0(0.0 to 0.0) | — | 0.0(0.0 to 0.0) |  | 47.1(35.5 to 58.8) | 121.8(-46.7 to 588.3) | 43.5(-65.5 to 345.1) |
| **Poland** | 20190(16855 to 24483) | 44(16 to 102) | 200(72 to 467) |  | 64.2(50.8 to 80.0) | — | 262.5(20.7 to 1504.0) |  | -33.8(-37.3 to -30.1) | -6.6(-68.7 to 141.6) | -42.0(-80.6 to 50.0) |
| **Portugal** | 186401(160915 to 215893) | 417(93 to 1115) | 3318(740 to 8867) |  | -15.6(-23.2 to -8.0) | — | 23.2(-56.4 to 322.1) |  | -35.8(-40.5 to -30.8) | -7.3(-77.4 to 161.7) | -40.7(-85.5 to 67.4) |
| **Puerto Rico** | 60489(52011 to 70338) | 79(16 to 239) | 997(198 to 3027) |  | -71.1(-74.0 to -67.2) | — | 246.3(-20.3 to 2753.2) |  | -39.4(-44.0 to -34.6) | -42.0(-86.5 to 85.3) | -62.3(-91.2 to 20.4) |
| **Qatar** | 2851(2308 to 3512) | 3(1 to 9) | 73(15 to 249) |  | -65.2(-68.9 to -61.0) | — | 174.7(-46.9 to 2984.5) |  | -36.8(-42.5 to -31.0) | 29.9(-71.3 to 368.7) | -14.4(-81.1 to 208.7) |
| **Republic of Korea** | 81281(69213 to 95853) | 183(26 to 722) | 1454(203 to 5746) |  | -6.2(-28.9 to 0.0) | — | 214.5(-46.4 to 1650.0) |  | -29.4(-35.3 to -23.4) | -7.7(-83.4 to 217.1) | -40.9(-89.4 to 102.6) |
| **Republic of Moldova** | 79507(45370 to 113857) | 18071(11833 to 25974) | 82473(54012 to 118583) |  | 30.4(9.8 to 52.9) | — | 6287.0(1574.6 to 57040.8) |  | -49.1(-69.3 to -31.1) | 19.0(-20.4 to 67.1) | -26.1(-50.6 to 3.9) |
| **Romania** | 96468(81741 to 114225) | 901(364 to 1855) | 4111(1663 to 8464) |  | 40.9(24.9 to 57.2) | — | 477.3(55.9 to 2159.4) |  | 5.3(-5.1 to 17.1) | 63.9(-59.5 to 545.9) | 1.8(-74.9 to 301.4) |
| **Russian Federation** | 262863(186930 to 352537) | 29173(14751 to 46715) | 133147(67318 to 213240) |  | 84.3(43.9 to 122.8) | — | 7085.8(1906.0 to 31031.4) |  | -34.6(-48.8 to -16.3) | 2.0(-39.6 to 72.2) | -36.6(-62.5 to 7.0) |
| **Rwanda** | 6462327(5483222 to 7628793) | 1534(587 to 3290) | 237778(91006 to 509971) |  | -2.2(-12.6 to 10.3) | — | 5631.0(1057.9 to 56900.7) |  | -44.5(-49.1 to -39.1) | 13.2(-71.0 to 400.0) | -24.7(-80.7 to 232.6) |
| **Saint Kitts and Nevis** | 747095(626975 to 893172) | 395(41 to 1804) | 4998(514 to 22843) |  | 83.1(63.6 to 106.3) | — | 40.4(-90.1 to 1377.3) |  | 1.6(-6.8 to 10.1) | 151.2(-56.2 to 1243.7) | 63.2(-71.5 to 774.3) |
| **Saint Lucia** | 122921(104695 to 144929) | 54(7 to 220) | 687(83 to 2786) |  | -59.0(-63.5 to -54.1) | — | -83.5(-98.7 to 149.7) |  | -24.5(-29.7 to -18.9) | 52.7(-71.6 to 648.4) | -0.8(-81.6 to 386.7) |
| **Saint Vincent and the Grenadines** | 344439(294454 to 403586) | 196(21 to 742) | 2484(267 to 9401) |  | -31.3(-38.5 to -22.5) | — | -75.9(-97.9 to 346.5) |  | -22.1(-27.5 to -16.2) | 68.7(-68.3 to 789.7) | 9.6(-79.4 to 477.2) |
| **Samoa** | 256932(220755 to 293990) | 19(2 to 68) | 205(22 to 742) |  | 89.5(78.3 to 101.7) | — | 446.8(-52.7 to 6336.5) |  | 34.5(26.9 to 42.5) | 36.2(-74.5 to 707.1) | -11.9(-83.5 to 422.6) |
| **San Marino** | 43855(37488 to 51191) | 76(10 to 273) | 604(81 to 2168) |  | -27.1(-32.1 to -22.5) | — | 115.3(-77.3 to 2733.6) |  | -20.9(-26.1 to -15.4) | 14.3(-80.0 to 432.8) | -26.9(-87.2 to 241.0) |
| **Sao Tome and Principe** | 523940(435941 to 617928) | 156(19 to 461) | 24133(3004 to 71392) |  | -45.9(-53.0 to -38.8) | — | 454.3(-39.2 to 6849.6) |  | -37.3(-43.4 to -31.0) | 3.9(-78.8 to 366.2) | -30.9(-85.9 to 210.2) |
| **Saudi Arabia** | 93025(75704 to 113221) | 116(21 to 349) | 3286(587 to 9884) |  | -4.3(-18.4 to 12.1) | — | 2413.7(482.4 to 25750.8) |  | -39.4(-45.1 to -33.5) | 2.5(-79.0 to 206.4) | -32.5(-86.2 to 101.9) |
| **Senegal** | 1296776(1116555 to 1517444) | 233(68 to 527) | 36075(10485 to 81583) |  | 64.7(48.7 to 82.8) | — | 4820.6(788.6 to 56662.2) |  | -31.2(-36.7 to -24.6) | 4.9(-78.6 to 357.0) | -30.2(-85.8 to 204.0) |
| **Serbia** | 59921(51190 to 70521) | 140(39 to 376) | 641(180 to 1716) |  | 16.9(-22.7 to 0.0) | — | 460.5(0.0 to 3009.4) |  | -41.6(-45.6 to -37.0) | -26.1(-80.2 to 99.3) | -54.1(-87.7 to 24.0) |
| **Seychelles** | 126715(109639 to 147357) | 32(2 to 158) | 346(24 to 1731) |  | 61.1(49.3 to 73.3) | — | 1059.4(-22.8 to 16021.1) |  | -14.9(-19.9 to -9.5) | -16.6(-89.9 to 420.8) | -46.0(-93.4 to 236.9) |
| **Sierra Leone** | 2465098(2108265 to 2872408) | 440(60 to 1434) | 68197(9283 to 222362) |  | 260.4(221.9 to 301.4) | — | 1277.6(59.0 to 11286.5) |  | -27.6(-34.1 to -20.3) | 8.4(-74.7 to 343.0) | -27.9(-83.2 to 194.5) |
| **Singapore** | 88608(76011 to 103626) | 155(67 to 324) | 1233(532 to 2574) |  | -5.2(-15.8 to 6.2) | — | 208.9(-8.6 to 1537.0) |  | -14.5(-20.1 to -8.2) | 12.9(-55.7 to 144.9) | -27.8(-71.6 to 56.7) |
| **Slovakia** | 928(786 to 1097) | 2(0 to 6) | 8(2 to 28) |  | -32.8(-41.5 to 0.0) | — | 60.9(-53.7 to 746.4) |  | -33.5(-37.8 to -28.7) | -35.2(-85.9 to 104.6) | -59.8(-91.2 to 27.0) |
| **Slovenia** | 4042(3485 to 4744) | 1(0 to 4) | 5(1 to 20) |  | -71.9(-75.0 to 0.0) | — | -73.4(-95.2 to 85.1) |  | -10.2(-16.5 to -3.9) | -59.7(-92.4 to 44.6) | -74.9(-95.3 to -10.2) |
| **Solomon Islands** | 189527(165026 to 215984) | 66(5 to 261) | 719(54 to 2858) |  | 74.1(64.3 to 84.6) | — | 522.0(-53.9 to 9329.1) |  | 26.2(18.2 to 34.9) | 122.4(-67.8 to 1293.8) | 43.9(-79.2 to 801.4) |
| **Somalia** | 1763649(1406905 to 2158152) | 1009(278 to 2700) | 156366(43095 to 418612) |  | 634.9(575.9 to 694.8) | — | 113236.0(28693.9 to 1109852.0) |  | -36.2(-47.1 to -28.6) | -5.4(-74.5 to 174.1) | -37.0(-83.1 to 82.3) |
| **South Africa** | 33354798(29098790 to 38149878) | 6792(2393 to 16007) | 1052689(371005 to 2481506) |  | 591.7(529.6 to 648.1) | — | 4614.7(1120.9 to 28825.6) |  | -37.5(-41.9 to -33.1) | -18.2(-78.5 to 187.5) | -45.6(-85.7 to 91.3) |
| **South Sudan** | 3491550(2908394 to 4096969) | 1127(183 to 3585) | 174643(28381 to 555736) |  | 236.9(198.2 to 272.4) | — | 13579.4(1561.5 to 166563.1) |  | -23.8(-31.5 to -15.0) | 44.7(-66.0 to 557.9) | -3.7(-77.4 to 337.5) |
| **Spain** | 48638(41747 to 56926) | 89(11 to 329) | 707(86 to 2614) |  | -78.1(-79.9 to -76.1) | — | -54.3(-92.2 to 198.9) |  | -40.8(-45.6 to -36.1) | -28.0(-84.8 to 155.8) | -53.9(-90.3 to 63.7) |
| **Sri Lanka** | 21933(18760 to 25741) | 9(1 to 36) | 101(10 to 395) |  | 19.7(10.6 to 29.5) | — | 893.9(-3.9 to 13916.9) |  | -7.6(-12.9 to -1.9) | 6.3(-80.7 to 452.9) | -31.3(-87.5 to 257.7) |
| **Sudan** | 232037(195614 to 273741) | 208(24 to 834) | 5889(665 to 23611) |  | 145.9(120.3 to 173.8) | — | 2466.0(145.3 to 33439.3) |  | -27.6(-33.8 to -20.5) | 15.9(-74.9 to 514.1) | -23.6(-83.5 to 304.8) |
| **Suriname** | 378648(322552 to 445994) | 141(17 to 508) | 1780(214 to 6432) |  | -26.0(-33.2 to -17.3) | — | -39.7(-94.8 to 555.4) |  | -24.5(-29.7 to -18.2) | 52.0(-73.5 to 774.0) | -1.3(-82.8 to 468.0) |
| **Sweden** | 24831(20611 to 29970) | 131(42 to 329) | 1043(333 to 2622) |  | -56.2(-59.5 to -52.7) | — | 141.2(-35.2 to 1412.7) |  | -10.3(-17.7 to -3.8) | 22.1(-61.7 to 202.0) | -21.8(-75.5 to 93.3) |
| **Switzerland** | 19169(16129 to 22456) | 124(48 to 265) | 986(384 to 2109) |  | 78.7(66.2 to 91.8) | — | 646.2(98.8 to 4535.8) |  | -33.1(-38.8 to -27.7) | 95.0(-31.2 to 348.5) | 24.8(-56.0 to 186.9) |
| **Syrian Arab Republic** | 5118(4269 to 6104) | 6(1 to 25) | 172(24 to 708) |  | -15.2(-25.9 to -4.0) | — | 1189.2(70.8 to 17809.5) |  | -17.8(-27.5 to -9.0) | 8.2(-77.2 to 318.6) | -28.7(-85.0 to 176.1) |
| **Taiwan (Province of China)** | 70090(60203 to 80463) | 168(19 to 626) | 1839(211 to 6869) |  | 213.5(162.4 to 245.3) | — | 816.3(-16.0 to 11987.7) |  | -3.5(-13.6 to 8.6) | 36.3(-73.3 to 645.8) | -11.8(-82.8 to 381.9) |
| **Tajikistan** | 42491(32302 to 53563) | 2055(905 to 3743) | 9379(4130 to 17096) |  | 45.1(20.1 to 66.8) | — | 36227.8(7585.8 to 338275.2) |  | -31.8(-49.2 to -12.6) | 24.2(-60.2 to 273.3) | -22.8(-75.2 to 132.0) |
| **Thailand** | 1900399(1624249 to 2244005) | 5230(1434 to 13078) | 57356(15720 to 143443) |  | -35.4(-41.6 to -29.3) | — | 168.3(-23.2 to 1617.0) |  | -12.1(-18.8 to -5.2) | 29.8(-68.0 to 259.7) | -16.0(-79.3 to 132.7) |
| **Timor-Leste** | 124420(109209 to 139978) | 68(7 to 293) | 743(78 to 3218) |  | 283.4(253.7 to 312.5) | — | 2008.1(83.3 to 29393.0) |  | -12.4(-17.5 to -6.2) | -31.1(-87.2 to 279.4) | -55.4(-91.7 to 145.6) |
| **Togo** | 3097922(2628709 to 3613819) | 606(93 to 1779) | 93884(14445 to 275742) |  | 185.3(151.1 to 220.2) | — | 1883.3(163.7 to 26048.9) |  | -49.5(-54.2 to -44.0) | -32.0(-84.6 to 209.4) | -54.8(-89.7 to 105.8) |
| **Tokelau** | 217650(189600 to 248461) | 74(6 to 379) | 808(65 to 4163) |  | 179.8(161.2 to 197.3) | — | 866.5(-19.7 to 14981.6) |  | 32.1(24.1 to 40.4) | 142.7(-57.8 to 1184.6) | 57.0(-72.7 to 731.5) |
| **Tonga** | 53587(46536 to 60702) | 18(2 to 74) | 194(19 to 815) |  | 51.7(43.1 to 60.9) | — | 371.5(-60.8 to 6759.3) |  | -19.1(-23.6 to -14.4) | 51.2(-75.2 to 672.7) | -2.2(-84.0 to 400.0) |
| **Trinidad and Tobago** | 272679(234158 to 316226) | 143(17 to 610) | 1816(216 to 7723) |  | -11.2(-20.0 to -1.1) | — | -24.9(-92.7 to 721.1) |  | -19.1(-25.8 to -12.4) | 69.9(-68.0 to 840.0) | 10.4(-79.2 to 510.2) |
| **Tunisia** | 33615(28714 to 39272) | 19(6 to 46) | 540(164 to 1311) |  | 52.0(38.3 to 66.6) | — | 2131.4(233.6 to 24697.3) |  | -7.0(-13.1 to -0.4) | 59.2(-60.3 to 584.4) | 4.9(-73.9 to 350.7) |
| **Turkey** | 10708(9142 to 12636) | 19(8 to 36) | 524(232 to 1034) |  | 35.9(-0.7 to 24.9) | — | 5115.9(0.0 to 34980.4) |  | -21.2(-27.6 to -14.9) | 34.2(-50.0 to 241.9) | -11.6(-67.0 to 125.4) |
| **Turkmenistan** | 99083(69154 to 127695) | 5670(2037 to 11473) | 25877(9301 to 52319) |  | -4.5(-21.4 to 11.2) | — | 19912.5(4474.7 to 201637.3) |  | -40.5(-59.2 to -21.4) | 18.8(-65.0 to 245.5) | -26.2(-78.3 to 114.5) |
| **Tuvalu** | 281981(248832 to 319693) | 94(8 to 435) | 1027(84 to 4780) |  | 191.0(174.7 to 208.2) | — | 891.0(-33.4 to 15273.4) |  | 37.3(29.4 to 44.7) | 145.3(-57.2 to 1493.3) | 58.7(-72.3 to 931.0) |
| **Uganda** | 14838478(13090770 to 16894162) | 3749(814 to 9734) | 581167(126225 to 1509022) |  | -25.1(-31.2 to -18.6) | — | 4379.0(953.5 to 47773.3) |  | -48.6(-53.2 to -43.1) | 14.2(-76.8 to 214.0) | -24.0(-84.6 to 108.8) |
| **Ukraine** | 424116(256087 to 593458) | 58214(30038 to 93286) | 265684(137052 to 425587) |  | 117.1(59.4 to 161.6) | — | 8753.6(2119.9 to 73401.6) |  | -34.8(-62.2 to 1.3) | 66.8(-30.2 to 308.2) | 3.6(-56.7 to 153.6) |
| **United Arab Emirates** | 100020(80624 to 124376) | 90(10 to 347) | 2561(284 to 9817) |  | 221.5(174.1 to 269.7) | — | 2698.1(181.7 to 32461.4) |  | 36.8(12.9 to 60.9) | 155.3(-57.2 to 1250.0) | 68.2(-71.8 to 789.7) |
| **United Kingdom** | 44321(36333 to 53529) | 83(28 to 200) | 658(225 to 1590) |  | -39.2(-42.6 to -35.2) | — | -8.9(-69.7 to 218.2) |  | -52.0(-54.1 to -50.0) | -28.0(-74.2 to 78.1) | -53.9(-83.5 to 14.0) |
| **United Republic of Tanzania** | 9259820(7786242 to 10874446) | 1814(334 to 5794) | 281207(51749 to 898685) |  | -18.3(-26.3 to -9.5) | — | 2442.0(343.2 to 35710.1) |  | -54.1(-58.3 to -49.6) | 20.4(-71.4 to 236.8) | -19.9(-81.0 to 124.0) |
| **United States of America** | 17194(14746 to 20082) | 37(14 to 81) | 293(108 to 643) |  | -80.3(-81.8 to -78.7) | — | -91.5(-95.3 to -83.5) |  | -39.0(-41.6 to -36.4) | 1.9(-61.3 to 119.4) | -34.8(-75.2 to 40.4) |
| **United States Virgin Islands** | 83510(71534 to 98232) | 39(5 to 145) | 488(57 to 1834) |  | -24.5(-32.1 to -15.5) | — | -43.6(-95.2 to 598.0) |  | -23.8(-28.8 to -19.1) | 62.0(-69.4 to 802.9) | 5.2(-80.1 to 486.4) |
| **Uruguay** | 314610(269632 to 370715) | 153(34 to 468) | 1219(269 to 3716) |  | 25.9(16.2 to 36.4) | — | 244.8(-47.1 to 3137.3) |  | 2.4(-4.4 to 9.7) | 84.1(-64.2 to 664.2) | 17.8(-77.1 to 389.2) |
| **Uzbekistan** | 52244(30128 to 71254) | 5161(1870 to 9879) | 23556(8536 to 45095) |  | -19.2(-32.7 to -5.4) | — | 45444.4(10752.4 to 511843.4) |  | -27.8(-58.1 to -0.9) | -3.7(-66.2 to 90.5) | -40.1(-79.0 to 18.4) |
| **Vanuatu** | 301958(267155 to 337120) | 61(5 to 260) | 674(56 to 2855) |  | 137.0(123.7 to 150.5) | — | 734.5(-46.6 to 8890.1) |  | 4.3(-1.1 to 10.6) | 151.7(-55.5 to 1556.9) | 62.9(-71.2 to 972.3) |
| **Venezuela (Bolivarian Republic of)** | 295720(251322 to 340789) | 547(66 to 1849) | 6920(833 to 23405) |  | -20.6(-29.4 to -11.1) | — | 922.2(9.7 to 13517.0) |  | 9.8(0.6 to 19.4) | 59.9(-68.3 to 632.3) | 3.9(-79.4 to 376.0) |
| **Viet Nam** | 1193097(1040634 to 1329342) | 5400(1587 to 13421) | 59228(17404 to 147234) |  | 67.9(57.1 to 79.0) | — | 1698.0(396.9 to 10024.3) |  | -23.6(-30.5 to -17.8) | 3.3(-73.3 to 183.2) | -33.2(-82.7 to 83.2) |
| **Yemen** | 87663(74258 to 103575) | 83(17 to 300) | 2354(478 to 8495) |  | -25.5(-33.4 to -16.5) | — | 1073.4(137.1 to 14173.4) |  | -16.3(-24.0 to -8.9) | 17.0(-73.8 to 303.5) | -22.9(-82.7 to 165.9) |
| **Zambia** | 26968586(22495850 to 32231363) | 7950(1243 to 27674) | 1232319(192527 to 4290397) |  | 1.0(-12.6 to 14.9) | — | 980.0(125.3 to 9712.2) |  | -41.1(-47.5 to -34.5) | 108.4(-57.4 to 532.0) | 38.7(-71.6 to 320.3) |
| **Zimbabwe** | 33622872(27601899 to 40234010) | 13240(1987 to 42770) | 2052166(308075 to 6626111) |  | 35.1(12.2 to 56.4) | — | 2051.3(110.2 to 27697.5) |  | -52.2(-58.5 to -44.4) | -23.7(-82.2 to 269.1) | -49.3(-88.1 to 145.6) |

| **S16** | | | | | | | | | | |
| --- | --- | --- | --- | --- | --- | --- | --- | --- | --- | --- |
| **Group** | **Number of DALYs (age-standardized, both sexes) with 95% uncertainty intervals (2019)** | | |  | **Annualized rate of change of age-standardized rate (%) with 95% uncertainty intervals** | | | | | |
|  |  |  |  |  | **1990-2010** | |  | **2010-2019** | | |
|  | **HIV/AIDS - Drug-susceptible Tuberculosis** | **HIV/AIDS - Extensively drug-resistant Tuberculosis** | **HIV/AIDS - Multidrug-resistant Tuberculosis without extensive drug resistance** |  | **HIV/AIDS - Drug-susceptible Tuberculosis** | **HIV/AIDS - Multidrug-resistant Tuberculosis without extensive drug resistance** |  | **HIV/AIDS - Drug-susceptible Tuberculosis** | **HIV/AIDS - Extensively drug-resistant Tuberculosis** | **HIV/AIDS - Multidrug-resistant Tuberculosis without extensive drug resistance** |
| **Global** | 13697119(10071811 to 17569090) | 52003(22943 to 93846) | 1341461(582207 to 2456710) |  | 116.5(73.1 to 174.5) | 3244.3(1842.2 to 5372.4) |  | -53.0(-55.9 to -49.9) | -21.2(-36.0 to -1.0) | -45.1(-61.1 to -23.1) |
| **Afghanistan** | 63763(28005 to 112091) | 555(63 to 1996) | 7583(952 to 26320) |  | -37.5(-55.5 to 168.0) | 3204.2(560.8 to 57085.2) |  | -43.1(-66.6 to -24.0) | -44.1(-89.0 to 69.7) | -63.0(-92.7 to 12.6) |
| **Albania** | 64786(39428 to 104550) | 524(74 to 1783) | 1180(166 to 3966) |  | -44.2(-66.4 to -8.4) | 274.0(-28.1 to 3066.9) |  | 13.8(2.6 to 27.8) | -9.3(-82.1 to 188.4) | -43.8(-89.0 to 77.7) |
| **Algeria** | 111259(63672 to 169450) | 415(32 to 1882) | 5823(451 to 25655) |  | -21.7(-40.0 to 8.0) | 0.5(-86.1 to 454.4) |  | -42.0(-51.7 to -28.8) | 7.4(-80.1 to 295.3) | -28.9(-87.0 to 160.8) |
| **American Samoa** | 158314(107323 to 231842) | 217(16 to 960) | 1194(90 to 5108) |  | 4.1(-12.7 to 76.3) | 222.9(-79.9 to 4583.6) |  | -22.0(-29.6 to -13.2) | 35.7(-75.4 to 701.0) | -11.5(-84.1 to 416.4) |
| **Andorra** | 263395(169041 to 392074) | 408(38 to 1916) | 1586(141 to 7835) |  | -39.9(-49.6 to -25.7) | -10.9(-92.1 to 832.4) |  | -3.6(-16.8 to 8.7) | 15.4(-82.4 to 570.9) | -26.3(-88.8 to 327.7) |
| **Angola** | 58716267(36141227 to 91695351) | 62747(6208 to 224735) | 4673617(483365 to 16316184) |  | 1351.1(491.2 to 2991.1) | 14204.4(1298.2 to 210335.0) |  | -41.0(-51.9 to -24.7) | -4.5(-78.4 to 330.5) | -36.1(-85.6 to 187.8) |
| **Antigua and Barbuda** | 596972(419072 to 820779) | 1231(91 to 5318) | 8198(626 to 34182) |  | -64.1(-68.6 to -57.0) | -81.6(-98.2 to 110.1) |  | -29.2(-38.7 to -19.4) | 60.9(-69.6 to 964.5) | 2.4(-80.7 to 578.4) |
| **Argentina** | 7603943(5856630 to 8260572) | 67195(7917 to 255194) | 259901(29394 to 949541) |  | 92.6(62.5 to 106.5) | 919.1(106.7 to 7933.3) |  | -11.5(-24.1 to -3.8) | 0.5(-79.6 to 187.3) | -35.3(-86.9 to 83.8) |
| **Armenia** | 611605(258459 to 995019) | 103940(23849 to 232313) | 234848(58663 to 513874) |  | 346.0(192.5 to 682.1) | 88007.4(19552.8 to 1093095.9) |  | 16.1(-45.1 to 87.3) | 68.2(-48.7 to 241.2) | 3.0(-68.5 to 110.3) |
| **Australia** | 66417(38720 to 109587) | 1414(355 to 3811) | 5460(1382 to 14170) |  | -79.8(-83.0 to -76.2) | -14.5(-80.5 to 417.3) |  | -15.1(-31.2 to 2.5) | 132.6(-34.6 to 679.6) | 48.7(-58.2 to 396.9) |
| **Austria** | 179033(112011 to 282974) | 3718(951 to 9288) | 14360(3776 to 34928) |  | -83.4(-85.6 to -80.7) | 37.2(-62.6 to 1135.7) |  | -21.9(-32.5 to -10.9) | -13.0(-66.5 to 86.4) | -44.3(-78.7 to 19.1) |
| **Azerbaijan** | 335265(151968 to 580630) | 70599(18957 to 143066) | 157692(47714 to 315261) |  | -1.5(-51.9 to 192.8) | 25627.6(5069.5 to 436771.6) |  | -38.8(-69.8 to 7.4) | 11.6(-64.3 to 168.9) | -30.7(-77.8 to 66.6) |
| **Bahamas** | 4081835(2603343 to 6258787) | 25753(2515 to 99832) | 158507(17738 to 577364) |  | -53.1(-58.7 to -47.1) | 435.8(-4.7 to 5537.0) |  | -18.8(-30.5 to -8.5) | 76.6(-74.6 to 709.6) | 14.7(-83.6 to 424.5) |
| **Bahrain** | 137854(91361 to 209391) | 726(95 to 2339) | 10199(1440 to 32226) |  | -66.3(-70.4 to -61.6) | 613.3(54.4 to 8000.3) |  | -28.9(-41.3 to -18.1) | 14.0(-82.4 to 306.6) | -25.2(-88.5 to 167.0) |
| **Bangladesh** | 413623(86816 to 1616935) | 2040(77 to 12236) | 41517(2254 to 263517) |  | — | — |  | -9.2(-49.2 to 57.8) | 92.7(-67.6 to 484.1) | 25.3(-76.7 to 273.4) |
| **Barbados** | 679340(455044 to 980341) | 919(52 to 4626) | 5716(322 to 28611) |  | -66.5(-70.5 to -61.2) | -92.2(-99.5 to 40.9) |  | -22.3(-30.2 to -13.6) | 81.7(-77.3 to 1305.8) | 15.8(-85.4 to 796.8) |
| **Belarus** | 286894(119700 to 570138) | 196149(98888 to 332986) | 444118(246515 to 713609) |  | 38.0(-17.4 to 104.4) | 5978.9(1201.2 to 56213.0) |  | -68.9(-83.7 to -52.2) | -40.5(-54.3 to -22.3) | -62.5(-71.4 to -51.3) |
| **Belgium** | 264096(163379 to 406427) | 3072(614 to 8861) | 11770(2584 to 34353) |  | -60.5(-64.5 to -55.6) | -9.2(-72.4 to 430.5) |  | -42.8(-50.4 to -34.6) | -30.0(-77.3 to 72.9) | -55.3(-85.4 to 10.7) |
| **Belize** | 7769911(4936481 to 11660997) | 15136(1222 to 66487) | 93102(7720 to 398322) |  | -43.7(-49.3 to -37.2) | -62.2(-96.1 to 305.0) |  | -31.0(-37.4 to -23.9) | 62.0(-69.2 to 832.4) | 5.5(-80.0 to 506.2) |
| **Benin** | 25878396(15805423 to 38830247) | 18346(2348 to 58995) | 1362801(200503 to 4179924) |  | 1052.0(489.2 to 2050.0) | 10982.1(2119.9 to 107279.8) |  | -28.3(-38.5 to -19.0) | 34.0(-73.5 to 289.5) | -11.1(-82.3 to 159.8) |
| **Bermuda** | 299493(212584 to 409531) | 344(16 to 1949) | 2682(144 to 15553) |  | -66.8(-73.4 to -57.9) | -86.8(-99.2 to 73.6) |  | -29.2(-43.3 to -12.4) | 170.9(-69.6 to 1884.5) | 69.5(-81.1 to 1120.2) |
| **Bhutan** | 523268(171515 to 1018745) | 3091(247 to 12243) | 60372(4771 to 212415) |  | -23.7(-69.5 to 366.7) | 857.3(-15.6 to 32522.3) |  | -27.3(-48.2 to 13.9) | 14.2(-70.7 to 419.1) | -24.6(-80.6 to 245.5) |
| **Bolivia (Plurinational State of)** | 2068106(733000 to 3830692) | 41809(5548 to 136390) | 257676(34907 to 786799) |  | -32.0(-72.0 to 235.3) | 203.5(-68.2 to 7097.0) |  | -53.0(-71.7 to -35.2) | 10.9(-70.4 to 384.8) | -26.8(-80.6 to 216.7) |
| **Bosnia and Herzegovina** | 79370(58040 to 106963) | 399(70 to 1224) | 884(166 to 2627) |  | -43.1(-55.5 to -28.9) | 17.8(-73.1 to 684.1) |  | -14.6(-30.2 to 6.7) | -0.1(-72.5 to 185.2) | -38.0(-82.9 to 76.9) |
| **Botswana** | 292772681(166709085 to 447103505) | 498311(67638 to 1519768) | 37193754(5380477 to 116075186) |  | 125.7(29.1 to 272.1) | 3116.9(729.7 to 25871.6) |  | -54.6(-64.7 to -44.4) | 0.6(-75.6 to 159.9) | -32.6(-83.7 to 72.9) |
| **Brazil** | 3664317(2328856 to 5485159) | 43301(6445 to 136959) | 265762(39383 to 837387) |  | -46.5(-53.0 to -35.8) | 2407.4(451.9 to 27869.8) |  | -32.4(-41.1 to -26.2) | 36.5(-66.9 to 226.0) | -11.0(-78.5 to 112.7) |
| **Brunei Darussalam** | 365709(235102 to 553052) | 814(82 to 3346) | 3099(339 to 11735) |  | -22.2(-34.0 to -7.4) | 27.1(-83.6 to 1341.1) |  | 17.8(7.0 to 29.3) | 184.8(-58.6 to 1478.4) | 81.4(-73.5 to 906.7) |
| **Bulgaria** | 32066(20689 to 48384) | 796(172 to 2130) | 1786(423 to 5043) |  | 4.5(-11.3 to 21.5) | 1636.9(317.5 to 15874.1) |  | -30.5(-41.1 to -19.1) | -46.5(-85.2 to 28.6) | -66.5(-90.8 to -20.1) |
| **Burkina Faso** | 34772545(25690562 to 43589241) | 36697(4433 to 117524) | 2762647(338100 to 8850294) |  | -67.6(-75.9 to -59.2) | 191.4(-57.2 to 3644.9) |  | -63.4(-69.6 to -54.6) | -49.2(-88.7 to 129.6) | -65.9(-92.5 to 53.0) |
| **Burundi** | 49507666(36565665 to 61143055) | 55752(5976 to 196999) | 4199699(526656 to 13749352) |  | -25.7(-64.2 to 71.1) | 2074.1(132.0 to 33950.0) |  | -78.8(-83.6 to -70.7) | -58.4(-89.4 to 65.6) | -72.0(-92.9 to 11.4) |
| **Cabo Verde** | 5450838(3358491 to 8761760) | 6777(621 to 24822) | 502963(50696 to 1700064) |  | -33.8(-52.9 to 19.1) | 486.4(-23.9 to 7378.4) |  | -53.8(-61.6 to -42.8) | -26.4(-82.4 to 228.6) | -51.4(-88.3 to 118.2) |
| **Cambodia** | 14344093(9667560 to 19938617) | 51094(5332 to 198568) | 272830(28332 to 1026963) |  | 12054.0(5862.8 to 21883.6) | 253313.0(46759.4 to 3338260.7) |  | -66.5(-71.5 to -60.5) | -71.2(-94.6 to -7.7) | -81.2(-96.4 to -40.0) |
| **Cameroon** | 91396878(51374164 to 145963481) | 77868(8174 to 294223) | 5766099(670558 to 20811234) |  | 515.8(206.5 to 1096.6) | 4456.0(344.4 to 56170.8) |  | -56.8(-62.7 to -49.9) | -39.4(-85.9 to 156.2) | -59.7(-90.5 to 71.6) |
| **Canada** | 283743(178830 to 435176) | 2259(536 to 6134) | 8691(2162 to 23347) |  | -77.3(-79.9 to -74.3) | -70.1(-88.8 to -0.7) |  | 1.1(-11.4 to 14.8) | 53.1(-45.0 to 265.1) | -1.9(-65.0 to 133.2) |
| **Central African Republic** | 288773594(228854929 to 385394270) | 152034(18259 to 502593) | 11424243(1383877 to 39051135) |  | 180.8(89.3 to 297.0) | 1285.0(78.5 to 13876.0) |  | -49.0(-57.9 to -38.4) | -13.9(-80.0 to 276.2) | -42.0(-86.6 to 151.7) |
| **Chad** | 87274230(60022752 to 118348863) | 85597(9445 to 292941) | 6408409(738072 to 21532774) |  | 178.6(41.0 to 403.9) | 2163.6(135.2 to 36662.2) |  | -16.5(-32.9 to 7.2) | 15.1(-74.2 to 382.6) | -23.5(-82.9 to 221.6) |
| **Chile** | 4892011(4103482 to 5181878) | 33497(8979 to 88797) | 129449(36121 to 345034) |  | 148.0(128.6 to 165.1) | 877.9(90.4 to 8046.2) |  | -7.3(-22.6 to -0.3) | 46.8(-66.7 to 491.8) | -5.5(-78.7 to 278.7) |
| **China** | 610906(384884 to 909465) | 10390(1639 to 33872) | 57117(8610 to 198428) |  | 32.5(-17.3 to 305.3) | 145.4(-34.8 to 1809.5) |  | -14.9(-30.2 to -0.8) | -9.3(-78.6 to 133.7) | -40.1(-85.9 to 56.7) |
| **Colombia** | 1691496(1042515 to 2592814) | 29472(4187 to 89417) | 182051(27685 to 550619) |  | -25.5(-38.3 to -9.8) | 1133.2(151.7 to 10092.2) |  | -27.4(-40.6 to -14.9) | 26.5(-67.1 to 193.3) | -17.4(-78.6 to 90.7) |
| **Comoros** | 804692(296292 to 3813932) | 1394(56 to 9861) | 123710(7324 to 770980) |  | 228.7(47.2 to 1267.8) | 19684.1(1077.3 to 323750.6) |  | 7.2(-41.8 to 96.0) | 233.0(-29.8 to 1875.8) | 123.5(-51.5 to 1135.5) |
| **Congo** | 73561238(42766519 to 118773183) | 57551(5486 to 219799) | 4235234(440790 to 17556719) |  | -50.2(-62.0 to -28.8) | 171.9(-75.8 to 3463.2) |  | -48.5(-55.7 to -41.4) | 1.3(-78.1 to 378.8) | -32.7(-85.4 to 219.1) |
| **Cook Islands** | 794405(403053 to 1192838) | 1015(63 to 4981) | 5457(373 to 26453) |  | 186.4(103.7 to 516.3) | 754.7(-34.5 to 12526.8) |  | 27.5(9.8 to 48.3) | 125.2(-61.3 to 1220.4) | 47.0(-74.9 to 760.9) |
| **Costa Rica** | 678447(430006 to 1038971) | 5747(660 to 21897) | 35129(4205 to 144100) |  | -60.2(-65.4 to -55.5) | 842.7(51.1 to 10742.9) |  | -28.2(-38.4 to -16.1) | 15.0(-74.0 to 245.9) | -25.1(-83.1 to 124.2) |
| **Croatia** | 92202(57412 to 143762) | 369(59 to 1202) | 821(132 to 2673) |  | -37.0(-61.3 to -0.6) | 27.7(-72.0 to 840.2) |  | -48.5(-54.6 to -41.6) | -53.7(-88.3 to 44.4) | -71.0(-92.7 to -10.3) |
| **Cuba** | 205140(138803 to 301030) | 1802(266 to 5680) | 11420(1777 to 35930) |  | -16.3(-26.3 to -5.0) | -30.9(-77.9 to 170.0) |  | 4.1(-11.0 to 17.4) | 180.1(-41.2 to 819.4) | 78.8(-62.8 to 485.3) |
| **Cyprus** | 91280(57163 to 138311) | 1072(159 to 3586) | 4170(610 to 13887) |  | -46.6(-59.4 to -27.0) | 509.4(31.6 to 6371.2) |  | 34.2(13.9 to 63.7) | -43.5(-87.3 to 58.9) | -64.2(-92.0 to 1.0) |
| **Czechia** | 113615(71856 to 179070) | 2395(613 to 6791) | 5353(1405 to 15310) |  | -4.0(-39.5 to 49.9) | 145.4(-35.7 to 1366.0) |  | 0.5(-11.2 to 11.9) | 48.2(-50.2 to 259.9) | -8.4(-69.1 to 123.0) |
| **C么te d'Ivoire** | 61696683(35792105 to 98184793) | 74305(9203 to 263525) | 5536196(765461 to 20189473) |  | 17.2(-11.8 to 90.8) | 214.4(-34.7 to 1834.6) |  | -59.2(-67.1 to -51.8) | -36.1(-83.4 to 89.1) | -57.4(-89.0 to 26.0) |
| **Democratic People's Republic of Korea** | 1732076(369054 to 3474578) | 33737(2639 to 110592) | 183239(18418 to 545282) |  | 430.8(67.3 to 2945.1) | 35561.3(5204.2 to 1335445.2) |  | 29.1(-10.9 to 142.1) | 113.1(-47.4 to 917.8) | 37.8(-65.7 to 550.7) |
| **Democratic Republic of the Congo** | 31708719(23375941 to 41085447) | 28903(2831 to 106889) | 2159202(212130 to 8051412) |  | 2.7(-24.9 to 38.7) | 568.4(-28.8 to 6517.8) |  | -77.4(-82.0 to -71.2) | -52.7(-90.2 to 83.1) | -68.4(-93.5 to 23.0) |
| **Denmark** | 108284(67171 to 165671) | 1148(220 to 3028) | 4482(934 to 12558) |  | 110.4(60.7 to 203.3) | 642.5(60.5 to 5715.7) |  | -44.8(-51.7 to -37.2) | 11.9(-66.7 to 244.9) | -27.8(-78.7 to 120.7) |
| **Djibouti** | 40030670(23037697 to 66158558) | 105853(28830 to 243629) | 7871898(2336504 to 17524514) |  | 12781.9(4109.3 to 39504.1) | 1658311.9(269529.7 to 27624940.6) |  | -51.6(-63.0 to -39.5) | 17.4(-64.3 to 310.2) | -21.5(-76.3 to 173.5) |
| **Dominica** | 4743310(3007077 to 7096551) | 12164(887 to 62796) | 74822(5594 to 341562) |  | -57.7(-62.6 to -50.8) | -84.8(-98.7 to 149.9) |  | -8.5(-19.4 to 2.1) | 112.1(-61.5 to 1212.5) | 38.2(-75.2 to 749.5) |
| **Dominican Republic** | 9308598(6033236 to 14101359) | 20842(1511 to 106799) | 126295(10380 to 612076) |  | 17.7(-21.2 to 123.8) | -9.6(-92.1 to 1090.2) |  | -31.7(-39.7 to -23.0) | 71.0(-71.8 to 970.7) | 11.5(-81.7 to 597.5) |
| **Ecuador** | 6395336(3717109 to 9995079) | 137857(19992 to 431239) | 853604(124977 to 2827240) |  | 358.6(178.9 to 594.5) | 10969.3(1563.7 to 110727.9) |  | -38.0(-52.2 to -25.8) | -11.5(-77.1 to 147.8) | -41.7(-85.1 to 61.6) |
| **Egypt** | 67262(41282 to 104437) | 566(99 to 1681) | 7975(1424 to 23343) |  | -62.2(-73.2 to -38.8) | 1127.9(198.0 to 11198.1) |  | -36.2(-48.6 to -23.7) | -20.6(-82.3 to 118.6) | -47.2(-88.3 to 45.0) |
| **El Salvador** | 1235346(786463 to 1891704) | 9802(981 to 39377) | 60063(6349 to 232387) |  | -59.3(-67.6 to -47.2) | 357.6(-52.4 to 4353.2) |  | -25.0(-34.8 to -13.4) | 5.6(-72.5 to 408.1) | -31.5(-82.2 to 229.9) |
| **Equatorial Guinea** | 56929709(32364145 to 96312290) | 51474(4443 to 176266) | 3850886(383870 to 13519435) |  | 515.1(173.3 to 1335.2) | 3788.4(329.5 to 58431.3) |  | -45.5(-54.4 to -36.2) | 13.8(-74.8 to 394.1) | -23.4(-83.1 to 230.9) |
| **Eritrea** | 37698498(22953528 to 54133464) | 54391(6340 to 188381) | 4055101(529086 to 14129441) |  | 85.9(-11.2 to 306.1) | 6092.6(548.1 to 120604.8) |  | -55.4(-64.2 to -45.5) | -11.1(-76.8 to 271.2) | -40.8(-84.5 to 148.4) |
| **Estonia** | 1153133(583158 to 1942228) | 253081(105173 to 478832) | 568133(256193 to 1017199) |  | 1691.7(937.7 to 2945.8) | 7499.6(3172.9 to 20048.5) |  | -40.2(-59.1 to -16.3) | -34.6(-61.7 to -3.9) | -59.3(-76.3 to -40.0) |
| **Eswatini** | 576689080(360183949 to 751286854) | 2261591(455282 to 5543592) | 167061728(38554534 to 362196877) |  | 9079.7(5054.4 to 15350.7) | 378189.9(105477.0 to 2378399.3) |  | -61.3(-74.0 to -49.3) | -43.3(-83.6 to 16.6) | -62.3(-89.0 to -22.2) |
| **Ethiopia** | 33369934(20924932 to 48369836) | 39214(6025 to 134958) | 2947094(480842 to 9515644) |  | 28.3(-32.7 to 130.3) | 4435.7(477.4 to 68319.6) |  | -67.2(-72.1 to -62.0) | -28.8(-79.8 to 108.0) | -52.1(-86.4 to 39.1) |
| **Fiji** | 883720(583595 to 1328965) | 807(63 to 4028) | 4301(332 to 20762) |  | 75.8(33.0 to 185.8) | 486.5(-61.3 to 8179.7) |  | -23.8(-33.6 to -14.8) | 86.0(-68.2 to 975.7) | 20.8(-79.4 to 596.2) |
| **Finland** | 137054(84055 to 177773) | 3053(742 to 8518) | 11763(2829 to 30933) |  | -56.6(-72.3 to -40.7) | 234.2(-24.3 to 2489.1) |  | -47.0(-56.3 to -37.0) | 26.7(-60.0 to 257.8) | -18.3(-74.4 to 128.1) |
| **France** | 753888(464133 to 1083473) | 9406(2120 to 27115) | 35704(8739 to 95805) |  | -86.8(-88.7 to -84.6) | -57.4(-85.8 to 83.1) |  | -25.1(-36.6 to -11.6) | 29.1(-56.3 to 198.4) | -17.9(-72.1 to 90.6) |
| **Gabon** | 69794875(39867481 to 110552469) | 74614(7261 to 300695) | 5530485(551217 to 20464970) |  | 301.7(51.2 to 818.5) | 2936.6(191.3 to 45597.5) |  | -59.3(-66.2 to -52.0) | -22.0(-81.2 to 232.8) | -47.9(-87.3 to 122.4) |
| **Gambia** | 51981208(31498998 to 79398324) | 42931(4234 to 164764) | 3196172(332932 to 11332154) |  | 934.2(400.8 to 1940.4) | 7892.7(668.5 to 118926.4) |  | -37.6(-46.7 to -26.2) | -18.1(-82.6 to 233.4) | -45.4(-88.4 to 120.8) |
| **Georgia** | 800806(470656 to 1078548) | 167303(80797 to 273688) | 378201(189538 to 617113) |  | 194.2(127.9 to 256.7) | 27898.7(8068.3 to 236508.2) |  | 115.1(42.8 to 177.8) | 366.3(176.2 to 594.7) | 180.9(63.5 to 313.1) |
| **Germany** | 165971(103973 to 261725) | 4216(1135 to 11079) | 16251(4816 to 42318) |  | -89.5(-90.7 to -88.2) | -66.7(-89.3 to 54.0) |  | -22.1(-34.2 to -10.9) | 99.5(-20.4 to 324.1) | 27.4(-49.2 to 171.3) |
| **Ghana** | 64871251(39195474 to 98147759) | 61458(7182 to 206306) | 4539402(533166 to 14442821) |  | 163.8(35.4 to 363.5) | 1742.1(115.1 to 24011.4) |  | -53.2(-59.5 to -44.0) | -28.8(-83.4 to 184.5) | -53.1(-88.9 to 90.4) |
| **Greece** | 419291(323432 to 467168) | 7901(1093 to 24891) | 30433(4649 to 97846) |  | -61.7(-67.5 to -56.9) | 371.1(1.0 to 4260.1) |  | 29.7(4.3 to 48.9) | 9.9(-75.1 to 186.2) | -29.5(-84.2 to 82.8) |
| **Greenland** | 3480396(2115845 to 5011746) | 44613(4651 to 161146) | 169935(18140 to 651006) |  | -47.3(-62.3 to -16.3) | -44.6(-93.9 to 378.8) |  | -12.9(-27.4 to 0.9) | 60.0(-66.7 to 691.0) | 1.3(-78.7 to 405.8) |
| **Grenada** | 581948(406329 to 814288) | 1529(112 to 7174) | 9937(741 to 51296) |  | -73.9(-77.1 to -69.6) | -78.8(-97.9 to 100.4) |  | -30.0(-38.4 to -20.9) | 124.8(-69.4 to 1429.0) | 47.7(-80.3 to 881.3) |
| **Guam** | 468224(318012 to 694940) | 1017(93 to 3935) | 5574(542 to 21374) |  | -15.5(-27.8 to 18.1) | 1065.9(130.4 to 13473.4) |  | -14.6(-22.5 to -4.7) | -66.7(-95.5 to 27.2) | -78.1(-97.1 to -17.5) |
| **Guatemala** | 4270042(2665799 to 6411863) | 45315(5560 to 148634) | 278710(35064 to 914912) |  | -27.9(-54.9 to 10.3) | 1106.6(86.1 to 12453.7) |  | -40.2(-48.6 to -30.4) | -18.0(-77.4 to 143.7) | -46.2(-85.3 to 58.7) |
| **Guinea** | 75936037(48667202 to 105431150) | 60578(7427 to 208063) | 4521911(540139 to 14540107) |  | 745.6(374.7 to 1205.5) | 5762.0(547.3 to 78938.6) |  | -29.4(-40.9 to -14.5) | -11.7(-77.4 to 286.1) | -40.7(-85.0 to 157.0) |
| **Guinea-Bissau** | 138734294(88848777 to 195345229) | 91401(10763 to 318441) | 6851824(809570 to 25406663) |  | 1197.3(672.9 to 1864.5) | 9810.3(1201.0 to 127252.1) |  | -43.2(-52.9 to -31.9) | -36.6(-85.6 to 170.7) | -57.2(-90.4 to 80.7) |
| **Guyana** | 15333086(9634355 to 23314470) | 44184(3534 to 230201) | 270435(22201 to 1435498) |  | 11.7(-1.2 to 30.0) | 75.0(-83.0 to 1969.1) |  | -38.5(-46.0 to -30.4) | 55.3(-73.1 to 868.4) | 1.7(-82.5 to 531.7) |
| **Haiti** | 18292955(11105734 to 28509732) | 32494(2388 to 142958) | 198543(14920 to 871728) |  | -34.8(-45.2 to -17.9) | -39.4(-94.7 to 513.8) |  | -54.1(-58.6 to -48.5) | 8.8(-82.5 to 671.7) | -28.7(-88.6 to 402.5) |
| **Honduras** | 2314276(1679567 to 2998427) | 24333(3080 to 79712) | 150190(20091 to 487453) |  | -49.4(-63.4 to -18.6) | 1251.0(108.0 to 16280.7) |  | -14.2(-35.7 to 5.6) | 34.3(-65.7 to 300.2) | -12.4(-77.8 to 160.5) |
| **Hungary** | 57042(36920 to 88499) | 782(101 to 2903) | 1772(237 to 6621) |  | -90.5(-91.7 to -89.2) | 7.4(-75.0 to 1018.3) |  | -50.0(-56.8 to -42.3) | -69.0(-93.9 to -0.6) | -80.6(-96.2 to -38.2) |
| **Iceland** | 120314(73980 to 191589) | 717(71 to 2666) | 2775(284 to 10440) |  | -78.3(-82.7 to -74.4) | 128.7(-60.0 to 2017.3) |  | -35.2(-44.1 to -20.6) | -80.4(-96.2 to -36.4) | -87.4(-97.6 to -59.2) |
| **India** | 3996586(2806986 to 5255277) | 33705(5833 to 87488) | 669883(113101 to 1641942) |  | 4205.7(2312.5 to 6693.2) | 281764.0(42930.7 to 6081649.4) |  | -65.0(-72.4 to -57.7) | -37.7(-81.7 to 55.4) | -58.5(-87.8 to 3.5) |
| **Indonesia** | 8697795(6980570 to 10725346) | 12385(1045 to 49815) | 72115(6517 to 287435) |  | — | — |  | 30.1(14.4 to 49.2) | 5.2(-79.3 to 535.1) | -35.6(-87.0 to 274.5) |
| **Iran (Islamic Republic of)** | 1541294(1071531 to 2060597) | 5063(1011 to 13732) | 69342(14678 to 198618) |  | 401.1(224.8 to 892.6) | 10916.1(1982.0 to 167282.1) |  | 5.4(-8.8 to 24.7) | 73.1(-62.1 to 630.3) | 13.3(-75.0 to 382.9) |
| **Iraq** | 5442(3380 to 8609) | 31(7 to 88) | 430(96 to 1148) |  | -13.6(-29.6 to 11.0) | 2115.8(448.6 to 34192.9) |  | -43.8(-52.7 to -33.0) | 0.3(-72.7 to 244.3) | -33.7(-82.0 to 127.2) |
| **Ireland** | 369405(269597 to 410380) | 3250(625 to 10102) | 12554(2644 to 38093) |  | -33.7(-51.5 to -24.6) | 118.9(-44.5 to 2065.3) |  | -34.2(-41.3 to -25.9) | -23.4(-81.3 to 128.0) | -50.9(-88.0 to 45.8) |
| **Israel** | 91630(55024 to 147333) | 3482(802 to 9469) | 13495(3338 to 35731) |  | -77.1(-80.7 to -72.5) | -51.3(-86.4 to 212.1) |  | -29.1(-46.7 to -14.2) | 21.0(-61.0 to 186.4) | -22.5(-75.1 to 83.8) |
| **Italy** | 117153(73204 to 178939) | 2021(530 to 5234) | 7786(2259 to 19438) |  | -83.6(-85.3 to -81.6) | -43.2(-83.3 to 203.7) |  | -17.0(-25.1 to -9.2) | -6.0(-62.6 to 91.1) | -39.8(-76.3 to 21.7) |
| **Jamaica** | 1029586(684750 to 1502501) | 5696(683 to 20378) | 35753(4664 to 124148) |  | -48.7(-55.3 to -40.8) | -3.6(-89.0 to 1496.7) |  | -33.1(-42.4 to -24.8) | 76.7(-66.9 to 837.4) | 13.3(-78.7 to 505.9) |
| **Japan** | 126297(77111 to 173041) | 685(65 to 2936) | 2621(249 to 11322) |  | 39.2(-3.1 to 98.9) | 69.2(-76.4 to 982.7) |  | -14.9(-22.4 to -8.4) | 45.6(-73.0 to 463.7) | -7.2(-82.8 to 260.3) |
| **Jordan** | 12787(7399 to 20136) | 96(13 to 338) | 1325(195 to 4474) |  | -56.6(-64.8 to -45.8) | 1403.2(238.9 to 17637.1) |  | -25.3(-42.1 to -9.3) | -27.8(-84.1 to 99.4) | -52.6(-89.6 to 31.5) |
| **Kazakhstan** | 237680(111703 to 404059) | 64067(27429 to 112844) | 145333(65888 to 259513) |  | -30.7(-57.7 to 1.1) | 8137.7(2047.0 to 76100.8) |  | -59.5(-72.6 to -42.2) | -66.3(-79.1 to -52.7) | -78.7(-86.8 to -70.3) |
| **Kenya** | 104624461(64043536 to 151243368) | 56989(11968 to 161673) | 4253416(1004526 to 11638282) |  | 39.3(7.9 to 83.0) | 4437.8(991.0 to 67051.0) |  | -54.8(-59.3 to -49.6) | -33.7(-84.1 to 168.5) | -55.7(-89.4 to 79.3) |
| **Kiribati** | 3031635(2007690 to 4020224) | 4254(338 to 18178) | 22627(1738 to 99747) |  | 63.5(7.2 to 110.9) | 466.8(-53.1 to 6626.4) |  | -23.4(-33.1 to -10.1) | 23.9(-77.8 to 671.8) | -19.7(-85.6 to 400.5) |
| **Kuwait** | 17924(11170 to 28429) | 54(10 to 167) | 759(145 to 2274) |  | -52.6(-58.5 to -46.5) | 399.9(15.1 to 7535.6) |  | -57.0(-62.5 to -50.9) | -30.7(-82.4 to 110.7) | -54.0(-88.3 to 40.0) |
| **Kyrgyzstan** | 642775(225177 to 1218052) | 213860(75246 to 411130) | 483353(185895 to 892461) |  | -14.3(-46.6 to 21.7) | 38348.7(8324.0 to 428217.8) |  | -36.3(-74.2 to 13.2) | -20.1(-65.3 to 41.1) | -49.7(-78.4 to -11.1) |
| **Lao People's Democratic Republic** | 10090341(1659638 to 45290250) | 21993(292 to 163381) | 116499(2535 to 847708) |  | — | — |  | -34.7(-67.3 to 44.8) | -45.1(-91.2 to 250.2) | -64.5(-94.1 to 124.9) |
| **Latvia** | 1653058(989188 to 2547032) | 180258(65375 to 376018) | 405406(147204 to 811218) |  | 31.6(2.4 to 86.3) | 1.6(-40.0 to 99.5) |  | -19.4(-37.5 to 2.7) | -10.0(-52.4 to 44.7) | -44.0(-70.4 to -10.2) |
| **Lebanon** | 182091(77668 to 350641) | 621(45 to 2654) | 8607(629 to 38038) |  | -57.8(-69.1 to -25.0) | 385.4(-41.0 to 6179.1) |  | -34.9(-53.9 to -18.8) | 20.7(-76.6 to 325.3) | -21.0(-84.6 to 180.6) |
| **Lesotho** | 1112636763(872521601 to 1403125606) | 1816753(480024 to 4553215) | 134539945(37732962 to 324816386) |  | 1374.5(901.1 to 1952.4) | 85850.2(19312.4 to 1028274.4) |  | -24.1(-37.0 to -10.1) | 35.4(-68.4 to 390.5) | -9.9(-78.9 to 226.3) |
| **Liberia** | 13968842(8409694 to 22222691) | 10313(993 to 40416) | 760189(74479 to 2956348) |  | 54.6(-14.3 to 223.0) | 831.7(-15.2 to 12470.2) |  | -42.5(-52.3 to -31.6) | -21.0(-80.3 to 250.5) | -47.5(-86.9 to 133.1) |
| **Libya** | 569383(146872 to 1157793) | 2006(62 to 10127) | 28442(1076 to 142233) |  | 25.8(-27.5 to 231.2) | 1069.1(12.2 to 22816.8) |  | -6.5(-60.0 to 25.8) | 54.5(-80.3 to 717.0) | 1.9(-86.7 to 436.0) |
| **Lithuania** | 437812(239425 to 720219) | 92828(40474 to 175798) | 208187(96454 to 384058) |  | 33.3(-1.5 to 72.1) | 432.9(111.1 to 1965.7) |  | -41.8(-56.9 to -25.7) | -9.4(-40.3 to 28.4) | -43.4(-62.9 to -20.1) |
| **Luxembourg** | 96953(60507 to 147071) | 1572(180 to 5722) | 6137(748 to 22463) |  | -82.4(-86.1 to -79.2) | 117.6(-58.8 to 1898.3) |  | -32.8(-43.8 to -18.7) | -35.9(-88.9 to 141.9) | -58.8(-92.9 to 55.0) |
| **Madagascar** | 2821114(1732029 to 4348737) | 2861(346 to 9941) | 213420(27963 to 712708) |  | 9795.1(3787.6 to 32664.0) | 305184.3(43034.9 to 5000222.2) |  | -20.8(-34.3 to -10.1) | 146.3(-42.5 to 669.5) | 64.7(-61.7 to 412.6) |
| **Malawi** | 228439780(190720315 to 271955324) | 159049(24608 to 528682) | 11886782(1938117 to 37495776) |  | 75.2(7.0 to 171.5) | 4404.9(805.8 to 55201.6) |  | -70.6(-75.5 to -61.7) | -12.9(-83.4 to 198.4) | -41.5(-88.9 to 100.8) |
| **Malaysia** | 5767222(3726080 to 8531271) | 23194(4039 to 74755) | 122980(23502 to 356188) |  | 603.6(326.6 to 1050.4) | 15567.1(2108.8 to 168117.4) |  | -16.7(-29.4 to -4.5) | -3.2(-81.4 to 417.2) | -36.9(-88.0 to 234.3) |
| **Maldives** | 303361(202930 to 393325) | 620(53 to 2619) | 3375(291 to 14615) |  | 186.6(125.2 to 234.3) | 1638.1(49.9 to 26018.8) |  | -28.3(-46.0 to -5.5) | -48.7(-90.5 to 175.6) | -66.8(-93.8 to 78.7) |
| **Mali** | 35171273(21502829 to 52798314) | 34681(3450 to 116797) | 2578209(267238 to 8457472) |  | 244.0(47.3 to 653.4) | 2521.7(184.5 to 37233.9) |  | -44.7(-54.0 to -33.3) | -20.1(-81.5 to 247.8) | -46.8(-87.7 to 132.9) |
| **Malta** | 98766(63080 to 151236) | 470(50 to 2022) | 1852(193 to 7846) |  | -65.5(-71.5 to -60.2) | 180.7(-58.9 to 3468.7) |  | -13.2(-24.7 to 1.3) | -62.9(-93.0 to 20.5) | -76.4(-95.5 to -23.6) |
| **Marshall Islands** | 1115883(214347 to 2131185) | 5368(244 to 24288) | 28718(1508 to 122033) |  | 72.1(-50.5 to 664.3) | 5578.4(725.9 to 118750.2) |  | 10.7(-38.3 to 87.0) | -25.1(-89.6 to 217.3) | -51.5(-93.1 to 106.3) |
| **Mauritania** | 1546182(588107 to 6748125) | 909(39 to 6649) | 77104(4493 to 475917) |  | -72.6(-91.7 to 8.9) | 136.1(-77.7 to 4476.0) |  | -33.3(-53.9 to 3.2) | -7.0(-79.5 to 307.5) | -39.4(-86.7 to 156.1) |
| **Mauritius** | 790474(533834 to 1156076) | 1738(282 to 5485) | 9460(1614 to 30210) |  | 10.4(-5.0 to 26.8) | 1466.1(172.7 to 16307.0) |  | 6.3(-5.9 to 19.6) | -31.2(-82.2 to 146.7) | -55.7(-88.6 to 59.3) |
| **Mexico** | 1871444(1191774 to 2817800) | 22621(3384 to 71929) | 139795(23323 to 438490) |  | -50.1(-61.3 to -35.7) | 2081.7(405.9 to 20560.9) |  | -26.2(-37.8 to -18.7) | 21.9(-70.6 to 246.6) | -19.7(-80.8 to 126.5) |
| **Micronesia (Federated States of)** | 8280011(1616219 to 13962895) | 11765(388 to 61319) | 63193(2225 to 302499) |  | 80.7(-43.1 to 159.6) | 467.4(-69.1 to 7722.9) |  | 41.6(-11.3 to 136.3) | 149.0(-58.5 to 1266.0) | 60.4(-73.1 to 783.6) |
| **Monaco** | 1145780(457194 to 1773566) | 8968(745 to 36335) | 34727(2912 to 141592) |  | -40.6(-66.9 to 42.3) | 65.8(-82.3 to 2267.9) |  | -15.3(-33.0 to -1.2) | 25.7(-75.2 to 516.2) | -19.0(-84.2 to 294.7) |
| **Mongolia** | 97298(45841 to 169364) | 9894(1943 to 27326) | 22479(4566 to 60268) |  | — | — |  | -16.2(-49.5 to 11.0) | 83.3(-42.0 to 351.0) | 15.6(-63.7 to 186.2) |
| **Montenegro** | 83565(51204 to 130958) | 835(93 to 3075) | 1846(211 to 7060) |  | 16.9(2.6 to 36.9) | 590.6(39.9 to 6700.7) |  | -21.1(-30.6 to -11.7) | -16.6(-85.4 to 201.7) | -48.4(-91.0 to 87.4) |
| **Morocco** | 1107562(187717 to 2817551) | 4394(219 to 18311) | 61397(3990 to 258980) |  | 125.5(-35.9 to 589.0) | 4223.2(451.6 to 77401.5) |  | -57.4(-73.9 to -31.6) | -24.1(-81.1 to 278.5) | -49.4(-87.5 to 145.9) |
| **Mozambique** | 535534260(361237197 to 710656453) | 1051559(260655 to 2639218) | 77780729(20050407 to 184999708) |  | 1408.9(796.1 to 2242.5) | 24491.3(5687.7 to 207520.5) |  | -33.9(-48.1 to -18.2) | 25.3(-59.5 to 202.1) | -16.6(-73.1 to 101.4) |
| **Myanmar** | 16258978(11118824 to 21820021) | 467491(132080 to 1109929) | 2487923(734165 to 5816796) |  | 13295.5(9363.5 to 17839.0) | 532742.1(108939.1 to 5535895.9) |  | -74.2(-80.3 to -68.5) | -53.9(-83.9 to -4.1) | -70.0(-89.5 to -37.3) |
| **Namibia** | 230809395(139953518 to 346924505) | 502670(134790 to 1200870) | 37450974(11216911 to 82838709) |  | 646.2(297.3 to 1049.5) | 56909.6(11141.1 to 564135.9) |  | -52.2(-63.1 to -39.4) | -13.8(-76.3 to 208.3) | -42.5(-84.3 to 105.8) |
| **Nauru** | 2130197(599954 to 3242776) | 2854(155 to 15641) | 15508(944 to 74895) |  | 392.0(152.9 to 1458.7) | 1341.6(-4.7 to 25183.5) |  | -13.3(-29.6 to 41.3) | 49.3(-74.7 to 839.1) | -1.7(-83.5 to 521.7) |
| **Nepal** | 1972482(325436 to 3966371) | 13353(719 to 46159) | 262900(16856 to 915215) |  | — | — |  | -58.3(-75.9 to -42.0) | -23.1(-82.8 to 99.8) | -48.9(-88.6 to 32.4) |
| **Netherlands** | 304173(189379 to 459267) | 3962(943 to 10724) | 15280(3773 to 39537) |  | -84.2(-86.7 to -81.4) | -34.0(-80.0 to 240.6) |  | -16.8(-28.5 to -5.1) | 19.1(-53.9 to 173.6) | -23.5(-70.5 to 75.0) |
| **New Zealand** | 82829(51224 to 129077) | 980(157 to 3208) | 3790(622 to 11619) |  | -82.6(-84.8 to -80.1) | -59.1(-89.1 to 164.4) |  | -21.3(-32.1 to -10.7) | 4.9(-73.8 to 212.3) | -32.8(-83.3 to 99.4) |
| **Nicaragua** | 672984(435001 to 1015941) | 5192(709 to 17199) | 31965(3988 to 104486) |  | -28.3(-41.9 to -2.5) | 294.5(-28.0 to 3098.4) |  | -28.0(-36.2 to -20.2) | 21.1(-70.8 to 245.5) | -20.6(-80.8 to 125.4) |
| **Niger** | 19476189(13335674 to 25610576) | 17315(2067 to 61048) | 1295705(150132 to 4310574) |  | 218.5(56.9 to 481.6) | 2402.2(220.3 to 37990.1) |  | -50.8(-61.6 to -38.6) | -39.0(-84.8 to 178.0) | -58.9(-89.9 to 85.5) |
| **Nigeria** | 41775832(27231790 to 61682597) | 59002(11463 to 172719) | 4445152(890832 to 12619813) |  | 179.8(69.5 to 351.7) | 12860.5(2768.4 to 152111.0) |  | -39.8(-50.1 to -31.5) | -10.2(-77.5 to 129.6) | -39.5(-85.0 to 53.6) |
| **Niue** | 1513094(485107 to 2293431) | 1827(125 to 8301) | 9758(749 to 44628) |  | 169.9(59.9 to 850.5) | 750.0(-44.6 to 12222.9) |  | 16.9(-8.5 to 41.3) | 90.4(-64.9 to 1291.0) | 22.2(-77.0 to 804.5) |
| **North Macedonia** | 127882(72445 to 214956) | 1927(386 to 5970) | 4237(872 to 12155) |  | 91.0(10.8 to 226.7) | 1839.0(300.5 to 18147.0) |  | -15.3(-25.5 to -4.5) | -21.8(-78.2 to 123.5) | -51.4(-86.5 to 38.6) |
| **Northern Mariana Islands** | 315847(213621 to 475396) | 1532(164 to 6605) | 8284(1000 to 35072) |  | -1.4(-26.7 to 137.0) | 247.3(-53.4 to 6435.3) |  | -8.0(-19.0 to 3.0) | 26.7(-78.3 to 389.8) | -18.1(-86.1 to 216.0) |
| **Norway** | 182846(114652 to 273493) | 2866(621 to 8521) | 10975(2427 to 30187) |  | -68.6(-71.8 to -64.9) | -38.9(-82.8 to 183.6) |  | -28.6(-37.2 to -21.0) | -4.7(-70.7 to 160.8) | -39.0(-81.3 to 66.9) |
| **Oman** | 79729(52735 to 117119) | 266(40 to 898) | 3806(610 to 12011) |  | -44.1(-60.7 to -27.6) | 10.9(-70.9 to 583.8) |  | -56.9(-62.3 to -51.2) | -30.6(-85.2 to 118.7) | -53.7(-90.0 to 46.0) |
| **Pakistan** | 911408(148548 to 1828390) | 6739(351 to 20698) | 134819(8871 to 415455) |  | 28.9(-72.1 to 572.9) | 11751.0(1276.4 to 311654.7) |  | 14.3(-41.1 to 112.0) | 120.9(-55.1 to 734.0) | 47.7(-68.4 to 448.5) |
| **Palau** | 1661379(446634 to 2534793) | 2428(117 to 11857) | 12912(721 to 67798) |  | 194.2(81.5 to 906.7) | 877.9(-27.8 to 14351.3) |  | 40.7(0.8 to 68.1) | 150.1(-61.0 to 1514.8) | 60.9(-74.9 to 944.0) |
| **Palestine** | 36879(22011 to 58651) | 145(12 to 648) | 2003(189 to 8736) |  | -33.0(-54.6 to -12.0) | 488.0(-36.2 to 7236.3) |  | -33.9(-46.1 to -17.8) | 20.2(-75.6 to 488.7) | -21.1(-84.2 to 287.0) |
| **Panama** | 6007499(3702808 to 9113705) | 57346(5307 to 226121) | 353297(35590 to 1356407) |  | -15.2(-31.4 to -4.2) | 1153.6(49.5 to 12353.2) |  | -32.3(-43.3 to -18.7) | -3.5(-79.0 to 333.2) | -37.0(-86.4 to 182.8) |
| **Papua New Guinea** | 16210822(8859589 to 26466860) | 294706(66366 to 748699) | 1566992(365075 to 4194499) |  | 17190.5(6035.5 to 61524.0) | 582273.2(89615.9 to 10773595.2) |  | -38.2(-48.0 to -28.9) | 181.8(-27.6 to 989.4) | 83.1(-52.9 to 611.7) |
| **Paraguay** | 5012360(3120853 to 7696934) | 69246(6856 to 249076) | 424632(43194 to 1503375) |  | 190.4(76.7 to 459.3) | 3329.5(513.9 to 34699.6) |  | -29.8(-43.0 to -18.6) | 50.6(-74.6 to 345.6) | -1.8(-83.5 to 190.1) |
| **Peru** | 2961400(1799322 to 4630900) | 100715(32317 to 216872) | 615226(212304 to 1307225) |  | -20.9(-51.1 to 21.5) | 727.1(180.1 to 3614.3) |  | -41.8(-52.2 to -30.0) | -27.2(-60.2 to 27.0) | -52.6(-74.1 to -17.4) |
| **Philippines** | 446244(286058 to 683033) | 5888(1159 to 16313) | 31309(6641 to 84363) |  | 46.6(27.8 to 69.8) | 6170.5(1581.5 to 61493.2) |  | -22.2(-32.7 to -13.1) | 11.6(-71.0 to 211.5) | -27.3(-81.1 to 102.6) |
| **Poland** | 170305(107443 to 264890) | 1442(367 to 4039) | 3195(815 to 9181) |  | 95.5(31.5 to 199.0) | 277.0(18.3 to 1746.6) |  | -34.1(-37.6 to -30.3) | -10.6(-69.3 to 123.5) | -44.2(-80.9 to 39.3) |
| **Portugal** | 2229332(1424152 to 3342128) | 21378(3632 to 70441) | 81891(13976 to 247325) |  | -24.6(-37.9 to 5.6) | 2.4(-63.6 to 263.4) |  | -33.1(-42.6 to -23.8) | -6.2(-77.1 to 152.8) | -39.9(-85.4 to 61.7) |
| **Puerto Rico** | 820187(525411 to 1215947) | 4393(625 to 14929) | 27114(3910 to 90018) |  | -89.5(-90.8 to -87.9) | 12.9(-73.3 to 803.0) |  | -22.2(-31.2 to -11.0) | -26.0(-83.1 to 128.2) | -52.3(-89.2 to 47.6) |
| **Qatar** | 7481(4990 to 11229) | 26(4 to 92) | 367(63 to 1279) |  | -82.0(-84.7 to -78.1) | 24.5(-73.4 to 1312.3) |  | -43.5(-51.7 to -36.8) | 8.7(-75.2 to 292.6) | -27.7(-83.6 to 161.2) |
| **Republic of Korea** | 348193(299690 to 379574) | 3081(368 to 11892) | 11859(1390 to 46294) |  | 57.4(37.9 to 95.9) | 371.2(-7.9 to 3156.6) |  | -26.2(-34.4 to -19.0) | -5.4(-81.7 to 208.3) | -39.7(-88.3 to 97.1) |
| **Republic of Moldova** | 384763(150439 to 731225) | 263724(132288 to 438673) | 598731(333511 to 947515) |  | 94.2(18.0 to 177.2) | 7150.1(1853.9 to 64709.4) |  | -73.2(-86.0 to -59.2) | -39.3(-52.8 to -20.4) | -61.6(-70.3 to -49.6) |
| **Romania** | 973037(603132 to 1526533) | 34247(9964 to 83103) | 76024(21987 to 186206) |  | -14.4(-37.5 to 1.5) | 178.3(-21.9 to 975.7) |  | -17.1(-33.6 to 12.6) | 26.1(-66.0 to 364.2) | -21.0(-78.8 to 189.9) |
| **Russian Federation** | 1417626(718103 to 2488450) | 407974(169475 to 748269) | 911826(410144 to 1652510) |  | 103.9(20.5 to 188.9) | 4365.2(1250.1 to 18433.5) |  | -53.2(-67.1 to -32.5) | -27.8(-50.6 to 7.1) | -54.7(-69.2 to -32.3) |
| **Rwanda** | 57622014(41372037 to 70096652) | 63878(17865 to 156784) | 4794312(1383595 to 11745894) |  | 68.8(-30.4 to 258.5) | 9553.0(1860.0 to 120481.7) |  | -60.0(-66.1 to -53.4) | -19.2(-78.6 to 229.6) | -45.5(-85.7 to 120.7) |
| **Saint Kitts and Nevis** | 4416788(2799224 to 6640854) | 9693(755 to 48434) | 59216(4940 to 288022) |  | -15.6(-37.3 to 66.8) | -41.4(-95.5 to 542.9) |  | -11.5(-20.8 to -0.9) | 116.8(-60.2 to 978.0) | 39.9(-74.2 to 600.6) |
| **Saint Lucia** | 1781994(1159591 to 2657818) | 3334(300 to 14108) | 20438(1998 to 83584) |  | -72.9(-75.5 to -69.7) | -89.3(-99.1 to 58.3) |  | -22.5(-29.8 to -14.3) | 58.3(-70.1 to 627.6) | 2.3(-80.6 to 371.2) |
| **Saint Vincent and the Grenadines** | 4084688(2660150 to 6087590) | 8842(691 to 40887) | 55196(4420 to 230882) |  | -51.7(-57.1 to -43.2) | -85.6(-98.6 to 164.9) |  | -13.1(-22.7 to -4.4) | 94.3(-65.1 to 850.6) | 26.9(-77.4 to 515.2) |
| **Samoa** | 1706095(344378 to 3370204) | 505(16 to 2368) | 2721(112 to 12472) |  | 79.2(-38.4 to 683.3) | 399.5(-70.8 to 8190.2) |  | 17.8(-37.0 to 115.6) | 14.3(-81.2 to 760.9) | -26.5(-87.2 to 444.8) |
| **San Marino** | 376481(227219 to 572358) | 2565(267 to 9322) | 9924(1040 to 34302) |  | -50.5(-59.8 to -35.7) | 23.7(-86.9 to 1497.7) |  | -8.3(-25.2 to 7.3) | 30.7(-75.4 to 508.0) | -16.9(-84.3 to 289.0) |
| **Sao Tome and Principe** | 816945(557769 to 1115407) | 583(61 to 1904) | 54919(5871 to 169485) |  | -41.6(-59.6 to -17.6) | 487.1(-35.0 to 7149.7) |  | -34.2(-47.5 to -20.1) | 18.8(-75.7 to 410.9) | -24.2(-84.9 to 220.8) |
| **Saudi Arabia** | 674024(420908 to 1040240) | 3685(553 to 12630) | 50363(8234 to 168288) |  | 24.9(-23.5 to 117.5) | 3154.0(641.2 to 40553.0) |  | -54.8(-62.6 to -44.8) | -28.4(-84.7 to 104.1) | -52.7(-89.9 to 35.3) |
| **Senegal** | 15592418(9676775 to 23544151) | 13274(2547 to 38108) | 985366(220660 to 2543792) |  | 135.4(23.2 to 332.3) | 7164.0(1122.7 to 96787.4) |  | -40.2(-49.0 to -31.3) | -9.0(-79.5 to 288.0) | -39.8(-86.3 to 159.1) |
| **Serbia** | 782610(497051 to 1122223) | 7545(1397 to 22171) | 16662(3300 to 48871) |  | — | — |  | -40.8(-49.6 to -19.0) | -27.8(-80.2 to 90.5) | -55.1(-87.7 to 18.4) |
| **Seychelles** | 1255770(828348 to 1843224) | 1318(84 to 7359) | 7058(470 to 41329) |  | 62.5(0.4 to 168.3) | 970.9(-19.0 to 13999.7) |  | -29.3(-35.4 to -20.9) | -34.2(-92.0 to 282.5) | -57.4(-94.8 to 150.2) |
| **Sierra Leone** | 29890352(18357932 to 46489489) | 24737(2653 to 92453) | 1856818(198612 to 6451191) |  | 497.6(162.1 to 1279.4) | 2505.7(183.4 to 26715.4) |  | -49.0(-55.8 to -39.3) | -23.2(-82.2 to 186.9) | -48.9(-88.1 to 91.8) |
| **Singapore** | 183305(119715 to 280381) | 1341(374 to 3700) | 5223(1514 to 13401) |  | -44.2(-59.2 to -15.9) | 61.1(-55.3 to 802.8) |  | -34.0(-40.4 to -26.5) | -14.7(-65.1 to 87.2) | -45.3(-77.7 to 19.8) |
| **Slovakia** | 10619(6246 to 17504) | 81(11 to 307) | 180(25 to 690) |  | — | — |  | -33.0(-40.4 to -25.5) | -35.9(-86.1 to 92.6) | -60.1(-91.3 to 19.7) |
| **Slovenia** | 43288(26346 to 70014) | 51(6 to 210) | 115(13 to 471) |  | -83.9(-86.1 to -81.6) | -86.4(-97.5 to 2.5) |  | -4.5(-15.2 to 7.8) | -59.6(-92.3 to 46.9) | -74.7(-95.2 to -8.8) |
| **Solomon Islands** | 1720299(305906 to 3453647) | 2731(54 to 12687) | 14115(369 to 64629) |  | 86.6(-47.4 to 731.3) | 559.6(-64.5 to 15964.0) |  | -0.8(-44.5 to 101.5) | 67.1(-77.3 to 1189.0) | 7.5(-84.5 to 716.4) |
| **Somalia** | 35129266(21898898 to 50195872) | 94280(21885 to 261948) | 6942614(1652949 to 18516166) |  | 5042.4(1508.8 to 17041.7) | 948067.9(177022.4 to 15959125.3) |  | -42.9(-60.8 to -29.0) | -18.3(-75.6 to 117.2) | -45.7(-83.8 to 44.9) |
| **South Africa** | 325986290(200160025 to 468307849) | 304251(70974 to 870941) | 22711931(5594660 to 58565853) |  | 3001.9(1652.9 to 4342.7) | 21491.5(5375.1 to 143430.2) |  | -58.4(-64.0 to -51.2) | -43.9(-83.8 to 85.9) | -62.2(-89.2 to 24.4) |
| **South Sudan** | 65542867(40030347 to 101779812) | 96081(10716 to 300016) | 7133825(879300 to 22638489) |  | 622.1(154.3 to 1782.2) | 28256.3(3319.7 to 432408.6) |  | -26.0(-43.9 to -9.3) | 37.0(-64.9 to 465.4) | -8.3(-76.6 to 277.2) |
| **Spain** | 420494(262331 to 646769) | 3267(320 to 14023) | 12374(1306 to 54745) |  | -86.9(-88.4 to -85.3) | -76.1(-95.8 to 62.0) |  | -35.3(-43.1 to -26.7) | -21.3(-83.8 to 161.2) | -50.3(-89.6 to 67.0) |
| **Sri Lanka** | 216437(138898 to 320581) | 396(27 to 1749) | 2103(162 to 9879) |  | -32.0(-52.9 to 36.3) | 423.9(-50.2 to 7771.5) |  | -26.6(-38.0 to -17.5) | -18.8(-85.2 to 322.9) | -46.7(-90.4 to 174.5) |
| **Sudan** | 2071808(1094407 to 3374096) | 7879(682 to 35432) | 108576(9894 to 467195) |  | 89.3(-0.3 to 472.2) | 1560.7(47.0 to 35993.8) |  | -45.8(-53.8 to -37.3) | -13.8(-81.3 to 312.6) | -42.8(-87.7 to 175.5) |
| **Suriname** | 5230003(3377231 to 7848043) | 8232(738 to 33451) | 50973(5025 to 196638) |  | -49.6(-54.6 to -43.9) | -62.6(-96.8 to 309.3) |  | -24.4(-31.6 to -16.3) | 52.6(-71.7 to 772.4) | -0.5(-81.7 to 463.3) |
| **Sweden** | 159160(96045 to 251416) | 3288(829 to 9228) | 12631(3219 to 32729) |  | -76.6(-80.9 to -71.7) | 15.0(-68.0 to 611.2) |  | -27.6(-38.1 to -17.2) | -5.1(-65.9 to 116.1) | -39.0(-78.2 to 38.9) |
| **Switzerland** | 125139(76375 to 192719) | 3232(785 to 8021) | 12496(3329 to 29963) |  | 94.2(26.0 to 195.6) | 646.0(116.0 to 4601.0) |  | -35.0(-45.8 to -25.4) | 85.1(-29.4 to 301.9) | 18.7(-54.9 to 158.1) |
| **Syrian Arab Republic** | 33921(20330 to 53689) | 164(18 to 599) | 2267(259 to 8739) |  | -53.3(-63.2 to -38.3) | 504.7(-12.8 to 8986.0) |  | 8.7(-9.3 to 28.5) | 47.6(-67.4 to 431.6) | -4.8(-79.1 to 242.9) |
| **Taiwan (Province of China)** | 461374(296275 to 687966) | 4362(375 to 17889) | 23811(2256 to 94957) |  | 319.3(174.8 to 525.5) | 1173.7(21.0 to 15393.8) |  | -3.3(-17.6 to 14.3) | 37.0(-71.7 to 587.3) | -10.7(-81.8 to 344.4) |
| **Tajikistan** | 422497(222050 to 691359) | 70891(25434 to 142298) | 158876(60522 to 307254) |  | 77.9(-3.5 to 280.7) | 36727.8(8328.2 to 474508.2) |  | -46.0(-66.9 to -16.3) | -3.8(-61.4 to 140.8) | -39.7(-75.9 to 51.0) |
| **Thailand** | 11085987(6954376 to 16710449) | 128835(24523 to 370076) | 684442(129329 to 1862490) |  | 131.4(35.8 to 278.0) | 833.1(139.2 to 5667.6) |  | -21.2(-32.1 to -8.7) | 8.6(-72.2 to 185.4) | -29.3(-82.0 to 85.4) |
| **Timor-Leste** | 1971094(568349 to 3476818) | 4837(239 to 23217) | 25085(1350 to 122026) |  | 175.7(46.7 to 1008.3) | 1246.9(11.3 to 28606.5) |  | -29.6(-50.5 to -10.8) | -44.4(-90.6 to 187.1) | -64.1(-93.9 to 86.1) |
| **Togo** | 40006878(23614442 to 63531119) | 36856(4395 to 126923) | 2742139(340900 to 9149880) |  | 309.8(138.1 to 754.8) | 2734.4(264.6 to 43713.5) |  | -61.3(-67.2 to -52.9) | -47.3(-87.3 to 128.8) | -65.0(-91.6 to 52.8) |
| **Tokelau** | 1898435(519853 to 2899592) | 2627(131 to 13826) | 14276(795 to 71329) |  | 189.0(48.5 to 1020.6) | 780.9(-41.3 to 20155.5) |  | 7.1(-10.7 to 40.4) | 85.9(-67.6 to 938.7) | 22.4(-78.8 to 565.3) |
| **Tonga** | 633659(417575 to 958218) | 856(71 to 3780) | 4647(395 to 20884) |  | 52.9(1.9 to 333.2) | 387.7(-63.7 to 9506.6) |  | -30.5(-38.0 to -22.6) | 20.3(-80.0 to 534.1) | -20.8(-87.0 to 311.7) |
| **Trinidad and Tobago** | 3200200(2052042 to 4835021) | 6785(525 to 30587) | 41359(3383 to 173933) |  | -24.9(-33.1 to -15.5) | -45.5(-94.6 to 490.4) |  | -16.1(-24.3 to -7.5) | 83.7(-64.4 to 907.6) | 17.3(-77.0 to 550.2) |
| **Tunisia** | 191418(109789 to 299143) | 473(93 to 1454) | 6594(1419 to 19643) |  | 33.5(1.0 to 107.4) | 1744.8(187.7 to 22843.9) |  | -14.0(-26.9 to 2.5) | 46.2(-61.9 to 504.5) | -3.9(-74.9 to 298.6) |
| **Turkey** | 60188(38787 to 91485) | 440(123 to 1061) | 6165(1933 to 14669) |  | — | — |  | -35.1(-44.0 to -26.0) | 8.5(-57.0 to 164.4) | -28.0(-71.6 to 75.1) |
| **Turkmenistan** | 1344323(612216 to 2265757) | 249791(74434 to 521536) | 557417(179477 to 1149573) |  | -26.1(-51.5 to -5.6) | 14224.8(3403.7 to 145329.5) |  | -47.7(-74.1 to -13.9) | -6.8(-66.9 to 127.4) | -41.7(-79.1 to 41.8) |
| **Tuvalu** | 2986245(595631 to 4680601) | 4127(178 to 19759) | 22062(1127 to 94274) |  | 278.7(77.5 to 1307.8) | 1102.2(-22.3 to 27208.5) |  | 17.3(-14.7 to 76.7) | 93.3(-66.8 to 1324.6) | 27.9(-77.8 to 824.7) |
| **Uganda** | 144692054(108775880 to 180839539) | 173066(29190 to 512875) | 12901411(2191734 to 36460607) |  | -60.8(-69.6 to -47.8) | 2085.7(427.6 to 26095.1) |  | -72.0(-77.6 to -64.0) | -39.3(-86.9 to 67.1) | -59.2(-91.2 to 11.7) |
| **Ukraine** | 2320055(968221 to 4391417) | 964894(414964 to 1734625) | 2165732(962607 to 3682968) |  | 114.0(16.7 to 214.1) | 6555.4(1668.3 to 56475.5) |  | -48.7(-77.2 to 1.0) | 15.3(-40.6 to 143.7) | -27.3(-63.5 to 54.0) |
| **United Arab Emirates** | 247100(114932 to 434631) | 778(51 to 3623) | 11711(867 to 51844) |  | 46.8(3.2 to 238.8) | 1032.3(18.1 to 14249.3) |  | 39.7(-19.2 to 93.5) | 181.1(-58.3 to 1509.6) | 81.1(-73.2 to 921.5) |
| **United Kingdom** | 216434(135628 to 336652) | 1761(444 to 4626) | 6801(1747 to 17132) |  | -66.2(-68.2 to -63.9) | -52.5(-81.4 to 31.8) |  | -29.0(-33.7 to -24.0) | 2.1(-59.8 to 123.7) | -34.6(-74.6 to 42.7) |
| **United Republic of Tanzania** | 100136298(62809811 to 136204360) | 93202(12336 to 332037) | 6975194(925949 to 23960459) |  | -18.5(-41.8 to 34.4) | 2348.0(327.7 to 37270.2) |  | -66.7(-74.0 to -59.7) | -14.4(-78.6 to 140.9) | -42.7(-85.8 to 61.3) |
| **United States of America** | 159191(103503 to 236248) | 1391(348 to 3778) | 5351(1414 to 13758) |  | -86.6(-88.0 to -85.0) | -94.6(-96.9 to -89.8) |  | -11.4(-17.2 to -6.3) | 42.0(-43.5 to 207.3) | -9.3(-63.9 to 96.3) |
| **United States Virgin Islands** | 851714(549238 to 1266953) | 1617(147 to 6360) | 10025(959 to 41937) |  | -42.4(-49.0 to -32.4) | -60.3(-96.3 to 372.6) |  | -0.9(-12.2 to 9.9) | 113.1(-57.6 to 1048.1) | 36.5(-72.7 to 635.9) |
| **Uruguay** | 1971853(1260921 to 2927972) | 4115(604 to 13801) | 15904(2417 to 52568) |  | -11.4(-34.3 to 30.8) | 132.3(-67.3 to 1952.3) |  | -17.7(-26.6 to -8.4) | 44.3(-71.8 to 492.5) | -7.1(-82.0 to 279.1) |
| **Uzbekistan** | 521488(195776 to 996554) | 156770(56437 to 305382) | 350116(127483 to 640414) |  | -22.9(-52.4 to 6.9) | 29927.2(7833.2 to 397211.3) |  | -35.3(-70.8 to 7.4) | -22.5(-65.9 to 27.8) | -51.6(-78.9 to -19.8) |
| **Vanuatu** | 3079843(422878 to 6977025) | 2703(46 to 15760) | 14601(334 to 88152) |  | 101.5(-41.5 to 968.0) | 666.7(-66.8 to 19517.8) |  | 19.0(-43.1 to 210.3) | 180.5(-62.0 to 2127.4) | 78.8(-74.1 to 1311.5) |
| **Venezuela (Bolivarian Republic of)** | 2881064(1808880 to 4370053) | 22562(2172 to 85250) | 137881(14551 to 480368) |  | -47.3(-55.8 to -40.9) | 503.9(-27.3 to 7655.0) |  | 2.2(-12.5 to 22.7) | 43.5(-69.8 to 584.3) | -7.0(-80.4 to 343.8) |
| **Viet Nam** | 9510245(6047069 to 13050680) | 181281(44475 to 483596) | 959347(238958 to 2477868) |  | 802.4(474.6 to 1146.6) | 10301.4(2715.6 to 59323.8) |  | -31.4(-47.1 to -13.9) | -15.2(-76.9 to 111.3) | -45.0(-85.0 to 37.2) |
| **Yemen** | 556947(130372 to 1241429) | 2233(139 to 10544) | 31494(2318 to 148135) |  | -37.1(-75.4 to 186.0) | 883.8(77.2 to 20298.2) |  | -28.3(-63.8 to -4.9) | -5.0(-85.0 to 229.8) | -36.6(-89.4 to 117.0) |
| **Zambia** | 300220550(196429200 to 377233719) | 388339(54202 to 1288428) | 28912759(4194745 to 92857983) |  | 39.1(-6.1 to 111.1) | 1286.0(207.4 to 13743.0) |  | -55.8(-66.9 to -46.5) | 49.1(-66.8 to 315.5) | -0.4(-77.8 to 178.0) |
| **Zimbabwe** | 342288463(246846061 to 402184287) | 604279(76506 to 1789772) | 44913961(6285833 to 132631308) |  | 237.6(75.6 to 598.1) | 4992.1(477.9 to 88761.7) |  | -70.5(-77.3 to -62.6) | -52.0(-87.9 to 105.1) | -67.7(-91.9 to 37.5) |

**S17**

| **Group** | **Number of Deaths (age-standardized, both sexes) with 95% uncertainty intervals (2019)** | | |  | **Annualized rate of change of age-standardized rate (%) with 95% uncertainty intervals** | | | | | |
| --- | --- | --- | --- | --- | --- | --- | --- | --- | --- | --- |
|  |  |  |  |  | **1990-2010** | |  | **2010-2019** | | |
|  | **HIV/AIDS - Drug-susceptible Tuberculosis** | **HIV/AIDS - Extensively drug-resistant Tuberculosis** | **HIV/AIDS - Multidrug-resistant Tuberculosis without extensive drug resistance** |  | **HIV/AIDS - Drug-susceptible Tuberculosis** | **HIV/AIDS - Multidrug-resistant Tuberculosis without extensive drug resistance** |  | **HIV/AIDS - Drug-susceptible Tuberculosis** | **HIV/AIDS - Extensively drug-resistant Tuberculosis** | **HIV/AIDS - Multidrug-resistant Tuberculosis without extensive drug resistance** |
| **Global** | 244923(173251 to 319002) | 998(429 to 1825) | 24454(10229 to 45371) |  | 129.0(78.1 to 203.7) | 3334.7(1888.3 to 5538.6) |  | -52.1(-55.2 to -48.9) | -19.0(-34.5 to 1.6) | -44.2(-59.9 to -21.9) |
| **Afghanistan** | 1127(481 to 2006) | 11(1 to 38) | 141(18 to 495) |  | -37.3(-56.1 to 211.0) | 3243.2(567.1 to 59683.4) |  | -46.9(-69.6 to -29.8) | -46.7(-89.7 to 60.6) | -65.0(-93.2 to 5.8) |
| **Albania** | 1122(624 to 1886) | 10(1 to 33) | 22(3 to 74) |  | -46.3(-70.7 to -6.0) | 260.6(-31.6 to 3060.0) |  | 14.6(1.8 to 29.9) | -11.8(-82.5 to 182.3) | -44.8(-89.1 to 75.4) |
| **Algeria** | 2116(1085 to 3443) | 9(1 to 40) | 120(9 to 530) |  | -16.8(-38.3 to 20.9) | 8.2(-85.3 to 492.8) |  | -36.3(-48.3 to -18.7) | 17.9(-77.8 to 326.5) | -22.1(-85.4 to 181.0) |
| **American Samoa** | 2600(1587 to 4040) | 4(0 to 18) | 22(2 to 93) |  | -7.3(-23.2 to 87.1) | 197.7(-81.4 to 4155.3) |  | -16.9(-26.9 to -5.8) | 43.0(-74.0 to 745.8) | -6.6(-83.2 to 447.2) |
| **Andorra** | 5760(3495 to 8819) | 10(1 to 46) | 37(3 to 184) |  | -30.9(-43.1 to -13.1) | 3.7(-90.8 to 998.6) |  | -5.0(-17.9 to 7.8) | 11.9(-83.0 to 547.5) | -28.2(-89.1 to 313.9) |
| **Angola** | 1183972(697044 to 1900625) | 1323(129 to 4765) | 97203(9848 to 336783) |  | 1567.7(559.9 to 3733.7) | 15711.4(1419.8 to 236568.6) |  | -38.8(-50.5 to -21.0) | 0.5(-77.1 to 352.9) | -33.1(-84.8 to 201.3) |
| **Antigua and Barbuda** | 8808(5456 to 13568) | 24(2 to 104) | 146(10 to 585) |  | -72.1(-74.6 to -69.0) | -83.9(-98.5 to 71.2) |  | -21.9(-31.0 to -11.1) | 64.8(-68.4 to 1029.9) | 7.0(-79.4 to 633.7) |
| **Argentina** | 153717(115787 to 166175) | 1428(170 to 5451) | 5425(618 to 20006) |  | 115.9(82.3 to 129.1) | 968.0(118.5 to 8235.0) |  | -8.5(-22.6 to -0.6) | 3.4(-78.9 to 196.8) | -33.5(-86.5 to 89.9) |
| **Armenia** | 10953(4191 to 18394) | 2034(467 to 4602) | 4439(1078 to 9911) |  | 483.1(265.4 to 667.9) | 100133.2(23067.8 to 1262114.2) |  | 23.1(-45.6 to 107.1) | 68.4(-48.0 to 239.9) | 4.8(-67.7 to 111.2) |
| **Australia** | 1403(784 to 2399) | 31(8 to 85) | 119(30 to 314) |  | -78.3(-81.9 to -74.1) | -7.7(-78.9 to 458.6) |  | -13.2(-30.5 to 6.3) | 136.2(-33.7 to 695.0) | 51.2(-57.6 to 408.7) |
| **Austria** | 3481(2084 to 5680) | 76(19 to 193) | 289(74 to 714) |  | -83.2(-85.9 to -80.3) | 41.4(-61.6 to 1169.1) |  | -20.1(-31.5 to -7.9) | -12.2(-66.1 to 87.9) | -43.6(-78.3 to 20.2) |
| **Azerbaijan** | 5724(2347 to 10207) | 1296(348 to 2643) | 2815(833 to 5601) |  | 3.7(-54.3 to 347.9) | 26476.7(5195.6 to 518534.2) |  | -38.8(-71.1 to 10.4) | 11.2(-64.3 to 163.8) | -31.2(-77.8 to 63.9) |
| **Bahamas** | 80537(47920 to 126852) | 542(52 to 2123) | 3269(353 to 11965) |  | -52.6(-58.5 to -46.2) | 448.1(-2.6 to 5655.8) |  | -18.5(-30.5 to -7.4) | 75.4(-74.7 to 707.7) | 14.2(-83.6 to 424.7) |
| **Bahrain** | 2519(1456 to 4018) | 15(2 to 50) | 207(28 to 661) |  | -66.9(-71.5 to -61.6) | 626.1(51.7 to 8055.0) |  | -27.0(-41.0 to -14.2) | 14.5(-82.2 to 308.1) | -24.6(-88.3 to 168.9) |
| **Bangladesh** | 5984(94 to 28314) | 36(0 to 234) | 697(7 to 4785) |  | — | — |  | -2.6(-70.9 to 101.8) | 101.8(-72.3 to 568.6) | 32.3(-81.7 to 342.1) |
| **Barbados** | 12225(7440 to 18718) | 19(1 to 96) | 112(6 to 565) |  | -69.8(-73.4 to -64.8) | -92.6(-99.5 to 34.4) |  | -17.3(-27.2 to -6.5) | 85.3(-76.6 to 1329.4) | 19.4(-84.8 to 828.0) |
| **Belarus** | 4932(1882 to 10301) | 3732(1855 to 6402) | 8125(4374 to 13423) |  | 35.1(-25.8 to 104.2) | 5762.9(1181.4 to 54110.6) |  | -69.3(-84.1 to -52.0) | -40.1(-54.0 to -21.7) | -62.7(-71.4 to -51.3) |
| **Belgium** | 5444(3212 to 8582) | 66(13 to 192) | 249(53 to 735) |  | -58.1(-62.8 to -52.6) | -3.8(-70.9 to 462.3) |  | -42.3(-50.5 to -33.5) | -29.4(-77.1 to 74.8) | -54.9(-85.4 to 11.8) |
| **Belize** | 148685(89941 to 229518) | 307(24 to 1355) | 1854(150 to 7950) |  | -44.3(-50.0 to -37.7) | -62.2(-96.1 to 306.6) |  | -30.3(-37.2 to -23.0) | 63.2(-69.0 to 838.2) | 6.2(-79.8 to 509.4) |
| **Benin** | 525657(306867 to 807975) | 394(50 to 1275) | 28759(4175 to 89712) |  | 1388.2(572.2 to 3059.6) | 13202.7(2586.0 to 130657.4) |  | -16.4(-29.4 to -4.9) | 57.3(-68.8 to 355.9) | 4.1(-79.2 to 203.3) |
| **Bermuda** | 2618(1567 to 4005) | 6(0 to 35) | 35(2 to 217) |  | -80.7(-82.6 to -78.3) | -90.8(-99.5 to 12.9) |  | -10.6(-23.2 to 3.8) | 194.3(-66.1 to 2048.9) | 94.9(-78.0 to 1295.2) |
| **Bhutan** | 9465(2676 to 19054) | 59(4 to 237) | 1128(86 to 4028) |  | -23.4(-70.9 to 678.6) | 850.3(-15.7 to 44577.7) |  | -29.4(-48.9 to 16.7) | 11.1(-71.2 to 401.9) | -26.7(-81.0 to 231.9) |
| **Bolivia (Plurinational State of)** | 36691(12552 to 67728) | 786(109 to 2534) | 4758(633 to 14490) |  | -34.2(-74.3 to 420.3) | 191.4(-69.7 to 9069.4) |  | -53.8(-72.1 to -36.1) | 10.3(-69.4 to 398.1) | -27.5(-80.1 to 223.5) |
| **Bosnia and Herzegovina** | 1552(1045 to 2343) | 8(1 to 25) | 18(3 to 55) |  | -45.3(-57.6 to -28.5) | 9.6(-75.0 to 643.4) |  | -13.8(-34.4 to 17.0) | 0.1(-74.3 to 190.5) | -37.8(-84.0 to 80.4) |
| **Botswana** | 6401450(3415363 to 10028892) | 11375(1546 to 35098) | 837498(117003 to 2636248) |  | 168.0(44.5 to 383.9) | 3611.7(847.2 to 30082.2) |  | -49.7(-61.5 to -38.1) | 12.2(-72.8 to 190.8) | -25.0(-81.9 to 93.5) |
| **Brazil** | 71883(43645 to 111025) | 898(133 to 2857) | 5407(757 to 17290) |  | -41.4(-49.4 to -28.8) | 2682.5(509.6 to 30597.0) |  | -30.4(-40.0 to -23.6) | 41.4(-65.8 to 241.0) | -8.1(-77.8 to 121.5) |
| **Brunei Darussalam** | 7633(4770 to 11721) | 18(2 to 72) | 66(7 to 254) |  | -20.7(-32.9 to -5.0) | 30.8(-83.2 to 1388.1) |  | 10.2(-0.4 to 21.6) | 164.2(-61.6 to 1365.9) | 68.5(-75.4 to 837.6) |
| **Bulgaria** | 604(369 to 945) | 16(3 to 43) | 35(8 to 101) |  | 7.4(-9.7 to 24.7) | 1700.4(331.2 to 16488.1) |  | -31.2(-42.4 to -18.9) | -47.5(-85.4 to 26.3) | -67.1(-90.9 to -21.6) |
| **Burkina Faso** | 705657(518019 to 901141) | 789(90 to 2559) | 58284(7079 to 187665) |  | -66.3(-75.6 to -56.8) | 204.6(-55.6 to 3808.9) |  | -62.5(-69.5 to -53.1) | -47.3(-88.1 to 138.7) | -64.8(-92.1 to 58.8) |
| **Burundi** | 982798(712266 to 1287038) | 1192(126 to 4365) | 87659(10335 to 303083) |  | -16.7(-61.4 to 95.1) | 2336.5(163.7 to 37701.1) |  | -79.4(-84.5 to -70.6) | -58.3(-89.4 to 62.0) | -72.3(-92.9 to 7.8) |
| **Cabo Verde** | 105499(61566 to 174958) | 140(13 to 516) | 10205(1018 to 34702) |  | -30.8(-51.7 to 30.2) | 520.6(-21.5 to 7734.6) |  | -51.9(-60.4 to -40.4) | -23.8(-81.7 to 239.9) | -49.7(-87.8 to 126.1) |
| **Cambodia** | 286469(186036 to 404848) | 1088(108 to 4220) | 5686(574 to 21533) |  | 22798.8(8684.9 to 62202.6) | 368015.9(64373.7 to 5812119.2) |  | -64.1(-69.6 to -57.7) | -68.4(-94.1 to 0.6) | -79.6(-96.2 to -34.9) |
| **Cameroon** | 1905635(1010037 to 3111736) | 1711(175 to 6495) | 124851(14289 to 455352) |  | 607.7(237.3 to 1359.6) | 4992.4(383.1 to 64185.9) |  | -53.8(-60.5 to -46.0) | -34.1(-84.6 to 176.6) | -56.3(-89.8 to 84.0) |
| **Canada** | 5906(3615 to 9301) | 49(12 to 134) | 187(45 to 513) |  | -75.8(-78.6 to -72.2) | -67.8(-88.2 to 6.4) |  | 1.5(-11.9 to 16.0) | 51.7(-45.4 to 261.6) | -2.6(-65.1 to 131.4) |
| **Central African Republic** | 6124655(4848410 to 8026283) | 3370(415 to 10867) | 250545(30365 to 845089) |  | 237.6(113.6 to 403.7) | 1554.1(111.4 to 16877.5) |  | -46.9(-56.4 to -35.7) | -8.9(-78.9 to 302.4) | -38.9(-85.9 to 167.7) |
| **Chad** | 1818839(1252238 to 2458020) | 1867(209 to 6432) | 137816(15799 to 470896) |  | 198.5(40.4 to 478.3) | 2253.5(145.5 to 41348.9) |  | -5.8(-25.7 to 22.8) | 30.4(-71.2 to 448.0) | -13.3(-80.8 to 264.6) |
| **Chile** | 100442(82369 to 105896) | 719(194 to 1912) | 2743(757 to 7360) |  | 188.2(171.6 to 201.6) | 959.5(102.6 to 8794.8) |  | -4.8(-21.1 to 2.8) | 50.0(-65.9 to 503.3) | -3.4(-78.2 to 286.0) |
| **China** | 11330(6476 to 17790) | 220(32 to 726) | 1149(165 to 3840) |  | 36.5(-21.6 to 448.3) | 152.5(-35.4 to 2137.5) |  | -11.7(-29.8 to 4.2) | -2.8(-76.9 to 150.2) | -36.8(-85.1 to 61.9) |
| **Colombia** | 32572(19152 to 51114) | 605(84 to 1849) | 3667(542 to 11080) |  | -28.2(-40.8 to -11.3) | 1104.8(146.3 to 9782.7) |  | -27.0(-41.5 to -13.9) | 27.2(-66.8 to 194.4) | -17.1(-78.5 to 91.2) |
| **Comoros** | 7990(122 to 70923) | 22(0 to 171) | 1624(12 to 13176) |  | 1057.7(-42.7 to 88432.9) | 47752.1(935.7 to 8488375.7) |  | 4.0(-67.4 to 356.9) | 204.9(-41.2 to 2880.7) | 105.4(-60.8 to 1882.9) |
| **Congo** | 1466211(818245 to 2411906) | 1194(113 to 4583) | 86792(8912 to 360844) |  | -47.9(-60.5 to -23.0) | 187.2(-74.4 to 3653.6) |  | -46.8(-54.6 to -39.1) | 5.7(-77.1 to 400.1) | -30.0(-84.8 to 232.7) |
| **Cook Islands** | 14341(5554 to 22449) | 21(1 to 103) | 108(7 to 543) |  | 180.1(88.5 to 609.7) | 745.7(-36.2 to 12942.2) |  | 35.6(10.0 to 59.7) | 142.1(-57.7 to 1316.7) | 57.4(-72.7 to 816.6) |
| **Costa Rica** | 12828(7836 to 20251) | 116(13 to 445) | 696(82 to 2867) |  | -60.6(-65.8 to -55.9) | 857.6(54.9 to 10853.2) |  | -28.1(-39.2 to -15.0) | 14.2(-74.1 to 243.4) | -25.5(-83.2 to 123.1) |
| **Croatia** | 1824(1098 to 2939) | 8(1 to 25) | 17(3 to 55) |  | -33.9(-61.1 to 6.6) | 33.3(-70.9 to 883.6) |  | -48.2(-54.7 to -41.1) | -53.7(-88.3 to 44.8) | -71.0(-92.7 to -10.1) |
| **Cuba** | 3405(2073 to 5240) | 35(5 to 113) | 211(32 to 662) |  | -20.7(-31.6 to -8.1) | -32.1(-78.3 to 164.5) |  | 16.2(-3.1 to 32.8) | 189.0(-39.6 to 850.5) | 88.6(-60.7 to 517.4) |
| **Cyprus** | 1985(1176 to 3148) | 25(4 to 85) | 96(13 to 324) |  | -45.7(-60.4 to -23.3) | 538.7(38.7 to 6655.7) |  | 33.4(11.6 to 68.2) | -45.6(-87.8 to 52.3) | -65.3(-92.2 to -2.5) |
[truncated: 215,348 more chars]
